# ﻿DNA barcoding, integrative taxonomy, citizen science, and Bush Blitz surveys combine to reveal 34 new species of *Apanteles* (Hymenoptera, Braconidae, Microgastrinae) in Australia

**DOI:** 10.3897/zookeys.1227.130467

**Published:** 2025-02-11

**Authors:** Mollie-Rosae Slater-Baker, Erinn P. Fagan-Jeffries, Katherine J. Oestmann, Olivia G. Portmann, Tiahni M. Bament, Andy G. Howe, Michelle T. Guzik, Tessa M. Bradford, Alana R. McClelland, Alice Woodward, Sylvia Clarke, Nathan Ducker, José Fernández-Triana

**Affiliations:** 1 School of Biological Sciences, The University of Adelaide, Adelaide, Australia The University of Adelaide Adelaide Australia; 2 South Australian Museum, Adelaide, Australia South Australian Museum Adelaide Australia; 3 Forest Research Institute, University of the Sunshine Coast, Sippy Downs, Australia University of the Sunshine Coast Sippy Downs Australia; 4 Murraylands and Riverland Landscape Board, Murray Bridge, Australia Murraylands and Riverland Landscape Board Murray Bridge Australia; 5 Western Australian Gould League, Wembley, Australia Western Australian Gould League Wembley Australia; 6 Canadian National Collection of Insects, Ottawa, Canada Canadian National Collection of Insects Ottawa Canada

**Keywords:** Insect investigators, microgastrines, parasitoid wasps

## Abstract

Microgastrinae is a megadiverse subfamily of wasps in the family Braconidae. As parasitoids of caterpillars, members of the subfamily play important roles in regulating native caterpillar populations, and several species are used commercially as biological control agents. The genus *Apanteles* comprises a large portion of total microgastrine diversity, however it has not been studied in Australia for more than 30 years, with only nine described species previously known from the continent. We explore the diversity and systematics of *Apanteles* in Australia, using cytochrome c oxidase subunit I (COI) and Wingless (wg) DNA barcodes from more than 400 Australian *Apanteles* specimens. Using molecular species delimitation in combination with reduced morphological diagnoses, at least 48 distinct molecular lineages of *Apanteles* are confirmed in Australia, and 34 new species are formally described, all authored by Slater-Baker, Fagan-Jeffries, Fernández-Triana, Portmann & Oestmann: *A.adustus*, *A.aeternus*, *A.alatomicans*, *A.allapsus*, *A.amicalis*, *A.apollo*, *A.apricus*, *A.artemis*, *A.aurantius*, *A.auroralis*, *A.banrock*, *A.breviflagellarius*, *A.brockhedgesi*, *A.cuprum*, *A.darthvaderi*, *A.doreenwatlerae*, *A.ethanbeaveri*, *A.fenestrinus*, *A.ferripulvis*, *A.focusalis*, *A.hades*, *A.insulanus*, *A.kelpiellus*, *A.lamingtonensis*, *A.ligdus*, *A.magicus*, *A.margaritarius*, *A.pellucidus*, *A.phantasmatus*, *A.pharusalis*, *A.ramsaris*, *A.rufiterra*, *A.sinusulus*, and *A.translucentis*.

## ﻿﻿Introduction

*Apanteles* Foerster, 1863 is a genus of parasitoid wasps in the family Braconidae, subfamily Microgastrinae. Currently, *Apanteles* s. str. is known to contain more than 600 species and the genus occurs worldwide (Fernández-Triana et al. 2020). Contemporary descriptions of *Apanteles* s. str. have been published from China (Liu et al. 2014, 2015, 2020; Liu and Chen 2023), Mexico (Sánchez-García et al. 2015), Réunion (Rousse and Gupta 2013), Argentina (Martínez et al. 2012), Canada and Alaska (Fernández-Triana 2010), Malaysia (van Achterberg et al. 2009), Mesoamerica (Fernández-Triana et al. 2014), and South America (Whitfield et al. 2001). Australia is expected to host a rich diversity of microgastrines (Fagan-Jeffries et al. 2018), and globally *Apanteles* s. str. comprises a large portion of known microgastrines. When last studied, before the advent of DNA barcoding, it was estimated that 100–150 *Apanteles* species may be present in Australia (Austin and Dangerfield 1992). When the genus was last studied in Australia (Austin and Dangerfield 1992), 12 *Apanteles* species had been recorded for the country, including *A.subandinus* introduced for biocontrol of the potato tuber moth in the 1960s (Callan 1974). Several species (now *Choerasaper* (Nixon, 1965)), *Dolichogenideaargiope* (Nixon, 1965), and *Parapantelesfolia* (Nixon, 1965) have recently been transferred to other genera (Fernández-Triana et al. 2020), leaving only nine recognised *Apanteles* in Australia.

Members of *Apanteles* can be recognised by having the fore wing without an areolet, hypopygium flexible and pleated, relatively long ovipositor sheaths (but there are exceptions), a propodeum fully to partially areolated without a medial carina, and a concave margin of the hind wing vannal lobe (sometimes straight) (Fernández-Triana et al. 2020). The genus is relatively morphologically conserved, and characters used for separating species are often subtle (e.g., Fernández-Triana et al. 2014). This combination, of high diversity with low morphological variation among species, means *Apanteles* is an ideal candidate for applying integrated taxonomic methods to document species more efficiently.

There is much discussion in the taxonomic community about methods to streamline and accelerate species discovery, documentation, and description. Indeed, the Australian Academy of Science Taxonomy Australia program has named taxonomy acceleration as a major strategic action to facilitate biodiversity discovery and documentation (Taxonomy Decadal Plan Working Group 2018). DNA barcoding using the 658 bp region of the mitochondrial cytochrome *c* oxidase subunit I gene (COI) has been used for efficient species-level identification, and investigations of biodiversity, for more than two decades (Hebert et al. 2003; Smith et al. 2008; Srivathsan et al. 2021; Höcherl et al. 2024; Vasilita et al. 2024). The method benefits from the use of rapidly growing online public repositories for COI sequences, such as the Barcode of Life Database (BOLD; Ratnasingham and Hebert 2007) where sequences are grouped with other similar sequences using the RESL algorithm (Ratnasingham and Hebert 2013) into Operational Taxonomic Units called BINs (Barcode Index Numbers). COI barcoding can be particularly useful for tiny, mega-diverse wasps, as they often display highly conserved or reduced morphology, making morphological species delimitation challenging (Quicke et al. 2012; Smith et al. 2013; Stahlhut et al. 2013; Sharkey et al. 2021; Fernández-Triana et al. 2023). The use of the global BOLD information also allows rapid assessment of potential endemicity of species. However, the use of COI alone for molecular analyses beyond DNA barcode identification is typically considered insufficient due to biases that may arise from the use of a single gene (Ahrens et al. 2021; Meier et al. 2021; Sharkey et al. 2022; Zamani et al. 2022). This is especially important to consider when using molecular data as a major line of evidence for species delimitation.

Here, we take a DNA-first approach supported by minimal morphology, where we use DNA barcodes as a ‘first-pass’ to group specimens into potential species based on several molecular species delimitation methods. We then use minimal morphology to provide supporting evidence for those species and highlight molecular species that require further examination. For microgastrine wasps, the use of a 2% COI difference threshold for species delimitation has been particularly well-established owing to the large amount of DNA barcoding data generated from parasitoid wasp rearing in the Area de Conservación Guanacaste in Costa Rica. These studies provide evidence that a ~ 2% COI threshold resolves morphologically or ecologically distinct species in the majority of cases (Smith et al. 2013; Fernández-Triana et al. 2023). There has been much discussion in the taxonomic community about the use of COI DNA barcodes as the primary evidence for recognising and describing species of braconid wasps, and here we take a slightly conservative approach explained in detail in the methods section.

In the present study, the diversity of Australian *Apanteles* is explored using a high-throughput DNA barcoding approach, including integrative species delimitation using molecular data and morphological evidence. Using specimens from a variety of sources including institutional bulk specimen archives, national surveys, and the schools-based citizen science program, ‘Insect Investigators’, 34 new species are described from Australia, with COI and Wingless (*wg*) DNA barcodes and images provided of each species. This study aims to contribute to the ‘Taxonomy Australia’ mission of documenting Australia’s biodiversity in an accelerated manner (Taxonomy Decadal Plan Working Group 2018).

## ﻿﻿Materials and methods

### ﻿﻿Specimens and sampling

A total of 389 Australian *Apanteles* specimens were obtained for use in the present study (Fig. 2, detailed specimen information is available in Suppl. material 3). The majority of this material was sourced from existing bulk ethanol collections in Australian museums, highlighting the value of these public resources. A number of specimens were collected as part of Bush Blitz surveys; Bush Blitz is Australia’s largest nature discovery program and aims to bring together taxonomists, land owners, rangers, Traditional Owners, and teachers to document flora and fauna across Australia (https://bushblitz.org.au/). Specimens used for morphological examination were mounted on card points, whilst representatives of most species were also kept in ethanol for ease of future genomic analysis. Many specimens have minor damage from storage, handling, and DNA barcoding.

Multiple specimens were contributed by the ‘Insect Investigators’ citizen science project (insectinvestigators.com.au), in which 50 regional schools in Australia collected arthropods using Malaise traps during March 2022. Students located and ran Malaise traps on or close to their schools, and sent trap contents to the University of Adelaide. Students participated in workshops, and teachers were provided additional lesson plans about entomology, DNA barcoding, and taxonomy, alongside receiving regular updates from the scientific team as their specimens were processed. We engaged students to name undescribed species caught at their schools through a two-step iterative process. Firstly, 1a) taxonomists supplied students with background information on the genus, including the distribution of known specimens, morphological features specific to the undescribed species, and a supporting video which explained wasp biology, scientific naming rules and instructions for naming; 1b) school teachers facilitated students to brainstorm names, describe reasons behind names, and submit two or three candidate names to an electronic notice board (Padlet). Secondly, 2a) taxonomists subsequently, or in real-time, commented on students’ candidate names, and provided Latin epithets and further guidance, after which 2b) teachers facilitated students to reach a consensus name, using a variety of self-determined voting methods.

### ﻿﻿DNA extraction and sequencing

DNA barcodes of COI and *wg* from Australian *Apanteles* specimens were obtained either from BOLD (see below), were sequenced for COI only as part of the ‘Insect Investigators’ project, or were newly sequenced either using the methods below or standard Sanger sequencing methods.

Specimens newly sequenced using high-throughput barcoding underwent DNA extraction using a modified version of the Canadian Centre for DNA Barcoding Glass Fibre Plate DNA Extraction Protocol (Ivanova et al. 2006). Library preparation for Illumina Miseq high-throughput sequencing was performed (Cruaud et al. 2017), for sequencing by the Australian Genome Research Facility (AGRF). Sequencing and bioinformatics followed Fagan-Jeffries et al. (2018), other than the modifications as follows. Whole bodies were used for DNA extraction and then removed from lysis as vouchers. Round one of PCR amplification was performed using the following reagents, totalling 12.5 μL: 7.75 μL nuclease free water, 2.5 μL MRT buffer (1 × Immolase buffer/1.5 mM MgCl2/0.8 mM dNTP), 0.6 μL of each forward (*wg*: LepWG1, Brower and Desalle 1998; COI: LCO1490, Folmer et al. 1994) and reverse (*wg*: LepWG2, (Brower and Desalle 1998); COI: III_C_R, (Shokralla et al. 2015)) primer, 0.05 μL Immolase (Bioline; NSW, Australia), and 1 μL of extracted DNA. PCR reactions were run as follows: 95 °C for 10 mins; 35 cycles of 95 °C for 45 s, then 48 °C for 45 s, then 72 °C for 1 min; and 72 °C for 10 mins. Round two of PCR amplification was performed using the following reagents, totalling 12.5 μL: 6.95 μL nuclease free water, 2.5 μL MRT buffer, 0.5 μL each of two 8-bp indexing primers (Meyer and Kircher 2010), 0.05 μL Immolase, and 2 μL of product from PCR one. Addition of indexing primers and PCR product were performed using an Eppendorf epMotion 5075 robot. Round two PCR reactions were run as follows: 95 °C for 10 mins; 7 cycles of 95 °C for 45 s, then 65 °C for 45 s, then 72 °C for 1 min; and 72 °C for 10 mins.

### ﻿﻿DNA sequence datasets

Two datasets were used in the study:

#### ﻿﻿Dataset 1: Australian *Apanteles*

The first dataset, used for molecular species delimitation analysis and construction of a phylogeny, included 623 *Apanteles* specimens, 269 of which were represented by both COI and *wg*, three that were only represented by *wg* and 351 which were represented by only COI. This dataset was constructed with:

All of the Australian specimens we identified as belonging to the genus *Apanteles* (either identified morphologically, or through their placement in a Neighbour Joining COI tree of Australian Microgastrinae) that were newly sequenced as part of the current project, or sequenced as part of previous Australian microgastrine DNA barcoding studies (e.g., Fagan-Jeffries et al. 2018).

Any other sequence from Australia that was available on BOLD as of February 2024, and was either identified as *Apanteles*, Microgastrinae or Braconidae, and that fell within the *Apanteles* clade in a Neighbour Joining tree. The majority of these sequences are from a single location in Queensland that was part of the Global Malaise Trap Program.

Any sequences from countries outside Australia that were in the same BIN as a sequence from Australia as of February 2024. These sequences were included to determine if there were patterns within the BINs that the species delimitation methods could verify.

All available sequences of species reported to occur in Australia according to Fernández-Triana et al. (2020) (or Austin and Dangerfield (1992) in the case of *A.galleriae*) that were not yet included using the criteria above, which included sequences of *A.carpatus*, *A.subandinus*, and *A.galleriae* from countries outside of Australia.

For this dataset, ten representatives from other microgastrine genera (*Choeras*, *Cotesia*, *Dolichogenidea*, *Glyptapanteles*, *Iconella*, and *Microplitis*) that had both COI and *wg* sequences were included as outgroups. The trees were rooted at *Microplitisdemolitor* based on previous phylogenomic results (Jasso-Martínez et al. 2022).

#### ﻿﻿Dataset 2: Global *Apanteles*

The second dataset was used to examine the relationships among Australian and global species. For this dataset, the COI sequences in Dataset 1 (Australian *Apanteles* and non-Australian members of BINs with an Australian representative) were combined with all sequences available on BOLD as of February 2024 that were identified as *Apanteles* (with those flagged by BOLD as contaminants or misidentifications removed). Duplicate identical sequences were then removed to reduce the size of the dataset to 3480 sequences (from an original 7233). A sequence of *Microplitisdemolitor* was used as the outgroup at which the tree was rooted.

We used BOLD as the primary database for building these datasets as it contains a much larger proportion of the publicly available COI sequences of insects compared to other DNA archives. However, in an effort to ensure we were not missing critical data, two checks were performed on the NCBI database: 1) The NCBI nucleotide database was searched with the terms “Apanteles Australia” and also with “Microgastrinae Australia”, but there were no additional sequences identified as *Apanteles* from Australia available; and 2) the COI sequence for the holotype of each newly described species was compared to the NCBI nucleotide database using BLASTN 2.16.0 (Zhang et al. 2000) to ensure there were no other closely related (<3% divergent) sequences available.

### ﻿﻿Sequence alignments

Sequences were aligned in Geneious Prime 2023.0.4 (https://www.geneious.com) using MAFFT v. 7.490 (Katoh et al. 2002; Katoh and Standley 2013) with default settings, to give four alignments for phylogenetics and species delimitation:

- concatenation of COI and *wg* for Australian *Apanteles* (Dataset 1)
- COI only for Australian *Apanteles* (Dataset 1)
- *wg* only for Australian *Apanteles* (Dataset 1)
- COI for global *Apanteles* (Dataset 2)

### ﻿﻿Phylogenetic frameworks for species delimitation

Phylogenetic analyses were performed for use as inputs for species delimitation methods, and to provide a framework to guide morphological exploration of Australian *Apanteles*. Tree appearance was modified using a combination of FigTree 1.4.4 (http://tree.bio.ed.ac.uk/software/figtree/), Inkscape 1.1.2 (https://inkscape.org/) and Adobe Photoshop 2024 software.

### ﻿﻿Maximum likelihood (ML) analyses

Phylogenies were produced for alignments A, B, and C using the web implementation of IQ-TREE 1.6.12 (Nguyen et al. 2015; Trifinopoulos et al. 2016). Best-fit substitution models were selected using the inbuilt ModelFinder (Kalyaanamoorthy et al. 2017) option, with partitioning by codon positions (with separate partitions for each gene for the concatenated alignment), and partition merging enabled. Best-fit models were input for tree building (A,B = GTR+I+G4+F, C = TPM2u = F = G4), using Ultrafast bootstrapping (Hoang et al. 2018), with 1000 replicates to assess branch support, and 1000 replicates for SH-aLRT.

#### ﻿﻿Visualising global relatedness among *Apanteles*

A phylogeny of Dataset 2 (Alignment D) was created using IQ-TREE 2.3.3 (Nguyen et al. 2015; Minh et al. 2020), with partitioning and models selected by the inbuilt ModelFinder and Ultrafast bootstrapping was run for 2000 replicates and convergence established. The resulting tree was visualised and edited using iTOL (Letunic and Bork 2024) and Adobe Acrobat.

### ﻿﻿Molecular species delimitation analyses

#### ﻿﻿Threshold methods

A 2% COI genetic distance threshold was implemented through the program SpeciesIdentifier v. 1.8.2 (Meier et al. 2006), clustering sequences into Molecular Operational Taxonomic Units (MOTUs) at 2% difference, using Kimura 2 parameter (K2P) corrected pairwise distances. SpeciesIdentifier was also used to cluster *wg* pairwise distances at 0% using Kimura 2 parameter (K2P) corrected pairwise distances to infer the number of *wg* haplotypes – this method has previously been used for species delimitation in microgastrines (Fagan-Jeffries et al. 2018).

#### ﻿﻿Distance based method

The web implementation of Assemble Species by Automatic Partitioning (ASAP) (Puillandre et al. 2021) was used for individual COI and *wg* alignments with the outgroup removed, with K2P substitution model selected and all other settings default. Partitions with the lowest ASAP score were selected for inclusion in consensus determination, but were first assessed with other highly ranked partitions based on their probability values and congruence with other results.

#### ﻿﻿Tree-based method

The web implementation of the Poisson Tree Process (PTP; Zhang et al. 2013: https://mptp.h-its.org/#/tree) was used, inputting IQ-TREE phylogenies for COI and *wg*, with options to crop the outgroup selected. All other settings were left default.

### ﻿﻿Imaging

Specimens were imaged using a Canon 5DS R with a MP-E 65mm 1–5× lens attached to a StackShot automontage system. Zerene Stacker v. 1.04 (http://zerenesystems.com/cms/stacker) was used for focus-stacking to produce the final images.

### ﻿﻿Species delimitation and designation

Species are defined in the present study according to the general lineage species concept (de Queiroz 1998), using a combination of molecular and morphological evidence, with an additional requirement of monophyly on a concatenated COI + *wg* phylogeny. Species delimitation for Australian *Apanteles* was performed using the four methods for the COI gene (BINS, 2% divergence, ASAP, and PTP) and three methods for the wingless gene (haplotypes, ASAP, and PTP) detailed above, to allow for assessment of congruence. The results of these analyses were compared to form a consensus delimitation hypothesis representing the most well-supported species groupings according to a majority rule.

### ﻿﻿Morphological examination

**Figure 1.**
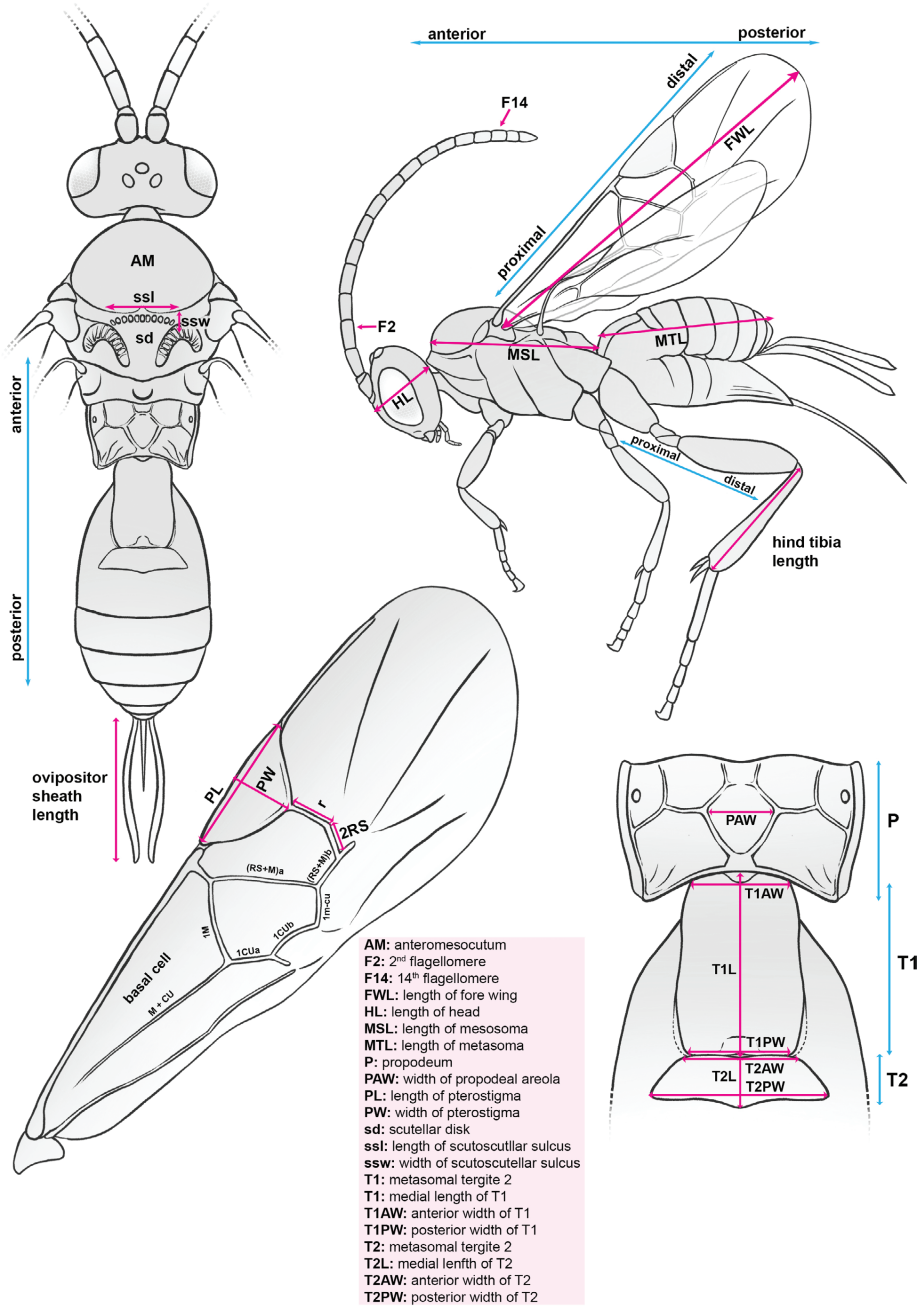
Key morphological terms and measurements used in the key and descriptions of *Apanteles* species.

Available specimens for each consensus species were examined using an Olympus SZX16 microscope. Measurements were conducted using the Olympus cellSens software following Fig. 1. Morphological characters were coded and measured only for holotypes, but all available paratypes were informally examined to ensure sequence contaminants or large variations in key characters were not present. A moderate selection of characters was coded for all species to create a baseline and identify potentially useful diagnostic characters. These data are presented in Suppl. material 3, but should be treated with some caution as many are inherently subjective and may vary slightly amongst members of the species.

Using these data alongside additional characters where needed, a key to species was constructed focussing on characters that were unambiguous and thought to be easy for non-experts to use when identifying *Apanteles* specimens. The characters in the key alongside a curated selection of other information form the brief diagnostic description for each species based on the holotype female, with any significant variation noticed in paratypes added where relevant. We do not present exhaustive, uniform descriptions, because we feel these are unlikely to be useful to either future taxonomists or applied entomologists attempting to use morphology to identify species of microgastrines. We supplement these brief diagnoses with multiple images of each species and DNA data, allowing various ways for future users to identify specimens or place new species in the framework of this revision.

**Figure 2.**
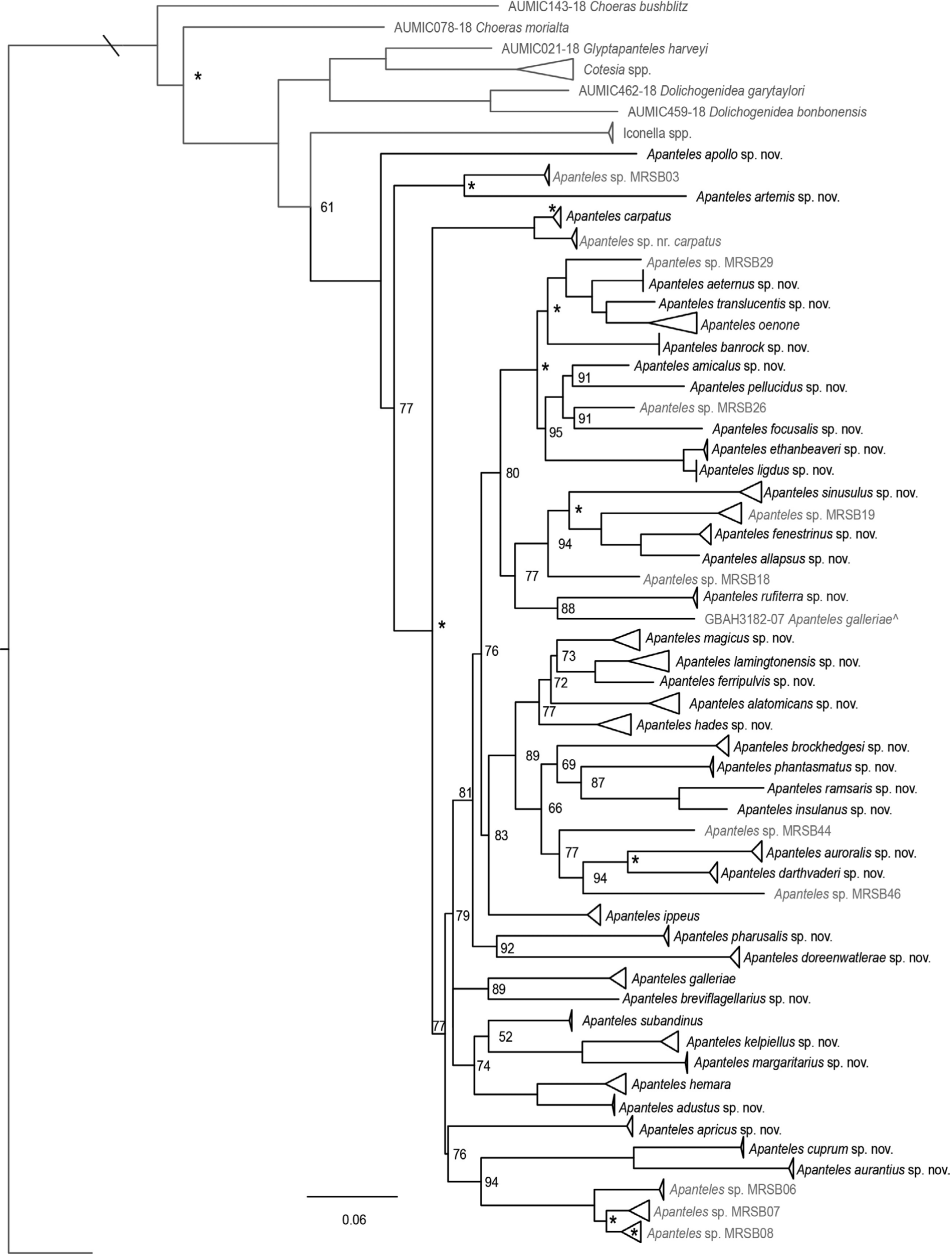
IQ-TREE extended majority rule consensus tree of the COI + *wg* concatenated alignment from Dataset 1 (Australian *Apanteles* specimens, sequences sharing a BIN with Australian material, and other species reported to occur in Australia). Ultrafast bootstrap support is shown on nodes where support was < 95, nodes where support was 95–99 are represented by an asterisk. Nodes without bootstrap support shown had support of 100. Outgroups, and unnamed species delimited by the molecular consensus but not described, are in grey. The tree is rooted with *Microplitisdemolitor* but cropped for clearer visualisation.

Due to the large number of *Apanteles* described worldwide, it was not feasible to morphologically diagnose new Australian species against all described *Apanteles*. To make the project logistically feasible, alongside evidence from within the study that there is a very high degree of species-level endemism in Australia, putative species were only morphologically diagnosed against the nine described species known from Australia. The only exceptions to this were when the delimited molecular lineage contained sequences from elsewhere in Australasia, in which case diagnoses were also made with reference to species described from the relevant country. This occurred once for a species which shares a BIN with a specimen from Fiji (although the record on BOLD was only made public after delimitation analyses were conducted) and once for a species which shares a BIN with private records from Pakistan. Where species might potentially be identified as belonging to *Parapanteles*, species were also diagnosed against species from Australia of that genus, and explanations are given as to why we have chosen to place them in *Apanteles*.

Many species are only described from a single or relatively few specimens, some of which are damaged. Whilst we acknowledge this is not ideal, there are so few taxonomic workers describing Australian braconids that only by providing a name, sequence data and available context for species, does the biodiversity start to become less opaque. Additional specimens are likely able to be easily assigned to species in the future using molecular data. All new species are authored by Slater-Baker, Fagan-Jeffries, Fernández-Triana, Portmann & Oestmann.

### ﻿﻿Distribution data

Distribution data is provided in Figs 4–6 for species of *Apanteles* confirmed for Australia during this study. These data include both material examined during this study and sequences on BOLD that fell within the delimited molecular lineages. Locations represented only by sequence data should be treated with some caution; see Suppl. material 3 for full details of the distribution data used to construct the maps. Distribution maps were created using uMap (https://umap-project.org/), with map tiles by CartoDB, under CC BY 3.0. and map data © OpenStreetMap contributors under ODbL.

### ﻿﻿Terminology

Terms for general morphology follow the Hymenoptera Anatomy Ontology (Yoder et al. 2010) and terminology for sculpture follows Eady (Eady 1968). Diagrams depicting specific measurement terms and practices are given in Fig. 1. When discussing parts of the body, we use ‘anterior’ to mean ‘closer to the head’ and ‘posterior’ to mean closer to the ovipositor (Fig. 1). When discussing characters on the wings and legs, we use ‘proximal’ to mean closer to the mesosoma, and ‘distal’ to mean further from the mesosoma (Fig. 1). We refer to the ‘outer side’ of the metafemur and metatibia in the key and in descriptions, which we define as the surface that is facing away from the body when the leg is at rest in lateral view, i.e., the surface visible in Fig. 1.

The following abbreviations are used throughout the key and descriptions:

**F2/F14** 2^nd^ flagellomere/14^th^ flagellomere

**L/W** length measurement divided by width measurement (used with reference to a particular body part)

**T1** first mediotergite

**T2** second mediotergite

**T3–T6** third to sixth mediotergites

#### ﻿﻿Acronyms

Australian states and territories:

**ACT** Australian Capital Territory

**NSW** New South Wales

**NT** Northern Territory

**QLD** Queensland

**SA** South Australia

**TAS** Tasmania

**VIC** Victoria

**WA** Western Australia

Collection codes:

**AM** Australian Museum, Sydney, Australia

**ANIC** Australian National Insect Collection, Canberra, Australia

**CBG** Centre for Biodiversity Genomics, Guelph, Canada

**CNC** Canadian National Collection of Insects, Ottawa, Canada

**MV** Museums Victora, Melbourne, Australia

**NHM** Natural History Museum, London, England

**QM** Queensland Museum, Brisbane, Australia

**SAMA** South Australian Museum, Adelaide, Australia

**TMAG** Tasmanian Museum and Art Gallery, Hobart, Australia

**WAM** Western Australian Museum, Perth, Australia

**WINC** Waite Insect and Nematode Collection, Adelaide, Australia

## ﻿﻿Results

### ﻿﻿Molecular species delimitation of Australian *Apanteles*

After the molecular species delimitation methods were compared and the consensus was established, 52 provisional species are present in Dataset 1, 48 of these containing material from Australia (Fig. 2, Suppl. material 1).

In more than 80% of cases, a majority of the molecular delimitation methods (i.e., at least five of the seven methods) were congruent (Suppl. material 1), and these molecular hypotheses were considered well supported species. In cases where there was incongruence between several methods, species hypotheses were examined more closely and a decision on the final delimitation was made taking into account all available evidence, including the number of available specimens, average COI distance among potential species, morphological characters, distribution data, and any available host data. An explanation of the reasoning behind the final decision in these cases is given in the remarks section of the relevant species. While this process introduces some subjectivity, we believe it strikes an appropriate balance between increasing the speed of documenting Australia’s biodiversity and creating a relatively robust taxonomic framework for future research.

Of the 45 molecular species hypotheses that did not match an existing named species, 34 delimited groups are described as new species. In six cases, a molecular hypothesis was not described as a new species because there was not a female specimen available for morphological examination during the project. In four cases, a molecular hypothesis was not described because the BIN containing the specimens also contained sequences from outside of Australia and therefore would need to be diagnosed against a very large number of described species, many of which have economic importance or have been introduced to various countries for biological control; to properly delimit or identify these lineages, dedicated projects will be needed. Images of these four lineages are provided as supplementary material (Suppl. material 2) to aid future work. In one final case, a molecular hypothesis was not described because it is very closely related to *A.carpatus* (Say, 1836), which is a complex cosmopolitan species where the limits are not well defined (Fernández-Triana et al. 2020). This species will need a dedicated project encompassing *A.carpatus* specimens from around the world to establish firm species boundaries.

### ﻿﻿Australian *Apanteles* in a global context

Of the 48 delimited molecular lineages that contained specimens from Australia, only eight shared BINs with sequences from other countries, indicating a high degree of endemism within the Australian fauna. Six of the delimited molecular lineages that contain sequences from other countries are so far only known from northern Australia, whilst *A.oenone* (found in Australia and Papua New Guinea) is found throughout the continent and *A.carpatus* (introduced around the world) was collected in SA.

**Figure 3.**
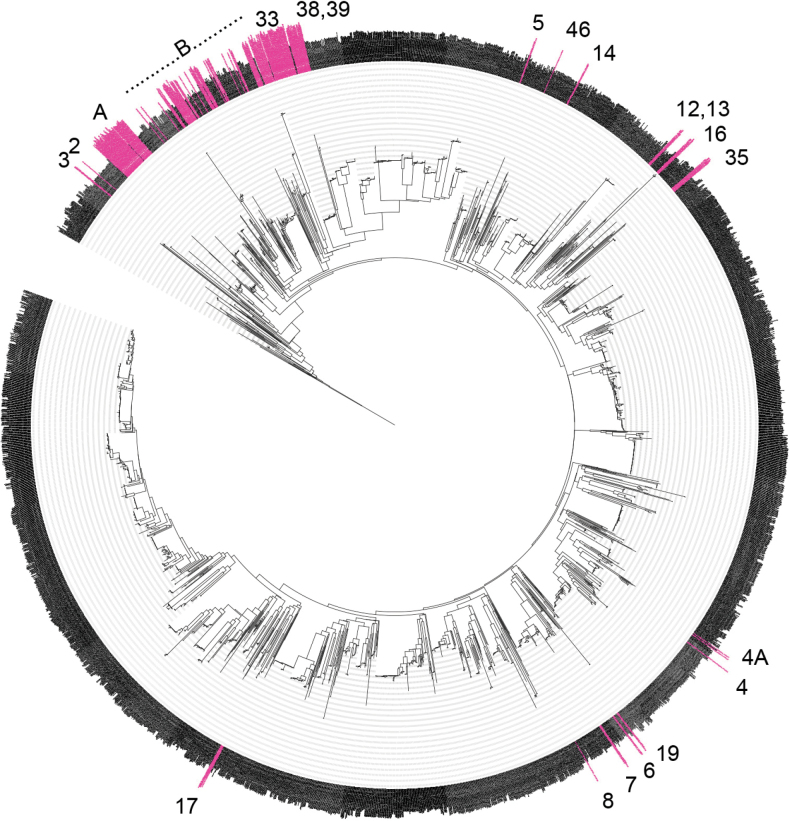
IQ-TREE extended majority rule consensus tree of the COI DNA barcoding region for global *Apanteles*. Australian specimens are shown in pink. Numbers next to species correlate to the ‘informal lineage’ code given in Table 1. The full tree in newick format is available as Suppl. material 5. Group A includes informal lineages 36, 37, 40, 41, 42, 43, 45; whilst group B includes informal lineages 1, 9, 10, 11, 15, 18, 20–32, 34, 46.

Based on COI, the majority of Australian *Apanteles* species currently thought to be endemic are clustered in one part of the tree (Fig. 3). Thirty of the Australian delimited molecular lineages (28 which are currently thought to be endemic, plus *A.oenone* which is known from Papua New Guinea, and *A.allapsus* which is potentially found in Pakistan) cluster between point ‘A’ and point ‘38&39’ on the tree, with specimens from non-Holarctic regions including Australasia, Afrotropical, Neotropical, and Oriental localities. Seven other currently endemic Australian species are found within a large cosmopolitan clade (the species labelled 5, 46, 12–14, 16, and 35 on Fig. 3).

Point ‘4’ on the tree indicates *A.carpatus*, a species that attacks tineid moths (family Tineidae) and that has been introduced around the world. Points 6, 7, 8, and 19 are all potential species (delimited with molecular data but not described in this study) found in Australia where we also have molecular evidence that they are found in Oriental (all of the species) and Afrotropical (one of the species) regions.

### ﻿﻿Taxonomy

**Figure 4.**
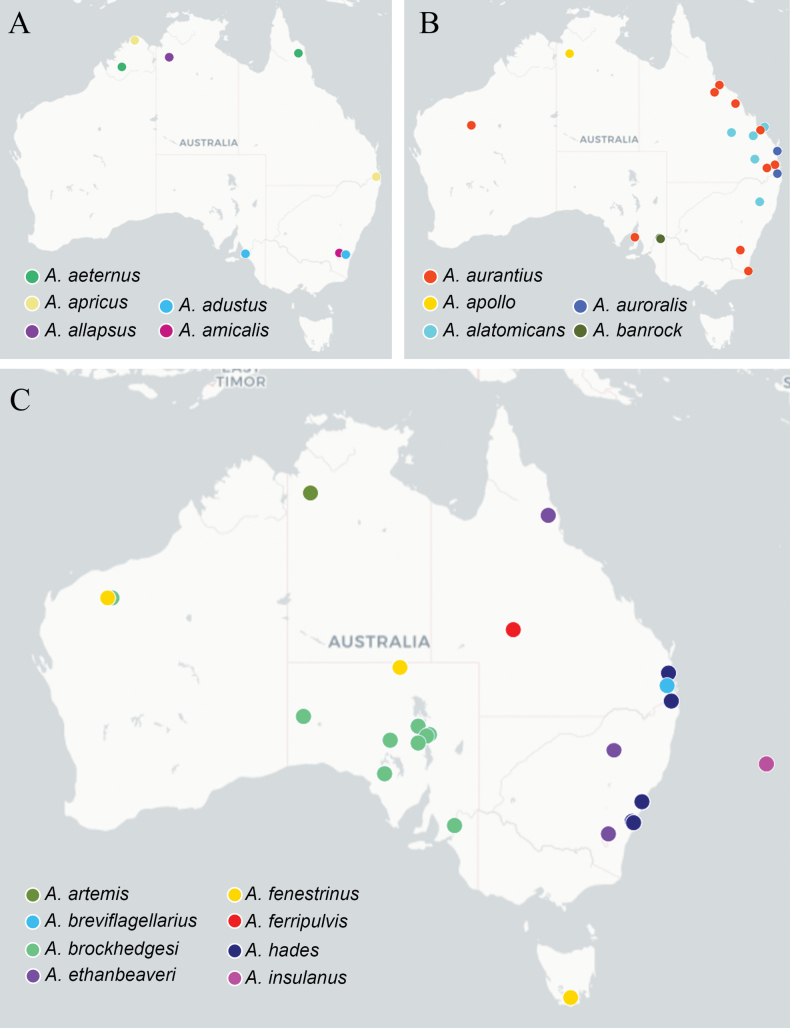
Known distribution of the *Apanteles* species in Australia **A***A.adustus*, *A.aeternus*, *A.allapsus*, *A.amicalis*, *A.apricus***B***A.alatomicans*, *A.apollo*, *A.aurantius*, *A.auroralis*, *A.banrock***C***A.artemis*, *A.breviflagellarius*, *A.brockhedgesi*, *A.ethanbeaveri*, *A.fenestrinus*, *A.ferripulvis*, *A.hades*, and *A.insulanus*. Maps include examined material and public DNA barcode records, see Suppl. material 3 for full details.

**Figure 5.**
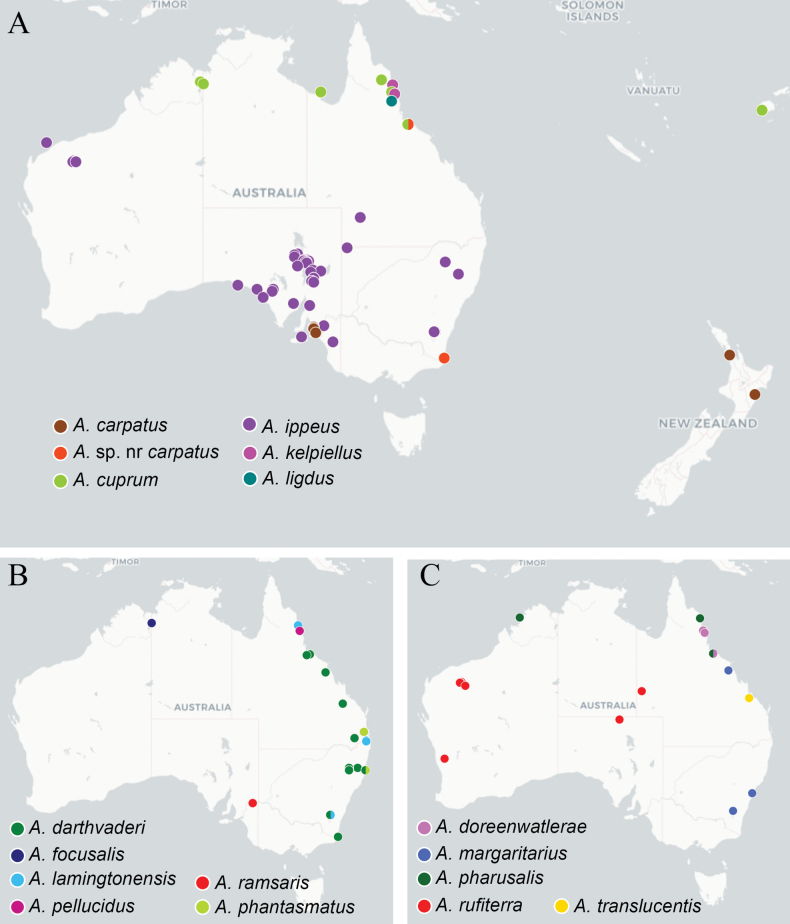
Known distribution in the of the following *Apanteles* species in Australia, Fiji, and New Zealand: **A***A.carpatus*, A.sp.nrcarpatus, *A.cuprum*, *A.ippeus*, *A.kelpiellus*, *A.ligdus***B***A.darthvaderi*, *A.focusalis*, *A.lamingtonensis*, *A.pellucidus*, *A.phantasmatus***C***A.doreenwatlerae*, *A.margaritarius*, *A.pharusalis*, *A.rufiterra*, *A.translucentis*. Maps include examined material and public DNA barcode records, see Suppl. material 3 for full details.

**Figure 6.**
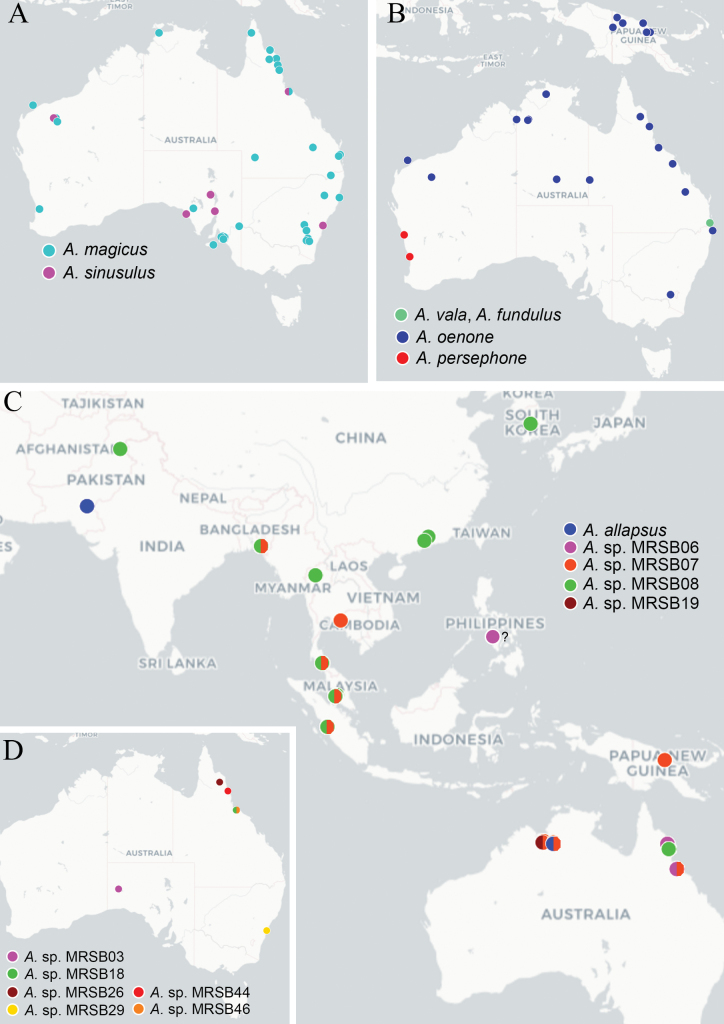
Known distribution of the following *Apanteles* species in Australia and neighbouring regions: **A***A.magicus*, *A.sinusulus***B***A.vala*, *A.fundulus*, *A.persephone* (all based on approximate locations in Nixon’s description), and *A.oenone***C***A.allapsus* and the undescribed lineages also potentially found overseas **D** undescribed lineages where we did not have access to a female specimen. The ‘?’ indicates a private sequence on BOLD where we were unable to obtain the GPS data. Maps include examined material and public DNA barcode records, see Suppl. material 3 for full details.

We here describe 34 new species of *Apanteles* found in Australia, increasing the number of named species reported from Australia to 43 (Table 1; Figs 4–6). Only two of the newly described species are potentially found in another country based on public sequence data (*A.cuprum* in Fiji, and *A.allapsus* in Pakistan).

**Table 1.** Species of *Apanteles* known from Australia, with their known distribution and host data, and the informal lineage code used in this study. Lineages delimited using molecular data but that are not described in this study are in cells with grey shading at the end of the table. Distribution data not sourced from within this study are taken from Austin and Dangerfield (1992) and Fernández-Triana et al. (2020). Host data (other than for new species) is from Austin and Dangerfield (1992) and, for *A.hemara*, from Fernández-Triana et al. (2017). ‘?’ Indicates host records we were unable to confirm. The informal lineage codes are provided for ease of data tracking in the future; ‘-’ indicates the species did not have molecular data available, and ‘*’ indicates the species was not recovered amongst Australian material in this study, so was not designated a lineage code.

Species	Distribution	Host	Informal lineage code
*Apantelesadustus* sp. nov.	NSW, SA	Unknown	MRSB14
*Apantelesaeternus* sp. nov.	QLD, WA	Unknown	MRSB30
*Apantelesalatomicans* sp. nov.	NSW, QLD, SA	Unknown	MRSB41
*Apantelesallapsus* sp. nov.	NT, Pakistan	Unknown	MRSB20
*Apantelesamicalis* sp. nov.	ACT	Unknown	MRSB24
*Apantelesapollo* sp. nov.	NT	Unknown	MRSB01
*Apantelesapricus* sp. nov.	QLD, WA	Unknown	MRSB05
*Apantelesartemis* sp. nov.	NT	Unknown	MRSB02
*Apantelesaurantius* sp. nov.	ACT, NSW, QLD, SA, WA	Unknown	MRSB10
*Apantelesauroralis* sp. nov.	QLD	Unknown	MRSB38
*Apantelesbanrock* sp. nov.	SA	Unknown	MRSB28
*Apantelesbreviflagellarius* sp. nov.	QLD	Unknown	MRSB11
*Apantelesbrockhedgesi* sp. nov.	NSW, SA, WA	Unknown	MRSB34
*Apantelescarpatus* (Say, 1836)	SA, QLD; Fiji, Hawaiian Islands, New Zealand; all world regions	Tineidae (multiple species)	MRSB04
*Apantelescuprum* sp. nov.	NT, QLD, WA; Fiji	Unknown	MRSB09
*Apantelesdarthvaderi* sp. nov.	ACT, NSW, QLD	Unknown	MRSB39
*Apantelesdoreenwatlerae* sp. nov.	QLD	Unknown	MRSB16
*Apantelesethanbeaveri* sp. nov.	ACT, NSW, QLD	Lycaenidae: *Jalmenuspseudictinus* Kerr & Macqueen, 1967, *J.ictinus* Hewitson, [1865], *Ogyrislanthis* Waterhouse, 1900	MRSB27B
*Apantelesfenestrinus* sp. nov.	SA, TAS, WA	Unknown	MRSB21
*Apantelesferripulvis* sp. nov.	QLD	Unknown	MRSB42
*Apantelesfocusalis* sp. nov.	NT	Unknown	MRSB25
*Apantelesfundulus* Nixon, 1965	QLD, Vietnam	Unknown	-
*Apantelesgalleriae* Wilkinson, 1932	QLD; Hawaiian Islands, New Zealand; found in all world regions	Pyralidae: *Galleriamellonella* (Linnaeus, 1758)	*
*Apanteleshades* sp. nov.	NSW, QLD	Unknown	MRSB40
*Apanteleshemara* Nixon, 1965	ACT; Afrotropical, Oriental and Palearctic	Choreutidae and Crambidae (several species)	*
*Apantelesinsulanus* sp. nov.	Lord Howe Island	Unknown	MRSB36
*Apantelesippeus* Nixon, 1965	ACT, NSW, QLD, SA, WA; Vietnam	Plutellidae: *Plutellaxylostella* (Linnaeus, 1758)	MRSB33
*Apanteleskelpiellus* sp. nov.	QLD	Unknown	MRSB13
*Apanteleslamingtonensis* sp. nov.	ACT, QLD	Unknown	MRSB43
*Apantelesligdus* sp. nov.	QLD	Lycaenidae: *Ogyrisiphis* Waterhouse & Lyell, 1914	MRSB27A
*Apantelesmagicus* sp. nov.	ACT, NSW, QLD, SA, WA	Unknown	MRSB45
*Apantelesmargaritarius* sp. nov.	ACT, NSW, QLD	Unknown	MRSB12
*Apantelesoenone* Nixon, 1965	NSW, NT, QLD, WA; Papua New Guinea	Gelechiidae: *Pectinophoragossypiella* (Saunders, 1844), ?*P.scutigera* (Holloway, 1926), Nolidae: ?*Eariasvittella* Fabricius, 1794	MRSB32
*Apantelespellucidus* sp. nov.	QLD	Unknown	MRSB23
*Apantelespersephone* Nixon, 1965	WA	Unknown	-
*Apantelesphantasmatus* sp. nov.	NSW, QLD	Unknown	MRSB35
*Apantelespharusalis* sp. nov.	QLD, WA	Unknown	MRSB15
*Apantelesramsaris* sp. nov.	SA	Unknown	MRSB37
*Apantelesrufiterra* sp. nov.	QLD, SA, WA	Unknown	MRSB17
*Apantelessinusulus* sp. nov.	NSW, QLD, SA, WA	Unknown	MRSB22
*Apantelessubandinus* Blanchard, 1947	ACT, NSW, QLD, SA, TAS, VIC, WA; New Zealand; Afrotropical and Neotropical	Gelechiidae: *Phthorimaeaoperculella* (Zeller, 1873)	*
*Apantelestranslucentis* sp. nov.	QLD	Unknown	MRSB31
*Apantelesvala* Nixon, 1965	QLD	Unknown	-
*Apanteles* sp. MRSB03	SA	Unknown	MRSB03
*Apanteles* sp. MRSB06	QLD; Philippines	Unknown	MRSB06
*Apanteles* sp. MRSB07	NT; several countries in South East Asia	Unknown	MRSB07
*Apanteles* sp. MRSB08	QLD; several countries in South East Asia and Oriental regions	Unknown	MRSB08
*Apanteles* sp. MRSB18	QLD	Unknown	MRSB18
*Apanteles* sp. MRSB19	NT, QLD; Indonesia	Unknown	MRSB19
*Apanteles* sp. MRSB26	QLD	Unknown	MRSB26
*Apanteles* sp. MRSB29	NSW	Unknown	MRSB29
*Apanteles* sp. MRSB44	QLD	Unknown	MRSB44
*Apanteles* sp. MRSB46	QLD	Unknown	MRSB46
Apantelessp.nrcarpatus	NSW, QLD	Unknown	MRSB04.B

#### ﻿Key to species

Some important points for users of the key to Australian *Apanteles*:

- Even just within this study, there are multiple lineages of *Apanteles* that have not been described, and there will be numerous additional unknown species present in under-sampled habitats in Australia. As such, users should be aware that even if a specimen is able to be keyed to a species, there is still a high possibility that it could be an undescribed species. Wherever feasible, morphological species identifications should be supported by DNA barcoding and the placement of the sequence in the context of a phylogeny.
- In several cases in the key, we do not provide a morphological character to separate a pair or triplet of closely related species, and recommend identification using molecular data. This only occurs when species were determined to be very distinct molecularly, and therefore very likely to be separate species despite the lack of clear, unambiguous diagnostic characters.
- Occasionally where there is a clear visual difference in a character, we refer the user to figures rather than attempting to describe the difference in words, to avoid convoluted and confusing couplets.

#### ﻿﻿Key to species of Australian *Apanteles*, based on female specimens

**Table:** 

1	T1 strongly narrowing posteriorly; AND pterostigma with pale centre AND propodeum smooth with areola poorly defined by rather short carinae on posterior 1/2 (as in Fig. 58B)	***A.subandinus* Blanchard, 1947**
–	T1 parallel, barrel shaped or only narrowing slightly posteriorly (e.g., Fig. 12A); OR pterostigma uniformly pigmented OR propodeum either strongly rugose, IF smooth THEN with areola usually better defined by longer carinae	**2**
2(1)	Metacoxa pale, significantly paler than mesosoma (e.g., Fig. 7A, B)	**3**
–	Metacoxa dark, same, or only slightly paler than mesosoma (e.g., Fig. 7C, D)	**4**
3(2)	T1 anterior width/posterior width ratio ~ 1.7	***A.doreenwatlerae* sp. nov.**
–	T1 anterior width/posterior width ratio ~ 1.2	***A.pharusalis* sp. nov.** ^1^
4(2)	Pterostigma with large pale spot in proximal corner (Fig. 7E) AND fore wing veins 1CUa, 1CUb, 1m-cu all pale/unpigmented (Fig. 7E) AND T1 and T2 strongly sculptured	***A.carpatus* (Say, 1836)**
–	Pterostigma either uniformly pigmented, or with paler centre (e.g., Fig. 7G), or with only a very small pale proximal spot (e.g., Fig. 7H); IF with large pale spot in proximal corner THEN at least some of fore wing veins 1CUa, 1CUb, 1m-cu have pigmentation (e.g., Fig. 7F) OR T1 or T2 smooth	**5**
5(4)	Metafemur with at least some areas significantly paler in colour (yellowish) compared to colour of mesosoma (when pale area only in proximal 1/4, trochanter and trochantellus also pale) (e.g., Fig. 8A–D)	**6**
–	Metafemur same colouration or only slightly paler (but still clearly brown, not yellowish) than mesosoma (e.g., Fig. 8E, F; note paler brown metafemur in Fig. 8F)	**14**
6(5)	Metafemur with at least some dark colouration (e.g., Fig. 8C, D)	**7**
–	Metafemur uniformly pale (e.g., Fig. 8A, B)	**11**
7(6)	T2 entirely sculptured with strong longitudinal striae; AND T1 slightly widening on posterior 1/2 and entirely and coarsely sculptured; AND propodeum mostly smooth but with complete and strong lateral carinae (Fig. 40B)	***A.hemara* Nixon, 1965**
–	T2 usually smooth, IF sculptured THEN only partially and with less strongly marked sculpture; OR T1 narrowing posteriorly and usually not as strongly sculptured on its entire surface; OR propodeum variably rugose, sometimes with lateral carina difficult to discern amongst sculpturing	**8**
8(7)	Metafemur mostly pale or orange with dark section only in distal 1/3 (Fig. 8C); T2 with at least some sculpturing	***A.aurantius* sp. nov.**
–	Metafemur mostly dark, with pale area only in proximal 1/4 (e.g., Fig. 8D); T2 smooth	**9**
9(8)	Anterior side of scape comparatively much paler than head colour (Fig. 9A)	***A.kelpiellus* sp. nov.**
–	Anterior side of scape same or only very slightly paler than head colour (e.g., Fig. 9B)	**10** ^2^
10(9)	T1 comparatively more strongly narrowing posteriorly (Fig. 9D)	***A.cuprum* sp. nov.**
–	T1 parallel sided, or at most slightly narrowing posteriorly (Fig. 9C)	***A.margaritarius* sp. nov.**
11(6)	Ovipositor sheath length < 0.5 × metatibia length; hypopygium without defined ventral pleats	**12**
–	Ovipositor sheath 0.8–1.0 × metatibia length; hypopygium with clearly defined ventral pleats	**13**
12(11)	T1 more or less parallel-sided; T2 comparatively more transverse (as in Fig. 20B)	***A.apollo* sp. nov.**
–	T1 narrowing towards posterior margin; T2 comparatively less transverse (as in Fig. 22B)	***A.artemis* sp. nov.**
13(11)	Propodeum with strong rugose sculpture, areola not well defined amongst strong sculpture; T1 slightly narrowing posteriorly (as in Fig. 21D)	***A.apricus* sp. nov.**
–	Propodeum comparatively smoother, areola clearly defined; T1 parallel-sided (as in Fig. 24B)	***A.auroralis* sp. nov.**
14(5)	Pterostigma with outer border darker than centre, centre of pterostigma pale or transparent/hyaline (e.g., Figs 7G, 11A, C, E, G; see Fig. 11E for a less extreme example)	**15**
–	Pterostigma centre uniformly pigmented (pterostigma may have pale spot proximally, but never hyaline in centre) (e.g., Fig. 7H)	**20**
15(14)	Fore wing with veins M+CU, 1 cu-a, 1M, 1CUa, 1CUb, (RS+M)a, 2RS, and 1m-cu all unpigmented, pale, or transparent (e.g., Fig. 10A)	**16**
–	Fore wing with some of those veins with pigmentation, either M+CU, 1 cu-a, 1M, 1CU, (RS+M)a, 2RS, or 1m-cu (e.g., Fig. 10B)	**18**
16(15)	T1 smooth	***A.fenestrinus* sp. nov.**
–	T1 with rugose sculpturing or at a minimum with strong punctures on lateral sides	**17**
17(16)	Metatibia mostly dark, or with dark colouration occupying at least 1/2 of tibia length (Fig. 10C, D)	***A.oenone* Nixon, 1965**
–	Metatibia mostly pale (Fig. 10E, F)	***A.aeternus* sp. nov.** or ***A.translucentis* sp. nov.**^3^
18(15)	Fore wing veins M+CU and 1M pigmented less than 1/2 their lengths (e.g., Fig. 11A, C); comparatively larger ocelli, OOD/POD < 2.5 (Fig. 11B, D)	***A.rufiterra* sp. nov.** and ***A.pellucidus* sp. nov.**^4^
–	Fore wing veins M+CU and 1M pigmented for most of their lengths (Fig. 11E, G); comparatively smaller ocelli, OOD/POD > 2.5 (Fig. 11F, H)	**19**
19(18)	T1 with strong sculpture; propodeal areola strongly V-shaped (as in Fig. 25D); propodeum with strong sculpture	***A.banrock* sp. nov.**
–	T1 smooth; propodeal areola slightly rounded (as in Fig. 18B); propodeum relatively smooth	***A.allapsus* sp. nov.**
20(14)	Ovipositor sheath length < 0.5 × metatibia length	**21**
–	Ovipositor sheath length > 0.6 × metatibia length	**23**
21(20)	T1 strongly narrowing posteriorly; propodeal areola comparatively narrow, median length of propodeum 2.0 × maximum width of areola (Fig. 12A)	***A.breviflagellarius* sp. nov.**
–	T1 parallel sided; propodeal areola comparatively broad, median length of propodeum 1.2 × maximum width of areola (Fig. 12B, C)	**22**
22(21)	Scutoscutellar sulcus narrower and with much smaller pits (Fig. 12B); T2 comparatively more transverse (Fig. 12B); T2 with posterior margin more or less straight or very slightly curved	***A.ligdus* sp. nov.**
–	Scutoscutellar sulcus wider and with much larger pits (Fig. 12C); T2 comparatively less transverse (Fig. 11C); T2 with posterior margin clearly curved	***A.ethanbeaveri* sp. nov.**
23(20)	Antennae significantly shorter (~ 0.6–0.7 ×) than body length AND T3 with multiple rows of setae (Fig. 38B)	***A.galleriae* Wilkinson, 1932**
–	Antennae usually similar size (at least 0.8 ×) or usually longer than body length; IF significantly shorter than body THEN with setae on T3 reduced to a single row along posterior margin	**24**
24(23)	Pterostigma with large conspicuous pale spot (as in Fig. 7F) AND propodeal areola comparatively narrow (as in Fig. 42B)	***A.ippeus* Nixon, 1965**
–	Pterostigma uniformly coloured (no pale spot), IF pale spot present, THEN small and restricted to very edge of pterostigma OR propodeal areola comparatively wide and V-shaped (as in Fig. 27B)	**25**
25(24)	Metatibia mostly dark with pale band discrete (tibia goes abruptly from dark to pale) and pale band restricted to proximal 1/2 of tibia (Fig. 13A–F)	**26**
–	Metatibia mostly pale with dark colouration only on distal 1/3 **OR** metatibia displaying a gradient of colouration from pale to dark, with a significant area that is an intermediate/blending of the two colours (Fig. 13G–L)	**31^5^**
26(25)	Fore wing membrane completely hyaline/transparent, without trace of any infuscation in the membrane (Fig. 13Q)	**27**
–	Fore wing membrane with at least a small amount of infuscation, particularly noticeable around r vein (Fig. 13N, O)	**29** ^6^
27(26)	T3–T6 with setae reduced to a single row on each tergite (occasionally tergites may have a few additional scattered setae but most are in single row); fore wing vein r particularly short, always shorter, or just equal in length compared to vein 2RS]	***A.darthvaderi* sp. nov.** ^7^
–	T3–T6 with setae not reduced, irregularly arranged or in multiple rows; fore wing with vein r equal in length or slightly longer than vein 2RS	**28**
28(27)	Pale band on metatibia comparatively shorter and more discrete, T2 posterior width/medial length ratio comparatively smaller (~ 4.1), T2 usually smooth	***A.phantasmatus* sp. nov.** ^8^
–	Pale band on metatibia comparatively longer and less discrete (Fig. 13M), T2 posterior width/medial length ratio comparatively larger (~ 2.6), T2 often with some shallow sculpturing	***A.adustus* sp. nov.**
29(26)	Infuscation on fore wing covering most of membrane; basal cell of fore wing comparatively more densely setose; pterostigma length/width ratio 2.6–3.0 (e.g., Fig. 13N)	**30**
–	Infuscation on fore wing restricted to area around veins r and 1CUb; basal cell of fore wing comparatively less densely setose; pterostigma length/width ratio 2.2–2.5 (e.g., Fig. 13O, P)	***A.hades* sp. nov., *A.alatomicans* sp. nov.**, and ***A.magicus* sp. nov.**^9^
30(29)	T3–T6 with setae reduced to a single row on each tergite	***A.lamingtonensis* sp. nov.**
–	T3–T6 with setae not reduced to a single row; setae irregularly arranged	***A.ferripulvis* sp. nov.**
31(25)	T1 mostly smooth, or at most with small rugose ‘trough’ in centre	**32**
–	T1 with strong sculpture over at least most of posterior 1/2 of tergite	**33**
32(31)	Pterostigma with small pale spot at proximal corner; setae on T3–T6 reduced to single row at posterior edge of tergites	***A.fundulus* Nixon, 1965**
–	Pterostigma without pale spot at proximal corner; T3–T6 with multiple rows of setae	***A.focusalis* sp. nov.** and ***A.sinusulus* sp. nov.**
33(31)	Mesoscutellar disc punctate throughout	***A.adustus* sp. nov.**
–	Mesoscutellar disc mostly smooth, or at most with scattered punctures along margins	**34**
34(33)	Fore wing vein 1M much less pigmented (often transparent/pale) compared to pigmentation of vein 1CUa (e.g., Fig. 14A)	**35**
–	Fore wing veins 1M and 1CUa of similar pigmentation (e.g., Fig. 14B)	**36**
35(34)	Scutoscutellar sulcus comparatively narrower and with comparatively smaller pits (Fig. 14C)	***A.brockhedgesi* sp. nov.**
–	Scutoscutellar sulcus comparatively wider and with comparatively larger pits (Fig. 14D)	***A.insulanus* sp. nov.** and ***A.ramsaris* sp. nov.**^10^
36(34)	T2 posterior length/width ratio ~ 5.0	***A.amicalis* sp. nov.** ^11^
–	T2 posterior length/width ratio ~ 3.5	***A.vala* Nixon, 1965 N**

**Figure 7.**
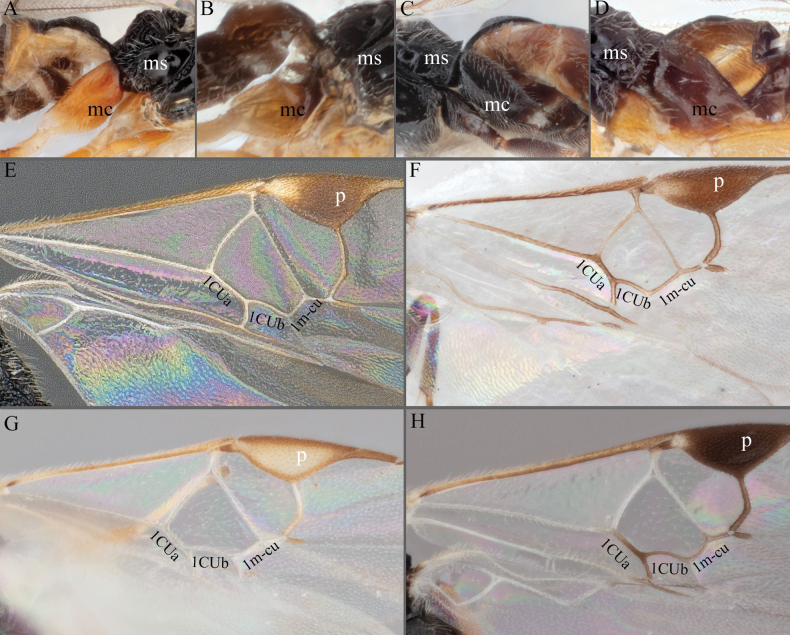
**A***A.doreenwatlerae***B***A.pharusalis***C***A.* MRSB41 **D***A.auroralis***E***A.carpatus***F***A.ippeus* (image flipped along vertical axis for ease of comparison) **G***A.oenone***H***A.brockhedgesi*. Abbreviations: mc: metacoxa; ms: mesosoma; p: pterostigma.

**Figure 8.**
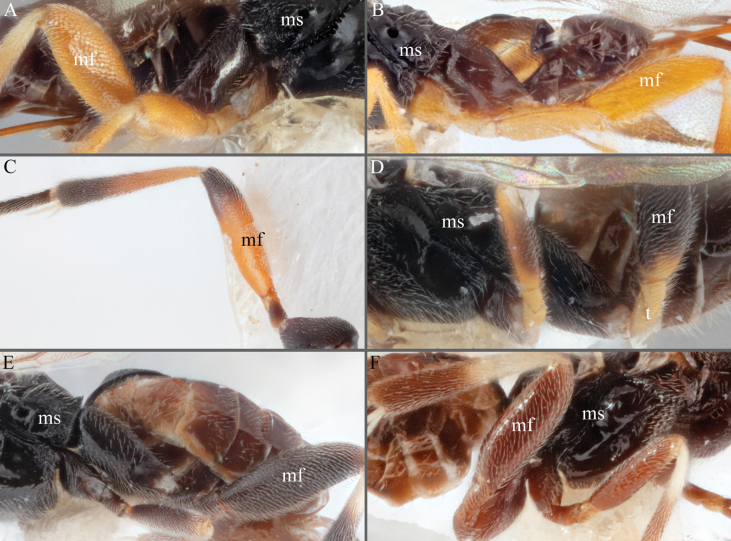
**A–D** metafemur with some pale areas **A***A.apricus***B***A.auroralis***C***A.aurantius***D***A.margaritarius***E, F** metafemur completely dark **E***A.alatomicans***F***A.lamingtonensis*. Abbreviations: ms: mesosoma; mf: metafemur; t: trochanter/trochantellus.

**Figure 9.**
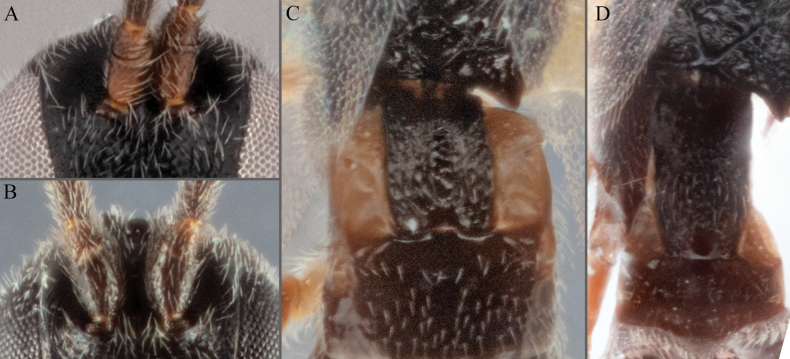
**A***A.kelpiellus***B***A.margaritarius***C***A.margaritarius***D***A.cuprum*.

**Figure 10.**
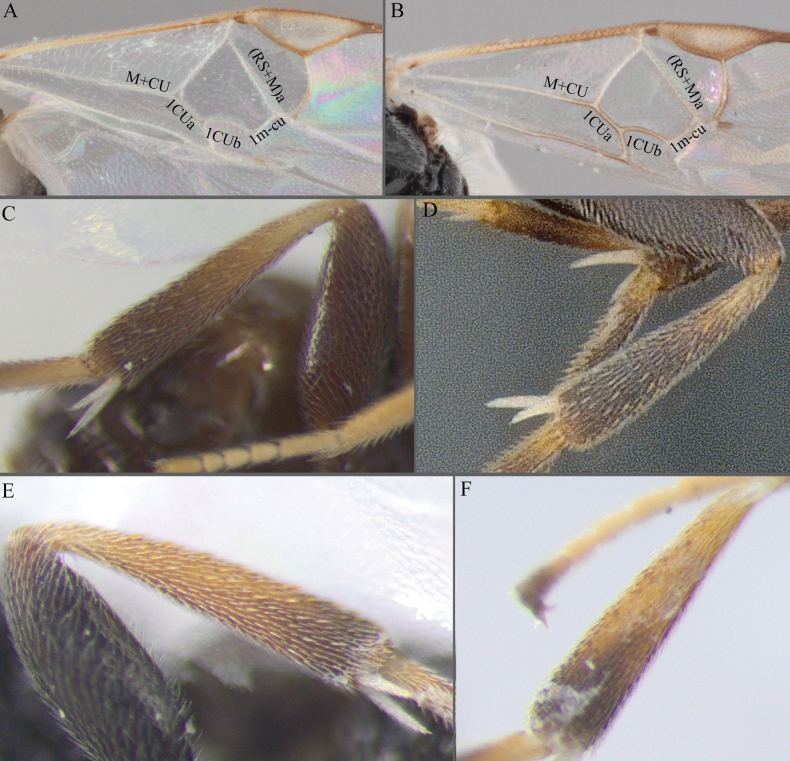
**A***A.aeternus* fore wing veins all pale **B***A.* sp. MRSB19 with veins 1CUa, 1CUb, and part of M+CU pigmented **D, E***A.oenone* hind tibia **E***A.translucentis* hind tibia **F***A.aeternus* hind tibia.

**Figure 11.**
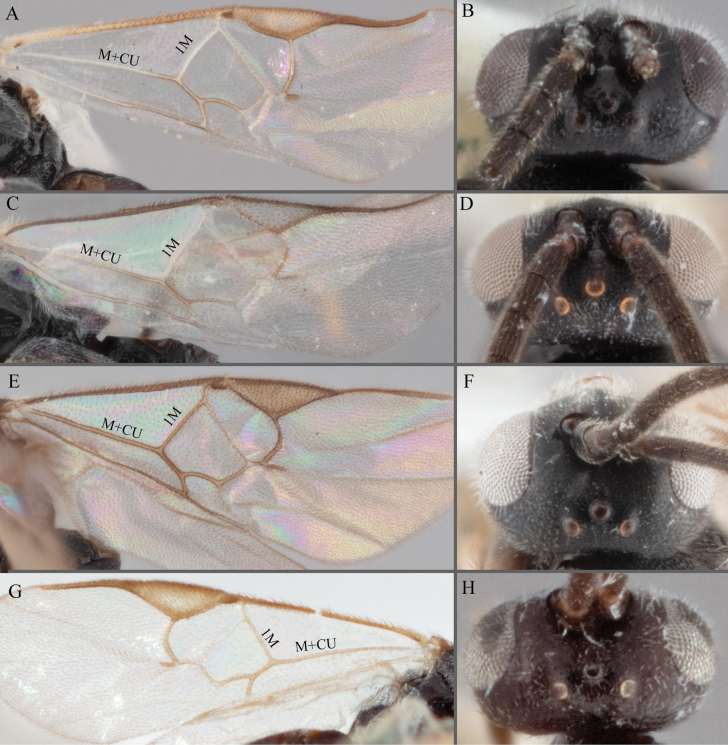
**A, B***A.* sp. MRSB19 **C, D***A.pellucidus***E, F***A.banrock***G, H***A.allapsus*.

**Figure 12.**
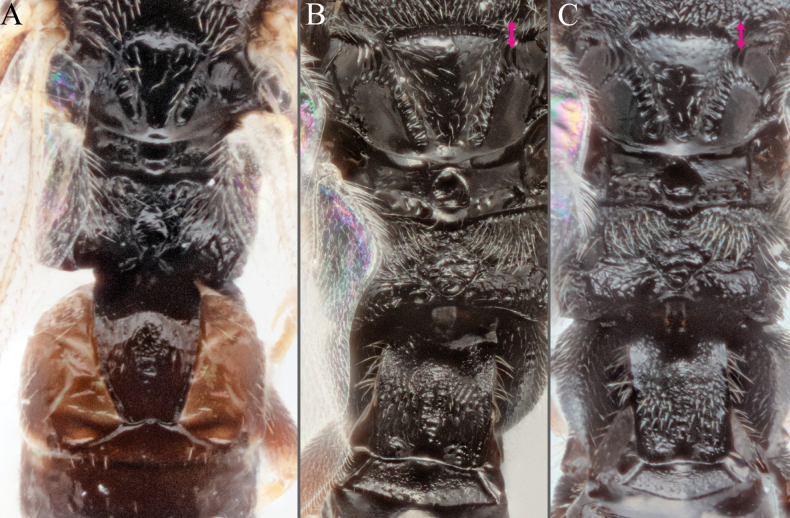
**A***A.breviflagellarius***B***A.ligdus***C***A.ethanbeaveri*. The pink arrow indicates the direction to assess the ‘width’ of the scutoscutellar sulcus.

**Figure 13.**
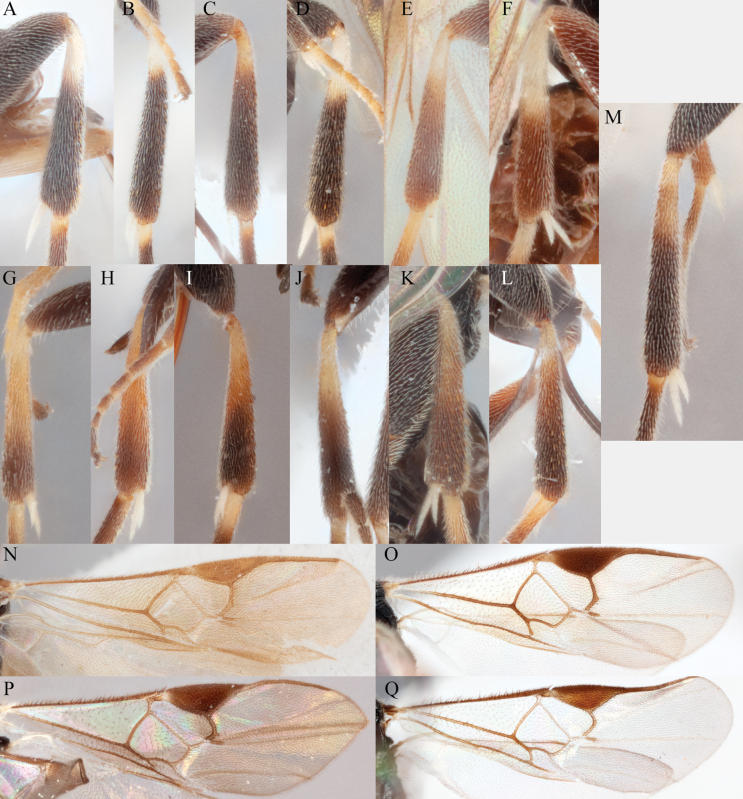
**A–M** hind tibia **N–Q** fore wing; **A***A.alatomicans***B***A.phantasmatus***C***A.darthvaderi***D***A.hades***E***A.ferripulvis***F***A.lamingtonensis***G***A.focasalis***H***A.sinusulus***I***A.brockhedgesi***J***A.amicalis***K***A.insulanus***L***A.ramsaris***M***A.adustus***N***A.lamingtonensis***O***A.magicus***P***A.alatomicans***Q***A.darthvaderi*.

**Figure 14.**
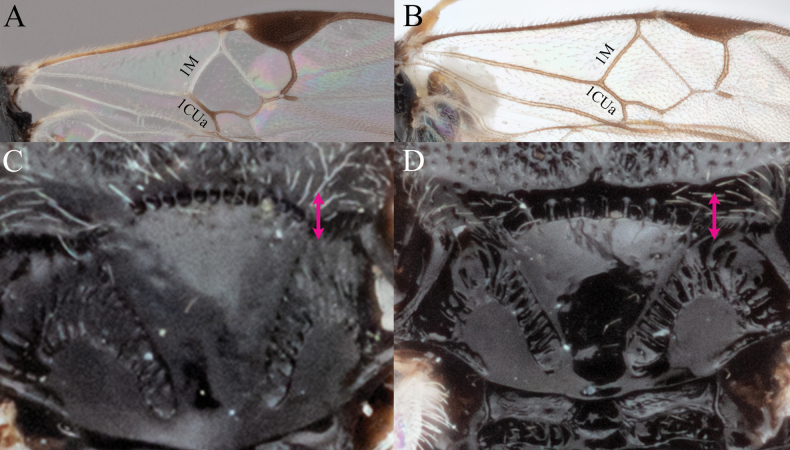
**A***A.brockhedgesi***B***A.amicalis***C***A.brockhedgesi***D***A.insulanus*. The pink arrow indicates the direction of ‘width’ of the scutoscutellar sulcus.

#### ﻿﻿Taxonomic treatment of species

##### ﻿﻿ Apanteles

Taxon classificationAnimaliaHymenopteraBraconidae

Foerster, 1863

9DC9D616-8DF2-5F77-AF6A-73EEB6112AAC

Apanteles Foerster, 1863: 245.

###### Type species.

*Microgasterobscurus* Nees, 1834, by original designation and monotypy.

###### Remarks.

See Fernández-Triana et al. (2020) for full list of synonyms and additional information, including discussions on the limits of the genus in a global context, and for a world checklist of species. We do not alter the diagnosis of *Apanteles* given in Fernández-Triana et al. (2020), which is as follows: *Apanteles* can be recognised by the propodeum usually fully to partially areolated, rarely smooth and never with a median longitudinal carina; fore wing without an areolet; hind wing with the vannal lobe usually strongly concave or straight; ovipositor sheaths relatively long (however, a few species, including some described in this paper, have the sheaths much shorter); and the hypopygium almost always flexible and pleated (with rare exceptions, including two species described here).

Species with longer ovipositor sheaths can often be misidentified as *Dolichogenidea*, especially if the vannal lobe setosity is difficult to assess (i.e., depending on how setae are distributed and its length, at times this character cannot be interpreted unambiguously); however, *Apanteles* is very distinct from this genus using molecular data. Species of *Apanteles* with short ovipositor sheaths and an inflexible hypopygium could be confused with *Parapanteles* or *Pholetesor*, and it is possible that they may actually belong in these genera. However, because the limits of both *Parapanteles* and *Pholetesor* are not well defined, and the species cluster closely with *Apanteles* in the phylogenies, we here choose to place these species in *Apanteles* and highlight them as candidates for future phylogenomic work revising the relationships within Microgastrinae.

##### ﻿﻿ Apanteles adustus

Taxon classificationAnimaliaHymenopteraBraconidae

Slater-Baker, Fagan-Jeffries, Fernández-Triana, Portmann & Oestmann sp. nov.

28DEDFDF-3EF3-5A78-A5E7-D157322E1DDB

https://zoobank.org/DA895592-D135-46C4-92CA-6F0C1FFFF66B

**Figure 15.**
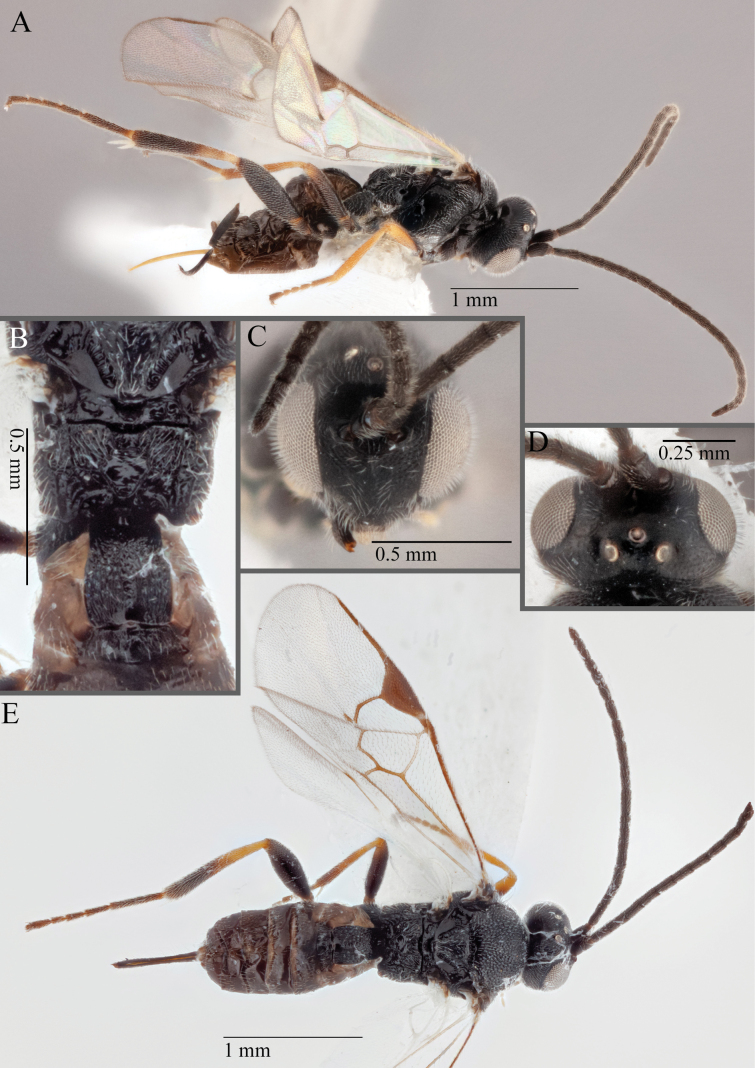
*Apantelesadustus***A** lateral habitus (holotype AUMIC247-18) **B** dorsal propodeum and T1–T3 (paratype AUMIC212-18) **C** anterior head (holotype AUMIC247-18) **D** dorsal head (holotype AUMIC247-18) **E** dorsal habitus and wings (paratype AUMIC212-18).

Fig. 4A (distribution), Fig. 15 (holotype and paratype)

###### Type material.

***Holotype*.** Australia • ♀; NSW, Braidwood, Glenmore Rd.; -35.4232, 149.771; 17–29 Dec. 2005; C. Stephens leg.; Malaise trap; exotic/native garden blend in pasture setting; BOLD Process ID: AUMIC247-18; AM: K.647409. ***Paratypes*.** Australia • ♀; as holotype except: BOLD Process ID: AUMIC204-18; AM: K.647410. • ♀; as previous except: BOLD Process ID: AUMIC212-18; AM: K.647411. • ♀; as previous except: BOLD Process ID: AUMIC248-18; AM: K.647412. • ♀; as previous except: BOLD Process ID: AUMIC249-18; AM: K.647413. • ♀; as previous except: BOLD Process ID: AUMIC250-18; SAMA: 32-47776. • ♀; as previous except: BOLD Process ID: AUMIC254-18; SAMA: 32-47777. • ♀ (head detached); as previous except: BOLD Process ID: AUMIC233-18; AM: K.647414. • ♀ (head detached); as previous except: BOLD Process ID: AUMIC252-18; AM: K.647415. • ♂ (head detached); SA, Cox Scrub Con. Pk.; -35.3311, 138.747; 1–14 May. 2016; E. Fagan-Jeffries leg.; Malaise trap; BOLD Process ID: AUMIC093-18; SAMA: 32-47778.

###### Diagnostic description.

***Size***: Total body length: 2.8 mm; fore wing length: 2.5 mm. ***Head***: anterior scape colour similar or only very slightly paler than head colour; F2L/W ratio: 2.9; F14L/W ratio: 1.4. ***Mesosoma***: scutoscutellar sulcus with seven pits; mesoscutellar disc punctate throughout; propodeal areola complete, or mostly so; propodeum mostly rugose; coxae colour (pro, meso, meta): all dark; metafemur colour mostly dark. ***Wings***: centre of pterostigma pigmented to same degree as the outer edges; fore wing r vein length/2RS vein length ratio: 1.4. ***Metasoma***: T1 shape mostly parallel, T1 medial length/anterior width between 1–2 × longer than wide; T1 mostly rugose; T2 mostly smooth; ovipositor sheath length/metatibia length ratio: 1.0.

*Apantelesadustus* can be separated from the other species of *Apanteles* in Australia that have a dark metacoxa and metafemur, ovipositor sheaths > 0.6 × metatibia length, antennae of similar size or longer than the length of the body, a completely hyaline fore wing membrane, and a uniformly pigmented pterostigma (no paler centre region, and no pale spot on proximal corner), by having T1 with strong rugose sculpture over most of length, the mesoscutellar disc punctate throughout, T3 densely setose, and T2 posterior width/medial length ratio ~ 2.6.

###### Etymology.

The species epithet is a Latin adjective meaning ‘singed/burnt’ and is inspired by the dark colouration, particularly the two-tone metatibia.

###### Distribution.

*Apantelesadustus* is currently known from two sites, one in NSW and one in SA.

###### Molecular information.

*Apantelesadustus* is currently represented by sequences in BIN BOLD:ADL3155. The COI sequences are at least 5% divergent from any of the other species treated here, or any available sequence on BOLD. The *wg* sequence of the holotype is ≥ 19 bp different to any other species. All molecular species delimitation methods separated *Apantelesadustus* as a distinct species.

##### ﻿﻿ Apanteles aeternus

Taxon classificationAnimaliaHymenopteraBraconidae

Slater-Baker, Fagan-Jeffries, Fernández-Triana, Portmann & Oestmann sp. nov.

3E08C000-1FAD-5AFB-9A83-D9A863A95562

https://zoobank.org/35B82166-6F3B-4132-B486-B3564F7CC9E4

**Figure 16.**
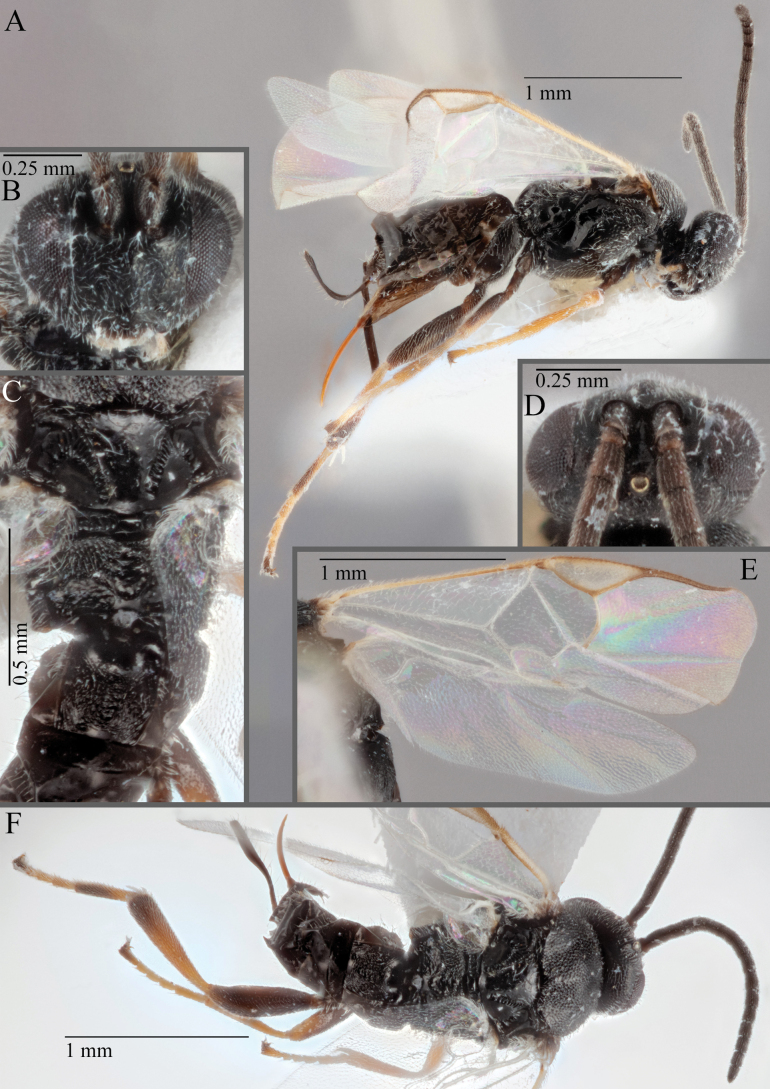
*Apantelesaeternus* (holotype AUMIC1220-24) **A** lateral habitus **B** anterior head **C** dorsal propodeum and T1–T3 **D** dorsal head **E** fore and hindwing **F** dorsal habitus.

Fig. 4A (distribution), Fig. 16 (holotype)

###### Type material.

***Holotype*.** Australia • ♀; QLD, roadside south of Laura; -15.6553, 144.544; 19 Nov. 2019; E. Fagan-Jeffries, J. B. Dorey & P. Ruhr leg.; Sweeping high on *Corymbiagrandifolia*; BOLD Process ID: AUMIC1220-24; QM: T261213. ***Paratype*.** Australia • ♂; WA, West Kimberley, Wunaamin Conservation Park, SSS3; -17.049, 125.236; 21 Jul. 2022; E.P. Fagan-Jeffries leg.; Sweeping; Bush Blitz West Kimberley; BOLD Process ID: AUHYM012-22; WAM: 130565.

###### Diagnostic description.

***Size***: Total body length: 2.4 mm; fore wing length: 2.4 mm. ***Head***: anterior scape colour similar or only very slightly paler than head colour; F2L/W ratio: 2.0; F14L/W ratio: 1.0. ***Mesosoma***: scutoscutellar sulcus with 11 pits; mesoscutellar disc mostly smooth, or with very shallow scattered indentations; propodeal areola complete, or mostly so; propodeum mostly smooth posteriorly, mostly rugose anteriorly; coxae colour (pro, meso, meta): dark all; metafemur colour mostly dark. ***Wings***: centre of pterostigma paler (more hyaline) than outer edges; fore wing r vein length/2RS vein length ratio: 1.8. ***Metasoma***: T1 shape mostly parallel, T1 medial length/anterior width between 1–2 × longer than wide; mostly rugose; T2 mostly smooth; ovipositor sheath length/metatibia length ratio: 1.0.

*Apantelesaeternus* can be separated from the other species of *Apanteles* in Australia which have the metacoxa and metafemur dark and the pterostigma with a paler centre by having fore wing with veins M+CU, 1 cu-a, 1M, 1CUa, 1CUb, (RS+M)a, 2RS, and 1m-cu all unpigmented or transparent, T1 with strong rugose sculpturing and the metatibia mostly pale. *Apantelesaeternus* is easily separated from *A.translucentis* using morphology, but the two species cluster discretely using COI and *wg* barcodes.

###### Etymology.

The species epithet is a Latin adjective meaning ‘everlasting/eternal’.

###### Distribution.

*Apantelesaeternus* is currently known from two remote sites, one in northern WA and one in northern QLD.

###### Molecular information.

Sequences of *Apantelesaeternus* currently form BIN BOLD:AEZ9092. The COI sequences are at least 4% divergent from any of the other species treated here, or any available sequence on BOLD. The *wg* sequence of the holotype is ≥ 6 bp different to any other species. Five species delimitation methods delimited *A.aeternus* as a discrete species, however COIASAP and *wg*PTP analyses grouped the sequences with *A.translucentis* and *A.oenone*.

##### ﻿﻿ Apanteles alatomicans

Taxon classificationAnimaliaHymenopteraBraconidae

Slater-Baker, Fagan-Jeffries, Fernández-Triana, Portmann & Oestmann sp. nov.

25D29275-3B97-5F75-92C6-B07BE1D0069B

https://zoobank.org/6EEC3062-6E0B-4E8E-BF95-16303C4BE292

**Figure 17.**
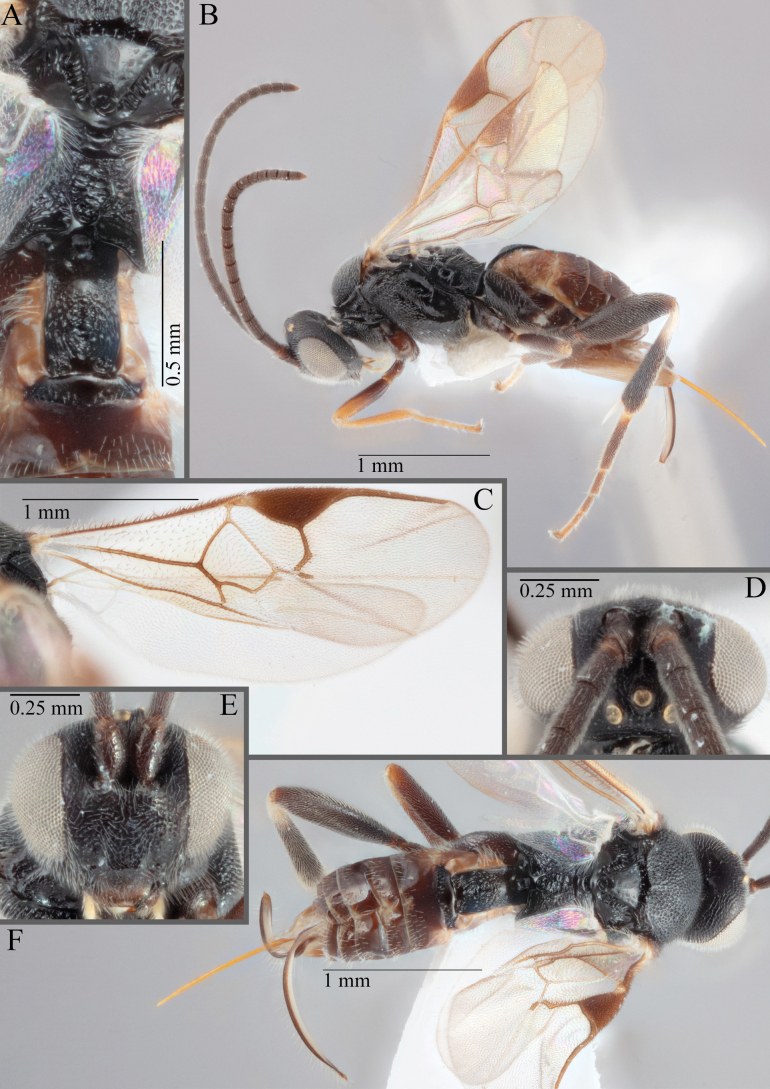
*Apantelesalatomicans* (holotype AUMIC430-18) **A** dorsal propodeum and T1–T3 **B** lateral habitus **C** fore and hindwing **D** dorsal head **E** anterior head **F** dorsal habitus.

Fig. 4B (distribution), Fig. 17 (holotype)

###### Type material.

***Holotype*.** Australia • ♀; QLD, Masthead Island; -23.537, 151.723; 5–7 Oct. 2008; QM/QPWS party leg.; Malaise trap; site 6 Casuarina camp site; BOLD Process ID: AUMIC430-18; QM: T208349. ***Paratypes*.** Australia • ♀; NSW, Bendemeer; -30.819, 151.142; 19 Dec. 2020–4 Jan. 2021; A. Goodwin, R. Noakes leg.; Malaise trap; BOLD Process ID: AUMIC628-23; AM: K.647425. • ♀; as previous except: BOLD Process ID: AUMIC630-23; AM: K.647426. • ♀; as previous except: BOLD Process ID: AUMIC631-23; QM: T261232. • ♀; as previous except: BOLD Process ID: AUMIC632-23; QM: T261233. • ♂; QLD, Chinchilla Botanic Parkland; -26.7393, 150.629; 08 Oct. 2020; E. Fagan-Jeffries leg.; Sweeping; BOLD Process ID: OZBOL414-21; QM: T261234. • ♀; QLD, Masthead Island; -23.537, 151.721; 5–7 Oct. 2008; QM/QPWS party leg.; Malaise trap; site 7 *Casuarina* forest; BOLD Process ID: AUMIC074-18; QM: T208348. • ♀; QLD, Prospect Creek State School; -24.4218, 150.43; 6 Oct.–4 Nov. 2020; E. Fagan-Jeffries & Prospect Creek State School leg.; Malaise trap; BOLD Process ID: AUMIC640-23; QM: T261235. • ♀; as previous except: BOLD Process ID: AUMIC641-23; QM: T261236. • ♀; as previous except: BOLD Process ID: AUMIC643-23; QM: T261237. • ♀; as previous except: BOLD Process ID: AUMIC644-23; QM: T261238. • ♀; as previous except: BOLD Process ID: AUMIC645-23; QM: T261239. • ♀; as previous except: BOLD Process ID: AUMIC646-23; AM: K.647427. • ♀; as previous except: BOLD Process ID: AUMIC647-23; QM: T261240. • ♀; as previous except: BOLD Process ID: AUMIC648-23; QM: T261241. • ♀; as previous except: BOLD Process ID: AUMIC649-23; QM: T261242. • ♀; as previous except: BOLD Process ID: AUMIC667-23; QM: T261243. • ♀; as previous except: BOLD Process ID: AUMIC669-23; QM: T261244. • ♀; as previous except: BOLD Process ID: AUMIC670-23; AM: K.647428. • ♀; as previous except: BOLD Process ID: AUMIC671-23; QM: T261245. • ♀; as previous except: BOLD Process ID: AUMIC657-23; QM: T261246. • ♀; as previous except: BOLD Process ID: AUMIC660-23; QM: T261247. • ♀; as previous except: BOLD Process ID: AUMIC661-23; QM: T261248. • ♀; as previous except: BOLD Process ID: AUMIC663-23; QM: T261249. • ♀; as previous except: BOLD Process ID: AUMIC633-23; QM: T261250. • ♀; as previous except: BOLD Process ID: AUMIC635-23; QM: T261251. • ♀; as previous except: BOLD Process ID: AUMIC636-23; QM: T261252. • ♀; as previous except: BOLD Process ID: AUMIC638-23; QM: T261253. • ♀; as previous except: BOLD Process ID: AUMIC639-23; QM: T261254. • ♂; as previous except: BOLD Process ID: AUMIC673-23; AM: K.647429. • ♀ (head detached); QLD, Springsure; -24.115, 148.087; 8–15 Mar. 2022; Springsure State School students leg.; Malaise trap; Insect Investigators; BOLD Process ID: ASMII486-22; QM: T261184. • ♂; as previous except: BOLD Process ID: ASMII482-22; QM: T261186. • ♂; as previous except: 22–29 Mar. 2022; BOLD Process ID: ASMII550-22; QM: T261185.

###### Diagnostic description.

***Size***: Total body length: 3.0 mm; fore wing length: 2.6 mm. Head: anterior scape colour similar or only very slightly paler than head colour; F2L/W ratio: 2.2; F14L/W ratio: 1.1. ***Mesosoma***: scutoscutellar sulcus with nine pits; mesoscutellar disc mostly smooth, or with very shallow scattered indentations; propodeal areola complete, or mostly so; propodeum mostly rugose; coxae colour (pro, meso, meta): dark all; metafemur colour mostly dark. ***Wings***: centre of pterostigma pigmented to same degree as the outer edges; fore wing r vein length/2RS vein length ratio: 1.4. ***Metasoma***: T1 shape mostly parallel, T1 medial length/anterior width between 1–2 × longer than wide; mostly rugose; T2 mostly smooth; ovipositor sheath length/metatibia length ratio: 1.1.

*Apantelesalatomicans* can be separated from most species of *Apanteles* in Australia that have a dark metacoxa and metafemur, and uniformly pigmented pterostigma by having the hind tibia mostly dark with a bright, discrete pale band in the proximal 1/3, and by having infuscation on the fore wing which is restricted to area around veins r and 1CUb, and the basal cell of fore wing comparatively less densely setose than found in *A.lamingtonensis* and *A.ferripulvis*. We recommend separating *A.alatomicans* from *A.hades* and *A.magicus* using COI or *wg* barcodes and evaluating the placement of the sequence in a phylogeny amongst the validated references provided here.

###### Etymology.

This species was named by students in year 9 at Springsure State School in QLD, who in year 7 ran the Malaise trap that collected several of the paratype specimens. The epithet should be treated as an adjective and is formed from the Latin ‘alatus’ (furnished with wings) and ‘micans’ (twinkling) and the wasp was affectionately given the nickname “Mr Twinkle Wings” in the taxonomy workshop. In the words of the class teacher, Peter Spencer: “The reason for naming refers to the beauty of the wasp and the shiny/twinkle of its wings particularly, and the fact the students also believe it is found in a place representing Australian beauty – the outback.”

###### Distribution.

*Apantelesalatomicans* is known from multiple locations in eastern QLD.

###### Molecular information.

Sequences of *Apantelesalatomicans* currently form BIN BOLD:ADL2797. The COI sequences are at least 5% divergent from any of the other species treated here, or any available sequence on BOLD. Other than the *wg* sequence of AUMIC673-23, which is likely a lab contamination, the *wg* sequence of the holotype is ≥ 3 bp different to any other species and is identical within *A.alatomicans*. Ignoring the likely contaminant, six species delimitation methods delimited *A.alatomicans* as a discrete species, however *wg*ASAP analysis grouped the sequences with *A.hades*.

##### ﻿﻿ Apanteles allapsus

Taxon classificationAnimaliaHymenopteraBraconidae

Slater-Baker, Fagan-Jeffries, Fernández-Triana, Portmann & Oestmann sp. nov.

53447515-79BD-5666-B5AB-586D9C2EBA67

https://zoobank.org/4D96E39D-2F13-4824-A589-1E7901671634

**Figure 18.**
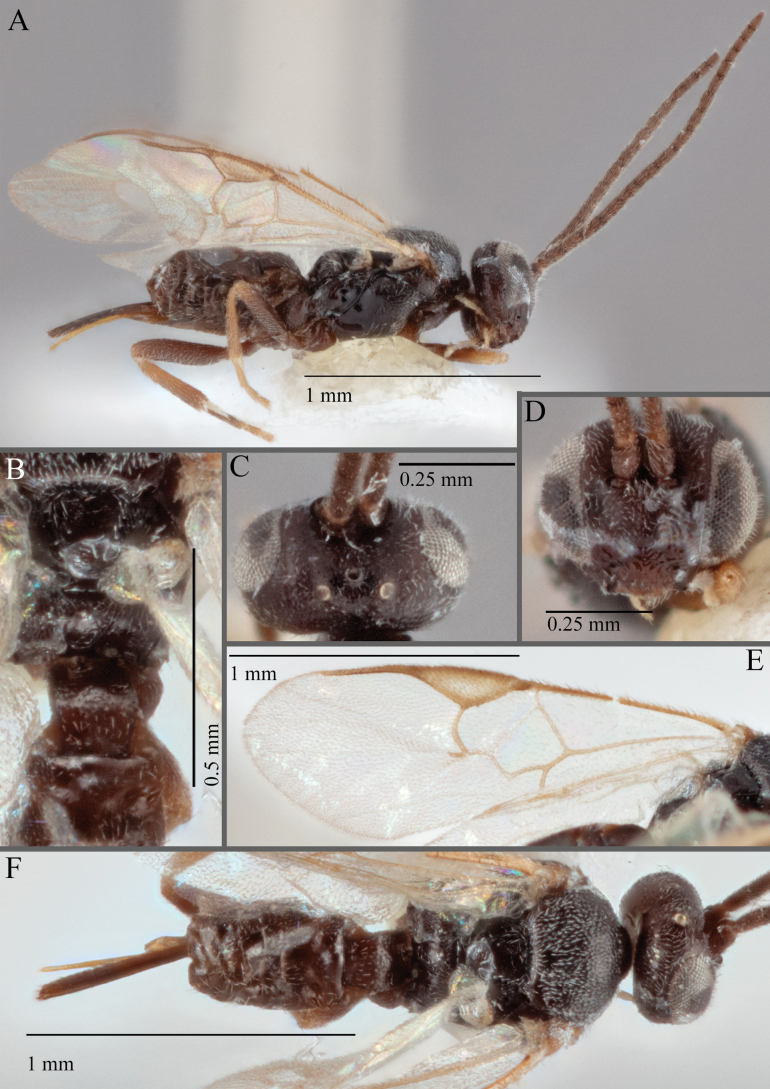
*Apantelesallapsus* (holotype AUMIC048-18) **A** lateral habitus **B** dorsal propodeum and T1–T3 **C** dorsal head **D** anterior head **E** fore wing **F** dorsal habitus.

Fig. 6D (distribution), Fig. 18 (holotype)

###### Type material.

***Holotype*.** Australia • ♀; NT, Gregory NP, Limestone Gorge; -16.0503, 130.402; 6–13 Jun. 2001; ME Irwin, FD Parker, C Lambkin leg.; Malaise in dry gully; BOLD Process ID: AUMIC048-18; ANIC: 32-130194.

###### Diagnostic description.

***Size***: Total body length: 1.6 mm; fore wing length: 1.7 mm., ***Head***: anterior scape colour similar or only very slightly paler than head colour; F2L/W ratio: 1.9; F14L/W ratio: 1.2. ***Mesosoma***: scutoscutellar sulcus with ten pits; mesoscutellar disc mostly smooth, or with very shallow scattered indentations; propodeal areola complete, or mostly so; propodeum mostly smooth; coxae colour (pro, meso, meta): dark all; metafemur colour mostly dark. ***Wings***: centre of pterostigma paler (more hyaline) than outer edges; fore wing r vein length/2RS vein length ratio: 1.3. ***Metasoma***: T1 shape mostly parallel, T1 medial length/anterior width between 1–2 × longer than wide; mostly smooth; T2 mostly smooth; ovipositor sheath length/metatibia length ratio: 1.0.

*Apantelesallapsus* can be separated from the other species of *Apanteles* in Australia that have a dark metacoxa and metafemur and the pterostigma with outer border darker than centre, centre of pterostigma pale or transparent/hyaline by having most fore wing veins pigmented, including 1CUa, 1CUb, M+CU, and 1M pigmented for most of their lengths, T1 smooth; propodeal areola slightly rounded, and the propodeum relatively smooth.

*Apantelesallapsus* can be separated from the described species known from Pakistan as follows: from *A.angustibasis* (Gahan, 1925) by having the ovipositor sheath/metatibia ratio ~ 1.0, and the antenna similar length to the body (*A.angustibasis* has the ovipositor and antenna quite short – images of the holotype examined); from *A.angalti* Muesebeck, 1956 by having the T2 comparatively more transverse (images of the holotype examined), from *A.cypris* Nixon, 1965 and from *A.significans* (Walker, 1860) by having the pterostigma pale/hyaline in the centre (both *A.cypris* and *A.signficans* have the pterostigma uniformly pigmented with a pale spot in proximal corner; images of the holotype of *A.cypris* were examined, and illustration of *A.significans*Nixon (1965; fig. 58)); and from *A.telon* Nixon, 1965 (original description used) by more complete carination of the propodeal areola (*A.telon* has the areola reduced), a shorter ovipositor sheath (*A.telon* described as having the sheath 1.5 × longer than metatibia), and much smaller in size (*A.telon* is > 3 mm long). We are unable to locate the description or images of *A.quadratus* Anjum & Malik, 1978, and therefore cannot compare versus this species.

###### Etymology.

The species epithet is a participle in the nominative case and is Latin for a ‘gliding approach/a flowing near’ and is an oblique reference to the comparatively smooth T1 and propodeum of this species, and also to the collection locality of Limestone Gorge.

###### Distribution.

*Apantelesallapsus* is known from one specimen from northern NT, and potentially from Pakistan (see note in ‘Molecular Information’.

###### Molecular information.

The holotype of *Apantelesallapsus* is currently in BIN BOLD:ADL2832, which also contains three private sequences from male specimens originating from a Malaise trap in Pakistan, with a maximum divergence of 1.4%. We have not been able to examine the specimens (other than images on BOLD), but this low divergence suggests that *A.allapsus* may also occur in Pakistan. The COI sequences in that BIN are at least 6% divergent from any of the other species treated here, or any available sequence on BOLD. The *wg* sequence of the holotype is ≥ 5 bp different to any other species. All species delimitation methods delimited *A.allapsus* as a discrete species.

##### ﻿﻿ Apanteles amicalis

Taxon classificationAnimaliaHymenopteraBraconidae

Slater-Baker, Fagan-Jeffries, Fernández-Triana, Portmann & Oestmann sp. nov.

A2AF1EC8-08DE-511A-81FF-44C6B8523895

https://zoobank.org/3B1CDE73-2E28-4FDD-872E-9EB5CC2BE153

**Figure 19.**
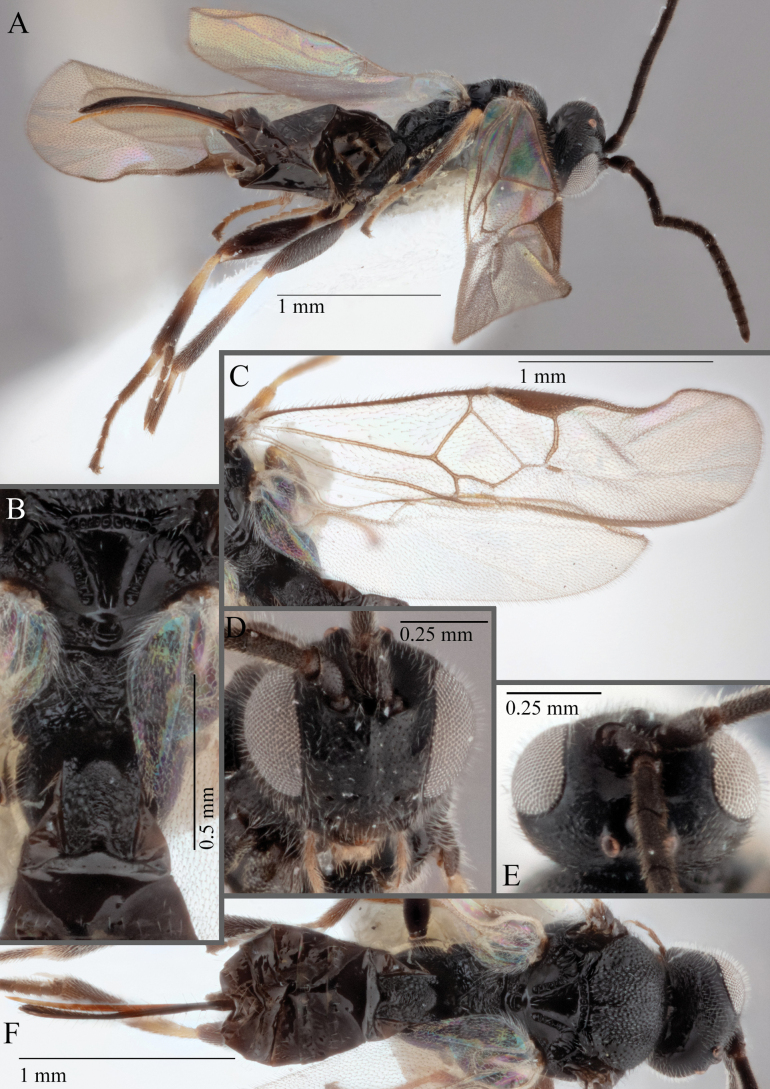
*Apantelesamicalis* (holotype AUMIC910-23) **A** lateral habitus **B** dorsal propodeum and T1–T3 **C** fore and hindwing **D** anterior head **E** dorsal head **F** dorsal habitus.

Fig. 4A (distribution), Fig. 19 (holotype)

###### Type material.

***Holotype*.** Australia • ♀; ACT, Canberra, Black Mtn Res; -35.2795, 149.105; 9–30 Apr. 2020; KM Bayless leg.; Malaise trap over stream near ANBG pumphouse; BOLD Process ID: AUMIC910-23; ANIC: 32-085529.

###### Diagnostic description.

***Size***: Total body length: 2.5 mm; fore wing length: 2.6 mm. ***Head***: anterior scape colour similar or only very slightly paler than head colour; F2L/W ratio: 3.6; F14L/W ratio: 1.5. ***Mesosoma***: scutoscutellar sulcus with eight pits; mesoscutellar disc mostly smooth; propodeal areola complete, or mostly so; propodeum mostly smooth posteriorly, mostly rugose anteriorly; coxae colour (pro, meso, meta): dark all; metafemur colour mostly dark. ***Wings***: centre of pterostigma pigmented to same degree as the outer edges; fore wing r vein length/2RS vein length ratio: 1.1. ***Metasoma***: T1 shape mostly parallel, T1 medial length/anterior width between 1–2 × longer than wide; T1 mostly rugose; T2 mostly smooth; ovipositor sheath length/metatibia length ratio: 1.2.

*Apantelesamicalis* can be separated from the other species of *Apanteles* in Australia that have a dark metacoxa and metafemur and the pterostigma uniformly pigmented (no pale centre or large pale spot on the proximal corner), the ovipositor sheath lengths > 0.8 × metatibia length and the antenna of similar size to the body length, by having T1 with strong sculpture over at most of posterior 1/2 of tergite, the mesoscutellar disc mostly smooth, fore wing veins 1M and 1CUa of similar pigmentation, and T2 posterior length/width ratio ~ 5.0.

This species is not diagnosable against *A.persephone* Nixon, 1965 at the current time, but as *A.persephone* was described from two specimens near Perth in WA, and we have only a single specimen from Canberra ACT, we think it is unlikely that it is the same species. However, we acknowledge that synonomy may be required in the future if DNA is able to be obtained from the holotype or paratype of *A.persephone*.

###### Etymology.

The species epithet is from a Latin adjective meaning friendly and refers to the friendly exchange of specimens between dipterist K.M. Bayless, the collector of the holotype, and the authors; they thank him for generously sharing bulk Hymenoptera from his many Malaise traps!

###### Distribution.

*Apantelesamicalis* is known from one specimen from the ACT.

###### Molecular information.

The holotype of *Apantelesamicalis* is the only sequence currently in BIN BOLD:AFF0137, and the COI sequences in that BIN are at least 6% divergent from any of the other species treated here, or any available sequence on BOLD. The *wg* sequence of the holotype is 1 bp different to the undescribed lineage *A.* sp. MRSB26, and ≥ 5 bp different to any other described species. Six of the molecular delimitation methods separated *A.amicalis* as a distinct species; the *wg*ASAP analysis grouped the species with *A.pellucidus* and *A.* sp. MRSB26.

##### ﻿﻿ Apanteles apollo

Taxon classificationAnimaliaHymenopteraBraconidae

Slater-Baker, Fagan-Jeffries, Fernández-Triana, Portmann & Oestmann sp. nov.

64F8536F-C167-5709-B32D-E9D3C2C46605

https://zoobank.org/6B507DDA-208A-4C30-A949-8C272E55EE59

**Figure 20.**
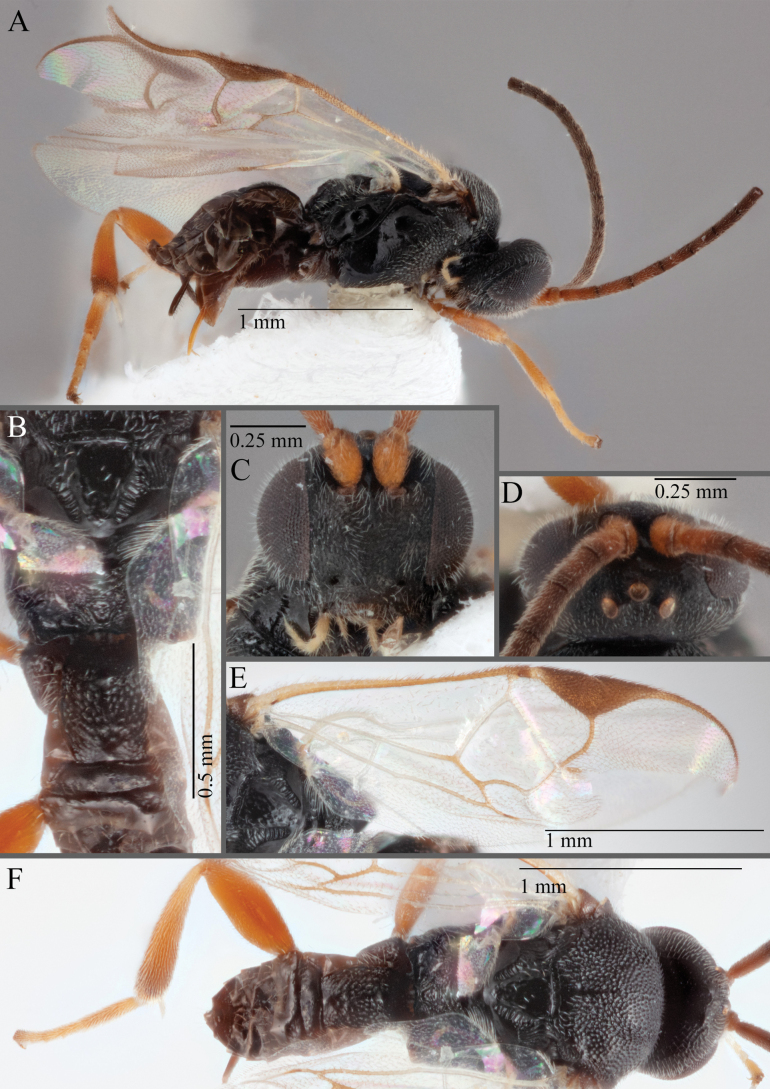
*Apantelesapollo* (holotype AUMIC051-18) **A** lateral habitus **B** dorsal propodeum and T1–3 **C** anterior head **D** dorsal head **E** fore wing **F** dorsal habitus.

Fig. 4B (distribution), Fig. 20 (holotype)

###### Type material.

***Holotype*.** Australia • ♀; NT, Gregory Nat Park 8.3 km N Humbert Junction; -16.0406, 130.455; 6–12 Jun. 2001; ME Irwin, FD Parker, C Lambkin leg.; Malaise in dry bed nr flowing ck; BOLD Process ID: AUMIC051-18; ANIC: 32-130197.

###### Diagnostic description.

***Size***: Total body length: 2.4 mm; fore wing length: 2.2 mm. ***Head***: anterior scape colour much paler, dramatically different colour than head (a bright orange in holotype); F2L/W ratio: 2.8. ***Mesosoma***: scutoscutellar sulcus with 11 pits; mesoscutellar disc mostly smooth; propodeal areola complete, or mostly so; propodeum mostly rugose; coxae colour (pro, meso, meta): dark all; metafemur colour uniformly pale (a bright orange in the holotype). ***Wings***: centre of pterostigma pigmented to same degree as the outer edges; fore wing r vein length/2RS vein length ratio: 1.5. ***Metasoma***: T1 shape mostly parallel, T1 medial length/anterior width between 1–2 × longer than wide; T1 mostly rugose; T2 mostly smooth; hypopygium without defined ventral pleats; ovipositor sheath length/metatibia length ratio: 0.4.

*Apantelesapollo* can be separated from other species of *Apanteles* in Australia that have the metacoxa dark and the metafemur uniformly pale by the ovipositor sheaths short (< 0.5 × metatibia length), the hypopygium without defined ventral pleats and T1 and T2 as in Fig. 20B. It is also one of the few species with the scape considerably paler than the head colour when viewed from the anterior side of the head.

Due to this species potentially being identified as *Parapanteles*, we also diagnose it against the three species of *Parapanteles* known from Australia. Images of the holotypes and the treatment in Nixon (1965) were referred to. *Apantelesapollo* can be separated from all three species by having the metafemur pale (all *Parapanteles* known in Australia have the metafemur dark) and additionally from *Parapantelesfolia* (Nixon, 1965) by having the pterostigma uniformly pigmented (*Apantelesfolia* has the pterostigma more hyaline in the centre); from *Parapanteleshyposidrae* (Wilkinson, 1928) by having a comparatively narrower T1; and from *Parapantelesmasoni* Austin & Dangerfield, 1992 by having the propodeum rugose (*P.masoni* has the propodeum relatively smooth with complete carina).

###### Etymology.

This species is named for Apollo, an Olympian god from Greek mythology.

###### Distribution.

*Apantelesapollo* is currently only known from one specimen from Gregory National Park in the NT.

###### Molecular information.

*Apantelesapollo* is currently BIN BOLD:ADL5064. The COI sequences are at least 7% divergent from any of the other species treated here, or any available sequence on BOLD. The *wg* sequence of the holotype is ≥ 26 bp different to any other species. All molecular species delimitation methods separated *Apantelesapollo* as a distinct species. The phylogenetic position of the species is unresolved, falling outside the main *Apanteles* clade in the concatenated analysis of COI and *wg* of Australian species (Fig. 2). However, in the tree of global *Apanteles*COI sequences, the sequences cluster within the larger clade that contains many Australian species.

###### Remarks.

This species is potentially better placed in *Parapanteles* because of the short ovipositor sheaths and the comparatively solid hypopygium without ventral pleats. However, *Parapanteles* is currently poorly defined and until a more conclusive revision of the genus is completed, we feel it is more useful to place *A.apollo* in *Apanteles* because of the molecular data clustering it with morphologically ‘true’ *Apanteles*. We note, however, that this species would be an ideal candidate to include in phylogenomic studies of microgastrine genera limits as it may well belong in a different genus.

##### ﻿﻿ Apanteles apricus

Taxon classificationAnimaliaHymenopteraBraconidae

Slater-Baker, Fagan-Jeffries, Fernández-Triana, Portmann & Oestmann sp. nov.

EB797968-2F98-5A7B-95EB-D38B648AF9D1

https://zoobank.org/CC0BD92F-FCF6-4E25-AD54-EB587435973D

**Figure 21.**
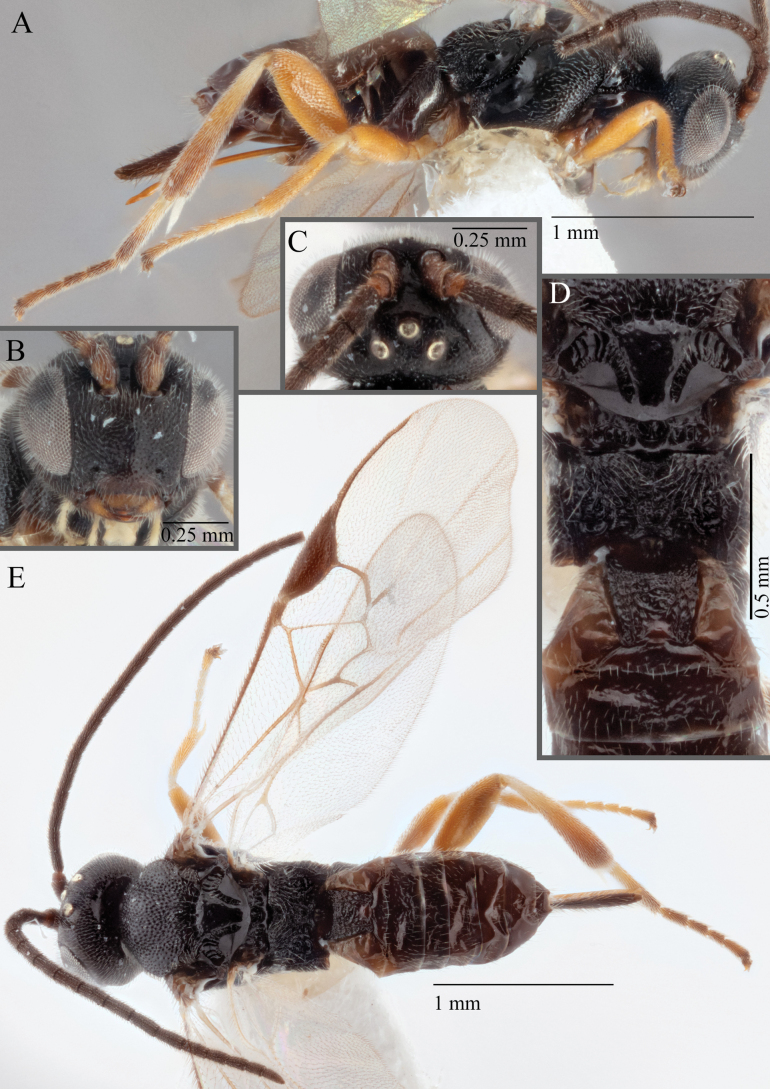
*Apantelesapricus* (holotype AUMIC068-18) **A** lateral habitus **B** anterior head **C** dorsal head **D** dorsal propodeum and T1–3 **E** dorsal habitus and wings.

Fig. 4A (distribution), Fig. 21 (holotype)

###### Type material.

***Holotype*.** Australia • ♀; QLD, Cainbable Quarry, OF; -28.145, 153.113; 03–19 Feb. 2009; F. Turco leg.; Malaise trap; BOLD Process ID: AUMIC068-18; QM: T208356. ***Paratype*.** Australia • ♀; WA, Kalumburu; -14.292, 126.642; 15–22 Mar. 2022; Kalumburu Remote Community School students leg.; Malaise trap; Insect Investigators; BOLD Process ID: ASMII13755-22; WAM: 130573.

###### Diagnostic description.

***Size***: Total body length: 2.8 mm; fore wing length: 2.8 mm. ***Head***: anterior scape colour moderately paler than head colour; F2L/W ratio: 3.2; F14L/W ratio: 1.9. ***Mesosoma***: scutoscutellar sulcus with seven pits; mesoscutellar disc mostly smooth, or with very shallow scattered indentations; propodeal areola complete, or mostly so; propodeum mostly rugose; coxae colour (pro, meso, meta): dark all; metafemur colour mostly pale. ***Wings***: centre of pterostigma pigmented to same degree as the outer edges; fore wing r vein length/2RS vein length ratio: 1.1. ***Metasoma***: T1 shape narrowing distally, T1 medial length/anterior width between 1–2 × longer than wide; T1 mostly rugose; T2 mostly smooth; ovipositor sheath length/metatibia length ratio: 0.8.

*Apantelesapricus* can be separated from other species of *Apanteles* in Australia with a dark metacoxa by having the metafemur uniformly pale, the ovipositor sheath 0.8–1.0 × metatibia length and hypopygium with clearly defined ventral pleats, and the propodeum with strong rugose sculpture, areola not well defined amongst strong sculpture and T1 slightly narrowing posteriorly.

###### Etymology.

The species epithet is a Latin adjective meaning sunny and relates to the sunshine-filled places the species is currently known from, as well as its yellowish metafemur.

###### Distribution.

*Apantelesapricus* is currently known from two specimens collected in northern Australia, one near Brisbane and one in northeastern WA.

###### Molecular information.

*Apantelesapricus* is currently BIN BOLD:ADL3302. The COI sequences are at least 6% divergent from any of the other species treated here, or any available sequence on BOLD. The *wg* sequence of the holotype is ≥ 17 bp different to any other species. All molecular species delimitation methods separated *Apantelesapricus* as a distinct species.

##### ﻿﻿ Apanteles artemis

Taxon classificationAnimaliaHymenopteraBraconidae

Slater-Baker, Fagan-Jeffries, Fernández-Triana, Portmann & Oestmann sp. nov.

CBE0DE6B-95C2-5BC0-85BA-87EAF2F62EDA

https://zoobank.org/8527B27F-5886-442E-BD21-0933BEBBFA78

**Figure 22.**
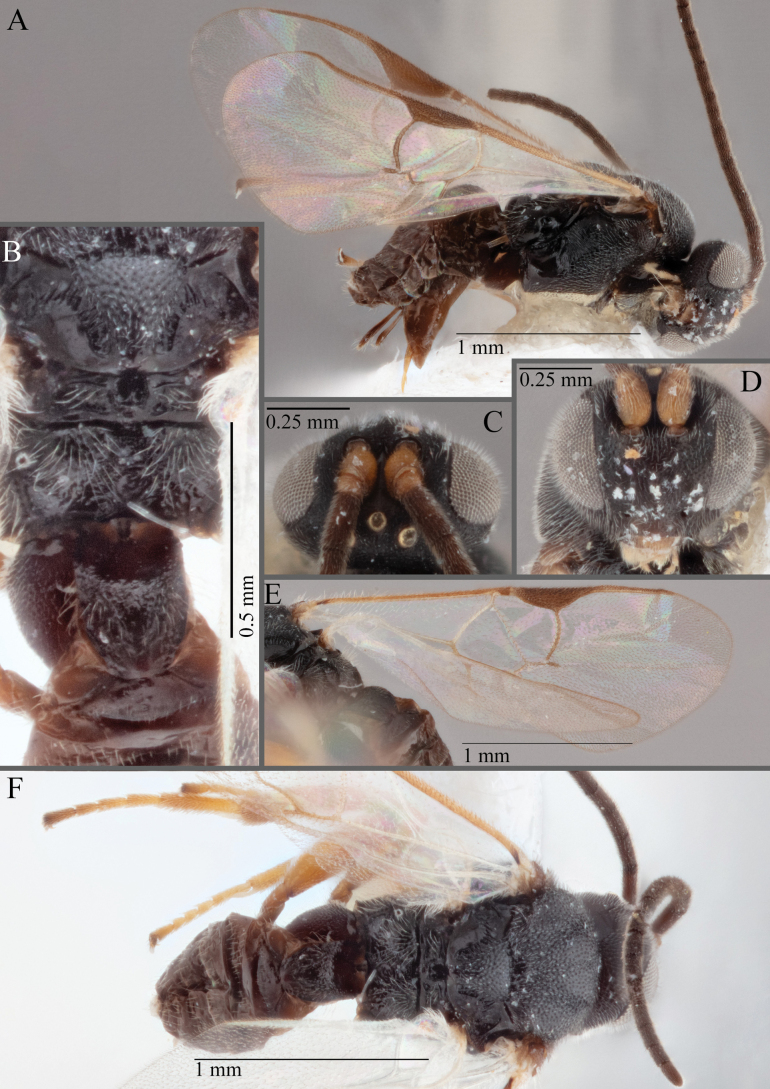
*Apantelesartemis* (holotype AUMIC479-18) **A** lateral habitus **B** dorsal propodeum and T1– 2 **C** dorsal head **D** anterior head **E** fore and hind wing **F** dorsal habitus.

Fig. 4C (distribution), Fig. 22 (holotype)

###### Type material.

***Holotype*.** Australia • ♀; NT, Gregory NP, 5.7 km N Humbert Junction; -16.0622, 130.451; 6–12 Jun. 2001; ME Irwin, FD Parker, C Lambkin leg.; Malaise in dry creek bed; BOLD Process ID: AUMIC479-18; ANIC 32-130236.

###### Diagnostic description.

***Size***: Total body length: 2.5 mm; fore wing length: 3.3 mm. ***Head***: anterior scape colour much paler, dramatically different colour than head; F2L/W ratio: 2.4; F14L/W ratio: 1.5., scutoscutellar sulcus with 11 pits; mesoscutellar disc punctate throughout; propodeal areola complete, or mostly so; propodeum mostly smooth posteriorly, mostly rugose anteriorly; coxae colour (pro, meso, meta): dark all; metafemur colour mostly pale; metafemur colour uniformly pale (a very light brown/orange in the holotype). ***Wings***: centre of pterostigma pigmented to same degree as the outer edges; fore wing r vein length/2RS vein length ratio: 1.7. ***Metasoma***: T1 shape mostly parallel, then narrowing in distal 1/3., T1 medial length/anterior width between 1–2 × longer than wide; T1 mostly rugose in distal 1/2, mostly in smooth basal 1/2; T2 mostly smooth; hypopygium without defined ventral pleats; ovipositor sheath length/metatibia length ratio: 0.3.

*Apantelesartemis* can be separated from other species of *Apanteles* in Australia that have the metacoxa dark and the metafemur uniformly pale by the ovipositor sheaths short (< 0.5 × metatibia length), the hypopygium without defined ventral pleats, T1 narrowing and T2 as in Fig. 22B. It is also one of the few species with the scape considerably paler than the head colour in when viewed from the anterior of the head.

Due to this species potentially being identified as *Parapanteles*, we also diagnose it against the three species of *Parapanteles* known from Australia. *Apantelesartemis* can be separated from all three species by having the metafemur pale (all *Parapanteles* known in Australia have the metafemur dark) and by having T1 more strongly narrowing posteriorly (all *Parapanteles* known in Australia have a relatively parallel T1).

###### Etymology.

This species is named for Artemis, an Olympian goddess from Greek mythology.

###### Distribution.

*Apantelesartemis* is currently only known from one specimen from Gregory National Park in the NT.

###### Molecular information.

*Apantelesartemis* is currently represented by sequences in BIN BOLD:ADL5654. The COI sequences are at least 8% divergent from any of the other species treated here, or any available sequence on BOLD. The *wg* sequence of the holotype is ≥ 15 bp different to any other species. All molecular species delimitation methods separated *Apantelesartemis* as a distinct species. The phylogenetic position of the species is unresolved, falling outside the main *Apanteles* clade in the concatenated analysis of COI and *wg* of Australian species (Fig. 2) and in the COI phylogeny of global *Apanteles* (Fig. 3). Based on the molecular data, *Apantelesartemis* is closely related to a delimited species from South Australia (*Apanteles* sp. MRSB03) which was not described because no female specimens were available.

###### Remarks.

This species is potentially better placed in *Parapanteles* because of the short ovipositor sheaths and the comparatively solid hypopygium without ventral pleats. However, *Parapanteles* is currently poorly defined and until a more conclusive revision of the genus is completed, we feel it is more useful to place *A.artemis* in *Apanteles* because of the molecular data clustering it with morphologically ‘true’ *Apanteles*. We note, however, that this species would be an ideal candidate to include in phylogenomic studies of microgastrine genera limits as it may well belong in a different genus.

##### ﻿﻿ Apanteles aurantius

Taxon classificationAnimaliaHymenopteraBraconidae

Slater-Baker, Fagan-Jeffries, Fernández-Triana, Portmann & Oestmann sp. nov.

FF79CC3B-90FD-5F01-A329-31A0430ED02E

https://zoobank.org/3C611CB3-163E-4560-A290-F1EE6A4C9C69

**Figure 23.**
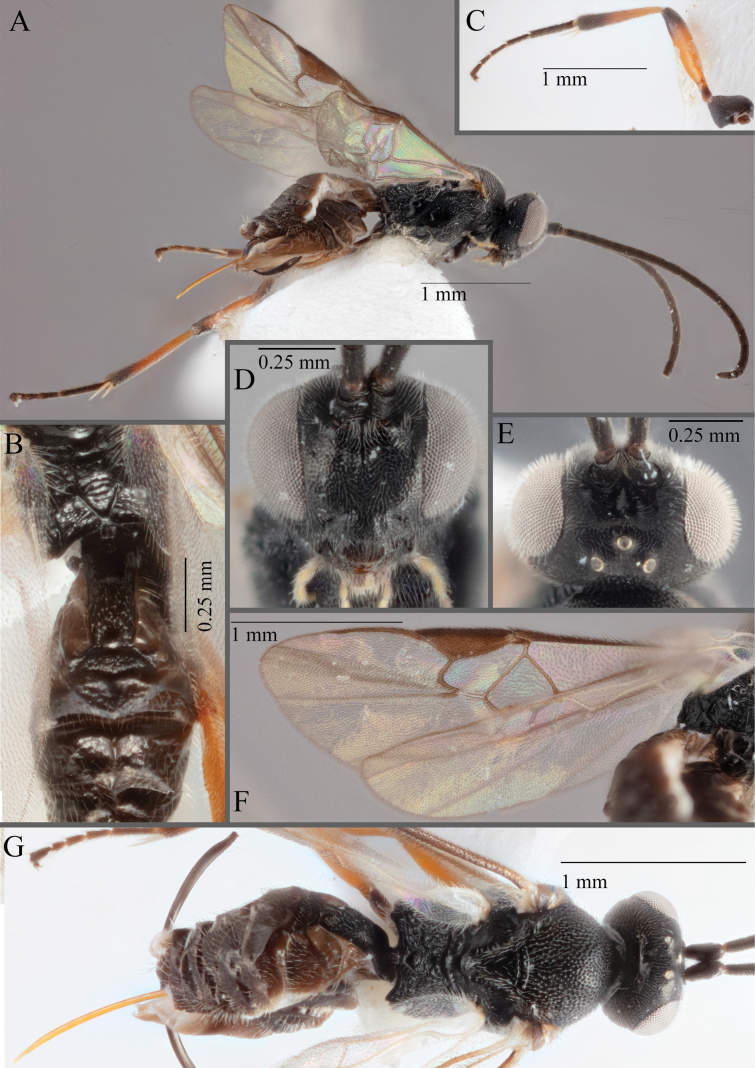
*Apantelesaurantius***A** lateral habitus (paratype AUMIC238-18) **B** dorsal propodeum and T1–3 (paratype AUMIC1063-24) **C** hind leg (paratype AUMIC238-18) **D** anterior head (paratype AUMIC238-18) **E** dorsal head (paratype AUMIC238-18) **F** fore and hind wing (paratype AUMIC238-18) **G** dorsal habitus (paratype AUMIC238-18).

Fig. 4B (distribution), Fig. 23 (holotype)

###### Type material.

***Holotype*.** Australia • ♀; QLD, Samsonvale Cemetery, 8.5 km SSE Dayboro; -27.2703, 152.856; 50 m; 22 Oct.–14 Nov. 2014; S. Wright leg.; Malaise trap; Casuarina/open forest; BOLD Process ID: AUMIC411-18; QM: T208366. ***Paratypes*.** Australia • ♂; ACT, Canberra, Cook, 8 Moss Street; -35.261, 149.059; 632 m; 10 Apr. 2011; P.Hebert, R.Labbee, V.Levesque-Beaudin, J.McCormick, J.Sones, J.Webb leg.; BOLD Process ID: AACTA4990-20; ANIC: 32-085566. • ♀; NSW, Eden, Bungo Street; -37.0611, 149.903; 21–27 Dec. 2005; C. Stephens leg.; Malaise trap; in exotic native garden blend nr Eucalypt Forest; BOLD Process ID: AUMIC238-18; ANIC: 32-085555. • ♀; QLD, Charters Towers; -20.058, 146.272; 1–8 Mar. 2022; Columba Catholic College students leg.; Malaise trap; Insect Investigators; BOLD Process ID: ASMII204-22; QM: T261167. • ♂; QLD, Eungella National Park, Credition Loop Road; -21.1908, 148.542; 23 Nov. 2019; E. Fagan-Jeffries, J. B. Dorey & P. Ruhr leg.; Sweeping vegetation; BOLD Process ID: AUMIC1214-24; QM: T261196. • ♂; QLD, Gladstone; -23.8522, 151.258; 29 m; 04 Oct. 2020; E. Fagan-Jeffries leg.; Sweeping; BOLD Process ID: OZBOL422-21; QM: T261197. • ♀; QLD, Samsonvale Cemetery, 8.5 km SSE Dayboro; -27.2703, 152.856; 50 m; 22 Oct.–14 Nov. 2014; S. Wright leg.; Malaise trap; Casuarina/open forest; BOLD Process ID: AUMIC088-18; QM: T208365. • ♀; QLD, Toowoomba, Kearney Springs Historical Park; -27.5941, 151.942; 665 m; 26 Nov. 2019; E. Fagan-Jeffries, J. B. Dorey & P. Ruhr leg.; Sweeping; BOLD Process ID: AUMIC1063-24; QM: T261198. • ? abdomen missing; QLD, Townsville, Hermit Park; -19.283, 146.801; 10 m; 21 Jul. 2011; G. V. Cocks leg.; BOLD Process ID: HYQTB106-11; QM: T261264. • ♂; as previous except: 03 Dec. 2016; UV Light Trap; BOLD Process ID: GCQT1055-17; QM: T261265. • ♂; as previous except: 22 Jul. 2016; UV Light Trap; BOLD Process ID: GCQT1101-17; QM: T261266. • ♂; as previous except: 11 Sep. 2011; BOLD Process ID: HYQTB145-12; QM: T261267. • ♀; SA, SE Kangaroo Island, Heritage Agreement 1302 S boundary ~ 5.5 km NW D`Estrees Bay; -34.0912, 137.557; 27 Dec. 2017; RV Glatz leg.; swept from foliage of *Leptospermumcontinentali* or dry grass at base of bush; BOLD Process ID: AUMIC859-23; QM: T261187. • ♀; WA, Newman; -23.363, 119.734; 15–22 Mar. 2022; South Newman Primary School students leg.; Malaise trap; Insect Investigators; BOLD Process ID: ASMII6178-22; WAM: 130552. • ♀; as previous except: 22–29 Mar. 2022; BOLD Process ID: ASMII6217-22; WAM: 130553.

###### Diagnostic description.

***Size***: Total body length: 2.7 mm; fore wing length: 2.5 mm. ***Head***: anterior scape colour similar or only very slightly paler than head colour; F2L/W ratio: 2.9; F14L/W ratio: 1.3. ***Mesosoma***: scutoscutellar sulcus with ten pits; mesoscutellar disc punctate throughout; propodeal areola complete, or mostly so; propodeum mostly rugose; coxae colour (pro, meso, meta): dark all; metafemur colour mostly Pale with dark band in distal 1/3. ***Wings***: centre of pterostigma pigmented to same degree as the outer edges; fore wing r vein length/2RS vein length ratio: 1.4. ***Metasoma***: T1 shape mostly parallel, T1 medial length/anterior width narrow, >2 × longer than wide; T1 mostly rugose; T2 mostly smooth; ovipositor sheath length/metatibia length ratio: 1.0.

*Apantelesaurantius* can be separated from other species of *Apanteles* in Australia by the distinctive colouration of the metafemur (mostly pale/orange with a dark area distally).

###### Etymology.

The species epithet is a Latin adjective meaning ‘orange’ and refers to the bright orange colour on the metafemur and metatibia.

###### Distribution.

*Apantelesaurantius* has a broad distribution, with collection records down the east coast of Australia, and isolated records in SA and WA.

###### Molecular information.

*Apantelesaurantius* contains sequences currently in BIN BOLD:AAG8155. The COI sequences are at least 10% divergent from any of the other species treated here, or any available sequence on BOLD. The *wg* sequence of the holotype is ≥ 7 bp different to any other species. All molecular species delimitation methods separated *Apantelesaurantius* as a distinct species.

##### ﻿﻿ Apanteles auroralis

Taxon classificationAnimaliaHymenopteraBraconidae

Slater-Baker, Fagan-Jeffries, Fernández-Triana, Portmann & Oestmann sp. nov.

0E237F12-5429-5CBB-9D12-986579F6006F

https://zoobank.org/6D483022-CF76-4948-9BC5-9D281141BD35

**Figure 24.**
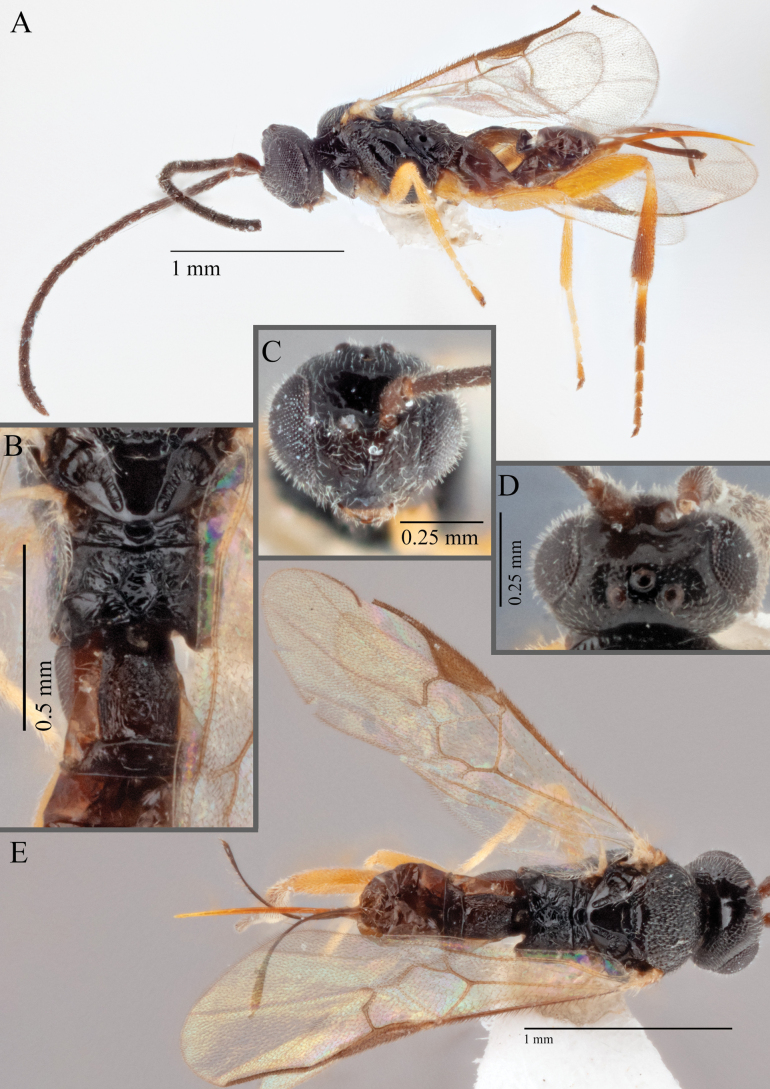
*Apantelesauroralis* (holotype AUMIC1526-24) **A** lateral habitus **B** dorsal propodeum and T1–3 **C** anterior head **D** dorsal head **E** dorsal habitus and wings.

Fig. 4B (distribution), Fig. 24 (holotype)

###### Type material.

***Holotype*.** Australia • ♀; QLD, Great Sandy NP, Bymien Picnic Area; -25.9536, 153.102; 05 Dec. 2017; D. Yeates & X. Li leg.; By hand; BOLD Process ID: AUMIC1526-24; QM. ***Paratypes*.** Australia • ♂; as holotype except: BOLD Process ID: AUMIC1523-24; ANIC: 32-085571. • ♂; as previous except: BOLD Process ID: AUMIC1524-24; ANIC: 32-085572. • ♂; as previous except: BOLD Process ID: AUMIC1525-24; QM: 32-085573. • ♀; QLD, Lamington NP; -28.148, 153.137; 13–23 Jan. 2007; C Lambkin, N. Starick leg.; Malaise trap; IBISCA Plot # IQ-300-A rainforest; BOLD Process ID: AUMIC077-18; QM: T208364.

###### Diagnostic description.

***Size***: Total body length: 2.1 mm; fore wing length: 2.2 mm. ***Head***: anterior scape colour moderately paler than head colour; F2L/W ratio: 2.6; F14L/W ratio: 1.3. ***Mesosoma***: scutoscutellar sulcus with 11 pits; mesoscutellar disc mostly smooth, or with very shallow scattered indentations; propodeal areola complete, or mostly so; propodeum mostly smooth; coxae colour (pro, meso, meta): pale, pale, dark; metafemur colour mostly pale. ***Wings***: centre of pterostigma pigmented to same degree as the outer edges; fore wing r vein length/2RS vein length ratio: 0.8. ***Metasoma***: T1 shape mostly parallel, T1 medial length/anterior width between 1–2 × longer than wide; mostly rugose; T2 mostly smooth; ovipositor sheath length/metatibia length ratio: 1.0.

*Apantelesauroralis* can be separated from most other species of *Apanteles* in Australia with a dark metacoxa, a completely pale metafemur and a uniformly pigmented pterostigma by the ovipositor sheath 0.8–1.0 × metatibia length and hypopygium with clearly defined ventral pleats. The species can be separated from *A.apricus* by the propodeum comparatively smoother, areola clearly defined and T1 parallel-sided; *A.apricus* has the propodeum with much stronger sculpturing and T1 slightly narrowing posteriorly.

###### Etymology.

The species epithet is an adjective formed from the Latin noun *aurora* meaning dawn/sunrise and relates to the strong colour contrast between the two yellow pro- and mesocoxae, and the dark metacoxa.

###### Distribution.

*Apantelesauroralis* is known from two sites in southern QLD.

###### Molecular information.

*Apantelesauroralis* contains sequences currently in BIN BOLD:ADL4428. The COI sequences are at least 11% divergent from any of the other species treated here, or any available sequence on BOLD. The *wg* sequence of the holotype is ≥ 3 bp different to any other species. Whilst the four COI delimitation methods separated *Apantelesauroralis* as a distinct species, there are two *wg* haplotypes within the species (1 bp difference), and the *wg*ASAP and PTP analyses grouped *A.auroralis* with *A.darthvaderi*.

##### ﻿﻿ Apanteles banrock

Taxon classificationAnimaliaHymenopteraBraconidae

Slater-Baker, Fagan-Jeffries, Fernández-Triana, Portmann & Oestmann sp. nov.

B9352B29-72EE-51D9-9635-E3DA59D73FB1

https://zoobank.org/16EE8822-6408-400F-A835-A3B1432876C0

**Figure 25.**
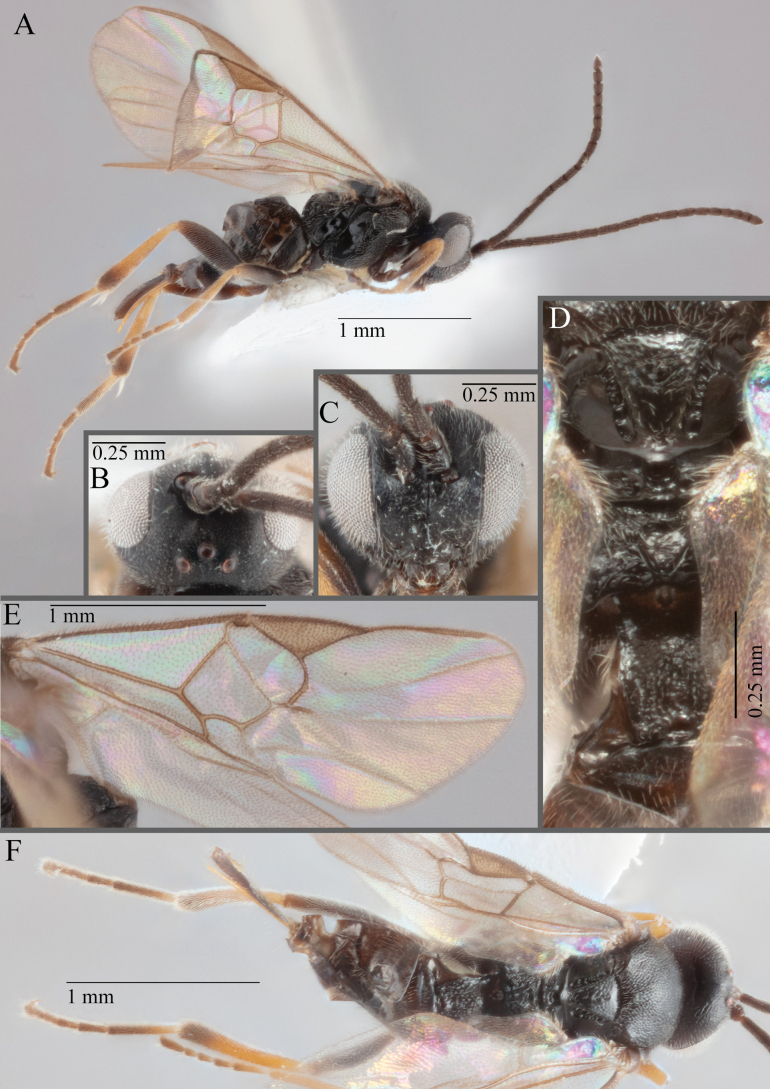
*Apantelesbanrock* (holotype AUMIC590-23) **A** lateral habitus **B** dorsal head **C** anterior head **D** dorsal propodeum and T1–2 **E** fore and hind wing **F** dorsal habitus.

Fig. 4B (distribution), Fig. 25 (holotype)

###### Type material.

***Holotype*.** Australia • ♀; SA, Banrock Site 10; -34.1891, 140.334; 21–22 Apr. 2018; R. Glatz leg.; Malaise trap; BOLD Process ID: AUMIC590-23; SAMA: 32-47750. ***Paratypes*.** Australia • ♀; SA, Banrock; -34.1891, 140.334; 20–21 Apr. 2018; R. Glatz leg.; Malaise trap; BOLD Process ID: AUMIC575-23; SAMA: 32-47751. • ♀; as previous except: BOLD Process ID: AUMIC576-23; SAMA: 32-47752. • ♀; as previous except: BOLD Process ID: AUMIC578-23; SAMA: 32-47753. • ♂; as previous except: BOLD Process ID: AUMIC577-23; SAMA: 32-47754. • ♀; as previous except: 21–22 Apr. 2018; BOLD Process ID: AUMIC655-23; SAMA: 32-47755. • ♀; as previous except: BOLD Process ID: AUMIC589-23; SAMA: 32-47756. • ♀; as previous except: BOLD Process ID: AUMIC592-23; SAMA: 32-47757. • ♀; as previous except: BOLD Process ID: AUMIC593-23; SAMA: 32-47758. • ♂; as previous except: BOLD Process ID: AUMIC591-23; SAMA: 32-47759. • ♀; as previous except: Banrock site 8; -34.1714, 140.314; BOLD Process ID: AUMIC584-23; SAMA: 32-47761. • ♂; SA, Banrock Site 10; -34.2045, 140.341; 20 Apr. 2021; R. Glatz leg.; on aquatic veg. (purple flowers and lanceolate leaves); BOLD Process ID: AUMIC603-23; SAMA: 32-47760.

###### Diagnostic description.

***Size***: Total body length: 2.5 mm; fore wing length: 2.5 mm. ***Head***: anterior scape colour similar or only very slightly paler than head colour; F2L/W ratio: 3.6; F14L/W ratio: 1.1. ***Mesosoma***: scutoscutellar sulcus with nine pits; mesoscutellar disc mostly smooth, or with very shallow scattered indentations, sculpturing stronger in anterior half; propodeal areola complete, or mostly so; propodeum mostly smooth posteriorly, mostly rugose anteriorly; coxae colour (pro, meso, meta): dark all; metafemur colour mostly dark. ***Wings***: centre of pterostigma paler (more hyaline) than outer edges; fore wing r vein length/2RS vein length ratio: 1.4. ***Metasoma***: T1 shape mostly parallel, then narrowing in distal 1/3; T1 medial length/anterior width between 1–2 × longer than wide; T1 mostly rugose; T2 mostly smooth; ovipositor sheath length/metatibia length ratio: 0.8.

*Apantelesbanrock* can be separated from most other species of *Apanteles* in Australia with a dark metacoxa and metafemur by the pterostigma with outer border darker than centre, centre of pterostigma pale or transparent/hyaline (but much less extreme than other species), the fore wing with M+CU, 1 cu-a, 1M, 1CU, (RS+M)a, 2RS and 1m-cu pigmented for most of their lengths, T1and propodeum with strong sculpture, and the propodeal areola strongly V-shaped.

###### Etymology.

This species is named for Banrock Station, the collection locality, to honour the commitment of the winery to restoring and protecting the natural environment. The epithet is a noun in apposition.

###### Distribution.

Currently known only from Banrock Station in the Riverland region of SA.

###### Molecular information.

*Apantelesbanrock* contains sequences currently in BIN BOLD:AFF1781. The COI sequences are at least 3.2% divergent from any of the other species treated here, or any available sequence on BOLD. The *wg* sequence of the holotype is ≥ 11 bp different to any other species. All delimitation methods separated *Apantelesbanrock* as a distinct species.

##### ﻿﻿ Apanteles breviflagellarius

Taxon classificationAnimaliaHymenopteraBraconidae

Slater-Baker, Fagan-Jeffries, Fernández-Triana, Portmann & Oestmann sp. nov.

171BFB02-FD3D-5208-888E-FAC39F75F611

https://zoobank.org/3619FEB1-465F-435C-AA30-90652B417A66

**Figure 26.**
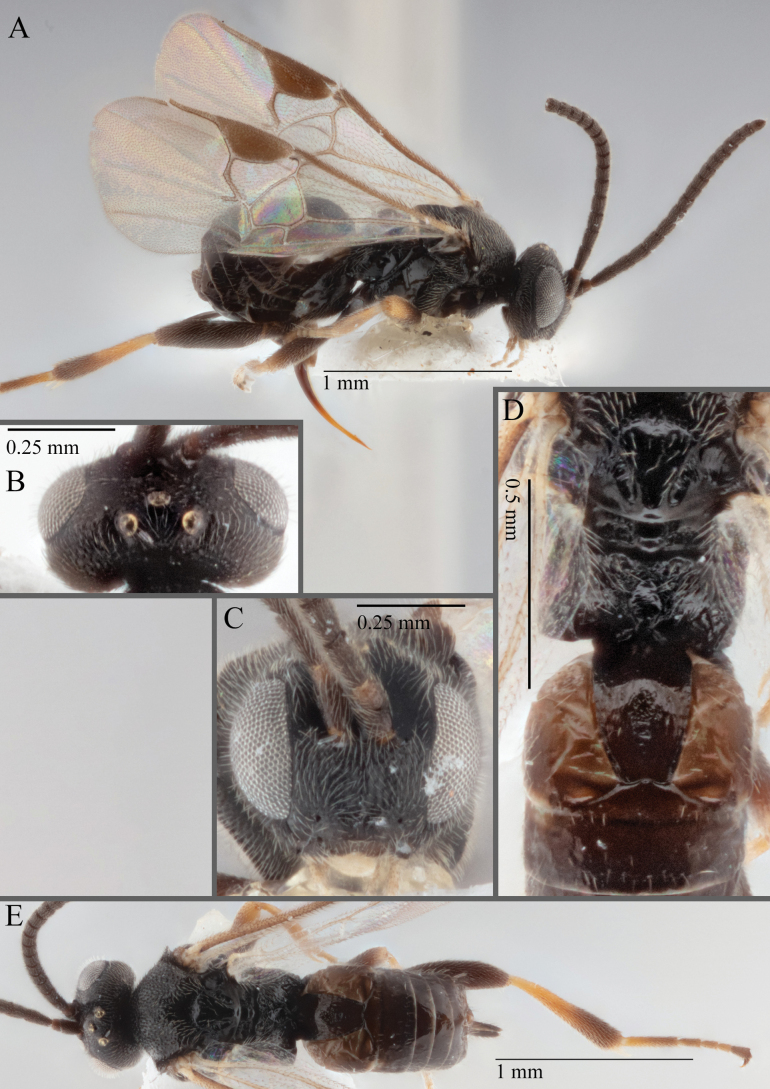
*Apantelesbreviflagellarius* (holotype AUMIC081-18) **A** lateral habitus **B** dorsal head **C** anterior head **D** dorsal propodeum and T1–T3 **E** dorsal habitus.

Fig. 4C (distribution), Fig. 26 (holotype)

###### Type material.

***Holotype*.** Australia • ♀; QLD, Samsonvale Cemetery, 8.5 km SSE Dayboro; -27.2703, 152.856; 50 m; 8 Feb.–28 Mar. 2015; S. Wright leg.; Malaise trap; Casuarina/open forest; BOLD Process ID: AUMIC081-18; QM: T208355.

###### Diagnostic description.

***Size***: Total body length: 2.1 mm; fore wing length: 2.0 mm. ***Head***: anterior scape colour similar or only very slightly paler than head colour; F2L/W ratio: 1.7; F14L/W ratio: 0.9. ***Mesosoma***: scutoscutellar sulcus with 11 pits; mesoscutellar disc mostly smooth, or with very shallow scattered indentations; propodeal areola complete, or mostly so; propodeum mostly smooth posteriorly, mostly rugose anteriorly; coxae colour (pro, meso, meta): dark all; metafemur colour mostly dark. ***Wings***: centre of pterostigma pigmented to same degree as the outer edges; fore wing r vein length/2RS vein length ratio: 1.0. ***Metasoma***: T1 shape mostly parallel, then narrowing in distal 1/3, T1 medial length/anterior width between 1–2 × longer than wide; T1 mostly smooth; T2 mostly smooth; ovipositor sheath length/metatibia length ratio: 0.4.

*Apantelesbreviflagellarius* is a slightly unusual species of *Apanteles* and quite distinct; it can be separated from other species of *Apanteles* in Australia by the dark metacoxa and metafemur, the short ovipositor sheaths and hypopygium with a ventral membranous area, the antennae shorter than the body length, pterostigma uniformly pigmented and T1 strongly narrowing posteriorly.

###### Etymology.

The species epithet is a Latin adjective formed from *brevi* meaning short, and *flagellum*, relating to the short antennae of this species.

###### Distribution.

*Apantelesbreviflagellarius* is currently only known from one specimen collected in southern QLD.

###### Molecular information.

*Apantelesbreviflagellarius* contains sequences currently in BIN BOLD:ADI5121. The COI sequences are at least 7% divergent from any of the other species treated here, or any available sequence on BOLD. The *wg* sequence of the holotype is ≥ 12 bp different to any other species. All delimitation methods separated *Apantelesbreviflagellarius* as a distinct species.

##### ﻿﻿ Apanteles brockhedgesi

Taxon classificationAnimaliaHymenopteraBraconidae

Slater-Baker, Fagan-Jeffries, Fernández-Triana, Portmann & Oestmann sp. nov.

C848786F-E4D4-59B5-B3CC-149F90FC7B46

https://zoobank.org/04DC65A2-909F-4B9B-BBB7-69AAC6E0FE56

**Figure 27.**
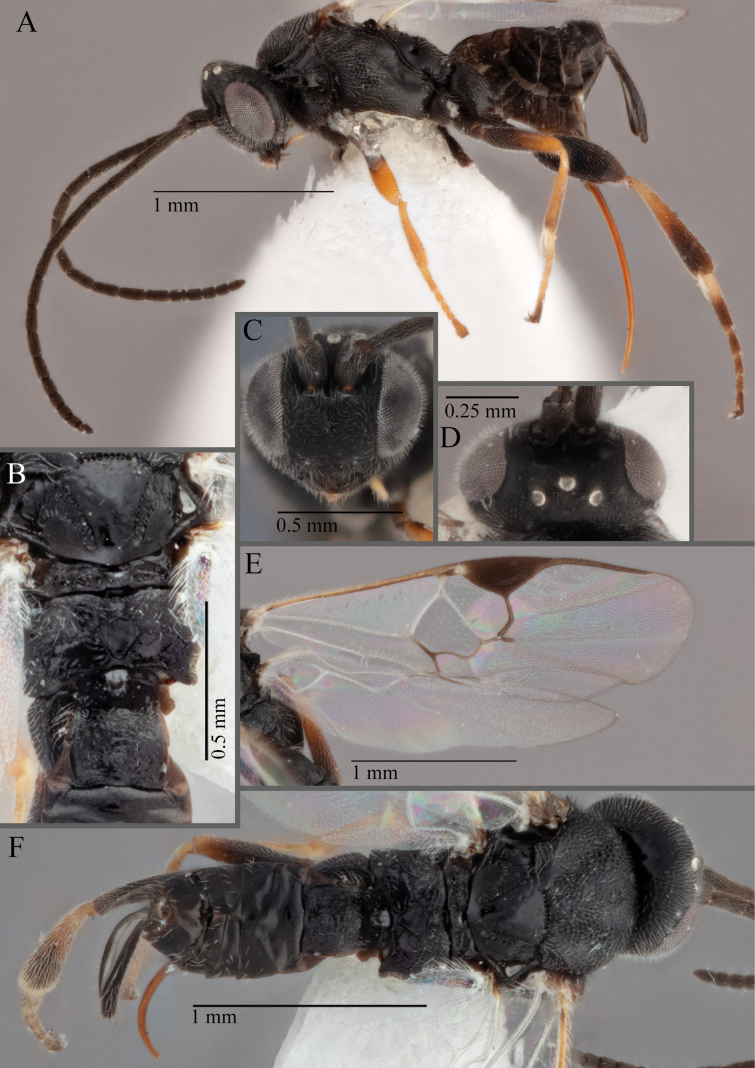
*Apantelesbrockhedgesi***A** lateral habitus (examined material AUMIC085-18) **B** dorsal propodeum and T1–2 (examined material AUMIC085-18) **C** anterior head (holotype AUMIC326-18) **D** dorsal head (examined material AUMIC085-18) **E** fore and hind wing (holotype AUMIC326-18) **F** dorsal habitus (examined material AUMIC085-18).

Fig. 4C (distribution), Fig. 27 (examined material and holotype)

###### Type material.

***Holotype*.** Australia • ♀; SA, Witchelina Stn; -30.1056, 137.738; 20 Oct. 2010; R. Kittel leg.; sweeping *Acacia* sp. in sand dune; Bush Blitz Svy RK069; BOLD Process ID: AUMIC326-18; SAMA: 32-47816. ***Paratypes*.** Australia • ♀; SA, Andamooka Arid Explorers Garden; -29.5519, 137.167; 2 May. 2021; E. Fagan-Jeffries, SA Museum Waterhouse Club members leg.; General vegetation sweep, several flowering plants; BOLD Process ID: AUMIC734-23; SAMA: 32-47817. • ♀; SA, Andamooka school road; -30.4479, 137.167; 2 May. 2021; E. Fagan-Jeffries, SA Museum Waterhouse Club members leg.; General vegetation sweep; BOLD Process ID: AUMIC740-23; SAMA: 32-47818. • ♀; SA, Bon Bon Stn; -30.3602, ?; 28 Oct. 2010; G.S. Taylor leg.; Swept *Acaciaaneura*; Bush Blitz 063 (B24); BOLD Process ID: AUMIC347-18; SAMA: 32-47819. • ♂; SA, Great Victoria Desert, Cook Road; -29.0067, 130.012; 29 Aug. 2015; J. A. Forrest & R. Leijs leg.; Vehicle net; 28.9684°S, 130.0772°E to 29.0449°S, 129.9475°E; BOLD Process ID: AUMIC1265-24; SAMA: 32-47767. • ♂; SA, Great Victoria Desert, Cook Road; -29.0067, 130.012; 29 Aug. 2015; J. A. Forrest & R. Leijs leg.; Vehicle net; 28.9684°S, 130.0772°E to 29.0449°S, 129.9475°E; BOLD Process ID: AUMIC1262-24; SAMA: 32-47768. • ♂; SA, Hiltaba; -32.1033, 135.092; 234 m; 26 Sep. 2021; E. Fagan-Jeffries leg.; sweeping vegetation; flowering yellow *Eucalyptus*; BOLD Process ID: AUMIC928-23; SAMA: 32-47820. • ♀; SA, Walker flat, Mallee Rd; -34.8086, 139.494; 2 May. 2021; BA Parslow leg.; sweeping vegetation; Eucalyptus flowers; BOLD Process ID: AUMIC865-23; SAMA: 32-47769. • ♀; SA, Witchelina Stn; -30.0186, 137.901; 23 Oct. 2010; R. Kittel leg.; sweeping *Acaciavictoriae*; Bush Blitz Svy RK091; BOLD Process ID: AUMIC352-18; SAMA: 32-47821. • ♀; as previous except: BOLD Process ID: AUMIC353-18; SAMA: 32-47822. • ♀; as previous except: BOLD Process ID: AUMIC354-18; SAMA: 32-47823. • ♀; WA, Western Pilbara, Nanutarra-Wittenoom Rd., 1.7 km north of Hamersley Rd. turnoff.; -22.2317, 117.983; 29 May. 2004; D.R. Britton & A. Donnelly leg.; light trap; BOLD Process ID: AUMIC1295-24; AM: K.247578.

###### Examined material.

Australia • ♀; SA, Bon Bon Stn; -30.3139, 135.448; 28 Oct. 2010; R. Kittel leg.; sweep netting on *Acaciavictoriae*; Bush Blitz Svy RK129; BOLD Process ID: AUMIC085-18; SAMA: 32-47815. [This specimen was not included with the paratype material as it was badly damaged during imaging, but the morphology is consistent with the holotype and the COI sequence is only 2 bp different to that of the holotype (*wg* sequence is identical).]

###### Diagnostic description.

***Size***: Total body length: 2.8 mm; fore wing length: 2.6 mm. ***Head***: anterior scape colour similar or only very slightly paler than head colour; F2L/W ratio: 2.7; F14L/W ratio: 1.1. ***Mesosoma***: scutoscutellar sulcus with 12 pits; mesoscutellar disc mostly smooth with very shallow scattered indentations; propodeal areola complete, or mostly so; propodeum mostly smooth posteriorly, mostly rugose anteriorly; coxae colour (pro, meso, meta): dark all; metafemur colour mostly dark. ***Wings***: centre of pterostigma pigmented to same degree as the outer edges with a pale spot proximally; fore wing r vein length/2RS vein length ratio: 1.2. ***Metasoma***: T1 shape mostly parallel, T1 medial length/anterior width between 1–2 × longer than wide; T1 mostly rugose; T2 mostly smooth; ovipositor sheath length/metatibia length ratio: 0.8.

*Apantelesbrockhedgesi* can be separated from most other species of *Apanteles* in Australia that have a dark metacoxa and metafemur and the pterostigma without a pale centre, the ovipositor sheath length > 0.6 × metatibia length and the antenna of similar size to the body length by the pterostigma having a small pale spot proximally, the metatibia displaying a gradient of colouration from pale to dark, the colours merging in the centre, T1 with strong sculpture over at least most of posterior 1/2 of tergite, mesoscutellar disc with at most scattered punctures along margins, fore wing vein 1M much less pigmented (often transparent/pale) compared to pigmentation of vein 1CUa, and the scutoscutellar sulcus comparatively narrower and with comparatively smaller pits than most species.

###### Etymology.

This species is named after Brock Hedges, who facilitated the field trip which resulted in the collection of the specimen at Hiltaba Nature Reserve. Dr Hedges has provided considerable support to several authors of this paper for many years.

###### Distribution.

*Apantelesbrockhedgesi* is a commonly collected species with a broad distribution across SA, with a single collection record in WA.

###### Molecular information.

*Apantelesbrockhedgesi* contains sequences currently in BIN BOLD:ADI3291. The COI sequences are at least 8% divergent from any of the other species treated here, or any available sequence on BOLD. The *wg* sequence of the holotype is ≥ 14 bp different to any other species. All delimitation methods separated *Apantelesbrockhedgesi* as a distinct species.

##### ﻿﻿ Apanteles carpatus

Taxon classificationAnimaliaHymenopteraBraconidae

(Say, 1836)

7700C989-C67B-5D2F-B7D2-BC3B2CB748A5

**Figure 28.**
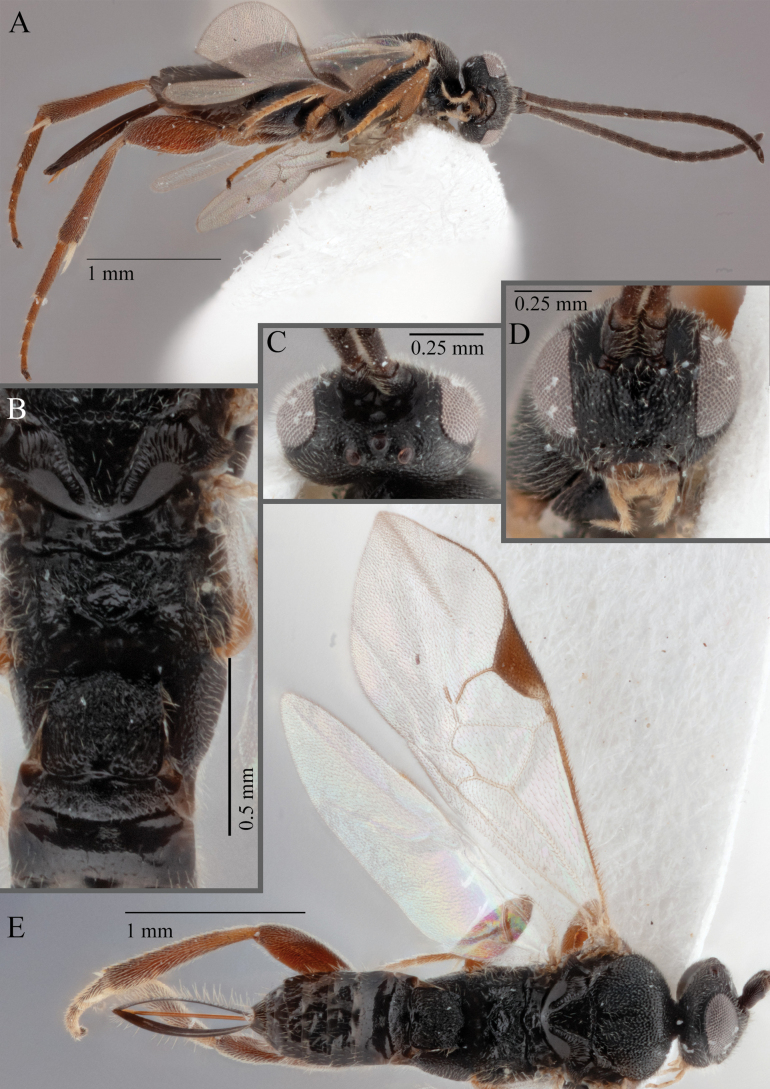
*Apantelescarpatus* (Australia – AUMIC864-23) **A** lateral habitus **B** dorsal propodeum and T1–3 **C** dorsal head **D** anterior head **E** dorsal habitus and wings.

**Figure 29.**
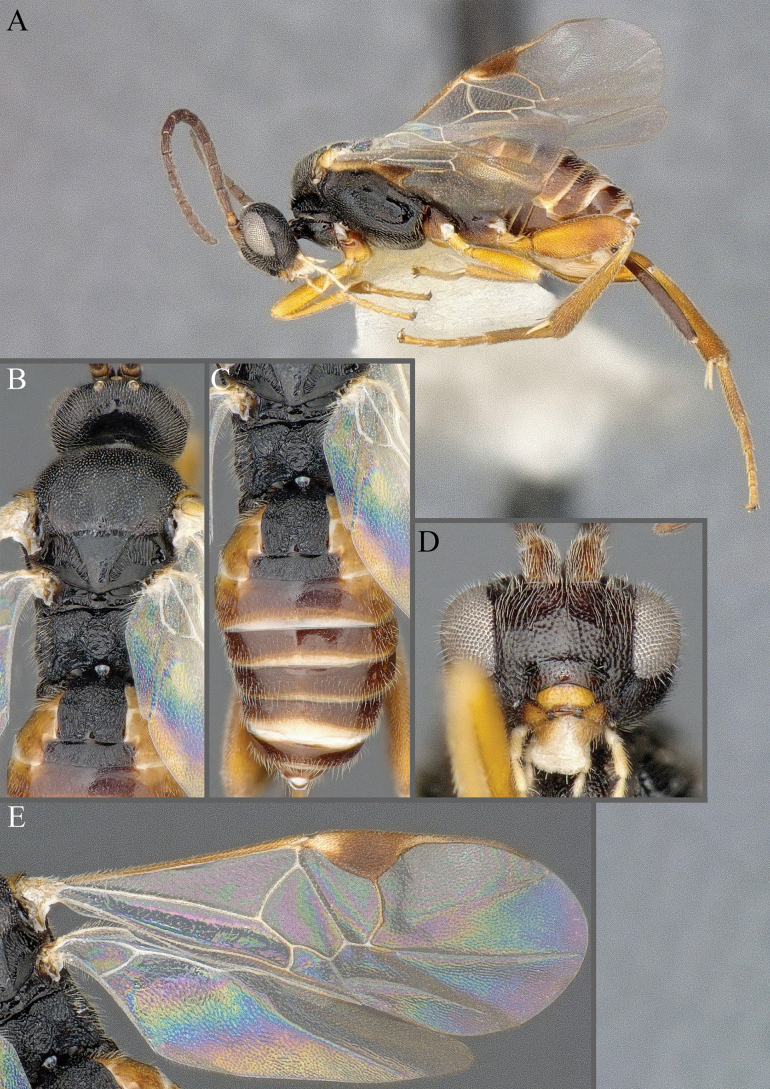
*Apantelescarpatus* (Canada – MIC000036) **A** lateral habitus **B** dorsal mesosome **C** dorsal metasoma **D** anterior head **E** wings.

Fig. 5A (distribution in Australia/New Zealand from this study), Fig. 28 (Australian specimen), Fig. 29 (Canadian specimen)

###### Holotype information.

♀; USA (lost).

###### Examined material.

Australia • ♀; SA, Aberfoyle Park, inside private residence; -35.0641, 138.607; 11 May. 2021; BA Parslow leg.; At light indoors; BOLD Process ID: AUMIC864-23; SAMA: 32-47772. • ♀; SA, Adelaide; -34.941, 138.643; 14 Nov. 2021; A. Bird leg.; hand caught; BOLD Process ID: OZBOL6285-22; SAMA: 32-47773. • ? (missing abdomen); SA, Cox Scrub Con. Pk.; -35.3319, 138.746; 03–17 Apr. 2016; E. Fagan-Jeffries leg.; Malaise trap; BOLD Process ID: AUMIC062-18; SAMA: 32-47774. • ♀; SA, The University of Adelaide, Darling Building; -34.919, 138.603; 25 May. 2021; B. Hedges leg.; hand caught; BOLD Process ID: AUMIC877-23; SAMA: 32-47775.

The material above matched identified DNA barcodes of *A.carpatus* on BOLD. Images of an identified specimen from the CNC (MIC000036, from Canada) were also examined.

###### Diagnosis.

*Apantelescarpatus* can be separated from all described species of *Apanteles* in Australia with the metacoxa dark by having the pterostigma with large pale spot in proximal corner, fore wing veins 1CUa, 1CUb, 1m-cu all pale/unpigmented and T1 and T2 strongly sculptured.

###### Notes.

The Australian specimens listed above have identical COI barcodes to verified specimens from Canada and New Zealand, their morphology aligns well with descriptions and available images of previously identified specimens (e.g., Fig. 29, *A.carpatus* from Canada, held in the CNC). Additionally, the collection locality of three specimens being inside buildings makes sense given the known hosts of *A.carpatus* are clothes moths (Tineidae). We feel this is a reasonably certain identification of these specimens, and we provide the first sequences (to our knowledge) of the species from Australia. We note the colouration of Australian specimens, particularly on the legs, is much darker than the specimen imaged from Canada.

##### ﻿﻿ Apanteles cuprum

Taxon classificationAnimaliaHymenopteraBraconidae

Slater-Baker, Fagan-Jeffries, Fernández-Triana, Portmann & Oestmann sp. nov.

1DB26814-7A9D-5F0B-A064-0936C6BC0583

https://zoobank.org/BE19CFB1-7FA2-44B8-95F5-82A3C055F632

**Figure 30.**
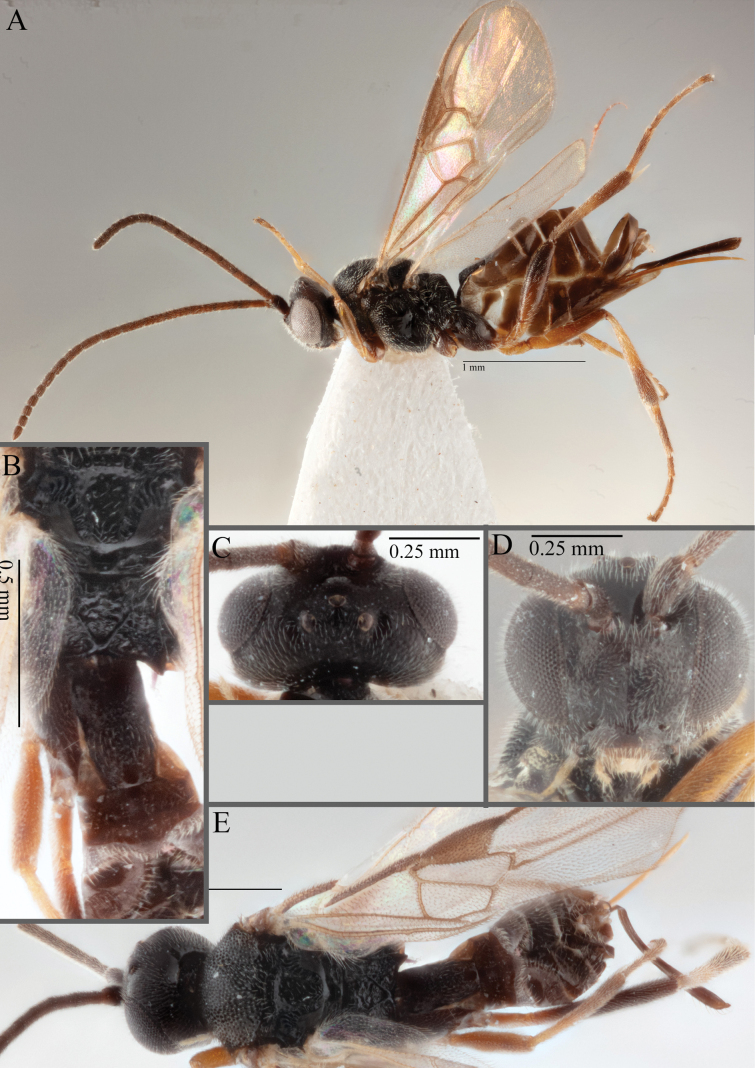
*Apantelescuprum***A** lateral habitus (paratype ASMII3877-22) **B** dorsal mesoscutellar disc, propodeum and T1–3 (holotype AUMIC1395-24) **C** dorsal head (holotype AUMIC1395-24) **D** anterior head (holotype AUMIC1395-24) **E** dorsal habitus (holotype AUMIC1395-24).

Fig. 5A (distribution), Fig. 30 (holotype)

###### Type material.

***Holotype*.** Australia • ♀; NT, Keep River NP, Hazard Creek, 23.7 km SSW Jarrnarm Camp Ground; -15.9592, 129.017; 3–8 Jun. 2001; M. E. Irwin, F. D. Parker & C. Lambkin leg.; Malaise trap; BOLD Process ID: AUMIC1395-24; ANIC: 32-085574. ***Paratypes*.** Australia • ♀; QLD, Gununa, Wellesley Islands; -16.666, 139.182; 1–8 Mar. 2022; Mornington Island State School P-10 students leg.; Malaise trap; Insect Investigators; BOLD Process ID: ASMII3877-22; QM: T261165. Australia • ♂; QLD, Mount Molloy; -16.674, 145.336; 8–15 Mar. 2022; Mount Molloy State School students leg.; Malaise trap; Insect Investigators; BOLD Process ID: ASMII12307-22; QM: T261166. • ♀; WA, Kununurra; -15.769, 128.737; 15–22 Mar. 2022; East Kimberley Kununurra students leg.; Malaise trap; Insect Investigators; BOLD Process ID: ASMII9771-22; WAM: 130551. FIJI • ♀; Viti Levu, Sigatoka Province, Sigatoka Sand Dunes Nat. Park; -18.1678, 177.506; 31 m; 28 Nov.–3 Dec. 2003; Irwin & Schlinger leg.; Malaise Trap; BOLD Process ID: CNCHW953-09; CNC: CNCH2363.

###### Examined material.

The following specimen agrees in general morphology to the type series, and has a matching COI barcode; however, it is significantly smaller in size; we leave this specimen out of the type material in case it represents a contaminated sequence.

Australia • ♂; QLD, Laura; -15.5811, 144.458; 15 Mar. 2017; R. Leijs leg.; vehicle netting from Laura caravan park to Quinkan Bush Blitz Site SSS2 (Welcome Rd); BOLD Process ID: AUMIC972-24; QM: T261164.

###### Diagnostic description.

***Size***: Total body length: 2.5 mm; fore wing length: 2.5 mm. ***Head***: anterior scape colour similar or only very slightly paler than head colour; F2L/W ratio: 3.8; F14L/W ratio: 1.3. ***Mesosoma***: scutoscutellar sulcus with seven pits; mesoscutellar disc punctate throughout; propodeal areola complete, or mostly so; propodeum mostly rugose; coxae colour (pro, meso, meta): dark all; metafemur colour mostly dark. ***Wings***: centre of pterostigma pigmented to same degree as the outer edges; fore wing r vein length/2RS vein length ratio: 1.4. ***Metasoma***: T1 shape mostly parallel but narrowing slightly in posterior 1/3 or 1/4, T1 medial length/anterior width between 1–2 × longer than wide; T1 mostly rugose; T2 with fine sculpture; ovipositor sheath length/metatibia length ratio: 1.0.

*Apantelescuprum* can be separated from the other species of *Apanteles* known from Australia with a dark hind coxa by the metafemur with pale area in the proximal 1/4, the trochanter and trochantellus also pale, the anterior side of the scape similar in colour to the head (not much paler), T2 smooth, and T1 narrowing posteriorly as in Fig. 30B.

Due to this species also occurring in Fiji (identified through a COI sequence from Fiji in the same BIN, with the specimen (CNC CNCHW953-09) examined and morphology aligning to that of the Australian material) we also diagnose this species against those known from Fiji. Diagnoses are based on the information in Nixon (1965) unless otherwise noted. *Apantelescuprum* can be separated from:

*Apantelesaglaus* Nixon, 1965 and *Apantelesdaimenes* Nixon, 1965 by the ovipositor sheath approximately the same length as the metatibia (*A.aglaus* and *A.daimenes* described as having the ovipositor sheath only 2/3 as long as the metatibia).

*Apanteleseurynome* Nixon, 1965 by the fore wing membrane hyaline (no infuscation) and the antennae at least as long as the body length (*A.eurynome* is described as having a “faint proximal cloud of the fore wing [that] hardly extends into the median cell” and “the antenna is short with the three preapical segments slightly transverse.”

*Apanteleshymeniae* Wilkinson, 1935 by having T2 smooth and the hind leg mostly brown (although pale on the proximal 1/2 of the metatibia and the trochanter), whilst *A.hymeniae* is described by Nixon (1965) as “characterised essentially by the weakly transverse, heavily rugose median field of tergite (2 + 3) and the bright yellow legs.”

*Apantelesorphne* Nixon, 1965 by the fore wing membrane hyaline and the ovipositor sheath approximately the same length as the metatibia; *A.orphne* is described as having a “proximal cloud of the fore wing [which fills] only about distal sixth of the median cell” and the ovipositor sheaths 1.5 × as long as the metatibia.

*Apantelessamoanus* Fullaway, 1940 by the T1 being close to 2 × longer than wide (if anterior width is measured) or > 2 × longer than wide (if posterior width is measured), and narrowing in posterior 1/3 or 1/4; whilst *A.samoanus* is described as the “1^st^ tergite a little longer than wide with parallel sides” (from original description, not treated by Nixon (1965)).

*Apantelestirathabae* Wilkinson, 1928 by the antennae similar length to the body length (*A.tirathabae* has the antenna short, thick, with segments 16 and 17 not longer than wide).

*Apantelestrifasciatus* Muesebeck, 1946 by having the fore wing membrane hyaline (*A.trifasciatus* has very strong infuscated areas, images of type compared: http://n2t.net/ark:/65665/3ff1ddf70-f69e-4d29-97f1-7c4dffd71683).

###### Etymology.

The species epithet is a Latin noun in apposition (genitive case) and was named by students at Mount Molloy State School, who collected two paratype specimens during ‘Insect Investigators’. The Latin noun means ‘copper’ (the metal) and the students chose the name to relate to the rich copper resources found in the Mount Molloy region. Whilst the species is found in many places without copper mining, we think this name is also apt because of the more coppery colour of this species compared to many others in the genus.

###### Distribution.

*Apantelescuprum* is found in the north of Australia (northern NT, QLD, WA) and also in Fiji.

###### Molecular information.

*Apantelescuprum* is currently represented by sequences in BIN BOLD:AAM7397. The COI sequences are at least 6% divergent from any of the other species treated here, or any available sequence on BOLD. There is only a *wg* sequence available for the specimen from Laura (AUMIC972-24), but all COI delimitation methods resolved *A.cuprum* as a discrete species.

##### ﻿﻿ Apanteles darthvaderi

Taxon classificationAnimaliaHymenopteraBraconidae

Slater-Baker, Fagan-Jeffries, Fernández-Triana, Portmann & Oestmann sp. nov.

129ACB05-7431-5BDF-9F49-B73328B76230

https://zoobank.org/AB7AC3CB-5E68-4A00-942D-59ED06CC5682

**Figure 31.**
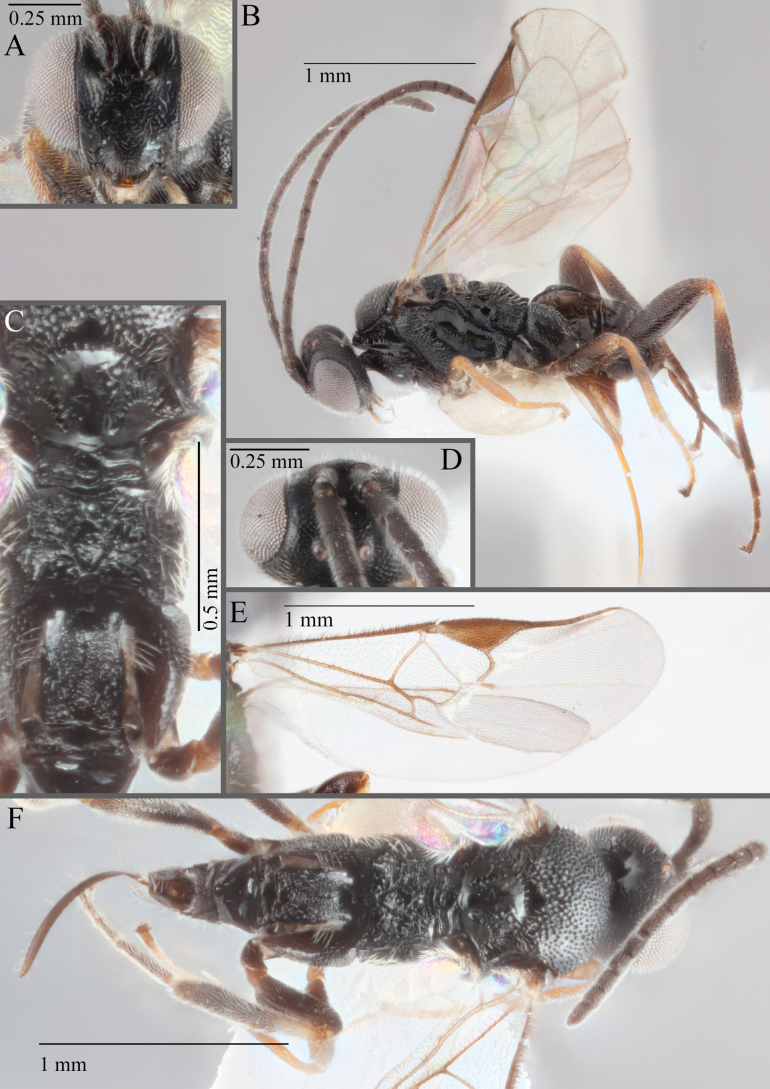
*Apantelesdarthvaderi* (holotype AUMIC637-23) **A** anterior head **B** lateral habitus **C** dorsal propodeum and T1-3 **D** dorsal head **E** fore and hind wing **F** dorsal habitus.

Fig. 5B (distribution), Fig. 31 (holotype)

###### Type material.

***Holotype*.** Australia • ♀; QLD, Prospect Creek State School; -24.4218, 150.43; 6 Oct.–4 Nov. 2020; E. Fagan-Jeffries & Prospect Creek State School leg.; Malaise trap; BOLD Process ID: AUMIC637-23; QM: T261222. ***Paratypes*.** Australia • ♀; ACT, Canberra, Cook, 8 Moss Street; -35.261, 149.059; 10 Apr. 2011; P.Hebert, R. Labbee, V.Levesque-Beaudin, J.McCormick, J.Sones, J.Webb leg.; BOLD Process ID: AACTA4853-20; ANIC: 32-085567. • ♀; as previous except 02 Apr. 2011; BOLD Process ID: AACTA5193-20; ANIC: 32-085568. • ♀; NSW, Bendemeer; -30.819, 151.142; 19 Dec. 2020–04 Jan. 2021; A. Goodwin, R. Noakes leg.; Malaise trap; BOLD Process ID: AUMIC626-23; AM: K.647416. • ♀; NSW, Bendemeer; -30.819, 151.142; 19 Dec. 2020–04 Jan. 2021; A. Goodwin, R. Noakes leg.; Malaise trap; BOLD Process ID: AUMIC627-23; AM: K.647417. ♀; as previous; BOLD Process ID: AUMIC629-23; AM: K.647418. • ♂; as previous; BOLD Process ID: AUMIC946-24; AM: K.647419. • ♂; NSW, Hat Head; -31.063, 153.052; 20–27 Dec. 2012; Paul Hebert leg.; Malaise trap; BOLD Process ID: AUSMA270-14; QM: T261271. • ♂; as previous; BOLD Process ID: AUSMA277-14; QM: T261275. • ♂; as previous except 10–17 Dec. 2010; BOLD Process ID: HYAS463-11; QM: T261276. • ♂; as previous except 22 Feb. 2019; BOLD Process ID: NSWHP4438-19; QM: T261277. • ♀; NSW, Oxley Wild Rivers National Park, East Kunderang Track; -30.8181, 152.136; 07 Nov. 2015; D.M. Bray leg.; pan trap - blue; BOLD Process ID: AUMIC1249-24; AM: K.379878. • ♀; as previous; BOLD Process ID: AUMIC1250-24; AM: K.646439. • ♀; NSW, Wonboyn; -37.2447, 149.903; 26 Jan.–10 Feb. 2020; P. Whitington, K-L. Harris leg.; Malaise trap; BOLD Process ID: AUMIC900-23; ANIC: 32-085561. • ♀; QLD, Back Plains; -27.892, 151.783; 1–8 Mar. 2022; Back Plains State School students leg.; Malaise trap; Insect Investigators; BOLD Process ID: ASMII13121-22; QM: T261181. • ♂; QLD, Cockburn Rvr Camp; -31.0522, 151.144; 10 Dec. 2019; J. B. Dorey leg.; General sweep over *Brachychiton* flowering species in sclerophyll forest along dry creekbed; Sunny and warm ~ 33C; BOLD Process ID: AUMIC1139-24; ANIC: 32-085562. ♂; as previous; BOLD Process ID: AUMIC1140-24; ANIC: 32-085563. • ♂; as previous; BOLD Process ID: AUMIC1141-24; QM: T261223. • ♂; as previous; BOLD Process ID: AUMIC1142-24; QM: T261224. • ♂; as previous; BOLD Process ID: AUMIC1180-24; QM: T261225. • ♂; as previous; BOLD Process ID: AUMIC1181-24; QM: T261226. • ♂; QLD, Crediton; -21.2079, 148.521; 23 Nov. 2019; JB Dorey leg.; General sweep over white flowering *Acacia* in farmland with big gums around.; Sunny and warm.; BOLD Process ID: AUMIC1226-24; QM: T261227. • ♀; QLD, Prospect; -24.42, 150.43; 22 Mar.–1 Apr. 2022; Prospect Creek State School students leg.; Malaise trap; Insect Investigators; BOLD Process ID: ASMII7957-22; QM: T261182. • ♀; as previous except 15–22 Mar. 2022; BOLD Process ID: ASMII7910-22; QM: T261183. • ♀; as previous except 6 Oct.–4 Nov. 2020; BOLD Process ID: AUMIC664-23; QM: T261228. • ♂; as previous; BOLD Process ID: AUMIC674-23; QM: T261229. • ♂; as previous; BOLD Process ID: AUMIC662-23; QM: T261230. • ♀; as previous except 12 Aug.–15 Sep. 2020; BOLD Process ID: OZBOL418-21; AM: K.647420. • ♀; QLD, Townsville, Hervey Range; -19.3812, 146.449; 380 m; 23 Jun. 2017; Graeme Cocks leg.; netted; BOLD Process ID: GCQT1802-17; QM: T261272.

###### Diagnostic description.

***Size***: Total body length: 2.2 mm; fore wing length: 2.2 mm. ***Head***: Anterior scape colour similar or only very slightly paler than head colour; F2L/W ratio: 3.1; F14L/W ratio: 1.2. ***Mesosoma***: Scutoscutellar sulcus with 12 pits; mesoscutellar disc mostly smooth, or with very shallow scattered indentations; propodeal areola complete, or mostly so; propodeum mostly rugose; coxae colour (pro, meso, meta): dark all; metafemur completely dark; outer side of metatibia mostly dark with pale area in proximal 1/3. ***Wing***: Fore wing membrane completely hyaline/transparent, without any trace of infuscation in the membrane. Centre of pterostigma pigmented to same degree as the outer edges; no large pale spot present on pterostigma; fore wing r vein length/2RS vein length ratio: 0.8. ***Metasoma***: T1 shape mostly parallel, T1 medial length/anterior width between 1–2 × longer than wide; T1 mostly rugose; T2 mostly smooth; T3–6 with setae reduced to a single row on each tergite (exceptionally there may be a few extra setae laterally on some tergites but most setae are in a single row on each tergite); ovipositor sheath length/metatibia length ratio: 1.0.

*Apantelesdarthvaderi* can be diagnosed from the other species with a dark metacoxa and metafemur, the outer side of metatibia mostly dark with a paler area in the proximal 1/3, an ovipositor > 0.6 × metatibia length, the pterostigma uniformly pigmented (no hyaline centre or large pale spot), and T1 with rugose sculpturing, by the fore wing membrane completely hyaline (no infuscation) and T3–T6 with setae mostly reduced to a single row on each tergite.

###### Etymology.

The species was named by students from Back Plains State School. The students named the species for a fictional character from the Star Wars space opera franchise, “because this mean little wasp is from the dark side like Darth Vader, because it sucks the life out of the caterpillars.” The species epithet is a noun in the genitive case.

###### Distribution.

*Apantelesdarthvaderi* is currently known from along the east coast of Australia, from as far north as Townsville, as far south as Wonboyn, and as far inland as Canberra.

###### Molecular information.

*Apantelesdarthvaderi* contains a single BIN (BOLD:AAU8286). The COI sequences are at least 8% divergent from any of the other species treated here, or any available sequence on BOLD. The species contains a single *wg* haplotype which is ≥ 3 bp different to any other species. We consider *A.darthvaderi* a well-supported species, with 5/7 congruent molecular delimitation methods. PTP and ASAP using *wg* grouped the species with *A.auroralis*, but as there are clear morphological differences (*A.darthvaderi* has the metafemur dark, *A.auroralis* has the metafemur completely pale), we believe these methods were too conservative in this case.

###### Remarks.

*Apantelesdarthvaderi* closely resembles *A.persephone* and runs to the same couplet in the key if *A.persephone* is treated as having the metatibia mostly dark. We do not have a strong morphological character to separate these species, but also hesitate to identify the specimens here as *A.persephone*. *Apantelespersephone* was described from two specimens collected from near Perth in Western Australia, whilst *A.darthvaderi* is only currently known from the east coast. We have decided to describe *A.darthvaderi* as new based on this geographic disparity, but note that DNA sequencing of the type material of *A.persephone*, or at minimum sequencing a matching specimen from near the type locality, would resolve these two species more confidently.

##### ﻿﻿ Apanteles doreenwatlerae

Taxon classificationAnimaliaHymenopteraBraconidae

Slater-Baker, Fagan-Jeffries, Fernández-Triana, Portmann & Oestmann sp. nov.

0897724C-BBFE-547A-9A2D-55ABFC283A65

https://zoobank.org/93DA20A7-D28B-447E-885C-C5DF174E9A7E

**Figure 32.**
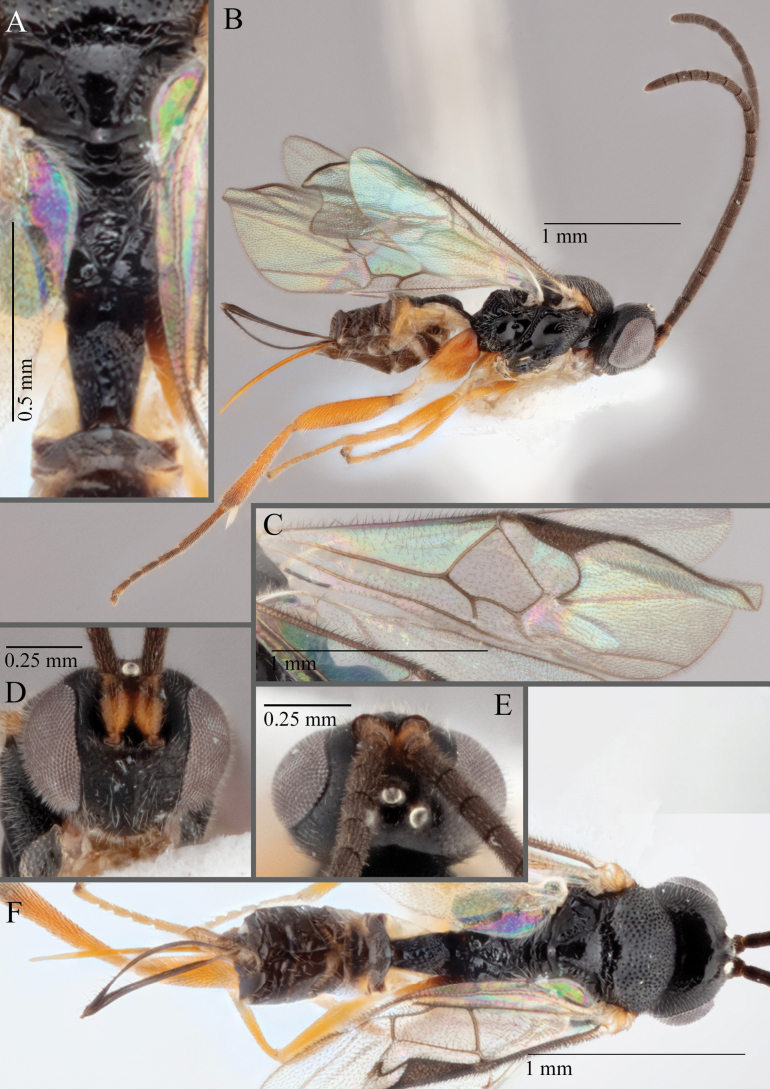
*Apantelesdoreenwatlerae* (holotype OZBOL431-21) **A** dorsal propodeum, T1, and T2**B** lateral habitus **C** fore wing **D** anterior head **E** dorsal head **F** dorsal habitus.

Fig. 5C (distribution), Fig. 32 (holotype)

###### Type material.

***Holotype*.** Australia • ♀; QLD, Kuranda; -16.814, 145.643; 395 m; 26 Jul.–26 Aug. 2020; M.S. Moulds leg.; Malaise trap; BOLD Process ID: OZBOL431-21; QM: T261203. ***Paratypes*.** Australia • ♀; QLD, Kuranda; -16.8135, 145.643; 317 m; 12 Feb.–6 Apr. 2020; M. S. Moulds leg.; Malaise trap; BOLD Process ID: AUMIC1491-24; QM: T261204. • ♂; as previous except: BOLD Process ID: AUMIC1506-24; QM: T261206. • ♂; QLD, Kuranda; -17.0564, 145.786; 19 May–8 Aug. 2017; M. S. Moulds leg.; Malaise trap; BOLD Process ID: AUMIC1294-24; QM: T261205.

###### Diagnostic description.

***Size***: Total body length: 2.6 mm; fore wing length: 2.4 mm. ***Head***: anterior scape colour much paler, dramatically different colour than head; F2L/W ratio: 2.9; F14L/W ratio: 1.4. ***Mesosoma***: scutoscutellar sulcus with seven pits; mesoscutellar disc mostly smooth, or with very shallow scattered indentations; propodeal areola complete, or mostly so; propodeum mostly smooth; coxae colour (pro, meso, meta): pale all; metafemur colour mostly pale. ***Wings***: centre of pterostigma pigmented to same degree as the outer edges; fore wing r vein length/2RS vein length ratio: 2.1. ***Metasoma***: T1 shape narrowing distally, T1 medial length/anterior width between 1–2 × longer than wide; T1 mostly rugose; T2 mostly smooth; ovipositor sheath length/metatibia length ratio: 1.0.

*Apantelesdoreenwatlerae* can be separated from all other described *Apanteles* species in Australia other than *A.pharusalis* by the pale metacoxa. It can be diagnosed against *A.pharusalis* by the T1 anterior width/posterior width ratio (~ 1.7 in *A.doreenwatlerae* and ~ 1.2 in *A.pharusalis*).

###### Etymology.

This species is named for Doreen Watler, for her contribution to hosting numerous visiting students and researchers (including EPFJ) at the CNC; her hospitality enables a considerable number of the international entomology exchanges in Ottawa.

###### Distribution.

*Apantelesdoreenwatlerae* is currently only known from northern QLD.

###### Molecular information.

*Apantelesdoreenwatlerae* contains sequences currently in BIN BOLD:AAM7396. The COI sequences are at least 9% divergent from any of the other species treated here, or any available sequence on BOLD. The *wg* sequence of the holotype is ≥ 7 bp different to any other species. All delimitation methods separated *Apantelesdoreenwatlerae* as a distinct species.

##### ﻿﻿ Apanteles ethanbeaveri

Taxon classificationAnimaliaHymenopteraBraconidae

Slater-Baker, Fagan-Jeffries, Fernández-Triana, Portmann & Oestmann sp. nov.

8852936B-D588-5229-B438-EF183C503946

https://zoobank.org/7C70625F-B5AF-40C4-9B70-61CF7C6DC1D2

**Figure 33.**
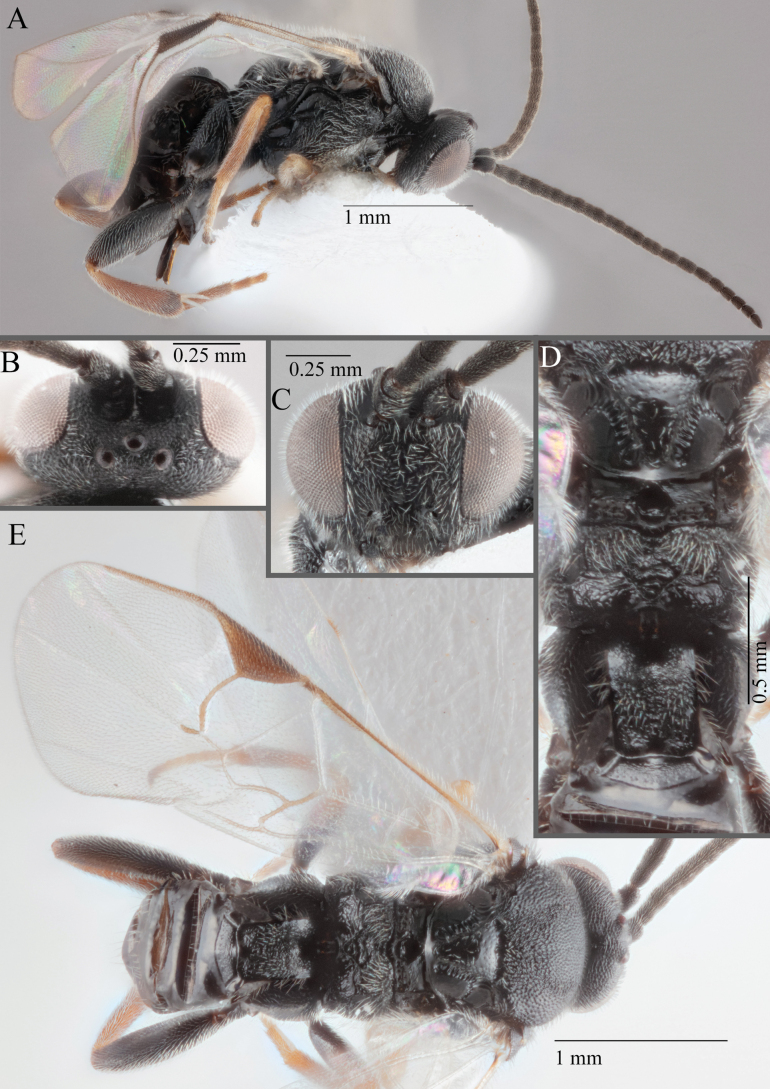
*Apantelesethanbeaveri* (holotype AUMIC730-23) **A** lateral habitus **B** dorsal head **C** anterior head **D** propodeum and T1–3 **E** lateral habitus and wings.

Fig. 4C (distribution), Fig. 33 (holotype)

###### Type material.

***Holotype*.** Australia • ♀; QLD, Specimen Hill, Herberton; -17.3823, 145.372; 08 Mar. 2021; E.P. Beaver, M.F. Braby leg.; Reared from Instar IV larva of *Jalmenuspseudictinus* (Lepidoptera: Lycaenidae) collected 04 March 2021 on *Acaciaflavescens*; wasp pupa next to dead larva. Adult wasp eclosed 08 March 2021; BOLD Process ID: AUMIC730-23; QM: T261210. ***Paratypes*.** Australia • ♀; ACT, Lyneham Ridge; -35.2386, 149.116; 11 Feb. 2017; M. F. Braby leg.; Reared from larva of *Jalmenusictinus* (Lep: Lycaenidae); BOLD Process ID: AUMIC492-18; QM: T261211. • ♂; NSW, Pilliga East SCA; -30.7918, 149.489; 22 Jun. 2021; E.P. Beaver, M.F. Braby leg.; Reared from larva *Ogyrislanthis* (Lep: Lycaenidae); BOLD Process ID: AUMIC731-23; QM: T261212.

###### Diagnostic description.

***Size***: Total body length: 3.6 mm; fore wing length: 2.9 mm. ***Head***: anterior scape colour similar or only very slightly paler than head colour; F2L/W ratio: 2.1; F14L/W ratio: 1.2. ***Mesosoma***: scutoscutellar sulcus with ten pits; mesoscutellar disc with punctures in outer regions, centre smooth; propodeal areola complete, or mostly so; propodeum mostly smooth posteriorly, mostly rugose anteriorly; coxae colour (pro, meso, meta): dark all; metafemur colour mostly dark. ***Wings***: centre of pterostigma pigmented to same degree as the outer edges; fore wing r vein length/2RS vein length ratio: 2.1. ***Metasoma***: T1 shape mostly parallel, T1 medial length/anterior width between 1–2 × longer than wide; T1 mostly rugose; T2 with fine sculpture; ovipositor sheath length/metatibia length ratio: 0.3.

*Apantelesethanbeaveri* can be separated from most other species of *Apanteles* in Australia that have a dark metacoxa and metafemur, the pterostigma without a pale centre, and the ovipositor sheath length < 0.5 × the metatibia length, by T1 parallel sided; the median length of propodeum 1.2 × the maximum width of areola. *Apantelesethanbeaveri* can be separated from *A.ligdus* by the scutoscutellar sulcus wider and with much larger pits, T2 comparatively less transverse, and T2 with posterior margin clearly curved.

###### Etymology.

This species is named for lepidopterist Ethan Beaver who reared two of the specimens and passed them on to EPFJ. Parasitoid wasp researchers owe much to lepidopterists who keep their parasitoid specimens and generously share their host data.

###### Distribution.

*Apantelesethanbeaveri* is known from three collection localities in eastern Australia, each from a different but closely related host species.

###### Molecular information.

*Apantelesethanbeaveri* contains sequences currently in BIN BOLD:ADL4962. The COI sequences are at least 2.4% divergent from any of the other species treated here, or any available sequence on BOLD. The *wg* sequence of the holotype is ≥ 1 bp different to *A.ligdus*, and ≥ 11 bp different to any other species. The molecular delimitation of this species relative to *A.ligdus* is poorly resolved: BINs, a 2% threshold, and the *wg* haplotypes split the two species, COIASAP and *wg*ASAP and PTP grouped the two species, whilst COIPTP split the two species and also split the ACT paratype (AUMIC492-18) from the other two specimens.

###### Remarks.

The collectors who reared the type series are professional lepidopterists, and therefore the host records of this species should be treated with reasonable confidence. The species is closely related to *A.ligdus* and may potentially be conspecific. We feel the available evidence at present supports them being distinct species (multiple subtle morphological differences, 2.7% COI divergence, *wg* barcodes 1 bp different, non-overlapping host species) but a larger sample size and further study may change this species hypothesis.

##### ﻿﻿ Apanteles fenestrinus

Taxon classificationAnimaliaHymenopteraBraconidae

Slater-Baker, Fagan-Jeffries, Fernández-Triana, Portmann & Oestmann sp. nov.

A0F2AC18-E889-57FC-B6C6-1E1FD589E6EF

https://zoobank.org/74C3BF3F-3B2C-4C47-B27F-2A41F62EC3F9

**Figure 34.**
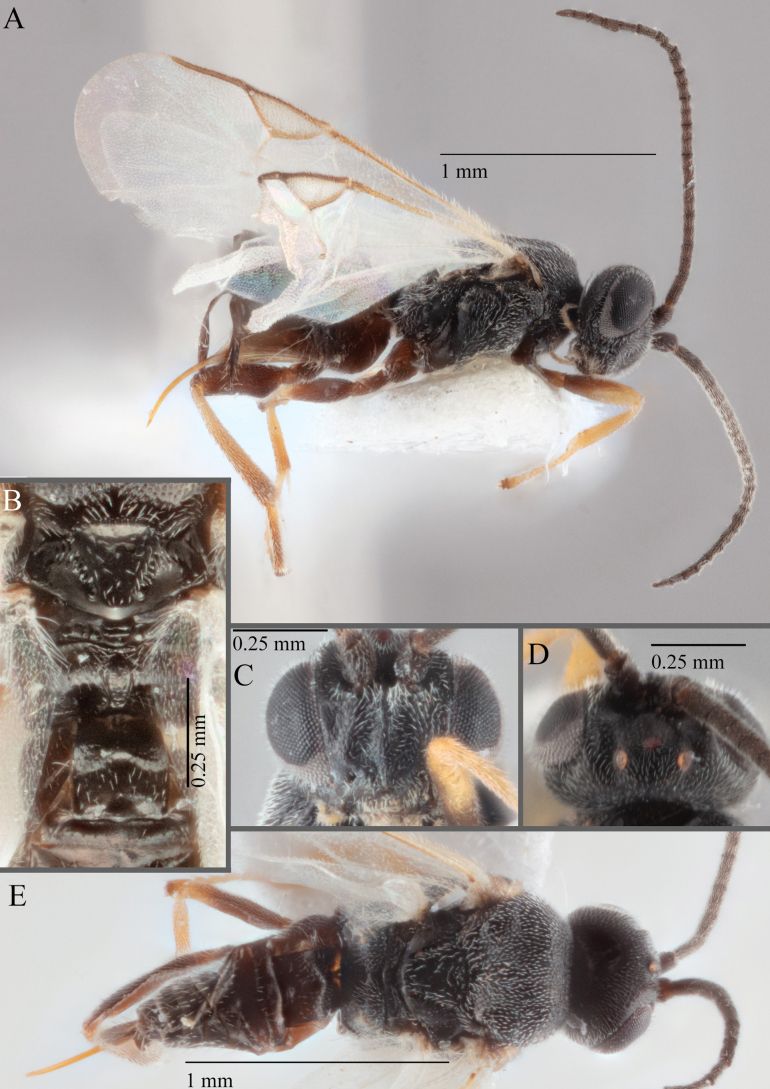
*Apantelesfenestrinus* (holotype AUMIC1310-24) **A** lateral habitus **B** dorsal propodeum, T1, and T2**C** anterior head **D** dorsal head **E** dorsal habitus.

Fig. 4C (distribution), Fig. 34 (holotype)

###### Type material.

***Holotype*.** Australia • ♀; WA, Western Pilbara Hamersley station, Railway Rd. just past Cooks Bore turnoff, Ridge paddock, UWA exclusion site; -22.3017, 117.695; 28 Oct.–02 Nov. 2005; Conservation Volunteers Australia leg.; Malaise trap; BOLD Process ID: AUMIC1310-24; WAM: 130559. ***Paratypes*.** Australia • ♀; SA, Witjira NP, Purni Bore, 88k EbS Mt Dare Hotel.; -26.2847, 136.085; 19–22 Mar. 2017; D. Yeates, A. Landford, Y. Su, X. Li, J. Lumbers & M. Irwin leg.; Malaise trap; BOLD Process ID: AUMIC1151-24; ANIC: 32-085569. • ♀; as previous except: BOLD Process ID: AUMIC1154-24; ANIC: 32-085570. • ♀; TAS, Southwest National Park, Hartz Mountain, Hartz Mountain Road.; -43.1844, 146.796; 1–9 Feb. 2016; K. Moore leg.; Yellow Pans; Bush Blitz; BOLD Process ID: AUMIC1155-24; TMAG: F150418. • ♀; WA, Western Pilbara, Hamersley station, Railway Rd. just past Cooks Bore turnoff, Ridge paddock, UWA exclusion site.; -22.3017, 117.695; 8 Oct.–02 Nov. 2005; Conservation Volunteers Australia leg.; Malaise trap; BOLD Process ID: AUMIC1345-24; WAM: 130560.

###### Diagnostic description.

***Size***: Total body length: 2.0 mm; fore wing length: 2.2 mm. ***Head***: anterior scape colour similar or only very slightly paler than head colour; F2L/W ratio: 2.8; F14L/W ratio: 1.4. ***Mesosoma***: scutoscutellar sulcus with 11 pits; mesoscutellar disc mostly smooth, or with very shallow scattered indentations; propodeal areola complete, or mostly so; propodeum mostly smooth; coxae colour (pro, meso, meta): dark all; metafemur colour mostly dark. ***Wings***: centre of pterostigma paler (more hyaline) than outer edges; fore wing r vein length/2RS vein length ratio: 0.8. ***Metasoma***: T1 shape mostly parallel, T1 medial length/anterior width between 1–2 × longer than wide; T1 mostly smooth; T2 mostly smooth; ovipositor sheath length/metatibia length ratio: 0.6.

*Apantelesfenestrinus* can be separated from the other described species of *Apanteles* known from Australia with a dark metacoxa and metafemur and the pterostigma with outer border darker than centre, centre of pterostigma pale or transparent/hyaline by having the fore wing with veins M+CU, 1 cu-a, 1M, 1CUa, 1CUb, (RS+M)a, 2RS, and 1m-cu all unpigmented, pale, or transparent and T1 smooth.

###### Etymology.

The species epithet is an adjective formed from the Latin noun *fenestra* meaning window and relates to the hyaline centre of the pterostigma.

###### Distribution.

*Apantelesfenestrinus* is represented by very dispersed collection records in northern WA, northern SA, and TAS.

###### Molecular information.

*Apantelesfenestrinus* contains sequences currently in BIN BOLD:AFQ4941. The COI sequences are at least 6.5% divergent from any of the other species treated here, or any available sequence on BOLD. The *wg* sequence of the holotype is ≥ 5 bp different to any other species. The molecular delimitation methods other than COIPTP separated *A.fenestrinus* as a discrete species; COIPTP split the Western Australian specimens from the individuals from SA/TAS.

##### ﻿﻿ Apanteles ferripulvis

Taxon classificationAnimaliaHymenopteraBraconidae

Slater-Baker, Fagan-Jeffries, Fernández-Triana, Portmann & Oestmann sp. nov.

E91443B0-FC94-5B15-9F4A-E3CE441B4BDE

https://zoobank.org/74C3BF3F-3B2C-4C47-B27F-2A41F62EC3F9

**Figure 35.**
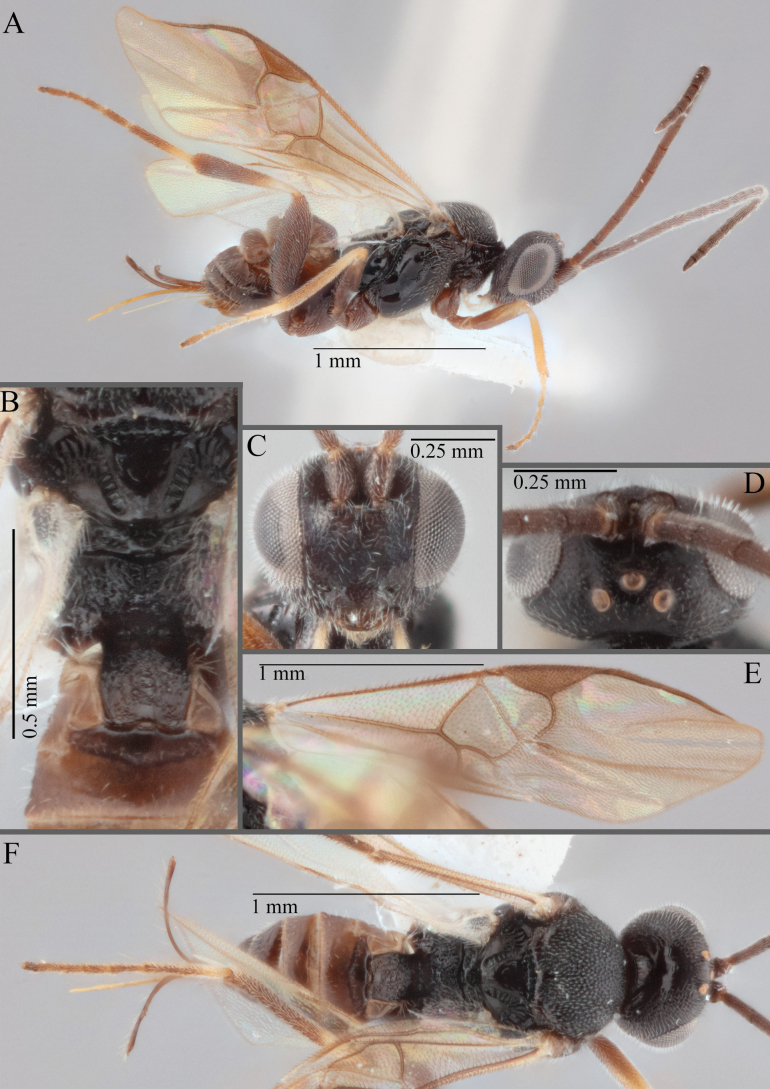
*Apantelesferripulvis* (holotype AUMIC403-18) **A** lateral habitus **B** dorsal propodeum and T1–3 **C** anterior head **D** dorsal head **E** fore wing **F** dorsal habitus.

Fig. 4C (distribution), Fig. 35 (holotype)

###### Type material.

***Holotype*.** Australia • ♀; QLD, 4 km SSE HS [homestead] Noonbah Station (NB3 M); -24.142, 143.196; 19 Jan.–7 Feb. 2009; A. Emmott leg.; Malaise trap; Sandy Plain, ghost gums; BOLD Process ID: AUMIC403-18; QM: T208351.

###### Diagnostic description.

***Size***: Total body length: 2.1 mm; fore wing length: 2.2 mm. ***Head***: anterior scape colour similar colour or only very slightly paler than head colour; F2L/W ratio: 3.7; F14L/W ratio: 1.4. ***Mesosoma***: scutoscutellar sulcus with eight pits; mesoscutellar disc mostly smooth, or with very shallow scattered indentations; propodeal areola complete, or mostly so; propodeum mostly smooth posteriorly, mostly rugose anteriorly; coxae colour (pro, meso, meta): dark all; metafemur colour mostly dark. ***Wings***: centre of pterostigma pigmented to same degree as the outer edges; fore wing r vein length/2RS vein length ratio: 1.4. ***Metasoma***: T1 shape mostly parallel, T1 medial length/anterior width between 1–2 × longer than wide; mostly rugose; T2 mostly smooth; ovipositor sheath length/metatibia length ratio: 0.9.

*Apantelesferripulvis* can be diagnosed from the other species with a dark metacoxa and metafemur, the outer side of metatibia mostly dark with a paler area in the proximal 1/3, antennae of similar length to the body, an ovipositor > 0.6 × metatibia length, the pterostigma uniformly pigmented (no hyaline centre or large pale spot), and the metatibia mostly dark with proximal pale band discrete, by infuscation on fore wing that covers most of membrane, and T3–T6 with setae not reduced to a single row, instead irregularly arranged.

###### Etymology.

The species epithet was formed from the Latin *ferrous* (made of iron) and *pulvis* (dust) and reflects both the red dust of the collection locality and the rusty-brown colour of the wings. The epithet is a noun in apposition.

###### Distribution.

*Apantelesferripulvis* is currently only known from a single specimen from central QLD.

###### Molecular information.

*Apantelesferripulvis* contains sequences currently in BIN BOLD:ADL4706. The COI sequences are at least 6% divergent from any of the other species treated here, or any available sequence on BOLD. The *wg* sequence of the holotype is ≥ 3 bp different to any other species. The molecular delimitation methods other than *wg*ASAP separated *A.ferripulvis* as a discrete species; *wg*ASAP grouped the species with the QLD individuals of *A.lamingtonensis*.

##### ﻿﻿ Apanteles focusalis

Taxon classificationAnimaliaHymenopteraBraconidae

Slater-Baker, Fagan-Jeffries, Fernández-Triana, Portmann & Oestmann sp. nov.

B36BC50B-A119-5533-8630-FCFBD12D0C8B

https://zoobank.org/D3D9964F-D8B9-47F8-9D0B-91825F827C4D

**Figure 36.**
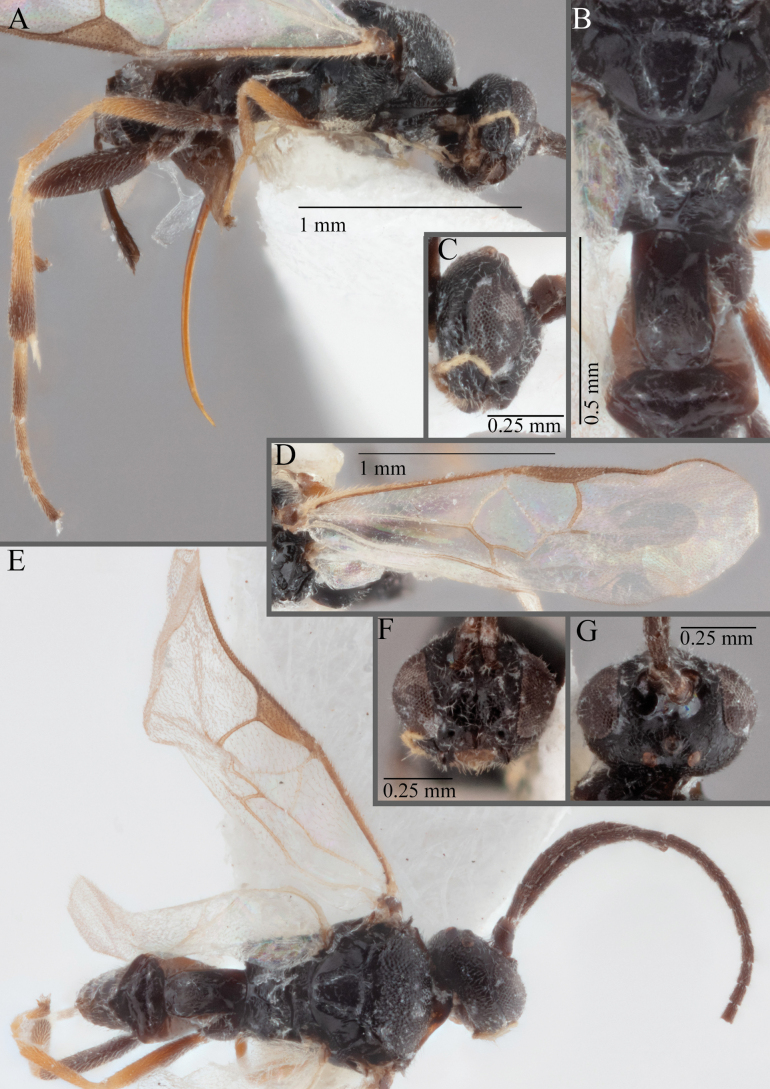
*Apantelesfocusalis* (holotype AUMIC474-18) **A** lateral habitus **B** dorsal propodeum and T1–T3 **C** lateral head **D** fore wing **E** dorsal habitus **F** anterior head **G** dorsal head.

Fig. 5B (distribution), Fig. 36 (holotype)

###### Type material.

***Holotype*.** Australia • ♀; NT, Keep River National Park: Bail-Me-Up Cr. 23.7 km SSW Jarrnarm Camp Ground; -15.9653, 129.031; 13–20 Jun. 2001; ME Irwin, FD Parker, C Lambkin leg.; Malaise in dry creekbed; BOLD Process ID: AUMIC474-18; ANIC: 32-130232.

###### Diagnostic description.

***Size***: Total body length: 2.1 mm; fore wing length: 2.3 mm. ***Head***: anterior scape colour similar or only very slightly paler than head colour; F2L/W ratio: 2.7. ***Mesosoma***: scutoscutellar sulcus with ten pits; mostly smooth, or with very shallow scattered indentations; propodeal areola complete, or mostly so; propodeum mostly rugose; coxae colour (pro, meso, meta): dark all; metafemur colour mostly dark. ***Wings***: centre of pterostigma pigmented to same degree as the outer edges; fore wing r vein length/2RS vein length ratio: 1.2. ***Metasoma***: T1 shape mostly parallel, T1 medial length/anterior width ~2 × longer than wide; T1 mostly smooth; T2 mostly smooth; ovipositor sheath length/metatibia length ratio: 0.8.

*Apantelesfocusalis* can be separated from most of the other species of *Apanteles* with dark metacoxa and metafemur, ovipositor sheaths > 0.6 × metatibia length, and antenna similar length or longer than body length, by having the metatibia mostly pale, the pterostigma uniformly coloured without a paler centre or pale spot on the proximal corner, and T1 mostly smooth with only a small rugose area in the centre. We do not diagnose this species against *A.sinusulus*, but as the species are not closely related based on molecular data, they can be identified through DNA barcoding and the placement of the sequences on a phylogeny in the context of the holotype barcodes.

###### Etymology.

This species was collected in a campground, a place which invokes imagery of campfires and warmth. The species was named for this location and also in honour of the love that KJO’s husband, Jordan Pincher, holds for cosy fires. The species epithet is a Latin adjective meaning ‘pertaining to hearth, fireplace, central point’.

###### Distribution.

*Apantelesfocusalis* is currently only known from a single specimen from northern NT.

###### Molecular information.

The holotype of *Apantelesfocusalis* is the only sequence in BIN BOLD:ADL3396. The COI sequences are at least 3% divergent from any of the other species treated here, or any available sequence on BOLD. The *wg* sequence of the holotype is at least 7 bp different to any other species and all delimitation methods resolved *A.focusalis* as a discrete species.

##### ﻿﻿ Apanteles fundulus

Taxon classificationAnimaliaHymenopteraBraconidae

Nixon, 1965

E8BC0BAA-4726-5F7C-92E0-4E5A9AA923E2

**Figure 37.**
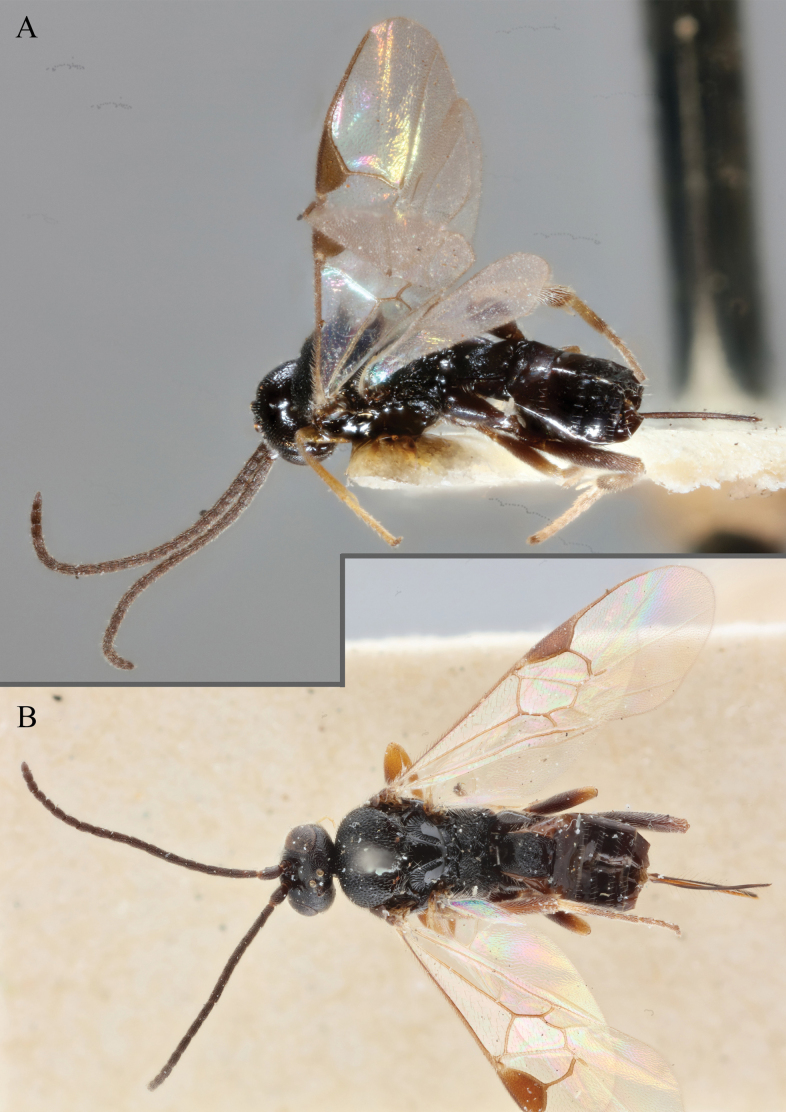
**A***Apantelesfundulus* holotype (NHM) **B***Apantelespersephone* holotype (NHM).

Fig. 6B (distribution), Fig. 37A (holotype)

###### Examined material.

***Holotype*.** Australia • ♀; S.E. QLD; Tambourine Mts.; 1–9 May1935; R. E. Turner leg.

An image of the holotype held in the NHM (Fig. 37A), with the description of the species (Nixon, 1965) were used as the basis of the diagnosis of this species.

###### Diagnosis.

*Apantelesfundulus* can be separated from other species of *Apanteles* known from Australia with a dark metacoxa, dark metafemur, the pterostigma without a pale centre area, the ovipositor sheath length > 0.6 × metatibia length, and the antenna similar length to the body by the metatibia mostly pale with a darker area only in the distal 1/3, T1 mostly smooth, with small rugose ‘trough’ in centre, the pterostigma with small pale spot at proximal corner; and setae on T3–T6 reduced to single row at posterior edge of tergite.

###### Notes.

We do not believe we have collected any material of *A.fundulus* in this study. As with all species before the advent of DNA barcoding, if it was possible to sequence DNA of the type, the placement of *A.fundulus* amongst modern material would be more certain.

##### ﻿﻿ Apanteles galleriae

Taxon classificationAnimaliaHymenopteraBraconidae

Wilkinson, 1932

D73865B0-3167-5EC6-B75D-B691548B416E

**Figure 38.**
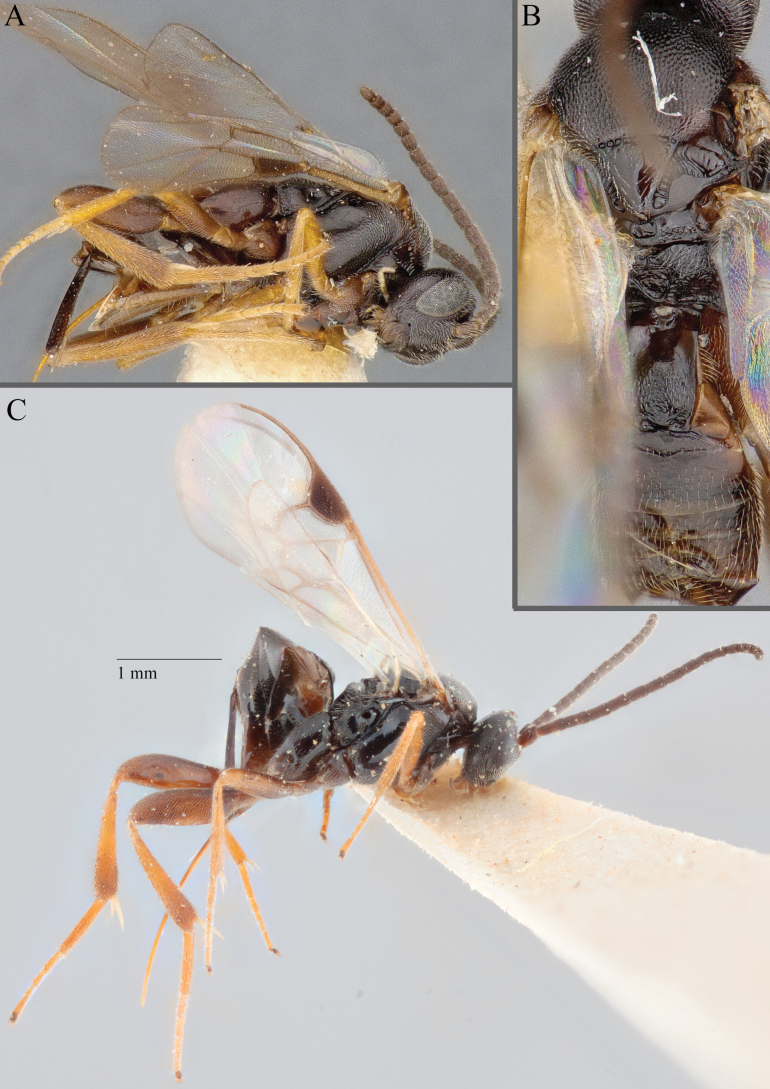
*Apantelesgalleriae***A** lateral habitus (France – CNC497178) **B** dorsal mesosoma and metasoma (France – CNC497178) **C** lateral habitus (India – WINC).

Fig. 38 (examined material)

###### Holotype information.

♀; France (NHM).

###### Examined material.

Type not examined, but images of a female specimen from France (CNC497178) and one female with label data “India. N.P. Bee Res. Stat. Nagrota - Bagwan (Kangra) VI.1984; of *Achroiagrisella* in hives of *Apisindica*, C.I.E.A. 16226; *Apantelesgalleriae* Wilk. det. A.D. Austin, 1984.” (WINC) formed the basis of the diagnosis.

###### Diagnosis.

*Apantelesgalleriae* can be separated from the other species of *Apanteles* known from Australia that have a dark metacoxa and metafemur, and the pterostigma without a pale centre and the ovipositor sheath length > 0.6 × metatibia length by having the antennae significantly shorter (~ 0.6–0.7×) than body length and T3 with multiple rows of setae.

###### Notes.

*Apantelesgalleriae* is a parasitoid of several species of wax moths (Pyralidae) associated with honey bees (Fernández-Triana et al. 2014) and the species is found in numerous countries around the world (Fernández-Triana et al. 2020). Whilst the species definitely occurs in New Zealand, confirmed by DNA barcoding (e.g., BOLD Process ID: NZMG202-11), we have not confirmed the presence of the species in Australia during this study. The record of *A.galleriae* occurring in Australia is from Austin and Dangerfield (1992), but we have not been able to trace that record to the corresponding literature or specimens.

##### ﻿﻿ Apanteles hades sp. nov.

Taxon classificationAnimaliaHymenopteraBraconidae

6B5B68F5-762C-548A-9F2D-904D54B9D600

https://zoobank.org/B484A091-F85E-4492-BFED-C4F10DD5247A

**Figure 39.**
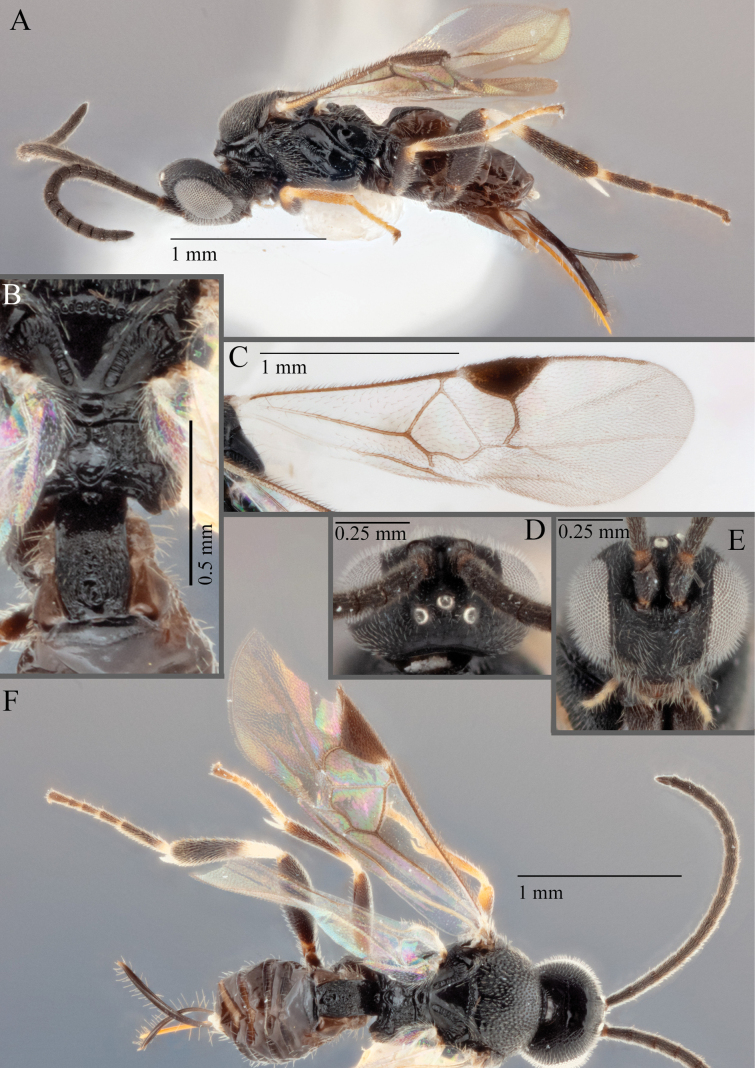
*Apanteleshades***A** lateral habitus (holotype AUMIC244-18) **B** propodeum and T1–3 (holotype AUMIC244-18) **C** fore wing (paratype OZBOL412-21) **D** dorsal head (holotype AUMIC244-18) **E** anterior head (holotype AUMIC244-18) **F** dorsal habitus and wings (holotype AUMIC244-18).

Fig. 4C (distribution), Fig. 39 (holotype/paratype)

###### Type material.

***Holotype*.** Australia • ♀; NSW, Pearl Beach, Cormellin Biological Field Station; -33.5511, 151.298; Dec. 2009; A.D. Austin leg.; BOLD Process ID: AUMIC244-18; AM: K.647421. ***Paratypes*.** Australia • ♂; NSW, Barren Grounds, NR 21 m NE off Barren Ground Rd.; -34.6697, 150.712; 1–6 Feb. 2020; K. M. Bayless & J. G. Lumbers leg.; Malaise trap; BOLD Process ID: AUMIC1166-24; ANIC: 32-085543. • ♂; as previous except: BOLD Process ID: AUMIC1168-24; ANIC: 32-085544. • ♀; NSW, Budderoo NP, Carrington Falls, off Boundary Tk.; -34.6276, 150.655; 3–10 Feb. 2020; K. M. Bayless, J. G. Lumbers & D. K. Yeates leg.; 6m Malaise trap; BOLD Process ID: AUMIC1171-24; ANIC: 32-085545. • ♀; NSW, Pearl Beach, Cormellin Biological Field Station; -33.5511, 151.298; Nov. 2009; A.D. Austin leg.; BOLD Process ID: AUMIC241-18; AM: K.647422. • ♀; as previous except: Dec. 2009; BOLD Process ID: AUMIC245-18; AM: K.647423. • ♀; as previous except: BOLD Process ID: AUMIC304-18; AM: K.647424. • ♀; QLD, Cainbable Quarry, OF; -28.145, 153.113; 06–22 Jan. 2009; G. Monteith leg.; Malaise trap; BOLD Process ID: AUMIC070-18; QM: T208347. • ♀; QLD, Cliento Conservation Reserve Nambour; -26.6146, 152.953; 02 Oct. 2020; E. Fagan-Jeffries leg.; Sweeping; BOLD Process ID: OZBOL412-21; QM: T261231.

###### Diagnostic description.

***Size***: Total body length: 2.5 mm; fore wing length: 2.3 mm. ***Head***: anterior scape colour similar or only very slightly paler than head colour; F2L/W ratio: 2.3; F14L/W ratio: 1.1. ***Mesosoma***: scutoscutellar sulcus with ten pits; mesoscutellar disc mostly smooth, or with very shallow scattered indentations; propodeal areola complete, or mostly so; propodeum mostly rugose; coxae colour (pro, meso, meta): dark all; metafemur colour mostly dark. ***Wings***: centre of pterostigma pigmented to same degree as the outer edges; fore wing r vein length/2RS vein length ratio: 1.6. ***Metasoma***: T1 shape mostly parallel, T1 medial length/anterior width between 1–2 × longer than wide; T1 mostly rugose; T2 mostly smooth; ovipositor sheath length/metatibia length ratio: 1.1.

*Apanteleshades* is part of a trio of species (with *A.alatomicans* and *A.magicus*) that are difficult to diagnose morphologically from each other; for the identification of these species, we recommend DNA barcoding and the placement of the unknown sequence in the context of a phylogeny that includes validated sequences. However, *A.hades* can be separated from the other described species of *Apanteles* known from Australia that have a dark metacoxa and metafemur, pterostigma without a pale centre, ovipositor sheaths approximately the same length as the metatibia, and antennae of similar length to the length of the body, by having the metatibia with a very pale (almost white) discrete band in the proximal 1/3 and otherwise dark and with infuscation on fore wing which is restricted to area around veins r and 1CUb.

###### Etymology.

The species epithet references Hades, the god of the Underworld in ancient Greek mythology, and was inspired by the morphological similarity of this species (amongst others) to *A. persephone* Nixon; Persephone was Queen of the Underworld and Hades’ spouse. The epithet should be treated as a noun in apposition.

###### Distribution.

*Apanteleshades* is known from along the east coast of Australia.

###### Molecular information.

Sequences of *Apanteleshades* are currently in two BINs: BOLD:ADL5325 and BOLD:ADL3408. The sequences in different BINs are up to 4.5% divergent, which is irregularly high for intraspecific divergence in microgastrines. However, the *wg* sequences of individuals in the two BINs are identical to each other, and at least 3 bp different to other species. COIASAP and *wg*PTP delimitation analyses resolved *A.hades* as a discrete species, COIPTP split the species into two (following the BINs/2% divergence grouping), whilst *wg*ASAP grouped the species with *A.alatomicans*. *Apanteleshades* is therefore not a well resolved species, however we have taken the balanced approach to split it from *A.alatomicans* but retain individuals from both BINs listed above in a single species because of the identical nuclear gene, despite the large divergence in COI. More data, including host information or further genetics, may resolve this species more clearly.

##### ﻿﻿ Apanteles hemara

Taxon classificationAnimaliaHymenopteraBraconidae

Nixon, 1965

608D43CA-DF65-5EAF-B77A-B8C72BBF5147

Fig. 40

###### Holotype information.

♀; India (NHM).

###### Examined material.

Images of the holotype were examined alongside images of verified specimens held in the CNC. The redescription in Fernández-Triana et al. (2017) was also consulted to form the diagnosis.

###### Diagnosis.

*Apantleshemara* is particularly distinctive amongst the other species of *Apanteles* in Australia. It can be distinguished from other species with a dark metacoxa and pale trochanter by the T2 entirely sculptured with strong longitudinal striae, the T1 slightly widening on posterior 1/2 and entirely and coarsely sculptured, and propodeum mostly smooth but with complete and strong lateral carinae.

**Figure 40.**
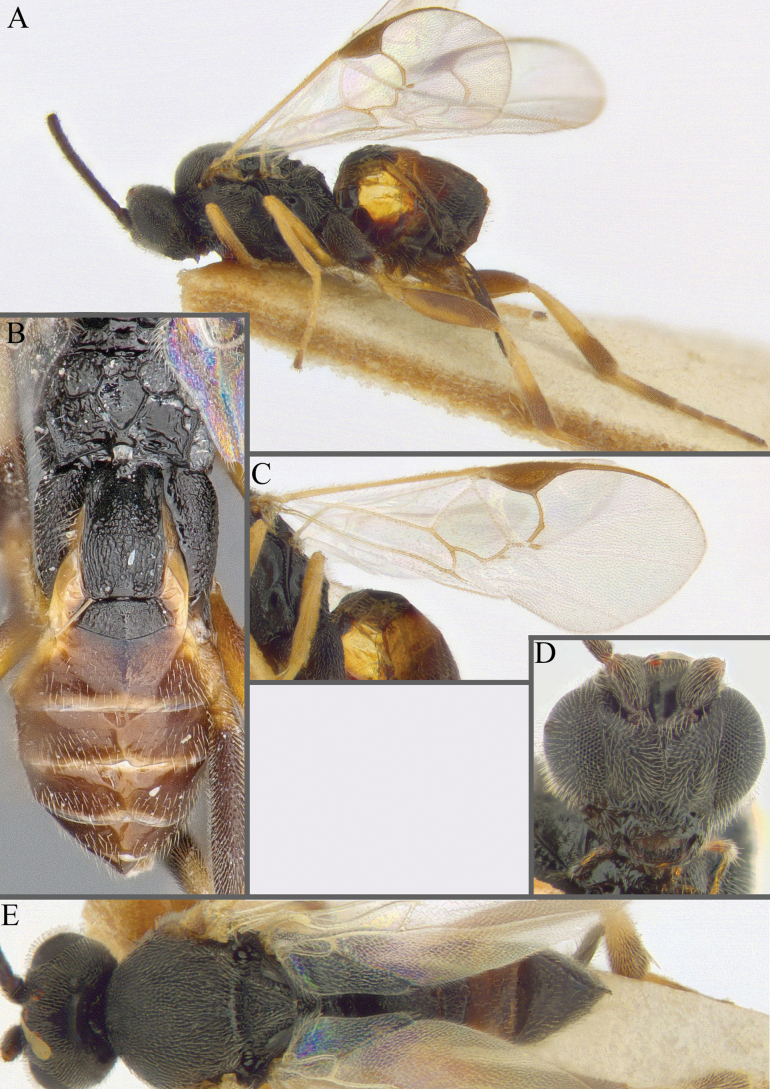
*Apanteleshemara***A** lateral habitus (holotype) **B** dorsal propodeum and metasoma (Yemen – CNC661375) **C** fore wing (holotype) **D** anterior head (holotype) **E** dorsal habitus (holotype).

###### Notes.

The record of *A.hemara* occurring in Australia comes from the original description of Nixon (1965) where he lists a specimen from the “F.C.T. [ACT]”. However, we have not located or examined the specimen, which we assume would be located in the NHM or potentially ANIC. Regardless, we did not collect any conspecific specimens in Australia during this study. *Apanteleshemara* is reported to parasitise several species of moths in the families Choreutidae and Crambidae, including species which occur in Australia.

##### ﻿﻿ Apanteles insulanus

Taxon classificationAnimaliaHymenopteraBraconidae

Slater-Baker, Fagan-Jeffries, Fernández-Triana, Portmann & Oestmann sp. nov.

6583EC46-AF3C-5276-8FC6-AFA27CD9D4E9

https://zoobank.org/94FDDCCE-4F92-406B-83FC-178BE993FB4C

**Figure 41.**
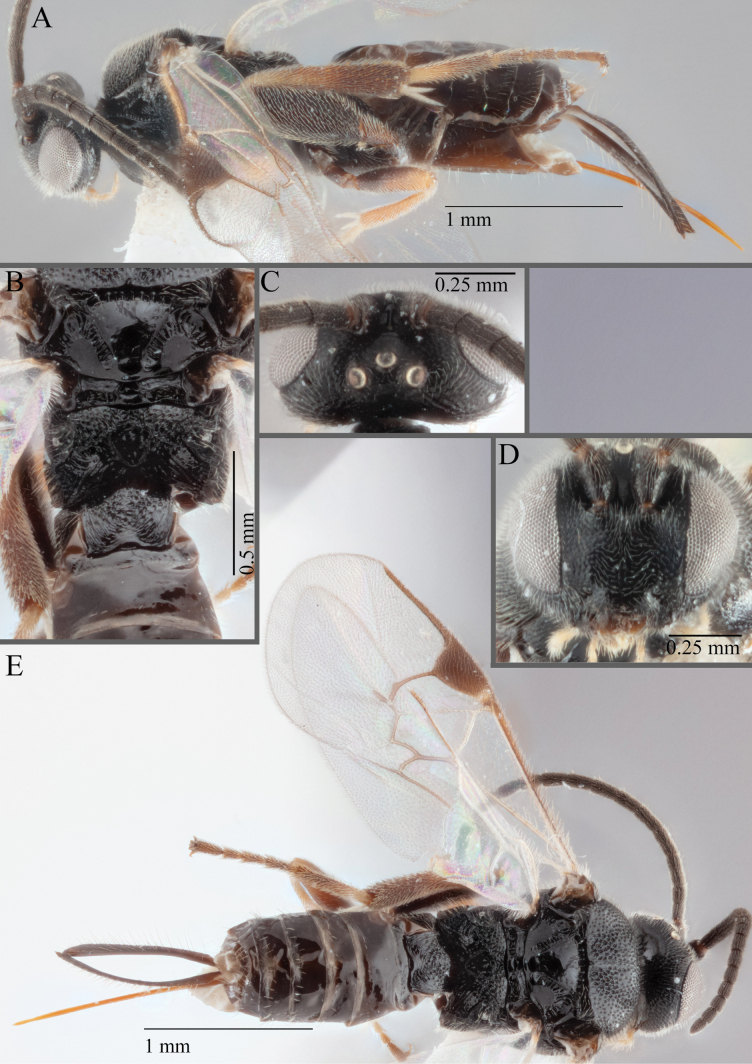
*Apantelesinsulanus* (holotype AUMIC1205-24) **A** lateral habitus **B** dorsal propodeum and T1–3 **C** dorsal head **D** anterior head **E** dorsal habitus and wings.

Fig. 4C (distribution), Fig. 41 (holotype)

###### Type material.

***Holotype*.** Australia • ♀; NSW, Lord Howe Island, Lidgbird, East shelf.; -31.5636, 159.085; 9–16 Feb. 2017; C.A. Reid leg.; Malaise trap; BOLD Process ID: AUMIC1205-24; AM: K.377411.

###### Diagnostic description.

***Size***: Total body length: 3.0 mm; fore wing length: 2.9 mm. ***Head***: anterior scape colour similar or only very slightly paler than head colour; F2L/W ratio: 2.5; F14L/W ratio: 1.2. ***Mesosoma***: scutoscutellar sulcus with nine pits; mesoscutellar disc mostly smooth, or with very shallow scattered indentations; propodeal areola complete, or mostly so; propodeum mostly smooth posteriorly, mostly rugose anteriorly; coxae colour (pro, meso, meta): dark all; metafemur colour mostly dark. ***Wings***: centre of pterostigma pigmented to same degree as the outer edges; fore wing r vein length/2RS vein length ratio: 1.7. ***Metasoma***: T1 shape mostly parallel, T1 medial length/anterior width between 1–2 × longer than wide; mostly rugose; T2 mostly smooth; ovipositor sheath length/metatibia length ratio: 1.4.

*Apantelesinsulanus* can be separated from most other species of *Apanteles* in Australia that have a dark metacoxa and metafemur and the pterostigma without a pale centre, the ovipositor sheath length >0.6 × metatibia length and the antenna of similar size to the body length by the pterostigma having a small pale spot proximally, the metatibia displaying a gradient of colouration from pale to dark, the colours merging in the centre, T1 with strong sculpture over at least most of posterior 1/2 of tergite, mesoscutellar disc with at most scattered punctures along margins, and fore wing vein 1M much less pigmented (often transparent/pale) compared to pigmentation of vein 1CUa. Compared to *A.brockhedgesi*, *A.insulanus* has the scutoscutellar sulcus comparatively wider and with comparatively larger pits. The species can currently be best separated from *A.ramsaris* by DNA barcoding, and placement of the unknown sequence in the context of a phylogeny with the holotype barcodes of both species.

###### Etymology.

The species epithet is a Latin adjective meaning ‘of or pertaining to an island’ and relates to the collection locality.

###### Distribution.

*Apantelesinsulanus* is currently only known from one specimen collected on Lord Howe Island as part of an Australian Museum expedition.

###### Molecular information.

The holotype of *Apantelesinsulanus* is the only sequence in BIN BOLD:AFQ4216. The COI sequences are at least 2.4% divergent from any of the other species treated here, or any available sequence on BOLD. The *wg* sequence of the holotype is at least 2 bp different to any other species. Most delimitation methods resolved *A.insulanus* as a discrete species, except for *wg*ASAP and PTP, which grouped the species with *A.ramsaris*.

###### Remarks.

We do not have a strong morphological character to separate *A.insulanus* from *A.ramsaris*, and as both the COI and *wg* sequences have low divergences, it is possible these species are conspecific. However, because of the concordance among the majority of the molecular delimitation methods, and the disparate collection localities (Riverland SA and Lord Howe Island) we split this group into two species for now.

##### ﻿﻿ Apanteles ippeus

Taxon classificationAnimaliaHymenopteraBraconidae

Nixon, 1965

93C1652A-5A9D-529D-9E2F-BFC82CE34FA3

**Figure 42.**
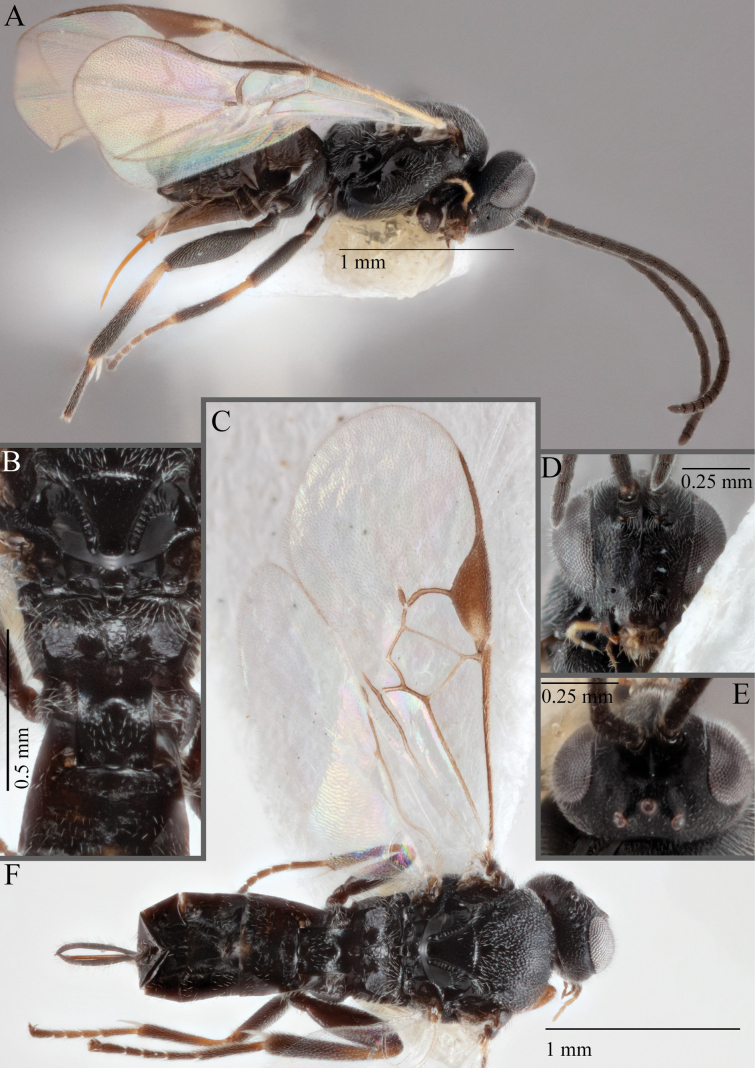
*Apantelesippeus* examined material **A** lateral habitus (AUMIC313-18) **B** dorsal propodeum and T1–3 (AUMIC745-23) **C** fore and hind wing (AUMIC745-23) **D** anterior head (AUMIC313-18) **E** dorsal head (AUMIC313-18) **F** dorsal habitus (AUMIC745-23).

**Figure 43.**
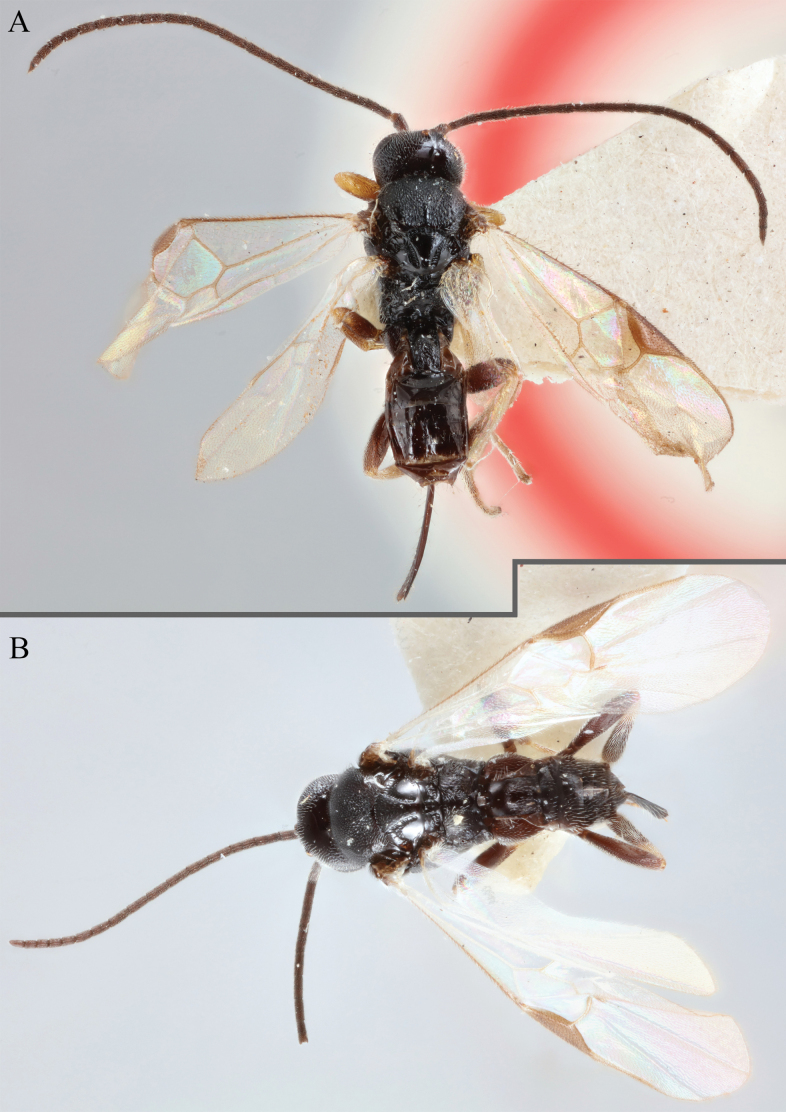
**A***Apantelesvala* (holotype, NHM) **B***Apantelesippeus* (holotype, NHM).

Fig. 5A (distribution), Fig. 42 (examined material), Fig. 43B (holotype)

###### Holotype information.

♀; Australia, Canberra, “bred from *Plutellamaculipennis*, (F. Wilson)” (NHM). Images of the type examined.

###### Examined material.

64♀, 13♂, 2?; from ACT, NSW, QLD, SA, and WA; see Suppl. material 3 for full collection details.

###### Diagnosis.

*Apantelesippeus* can be separated from the other species of *Apanteles* in Australia that have a dark metacoxa and metafemur, the pterostigma without a pale centre, the ovipositor sheaths > 0.6 × metatibia length and the antenna of similar length to the body length by T1 having very straight parallel sides, the pterostigma with large conspicuous pale spot and propodeal areola narrower than most species (i.e., as in Fig. 42B).

###### Notes.

The specimens collected in this study were initially identified through a DNA barcode match to a specimen collected and sequenced as part of an investigation into predators and parasitoids in brassica crops in southern Australia (Juen et al. 2012). Morphology aligned with that of the holotype of *A.ippeus*, reared from *Plutellaxylostella*, a known pest of brassica in Australia, and we therefore consider this a reasonably reliable identification.

##### ﻿﻿ Apanteles kelpiellus

Taxon classificationAnimaliaHymenopteraBraconidae

Slater-Baker, Fagan-Jeffries, Fernández-Triana, Portmann & Oestmann sp. nov.

975B4319-8C35-56E7-8E45-1CBCD17C2C09

https://zoobank.org/E775FEF6-7634-42DC-BEA8-7012DF6FA7DA

**Figure 44.**
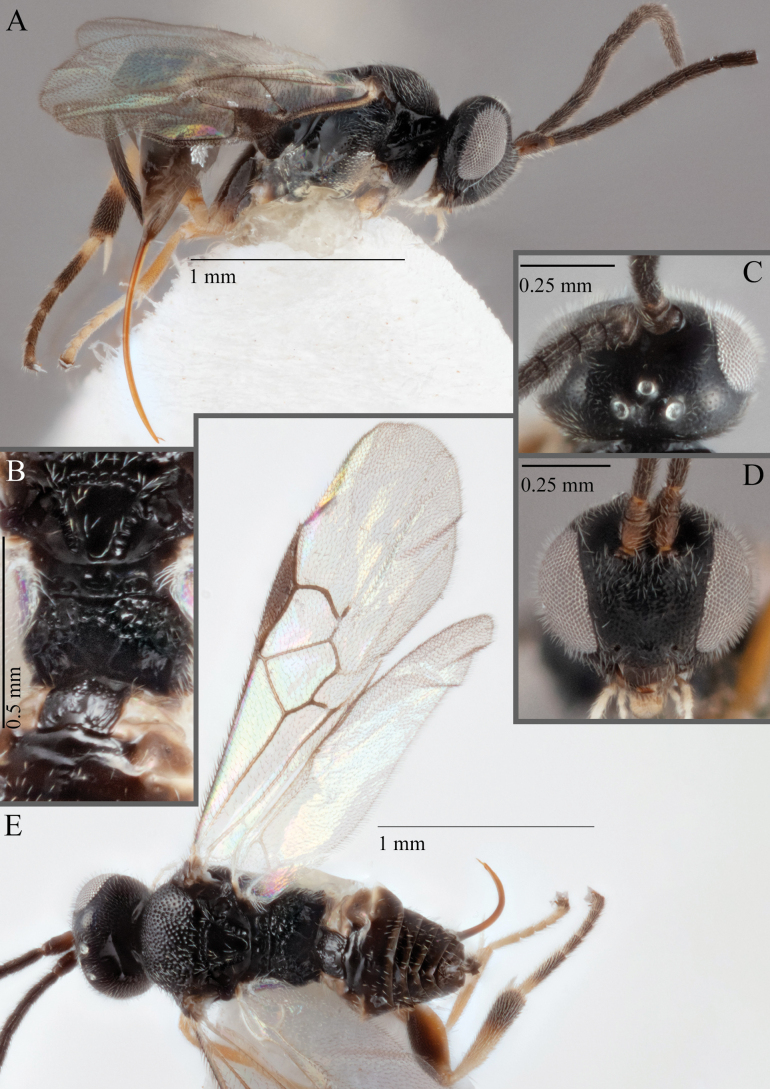
*Apanteleskelpiellus* (holotype AUMIC334-18) **A** lateral habitus **B** dorsal propodeum T1–2 **C** dorsal head **D** anterior head **E** dorsal habitus and wings.

Fig. 5B (distribution), Fig. 44 (holotype)

###### Type material.

***Holotype*.** Australia • ♀; QLD, Kuranda; -16.8154, 145.643; 16 Mar.–12 Apr. 2017; M.S. Moulds leg.; Malaise trap; BOLD Process ID: AUMIC334-18; QM: T261199. ***Paratypes*.** Australia • ♂; QLD, Daintree National Park; -16.0765, 145.472; 28 m; 17 Nov. 2019; E. Fagan-Jeffries & J. B. Dorey leg.; Sweeping vegetation; BOLD Process ID: AUMIC1233-24; QM: T261200. • ♀; QLD, Kuranda; -16.8154, 145.643; 16 Mar.–12 Apr. 2017; M.S. Moulds leg.; Malaise trap; BOLD Process ID: AUMIC330-18; QM: T261201.

###### Diagnostic description.

***Size***: Total body length: 2.0 mm; fore wing length: 2.2 mm. ***Head***: anterior scape colour moderately paler than head colour; F2L/W ratio: 3.2; F14L/W ratio: 1.4. ***Mesosoma***: scutoscutellar sulcus with eight pits; mesoscutellar disc mostly smooth, or with very shallow scattered indentations; propodeal areola complete, or mostly so; propodeum mostly smooth posteriorly, mostly rugose anteriorly; coxae colour (pro, meso, meta): dark all; metafemur colour mostly dark. ***Wings***: centre of pterostigma pigmented to same degree as the outer edges; fore wing r vein length/2RS vein length ratio: 1.0. ***Metasoma***: T1 shape mostly parallel, T1 medial length/anterior width between 1–2 × longer than wide; T1 mostly rugose; T2 mostly smooth; ovipositor sheath length/metatibia length ratio: 0.7.

*Apanteleskelpiellus* can be separated from the other described species of *Apanteles* known from Australia with a dark metacoxa by the metafemur mostly dark with pale area only in proximal 1/4, T2 smooth, and the anterior side of scape moderately paler than head colour.

###### Etymology.

The species epithet means ‘little kelpie’. A kelpie is a breed of dog in Australia commonly used as a sheepdog or companion animal. The epithet references the yellow anterior of the scape of the new species, which resembles the spots above a kelpie’s eyes. The epithet is a noun in apposition.

###### Distribution.

*Apanteleskelpiellus* is currently only known from northern QLD.

###### Molecular information.

Sequences of *Apanteleskelpiellus* are currently in BIN BOLD:ADL3948. The COI sequences are at least 7% divergent from any of the other species treated here, or any available sequence on BOLD. The *wg* sequence of the holotype is at least 5 bp different to any other species. Most delimitation methods resolved *A.kelpiellus* as a discrete species, however COIPTP split one of the paratypes apart, and *wg*PTP grouped the species with *A.margaritarius*.

##### ﻿﻿ Apanteles lamingtonensis

Taxon classificationAnimaliaHymenopteraBraconidae

Slater-Baker, Fagan-Jeffries, Fernández-Triana, Portmann & Oestmann sp. nov.

1CDE045E-B5B0-5E91-8A15-E2093BFEE586

https://zoobank.org/0C18363E-00D1-4563-980D-5301FD1B112E

**Figure 45.**
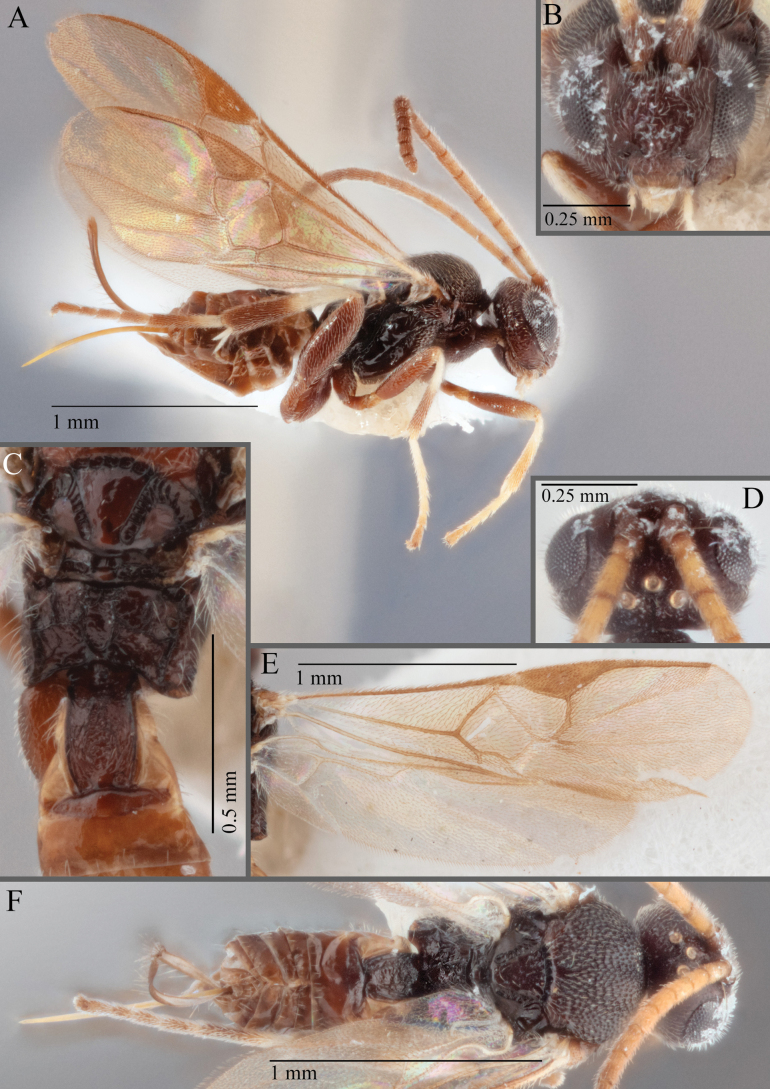
*Apanteleslamingtonensis* (holotype AUMIC436-18) **A** lateral habitus **B** anterior head **C** dorsal propodeum and T1–T3 **D** dorsal head **E** fore and hind wing **F** dorsal habitus.

Fig. 5B (distribution), Fig. 45 (holotype)

###### Type material.

***Holotype*.** Australia • ♀; QLD, Lamington NP; -28.21, 153.139; 15–25 Jan. 2007; C Lambkin, N. Starick leg.; Malaise trap; IBISCA Plot # IQ-500-C rainforest; BOLD Process ID: AUMIC436-18; QM: T208354. ***Paratypes*.** Australia • ♀; QLD, Lamington NP; -28.21, 153.139; 15–25 Jan. 2007; C Lambkin, N. Starick leg.; Malaise trap; IBISCA Plot # IQ-500-C rainforest; BOLD Process ID: AUMIC435-18; QM: T208352. • ♀; QLD, Lamington NP; -28.188, 153.121; 10–20 Apr. 2007; Lambkin, Marcora, Starick leg.; Malaise trap; IBISCA Plot # IQ-700-A rainforest; BOLD Process ID: AUMIC439-18; QM: T208353.

###### Examined material.

Australia • ♀; ACT, Canberra, CSIRO Black Mountain, close to Botanic Garden fence; -35.2736, 149.102; 31 Jan.–6 Feb. 2018; T. Pleines & J. Rodriguez leg.; Malaise trap; BOLD Process ID: AUMIC1149-24; ANIC: 32-085546. • ♀; as previous except: 2 Apr.–17 Aug. 2017; BOLD Process ID: AUMIC1000-24; ANIC: 32-085547.

###### Diagnostic description.

***Size***: Total body length: 2.0 mm; fore wing length: 2.1 mm. ***Head***: anterior scape colour slightly paler than head colour; F2L/W ratio: 3.0; F14L/W ratio: 0.9. ***Mesosoma***: scutoscutellar sulcus with six pits; mesoscutellar disc mostly smooth, or with very shallow scattered indentations; propodeal areola complete, or mostly so; propodeum mostly smooth; coxae colour (pro, meso, meta): dark all; metafemur colour mostly dark. ***Wings***: centre of pterostigma pigmented to same degree as the outer edges; fore wing r vein length/2RS vein length ratio: 1.3. ***Metasoma***: T1 shape mostly parallel, T1 medial length/anterior width between 1–2 × longer than wide; T1 mostly rugose; T2 mostly smooth; ovipositor sheath length/metatibia length ratio: 1.0.

*Apanteleslamingtonensis* can be diagnosed from the other species with a dark metacoxa and metafemur, the outer side of metatibia mostly dark with a discrete proximal paler area, antennae of similar length to the body, an ovipositor > 0.6 × metatibia length, and the pterostigma uniformly pigmented (no hyaline centre or large pale spot), by infuscation on fore wing that covers most of membrane, and T3–6 with setae reduced to a single row.

###### Etymology.

The epithet is an adjective formed from the type locality, Lamington National Park.

###### Distribution.

*Apanteleslamingtonensis* is currently known from the east coast of QLD and potentially from the ACT (see Remarks).

###### Molecular information.

Sequences of *Apanteleslamingtonensis* are currently in BIN BOLD:ADL3013. The COI sequences are at least 5% divergent from any of the other species treated here, or any available sequence on BOLD. The *wg* sequence of paratype AUMIC439-1 (no *wg* available for the holotype) is at least 3 bp different to any other species. The molecular delimitation of this species is poorly resolved, with COI divergence <2%, and the sequences of both the QLD and ACT material group in a single BIN. However, COIASAP groups the species with *A.magicus*, COIPTP splits one sequence (not examined material, a sequence on BOLD from QLD) from the other *A.lamingtonensis* material, there are two *wg* haplotypes 5 bp different that match the *wg*PTP split (split between QLD and ACT individuals), and the *wg*ASAP analysis groups the species with *A.ferripulvis*. In short, molecular delimitation failed to reach any sort of concordance.

###### Remarks.

It is important to note that the individuals from the ACT are much darker (the classic black/dark brown colouration found in most Australian *Apanteles*) than the holotype, the antennae are uniformly dark brown, and they have less infuscation on the membrane (so that they become difficult to separate from the wing infuscation of species like *A.alatomicans*). There is also slight variation in the rugosity of the propodeum, with the ACT specimens with more rugose sculpturing on the anterior 1/2 of the propodeum. With the incongruence of the molecular delimitation, including the *wg* barcodes being 5 bp different to the individuals from Lamington NP (the type locality) it is very possible that the ACT specimens are actually a different species. For now, we assign the ACT material to *A.lamingtonensis* but do not include them in the type series, and hope that greater sampling effort can help better define the species boundaries.

##### ﻿﻿ Apanteles ligdus

Taxon classificationAnimaliaHymenopteraBraconidae

Slater-Baker, Fagan-Jeffries, Fernández-Triana, Portmann & Oestmann sp. nov.

2D4EF4E9-A85D-58C5-A25D-30C7D3F3F82B

https://zoobank.org/4F444A0A-3DC8-4EED-B65E-8741B83AB7D8

**Figure 46.**
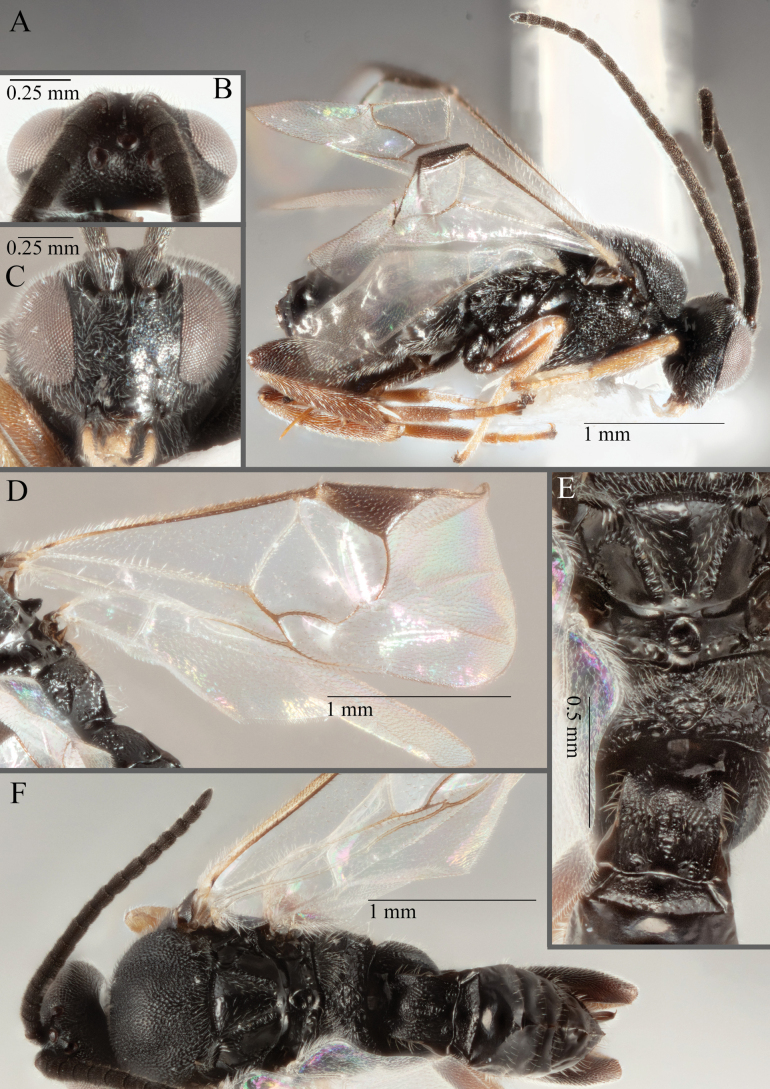
*Apantelesligdus* (holotype AUMIC729-23) **A** lateral habitus **B** dorsal head **C** anterior head **D** fore and hind wing **E** dorsal propodeum and T1–3 **F** dorsal habitus.

Fig. 5A (distribution), Fig. 46 (holotype)

###### Type material.

***Holotype*.** Australia • ♀; QLD, Specimen Hill, Herberton; -17.3823, 145.372; 14 Mar. 2021; E.P. Beaver, M.F. Braby leg.; Reared from Instar III larva of *Ogyrisiphis* (Lepidoptera: Lycaenidae) collected 05 March 2021 on *Dendrophthoe* sp.; wasp larva emerged and pupated same day. Adult eclosed 14 March 2021; BOLD Process ID: AUMIC729-23; QM: T261208. ***Paratype*.** Australia • ♂; as previous except: wasp larva emerged and pupated 14 March 2021, adult eclosed 21 March 2021; BOLD Process ID: AUMIC728-23; QM: T261209.

###### Diagnostic description.

***Size***: Total body length: 3.4 mm; fore wing length: 2.8 mm. ***Head***: anterior scape colour similar or only very slightly paler than head colour; F2L/W ratio: 2.3; F14L/W ratio: 1.4. ***Mesosoma***: scutoscutellar sulcus with 13 pits; mesoscutellar disc with punctures in outer regions, centre smooth; propodeal areola complete, or mostly so; propodeum mostly rugose; coxae colour (pro, meso, meta): dark all; metafemur colour mostly dark. ***Wings***: centre of pterostigma pigmented to same degree as the outer edges; fore wing r vein length/2RS vein length ratio: 1.6. ***Metasoma***: T1 shape mostly parallel, T1 medial length/anterior width between 1–2 × longer than wide; T1 mostly rugose; T2 with fine sculpture; ovipositor sheath length/metatibia length ratio: 0.4.

*Apantelesligdus* can be separated from most other species of *Apanteles* in Australia that have a dark metacoxa and metafemur, the pterostigma without a pale centre, and the ovipositor sheath length < 0.5 × the metatibia length, by T1 parallel sided; the median length of propodeum 1.2 × the maximum width of areola. *Apantelesligdus* can be separated from *A.ethanbeaveri* by the scutoscutellar sulcus narrower and with much smaller pits, T2 comparatively more transverse and T2 with posterior margin more or less straight or very slightly curved.

###### Etymology.

The species epithet references Ligdus from Greek mythology, who threatened to kill his child (Iphis) if it was born female; relevant because the new species parasitises and kills the lepidopteran *Ogyrus iphis*. The epithet is a noun in apposition.

###### Distribution.

*Apantelesligdus* is currently only known from one collection event in northern QLD, from two different host individuals of *Ogyrisiphis* on *Dendrophthoe* sp.

###### Molecular information.

The two sequences of *A.ligdus* are currently in BIN BOLD:AFF1514. The COI sequences are at least 2.4% divergent from any of the other species treated here, or any available sequence on BOLD. The *wg* sequence of the holotype is ≥ 1 bp different to *A.ethanbeaveri*, and ≥ 10 bp different to any other species. The molecular delimitation of this species relative to *A.ethanbeaveri* is poorly resolved: BINs, a 2% threshold, COIPTP and the *wg* haplotypes split the two species, COIASAP and *wg*ASAP and *wg*PTP grouped the two species together.

###### Remarks.

The collectors who reared the type series are professional lepidopterists, and therefore the host records of this species should be treated with reasonable confidence. The species is closely related to *A.ethanbeaveri* and may potentially be conspecific. We feel the available evidence at present supports considering them as being distinct species (multiple subtle morphological differences, > 2% COI divergence, *wg* barcodes 1 bp different, non-overlapping host species) but a larger sample size and further study may change this species hypothesis.

##### ﻿﻿ Apanteles magicus

Taxon classificationAnimaliaHymenopteraBraconidae

Slater-Baker, Fagan-Jeffries, Fernández-Triana, Portmann & Oestmann sp. nov.

5A0F58D7-0E40-5170-A490-E00B390E69AE

https://zoobank.org/23F2FDF6-8F1C-41DC-B93E-F4867C299103

**Figure 47.**
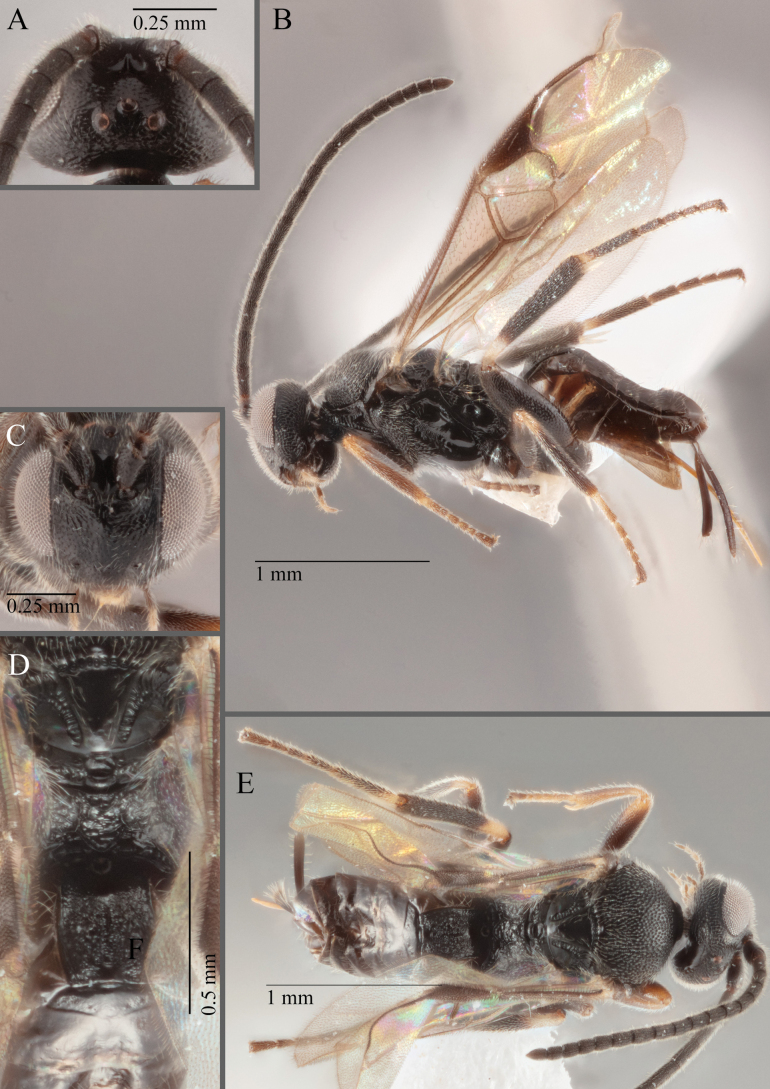
*Apantelesmagicus* (holotype AUMIC620-23) **A** dorsal head **B** lateral habitus **C** anterior head **D** dorsal mesoscutellar disc, propodeum and T1–3 **E** dorsal habitus.

**Figure 48.**
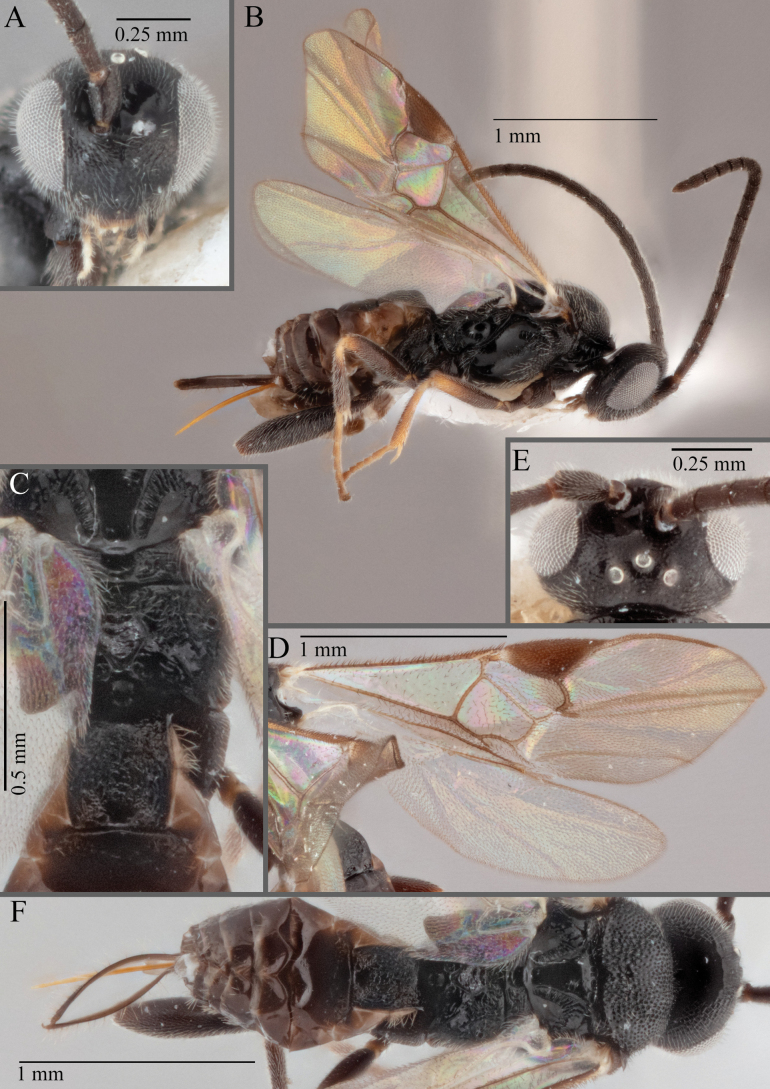
*Apantelesmagicus* (paratype AUMIC283-18) **A** anterior head **B** lateral head **C** dorsal propodeum and T1– 2 **D** fore and hind wing **E** dorsal head **F** dorsal habitus.

Fig. 6A (distribution), Fig. 47 (holotype), Fig. 48 (paratype)

###### Type material.

***Holotype*.** Australia • ♀; SA, Kangaroo Island, Lot 51 HA just near end of driveway near 3^rd^ power pole; -35.9038, 137.551; 13–17 Apr. 2020; R. Glatz leg.; Malaise trap; BOLD Process ID: AUMIC620-23; SAMA: 32-47763. ***Paratypes*.** Australia • ♀; ACT, Canberra, Black Mtn Res; -35.2741, 149.111; 28 Feb.–19 Mar. 2018; K. M. Bayless leg.; Malaise trap; BOLD Process ID: AUMIC1200-24; ANIC: 32-085548. • ♀; ACT, Namadgi NP Birrigai, track to Mushroom Rock; -35.458, 148.961; 27 Nov.–05 Dec. 2018; Evangelista & Rodriguez leg.; Malaise trap; Bush Blitz; BOLD Process ID: AUMIC986-24; ANIC: 32-085549. • ♀; ACT, Tidbinbilla Nat. Res., Birrigai Outdoor School; -34.4538, 148.959; 27 Nov.–05 Feb. 2018; Evangelista & Rodriquez leg.; Malaise trap; Bush Blitz; BOLD Process ID: AUMIC1170-24; ANIC: 32-085550. • ♀; NSW, Bendemeer; -30.819, 151.142; 9–23 Feb. 2020; A. Goodwin & R. Noakes leg.; Malaise trap; BOLD Process ID: AUMIC1006-24; ANIC: 32-085564. • ♀; NSW, Yanununbeyan; Apple Box Flat; Woolcara Ln; -35.5478, 149.352; 6 Nov.–03 Dec. 2020; KM Bayless, T Wallenius leg.; Malaise trap over seasonal stream; BOLD Process ID: AUMIC844-23; ANIC: 32-085551. • ♀; NT, Casurina coastal Reserve Darwin; -12.3617, 130.868; 14 Jun. 2005; Austin/PCD leg.; Sweeping; BOLD Process ID: AUMIC111-18; ANIC: 32-085565. • ♂; QLD, Cape Melville NP, Bathurst Bay, creek upstream from Nookai day-use area; -14.3994, 144.458; 7–11 Nov. 2019; C. J. Burwell leg.; Malaise trap; BOLD Process ID: AUMIC976-24; QM: T261178. • ♂; QLD, as previous except: BOLD Process ID: AUMIC978-24; QM: T261179. • ♀; QLD, Cape York, Steve Irwin Reserve, Bluebottle Ck.; -12.3589, 142.237; 24–26 Jul. 2018; C. Lambkin leg.; White pan trap; BOLD Process ID: AUMIC981-24; QM: T261180. • ♀; QLD, Cliento Conservation Reserve Nambour; -26.6146, 152.953; 02 Oct. 2020; E. Fagan-Jeffries leg.; Sweeping; BOLD Process ID: OZBOL410-21; QM: T261255. • ♀; QLD, Cooktown, Grassy hill lookout; -15.4604, 145.254; 18 Nov. 2019; E. Fagan-Jeffries & J. B. Dorey leg.; Sweeping vegetation; BOLD Process ID: AUMIC1224-24; QM: T261256. • ♀; QLD, Cow Bay, Cape Tribulation, Daintree Discovery Centre; -16.2382, 145.427; 13 Jun. 2015; C.J. Bennett leg.; Malaise trap; BOLD Process ID: GMQQT456-18; QM: T261273. • ♀; QLD, Cowra; -33.8345, 148.683; 12 Dec. 2019; J. B. Dorey leg.; General sweep over tall ?*Angophora* sp. in park right next to river.; Some other floral resources available nearby, *Eucalyptus* on far side of river. Sunny, warm and quite smokey 30C; BOLD Process ID: AUMIC1120-24; QM: T261257. • ♀; QLD, Kuranda; -16.8135, 145.643; 8 Jan.–11 Feb. 2020; M. S. Moulds leg.; Malaise trap; BOLD Process ID: AUMIC1194-24; QM: T261258. • ♀; QLD, Laura; -15.5594, 144.445; 15 Nov. 2019; J. B. Dorey leg.; General sweep off tree near end of flowering in township.; Hot and sunny ~ 32C; BOLD Process ID: AUMIC1184-24; QM: T261259. • ♀; QLD, Noosa National Park; -26.4008, 153.106; 02 Oct. 2020; E. Fagan-Jeffries leg.; Sweeping; BOLD Process ID: OZBOL420-21; QM: T261260. • ♂; as previous except: BOLD Process ID: OZBOL421-21; QM: T261261. • ♀; QLD, Plevna Downs, Tompilly Hill base (PD6 M); -26.728, 142.651; 16 Sep.–2 Oct. 2008; Lambkin Mackenzie Starick leg.; Malaise trap; Gidgee; BOLD Process ID: AUMIC114-18; QM: T208346. • ♀; QLD, Stanthorpe, Mt Marlay Lookout; -28.6537, 151.946; 27 Nov. 2019; E. Fagan-Jeffries, J. B. Dorey & P. Ruhr leg.; Sweeping vegetation; BOLD Process ID: AUMIC1206-24; QM: T261262. • ♀; QLD, Townsville, Hermit Park; -19.2829, 146.801; 01 Jan. 2017; Graeme Cocks leg.; light trap; BOLD Process ID: GCQT064-17; QM: T261274. • ♀; QLD, Wide water reserve bush camp near Taroom; -25.6262, 149.794; 25 Nov. 2019; E. Fagan-Jeffries, J. B. Dorey & P. Ruhr leg.; Sweeping vegetation; BOLD Process ID: AUMIC1068-24; QM: T261263. • ♂; SA, Adelaide, Sheidow Park, Hamilton Court; -35.0774, 138.536; 31 Mar. 2020; E. Fagan-Jeffries & R. Ellinger leg.; Sweeping; BOLD Process ID: AUMIC966-24; SAMA: 32-47824. • ♀; SA, Belair N.P. Gate 11; -35.009, 138.654; 11–24 Nov. 2007; J.T. Jennings leg.; Malaise trap; BOLD Process ID: AUMIC206-18; SAMA: 32-47825. • ♀; as previous except: 08–30 Mar. 2008; BOLD Process ID: AUMIC209-18; SAMA: 32-47826. • ♀; as previous except: BOLD Process ID: AUMIC211-18; SAMA: 32-47827. • ♀; as previous except: BOLD Process ID: AUMIC215-18; SAMA: 32-47828. • ♀; as previous except: BOLD Process ID: AUMIC426-18; SAMA: 32-47836. • ♀; as previous except: BOLD Process ID: AUMIC216-18; SAMA: 32-47829. • ♀; as previous except: BOLD Process ID: AUMIC218-18; SAMA: 32-47830. • ♀; as previous except: BOLD Process ID: AUMIC263-18; SAMA: 32-47831. • ♀; as previous except: BOLD Process ID: AUMIC265-18; SAMA: 32-47832. • ♀; as previous except: BOLD Process ID: AUMIC260-18; SAMA: 32-47840. • ♀; as previous except: 1–8 Mar. 2008; BOLD Process ID: AUMIC266-18; SAMA: 32-47833. • ♀; as previous except: BOLD Process ID: AUMIC267-18; SAMA: 32-47834. • ♀; as previous except: BOLD Process ID: AUMIC268-18; SAMA: 32-47835. • ♀; as previous except: BOLD Process ID: AUMIC227-18; SAMA: 32-47838. • ♀; as previous except: 25 Nov.–1 Dec. 2007; BOLD Process ID: AUMIC461-18; SAMA: 32-47837. • ♀; as previous except: 2–5 Dec. 2006; BOLD Process ID: AUMIC351-18; SAMA: 32-47839. • ♂; SA, Calperum station homestead; -34.0428, 140.712; 14 Apr. 2021; B.A. Parslow, A.J. Bird leg.; Light sheet (LepiLED); BOLD Process ID: AUMIC867-23; SAMA: 32-47770. • ♂; as previous except; BOLD Process ID: AUMIC869-23; SAMA: 32-47771. • ♀; SA, Cox Scrub Con. Pk.; -35.3311, 138.747; 27 Dec. 2003–17 Jan. 2004; A. Austin leg.; Malaise trap; BOLD Process ID: AUMIC283-18; SAMA: 32-47841. • ♂; SA, Hiltaba Shearers Quarters; -32.1608, 135.092; 27 Sep. 2021; E. Fagan-Jeffries leg.; Methane vapour light; BOLD Process ID: AUMIC697-23; WINC: 32-47842. • ♀; SA, Kangaroo Island, Lot 51 HA just near end of driveway near 3^rd^ power pole; -35.9038, 137.551; 20–26 Mar. 2021; R. Glatz leg.; Malaise trap; BOLD Process ID: AUMIC921-23; SAMA: 32-47764. • ♀; SA, Mt Barker, 8 km S Bugle Ranges; -35.113, 138.871; 31 Mar.–7 Apr. 2008; R. Lavigne leg.; Mallee Scrub; BOLD Process ID: AUMIC009-18; SAMA: 32-47843. • ♀; SA, nr Wistow; -35.115, 138.912; 01.–08 Oct. 2001; Malaise Trap; BOLD Process ID: AUMIC270-18; SAMA: 32-47844. • ♀; WA, Albany Highway. Gleneagle State Forest.; -32.2711, 116.163; 03 Apr.–7 May. 2005; M.S. Harvey leg.; Malaise trap; BOLD Process ID: AUMIC016-18; WAM: 130569. • ♀; WA, Barrow Island; -20.8647, 115.407; 6 May. 2006; S. Callan & R. Graham leg.; N05 SUC; BOLD Process ID: AUMIC032-18; WAM: 130570. • ♀; WA, Gleneagle State Forest; -32.2711, 116.163; 29 Nov. 2005; M.S. Harvey leg.; Malaise Trap; date assumed trap collection end, unknown start date; BOLD Process ID: AUMIC018-18; WAM: 130571. • ♀; as previous except: BOLD Process ID: AUMIC284-18; WAM: 130572. • ♂; WA, Kariijini NP, Weano Gorge Rd; -22.3883, 118.256; 711 m; 25 Apr.–14 May. 2003; C Lambkin, T Weir leg.; Malaise beside drying pool bank in grassland; ANIC 2054; BOLD Process ID: OZBOL472-21; ANIC: 32-085552. • ♀; WA, Karikini NP, Juna Downs Rd; -22.7394, 118.407; 19–25 Apr. 2003; C. Lambkin & T. Weir leg.; Malaise trap; BOLD Process ID: AUMIC1389-24; ANIC: 32-085553. • ♂; as previous except: BOLD Process ID: AUMIC1388-24; WAM: 32-085554.

###### Diagnostic description.

***Size***: Total body length: 2.6 mm; fore wing length: 2.3 mm. ***Head***: anterior scape colour similar or only very slightly paler than head colour; F2L/W ratio: 2.6; F14L/W ratio: 1.2. ***Mesosoma***: scutoscutellar sulcus with ten pits; mesoscutellar disc mostly smooth, or with very shallow scattered indentations; propodeal areola complete, or mostly so; propodeum mostly smooth posteriorly, mostly rugose anteriorly; coxae colour (pro, meso, meta): dark all; metafemur colour mostly dark. ***Wings***: centre of pterostigma pigmented to same degree as the outer edges; fore wing r vein length/2RS vein length ratio: 1.5. Some infuscation present on the fore wing membrane (not present on all specimens). ***Metasoma***: T1 shape mostly parallel, T1 medial length/anterior width between 1–2 × longer than wide; mostly rugose; T2 mostly smooth; ovipositor sheath length/metatibia length ratio: 0.7 (0.7–1.2 in paratypes).

*Apantelesmagicus* can be separated from most of the other species of *Apanteles* in Australia by the metafemur and metacoxa dark in colouration, the ovipositor sheath length > 0.6 × metatibia length, the antenna similar length to the body length, the pterostigma not paler in the centre, the metatibia mostly dark, and T1 with strong rugose sculpturing, and infuscation on the fore wing restricted to the area around r and 1CUb veins. *Apantelesmagicus* can be difficult to morphologically diagnose against *A.hades* and *A.alatomicans*, and some specimens lack the infuscation in the fore wing. This species is morphologically not particularly distinct, and ideally identification should be confirmed with molecular data.

###### Etymology.

This species name is inspired by EPFJ’s husband, Rob Ellinger, who helped collect one of the specimens during a suburban walk during COVID lockdowns in South Australia. The epithet *magicus* is the Latin adjective for magical and relates to R. Ellinger’s profession as a magician. The name is also fitting for this species because of its seemingly magical distribution and common collection yet complete lack of reared records and host data... and perhaps, like a magic trick, the species is not all that it seems as suggested by the complex molecular delimitation and potential for cryptic species.

###### Distribution.

*Apantelesmagicus* is a very broadly distributed species throughout Australia that appears to be found in an incredibly wide range of habitats.

###### Molecular information.

The specimens assigned to *Apantelesmagicus* are currently spread across five BINs on BOLD: BOLD:ADG0556, BOLD:ADY2399, BOLD:ADW8360, BOLD:ADL5561, BOLD:ADG0556, and BOLD:ADL5484. Despite this, there is only a maximum of 2.6% divergence among the COI barcodes of the species, and > 6% divergence to any other species included in the analysis. There are three *wg* haplotypes currently assigned to the species, up to 3 bp difference among them.

###### Remarks.

This species appears to be highly variable, with multiple COI and *wg* haplotypes and variation amongst paratypes in morphological characters such as the wing infuscation and hind tibia colouration. It is also one of the most commonly collected and widely distributed species of *Apanteles* in Australia, and would be a good candidate for intensive research to establish the species boundaries more definitively.

##### ﻿﻿ Apanteles margaritarius

Taxon classificationAnimaliaHymenopteraBraconidae

Slater-Baker, Fagan-Jeffries, Fernández-Triana, Portmann & Oestmann sp. nov.

ABCD43B7-3B70-5644-8D87-8092E46850F6

https://zoobank.org/76E36B28-8CB6-4753-8809-E3CEEC9DB092

**Figure 49.**
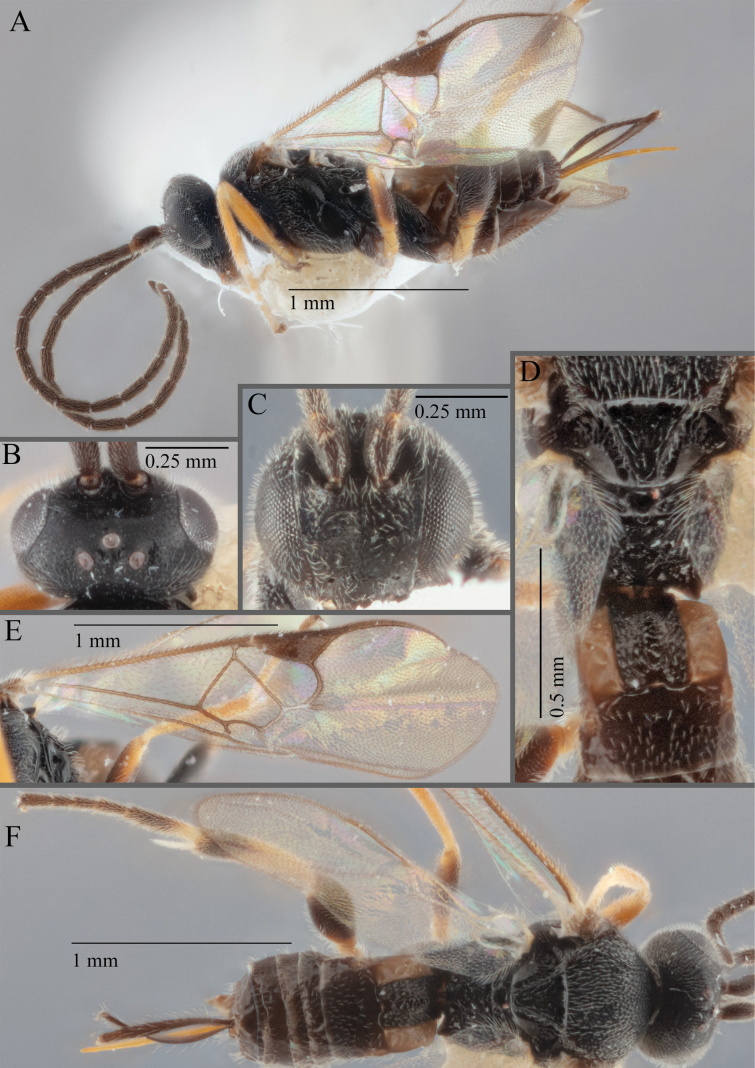
*Apantelesmargaritarius***A** lateral habitus (paratype AUMIC282-18) **B** dorsal head (paratype AUMIC282-18) **C** anterior head (holotype AUMIC1148-24) **D** dorsal propodeum and T1–3 (paratype AUMIC282-18) **E** fore wing (paratype AUMIC282-18) **F** dorsal habitus (paratype AUMIC282-18).

Fig. 5C (distribution), Fig. 49 (paratype)

###### Type material.

***Holotype*.** Australia • ♀; ACT, Canberra, CSIRO Black Mountain, close to Botanic Garden fence; -35.2736, 149.102; 538 m; 31 Jan.–6 Feb. 2018; T. Pleines & J. Rodriguez leg.; Malaise trap; BOLD Process ID: AUMIC1148-24; ANIC: 32-085527. ***Paratypes*.** Australia • ♀; NSW, near Crommellin Biological Field Stn.; -33.5518, 151.299; 18 Feb. 2008; A.D. Austin leg.; Sweeping; BOLD Process ID: AUMIC282-18; ANIC: 32-085556. • ♂; QLD, Eungella National Park, Sky Window; -21.1453, 148.499; 768 m; 23 Nov. 2019; E. Fagan-Jeffries, J. B. Dorey & P. Ruhr leg.; Sweeping vegetation; BOLD Process ID: AUMIC1239-24; ANIC: 32-085557.

###### Diagnostic description.

***Size***: Total body length: 2.0 mm; fore wing length: 2.1 mm. ***Head***: anterior scape colour similar or only very slightly paler than head colour; F2L/W ratio: 3.1; F14L/W ratio: 1.6. ***Mesosoma***: scutoscutellar sulcus with nine pits; mesoscutellar disc mostly smooth, or with very shallow scattered indentations; propodeal areola complete, or mostly so; propodeum mostly smooth posteriorly, mostly rugose anteriorly; coxae colour (pro, meso, meta): dark all; metafemur colour mostly dark. ***Wings***: centre of pterostigma pigmented to same degree as the outer edges; fore wing r vein length/2RS vein length ratio: 1.1. ***Metasoma***: T1 shape mostly parallel, T1 medial length/anterior width between 1–2 × longer than wide; mostly rugose; T2 mostly smooth; ovipositor sheath length/metatibia length ratio: 0.9.

*Apantelesmargaritarius* can be separated from the other described species of *Apanteles* known from Australia with a dark metacoxa by the metatrochanter pale, the metafemur mostly dark with small pale area proximally, T2 smooth, anterior side of scape same or only very slightly paler than head colour, and T1 parallel sided, not strongly narrowing posteriorly.

###### Etymology.

The species epithet is a Latin adjective meaning ‘of/related to pearl’ and connects to the collection locality of one of the paratypes, near Pearl Caves.

###### Distribution.

*Apantelesmargaritarius* is currently known from three collection localities in eastern Australia.

###### Molecular information.

Sequences of *Apantelesmargaritarius* are currently in BIN BOLD:ADL4093. The COI sequences are at least 8% divergent from any of the other species treated here, or any available sequence on BOLD. The *wg* sequence of the holotype is at least 5 bp different to any other species. Most delimitation methods resolved *A.margaritarius* as a discrete species, however and *wg*PTP grouped the species with *A.kelpiellus*.

##### ﻿﻿ Apanteles oenone

Taxon classificationAnimaliaHymenopteraBraconidae

Nixon, 1965

58FB8E41-D6C2-5219-B0A2-9F697524043B

**Figure 50.**
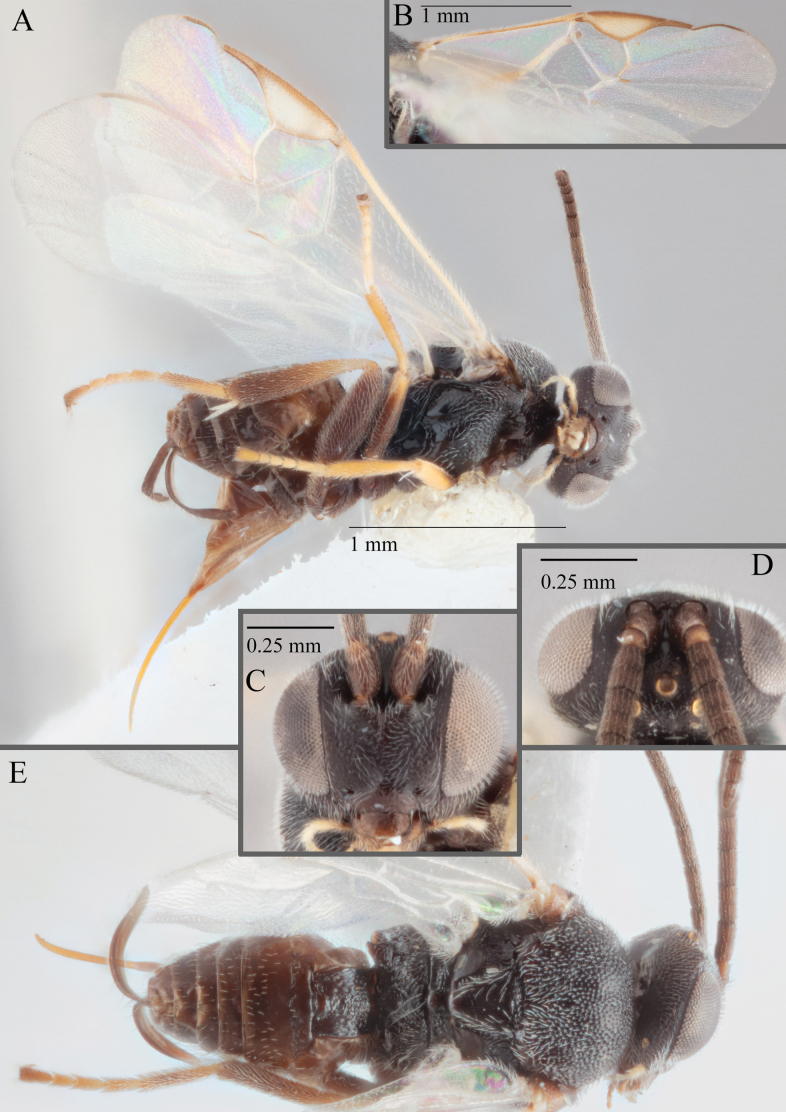
*Apantelesoenone* (AUMIC036-18) **A** lateral habitus **B** fore wing **C** anterior head **D** dorsal head **E** dorsal habitus.

**Figure 51.**
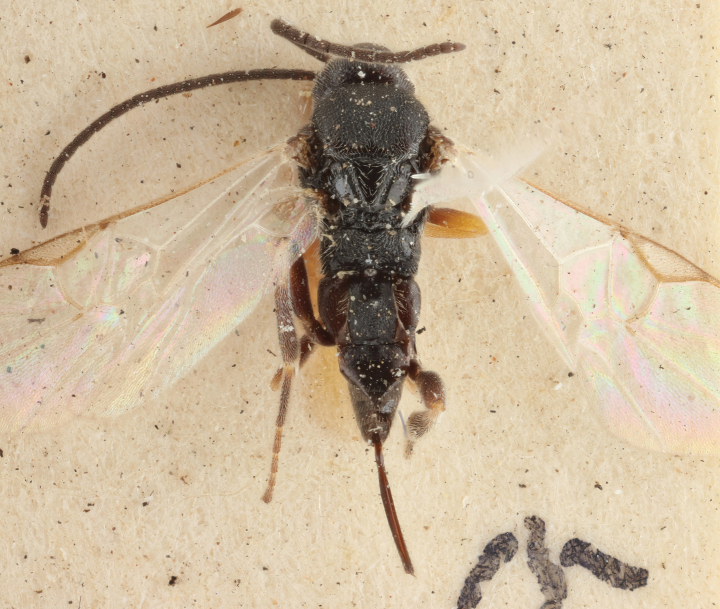
*Apantelesoenone* holotype.

Fig. 6B (distribution), Fig. 50 (examined material), Fig. 51 (holotype)

###### Holotype information.

♀; Australia, QLD, Ayr, 15 Jun. 1949, bred from “*Eariashuegeli* (W.A.S.)” [syn. *Eariasvittella* (Fabricius, 1794)] (NHM).

###### Examined material.

Australia • ♀; NT, 18 km W of Gemtree Caravan Park; -22.9651, 134.069; 01 Oct. 2018; M.M. Giannotta leg.; Sweeping; Collected from Capparis spinosa; BOLD Process ID: AUMIC862-23; ANIC: 32-085558. • ♀; NT, Gregory NP, 17.4 km N Humbert Junction; -15.9714, 130.488; 24 May–4 Jun. 2001; T Weir, K Pullen, P Bouchard leg.; Malaise in damp meadow; BOLD Process ID: AUMIC103-18; ANIC: 32-130203. • ♀; NT, Gregory NP, 5.7 km N Humbert Junction; -16.0622, 130.451; 6–12 Jun. 2001; ME Irwin, FD Parker, C Lambkin leg.; Malaise in dry creekbed; BOLD Process ID: AUMIC478-18; ANIC: 32-130235. • ♂; NT, Kakadu NP, Mirray Lookout; -12.8768, 132.704; 17 Nov. 1992; Austin/PCD leg.; Sweep net open acacia; BOLD Process ID: AUMIC110-18; ANIC: 32-085559. • ♂; NT, Keep River National Park, Bail-Me-Up Cr., 23.7 km SSW Jarrnarm Camp Ground.; -15.9653, 129.017; 13–20 Jun. 2001; M. E. Irwin, F. D. Parker & C. Lambkin leg.; Malaise trap; BOLD Process ID: AUMIC1412-24; ANIC: 32-085531. • ♀; as previous except: BOLD Process ID: AUMIC037-18; ANIC: 32-130185. • ♀; as previous except: BOLD Process ID: AUMIC473-18; ANIC: 32-130231. • ♀; QLD, Banana, Roadside 2 km north of Banana; -24.4524, 150.138; 25 Nov. 2019; E. Fagan-Jeffries, J. B. Dorey & P. Ruhr leg.; Sweeping vegetation, mistletoe: *Dendrophthoeglabrescens*; BOLD Process ID: AUMIC1218-24; QM: T261215. • ♂; QLD, Eungella National Park, Fern Flats Campground; -21.1697, 148.5; 22 Nov. 2019; E. Fagan-Jeffries, J. B. Dorey & P. Ruhr leg.; To light sheet with blue, green and UV LEDs; BOLD Process ID: AUMIC1212-24; QM: T261216. • ♀; QLD, Kuranda; -16.8112, 145.635; 20 Nov. 2019; J. B. Dorey leg.; Sweeping; BOLD Process ID: AUMIC1186-24; QM: T261217. • ♂; QLD, Laura; -15.5811, 144.458; 15 Mar. 2017; R. Leijs leg.; Vehicle net; vehicle netting from Laura caravan park to Quinkan Bush Blitz Site SSS2 (Welcome Rd); BOLD Process ID: AUMIC973-24; QM: T261169. • ♀; QLD, Nardoo Patch, 11 km NNE 12 Mile Bore; -23.0667, 138.192; 18–21 Apr. 2007; Malaise trap; BOLD Process ID: AUMIC034-18; ANIC: 32-130182. • ♀; QLD, Newrybar Macadamia farm; -28.7355, 153.548; 28 Nov. 2019; E. Fagan-Jeffries, J. B. Dorey & P. Ruhr leg.; Sweeping; BOLD Process ID: AUMIC1062-24; QM: T261218. • ♀; QLD, Townsville, Mt. Stuart; -19.3509, 146.801; 18 Feb. 2017; Graeme Cocks leg.; Sweeping; BOLD Process ID: GCQT689-17; CBG: gvcT09199. • ♀; QLD, Yolde Camp; -35.5452, 148.303; 12 Dec. 2019; J. B. Dorey leg.; General sweep over lots of Kunzea along roadside in schlerophyll forest.; Sunny and warm ~ 28C; BOLD Process ID: AUMIC1135-24; QM: T261219. • ♀; WA, Barrow Island; -20.7847, 115.394; 1 May. 2007; S. Callan K. Edwards leg.; N28 DHC; BOLD Process ID: AUMIC183-18; WAM: 130566. • ♀; as previous except: BOLD Process ID: AUMIC186-18; WAM: 130567. • ♀; WA, Kariijini NP, Juna Downs Rd; -22.7394, 118.413; 25 Apr.–14 May. 2003; C Lambkin T weir leg.; Malaise dry Turee creek grassy open Eucalypt scrub; BOLD Process ID: AUMIC036-18; ANIC: 32-130184. • ♂; WA, Kununurra; 11 Jun. 1975; R. A. Meedved leg.; “ex. *Pectinophoragossypiella* on Cotton, R 75-44; *Apantelesoenone*”; BOLD Process ID: HYCND1892-11; CNC: CNCHYM 00182. • ♀; USA, Riverside Co., ex. pink bollworm; 28 Jul. 1975; lab reared, “*Apantelesoenone* Nixon, det. Marsh”; BOLD Process ID: HYCND1893-11; CNC: CNCHYM 00183.

###### Diagnosis.

*Apantelesoenone* can be separated from the other species of *Apanteles* in Australia with a dark metacoxa and dark metafemur, and a pterostigma with outer border darker than centre, the centre of pterostigma pale or transparent/hyaline by the fore wing with veins M+CU, 1 cu-a, 1M, 1CUa, 1CUb, (RS+M)a, 2RS, and 1m-cu all unpigmented/pale, T1 with rugose sculpturing, and metatibia mostly dark, or at least with distal 1/2 dark in colouration.

###### Notes.

The examined material was identified through a DNA match (0–4 bp difference) to two partial barcodes on BOLD from specimens identified as *A.oenone*. The specimens, held at the CNC, were examined and the morphology aligned with the examined material and with the original description of *A.oenone*.

##### ﻿﻿ Apanteles pellucidus

Taxon classificationAnimaliaHymenopteraBraconidae

Slater-Baker, Fagan-Jeffries, Fernández-Triana, Portmann & Oestmann sp. nov.

7260D59A-87FF-52FD-8AF1-3D95F93B1ACC

https://zoobank.org/A4D210B9-6498-45C0-8582-8BC34EFA2DB4

**Figure 52.**
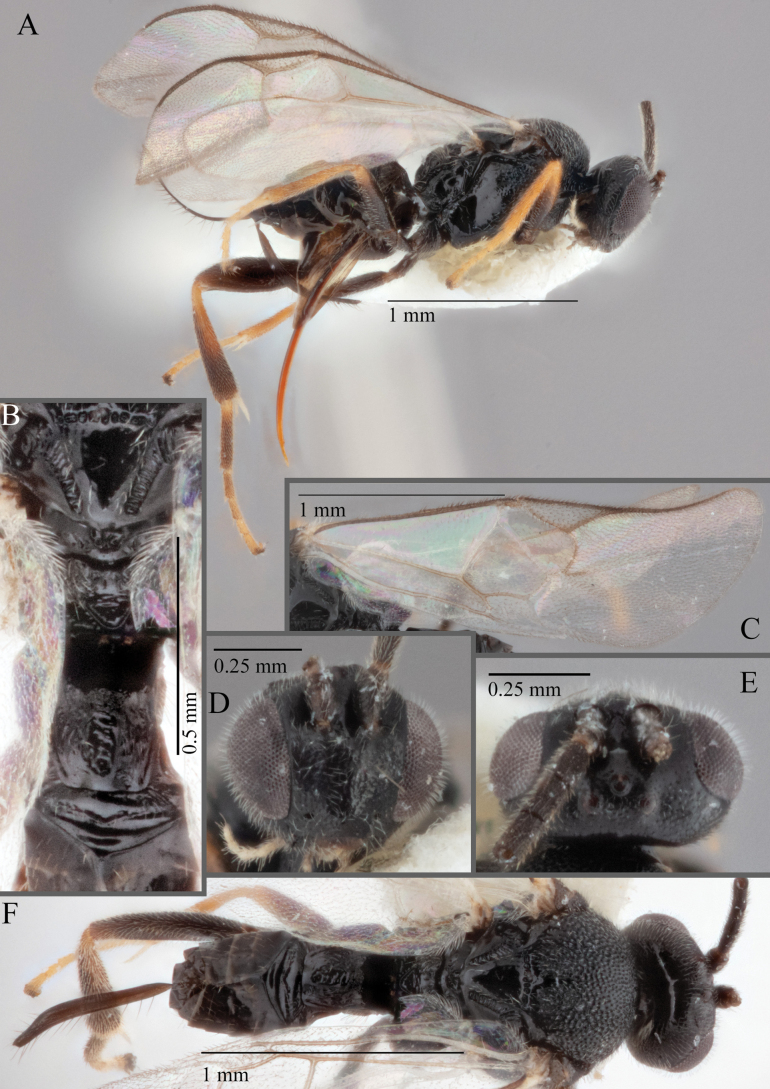
*Apantelespellucidus* (holotype AUMIC333-18) **A** lateral habitus **B** dorsal propodeum and T1–3 **C** fore wing **D** anterior head **E** dorsal head **F** dorsal habitus.

Fig. 5B (distribution), Fig. 52 (holotype)

###### Type material.

***Holotype*.** Australia • ♀; QLD, Kuranda; -16.8154, 145.643; 16 Mar. 2017; M.S. Moulds leg.; Malaise trap; BOLD Process ID: AUMIC333-18; QM: T261207.

###### Diagnostic description.

***Size***: Total body length: 2.1 mm; fore wing length: 2.3 mm. ***Head***: anterior scape colour similar or only very slightly paler than head colour; F2L/W ratio: 2.9. ***Mesosoma***: scutoscutellar sulcus with nine pits; mesoscutellar disc mostly smooth, or with very shallow scattered indentations; propodeal areola complete, or mostly so; propodeum mostly rugose; coxae colour (pro, meso, meta): dark all; metafemur colour mostly dark. ***Wings***: centre of pterostigma paler (more hyaline) than outer edges; fore wing r vein length/2RS vein length ratio: 0.6. ***Metasoma***: T1 shape mostly parallel, T1 medial length/anterior width between 1–2 × longer than wide; mostly rugose; T2 mostly smooth; ovipositor sheath length/metatibia length ratio: 0.9.

*Apantelespellucidus* can be separated from most of the other described species of *Apanteles* known from Australia with a dark metafemur and metacoxa and the centre of the pterostigma pale/hyaline by fore wing vein 1CUa pigmented whilst fore wing veins M+CU and 1M pigmented no more than half of their lengths. We do not morphologically diagnose this species against *A.rufiterra*, but as the species are not closely related based on molecular data, they can be identified through DNA barcoding and the placement of the sequences on a phylogeny in the context of the holotype barcodes.

###### Etymology.

The species epithet is a Latin adjective meaning ‘clear/transparent’ and relates to the hyaline centre of the pterostigma.

###### Distribution.

*Apantelespellucidus* is currently only known from one collection locality in northeastern QLD.

###### Molecular information.

The holotype sequence of *Apantelespellucidus* is currently in BIN BOLD:ADL5338. The COI sequence is at least 8% divergent from any of the other species treated here, or any available sequence on BOLD. The *wg* sequence of the holotype is at least 5 bp different to any other species. Most delimitation methods resolved *A.pellucidus* as a discrete species, however and *wg*ASAP grouped the species with *A.amicalis* and *A.* sp. MRSB26 (undescribed lineage).

##### ﻿﻿ Apanteles persephone

Taxon classificationAnimaliaHymenopteraBraconidae

Nixon, 1965

2D9F87A0-14F9-55C5-AB22-86852FC71A45

Fig. 6B (distribution), Fig. 37B (holotype)

###### Holotype information.

♀; Australia, WA, Yanchep, 3–19 Dec. 1935, R. E. Turner leg. (NHM).

###### Examined material.

Image of the holotype and original description used to form the diagnosis.

###### Diagnosis.

*Apantelespersephone* is a difficult species to diagnose due to us having access to only a single image of the holotype. The species can be separated from many *Apanteles* in Australia by the dark metacoxa and metafemur, antenna of similar length to the body length, uniformly coloured pterostigma, fore wing veins 1M and 1CUa of similar pigmentation, hyaline fore wing membrane, parallel-sided and rugose T1, smooth T2, smooth mesoscutellar disc, and setae reduced to a single row on each of T3–T6. *Apantelespersephone* is difficult to diagnose against *A.darthvaderi* and *A.amicalis* (see key and notes under those species for more information).

###### Notes.

Whilst we collected several species that closely resembled *A.persephone*, we hesitate to designate any of our delimited lineages as this species without either DNA of the type, or at minimum a specimen from close to the type locality (currently not available).

##### ﻿﻿ Apanteles phantasmatus

Taxon classificationAnimaliaHymenopteraBraconidae

Slater-Baker, Fagan-Jeffries, Fernández-Triana, Portmann & Oestmann sp. nov.

7C14FCB0-B7F8-5B05-A195-6F47FA3FAB48

https://zoobank.org/B931DC22-F104-4B5B-912B-19205E5BE493

**Figure 53.**
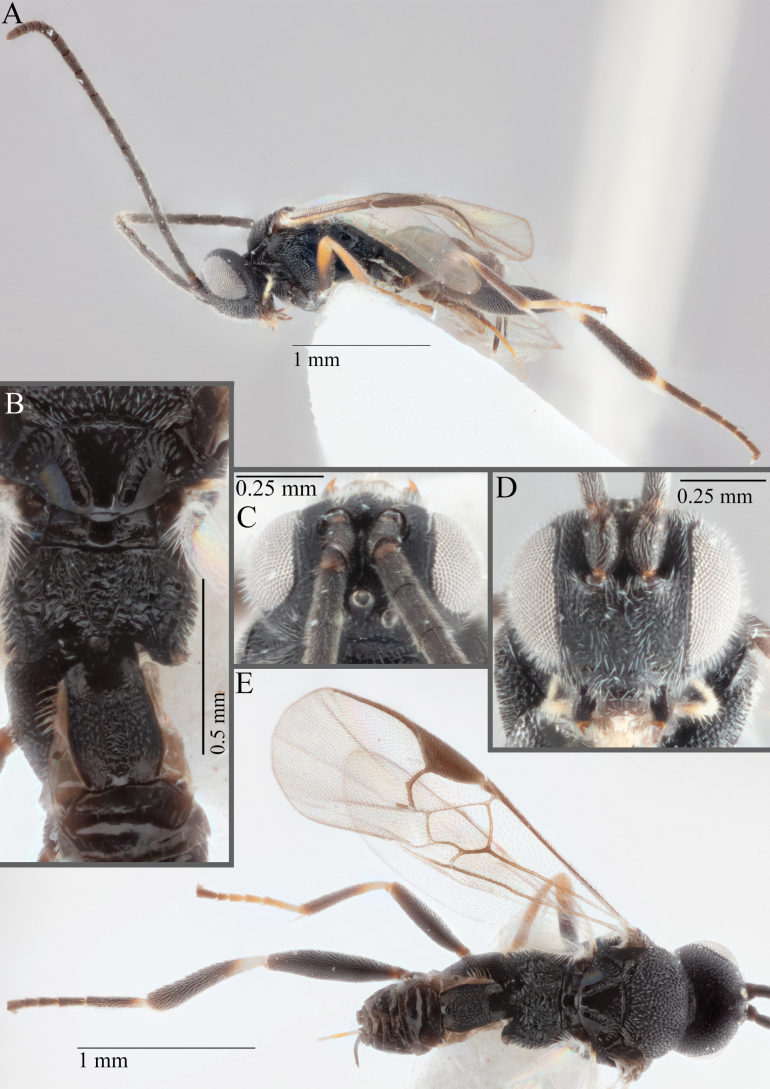
*Apantelesphantasmatus* (holotype AUMIC087-18) **A** lateral habitus **B** dorsal propodeum and T1–3 **C** dorsal head **D** anterior head **E** dorsal habitus and wings.

Fig. 5B (distribution), Fig. 53 (holotype)

###### Type material.

***Holotype*.** Australia • ♀; QLD, Samsonvale Cemetery, 8.5 km SSE Dayboro; -27.2703, 152.856; 5–22 Oct. 2014; S. Wright leg.; Malaise trap; Casuarina/open forest; BOLD Process ID: AUMIC087-18; QM: T208361. ***Paratypes*.** Australia • ♀; NSW, Hat Head; -31.063, 153.052; 26 Dec. 2009; Paul D.N. Hebert leg.; Malaise trap; BOLD Process ID: HYAS060-10; QM: T261269. • ♂; as previous except: 18 Feb. 2018; Trap #4; BOLD Process ID: NSWHO4562-18; QM: T261270. • ♀; as holotype except; 23 Sep.–05 Oct. 2014; BOLD Process ID: AUMIC071-18; QM: T208357. • ♀; as holotype except; 14 Nov.–16 Dec. 2014; S. Wright leg.; Malaise trap; BOLD Process ID: AUMIC090-18; QM: T208362. • ♀; as previous except: BOLD Process ID: AUMIC091-18; QM: T208358. • ♀; as holotype except: 22 Oct.–14 Nov. 2014; BOLD Process ID: AUMIC410-18; QM: T208363. • ♀; as holotype except: BOLD Process ID: AUMIC413-18; QM: T208359. • ♀; as holotype except: 6 Jan.–8 Feb. 2015; BOLD Process ID: AUMIC414-18; QM: T208360. • ♂; as previous except: BOLD Process ID: AUMIC1437-24; QM: T261171. • ♂; as previous except: BOLD Process ID: AUMIC1439-24; QM: T261172. • ♂; as previous except: BOLD Process ID: AUMIC1441-24; QM: T261173. • ♂; as previous except: BOLD Process ID: AUMIC1443-24; QM: T261174. • ♂; as previous except: BOLD Process ID: AUMIC1444-24; QM: T261175. • ♂; as previous except: BOLD Process ID: AUMIC1445-24; QM: T261176. • ♂; as previous except: BOLD Process ID: AUMIC1442-24; QM: T261177.

###### Diagnostic description.

***Size***: Total body length: 2.3 mm; fore wing length: 2.3 mm. ***Head***: anterior scape colour similar or only very slightly paler than head colour; F2L/W ratio: 3.1; F14L/W ratio: 1.3. ***Mesosoma***: scutoscutellar sulcus with eight pits; mesoscutellar disc mostly smooth, or with very shallow scattered indentations; propodeal areola complete, or mostly so; propodeum mostly rugose; coxae colour (pro, meso, meta): dark all; metafemur colour mostly dark. ***Wings***: centre of pterostigma pigmented to same degree as the outer edges; fore wing r vein length/2RS vein length ratio: 1.1. ***Metasoma***: T1 shape mostly parallel, T1 medial length/anterior width between 1–2 × longer than wide; mostly rugose; T2 mostly smooth; ovipositor sheath length/metatibia length ratio: 0.6.

*Apantelesphantasmatus* can be separated from the other species of *Apanteles* in Australia that have a dark metacoxa and metafemur, ovipositor sheaths > 0.5 × metatibia length, antennae of similar size or longer than the length of the body, a completely hyaline fore wing membrane, and a uniformly pigmented pterostigma (no paler centre region, and no large pale spot on proximal corner), by the metatibia mostly dark with a very pale (almost white) band discrete and restricted to proximal 1/3 of the tibia, T3 with setae not reduced to a single row (setae more irregularly arranged), and T2 posterior width/medial length ratio ~ 4.1). The species could be confused with the individuals of *A.magicus* that lack fore wing infuscation, but the DNA barcodes of the two species cluster discretely in a phylogeny.

###### Etymology.

The species epithet is an adjective formed from the Latin noun *phantasma* meaning ghost and relates to the collection of most of the specimens from a cemetery.

###### Distribution.

*Apantelesphantasmatus* is currently known from the east coast of Australia.

###### Molecular information.

Sequences of *Apantelesphantasmatus* are currently in BIN BOLD:AAU8268. The COI sequences are at least 9% divergent from any of the other species treated here, or any available sequence on BOLD. The *wg* sequences of the species are at least 8 bp different to any other species. All delimitation methods resolved *A.phantasmatus* as a discrete species.

##### ﻿﻿ Apanteles pharusalis

Taxon classificationAnimaliaHymenopteraBraconidae

Slater-Baker, Fagan-Jeffries, Fernández-Triana, Portmann & Oestmann sp. nov.

8E7D7112-E20F-5482-ABC4-E27FDEDCA848

https://zoobank.org/DBCF9EDA-70C4-4DFC-A46F-B32AF3EAA4F7

**Figure 54.**
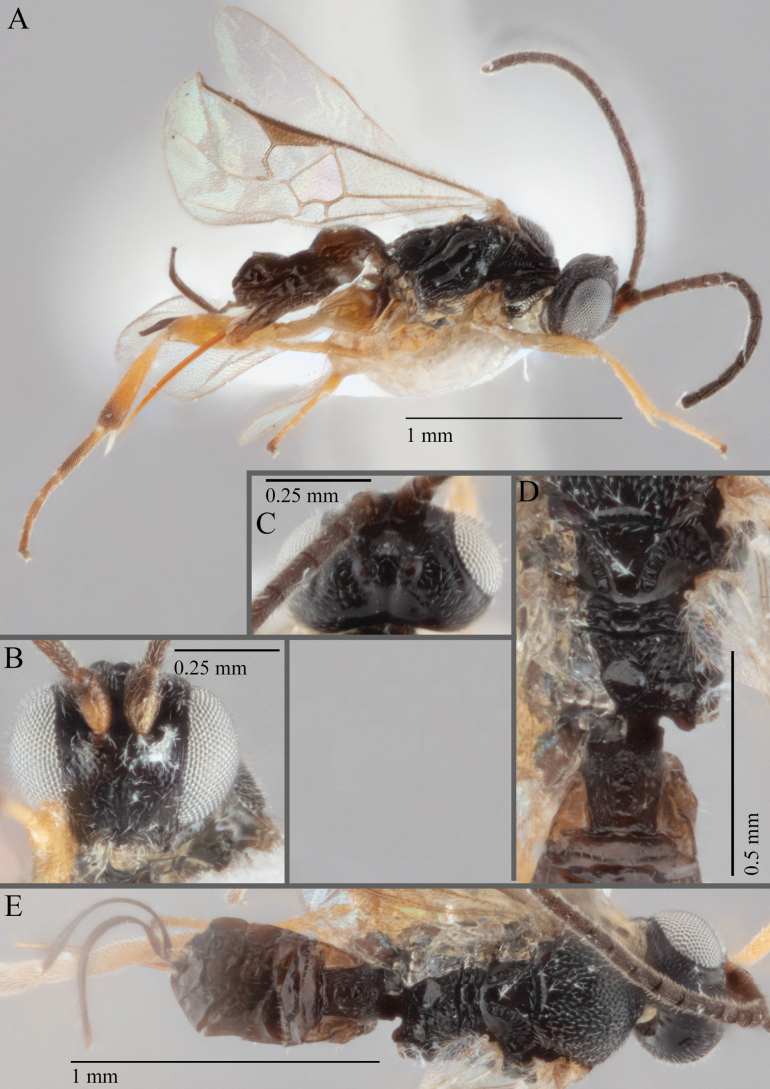
*Apantelespharusalis* (holotype AUMIC1223-24) **A** lateral habitus **B** anterior head **C** dorsal head **D** dorsal propodeum and T1, T2**E** dorsal habitus.

Fig. 5C (distribution), Fig. 54 (holotype)

###### Type material.

***Holotype*.** Australia • ♀; QLD, Cooktown, Grassy hill lookout; -15.4604, 145.254; 140 m; 18 Nov. 2019; E. Fagan-Jeffries & J. B. Dorey leg.; Sweeping vegetation; BOLD Process ID: AUMIC1223-24; QM: T261202. ***Paratypes*.** Australia • ♂; QLD, Townsville, Mt. Stuart; -19.3509, 146.801; 400 m; 04 Jul. 2017; Graeme Cocks leg.; netted; BOLD Process ID: GCQT1820-17; QM: T261268. • ♀; WA, Northwest Kimberly, Dambimangari Spatial block 2, M26/1R3se; -15.4011, 124.649; 28 Jan. 2013; OR Edwards & RK Didham CSIRO leg.; Malaise trap sample (7 days); BOLD Process ID: AUMIC056-18; WAM: 130554.

###### Diagnostic description.

***Size***: Total body length: 1.9 mm; fore wing length: 1.7 mm. ***Head***: anterior scape colour much paler, dramatically different colour than head; F2L/W ratio: 3.0; F14L/W ratio: 1.3. ***Mesosoma***: scutoscutellar sulcus with nine pits; mesoscutellar disc mostly smooth, or with very shallow scattered indentations; propodeal areola complete, or mostly so; propodeum mostly smooth posteriorly, mostly rugose anteriorly; coxae colour (pro, meso, meta): all pale; metafemur colour mostly pale. ***Wings***: centre of pterostigma pigmented to same degree as the outer edges; fore wing r vein length/2RS vein length ratio: 1.5. ***Metasoma***: T1 shape mostly parallel, T1 medial length/anterior width between 1–2 × longer than wide; T1 mostly rugose; T2 with fine sculpture; ovipositor sheath length/metatibia length ratio: 1.0.

*Apantelespharusalis* can be separated from all other described *Apanteles* species in Australia other than *A.doreenwatlerae* by the pale metacoxa. It can be diagnosed against *A.doreenwatlerae* by the T1 anterior width/posterior width ratio (~ 1.2 in *A.pharusalis* and ~ 1.7 in *A.doreenwatlerae*).

###### Etymology.

This species is named for the lighthouse present at the collection locality of the holotype. The species epithet is an adjective formed from the Latin *pharus*, which comes from Pharos, an island near Alexandria where there was a famous lighthouse.

###### Distribution.

*Apantelespharusalis* is currently known from northern Australia, from sites on both the east and west coast.

###### Molecular information.

Sequences of *Apantelespharusalis* are currently in BIN BOLD:ADL3000. The COI sequences are at least 9% divergent from any of the other species treated here, or any available sequence on BOLD. The *wg* sequences of the holotype is at least 7 bp different to any other species. All delimitation methods resolved *A.pharusalis* as a discrete species.

##### ﻿﻿ Apanteles ramsaris

Taxon classificationAnimaliaHymenopteraBraconidae

Slater-Baker, Fagan-Jeffries, Fernández-Triana, Portmann & Oestmann sp. nov.

3BC20971-36A5-56EC-954D-70ECB2685EC3

https://zoobank.org/790D8547-2D60-4F77-8DB8-02578534D916

**Figure 55.**
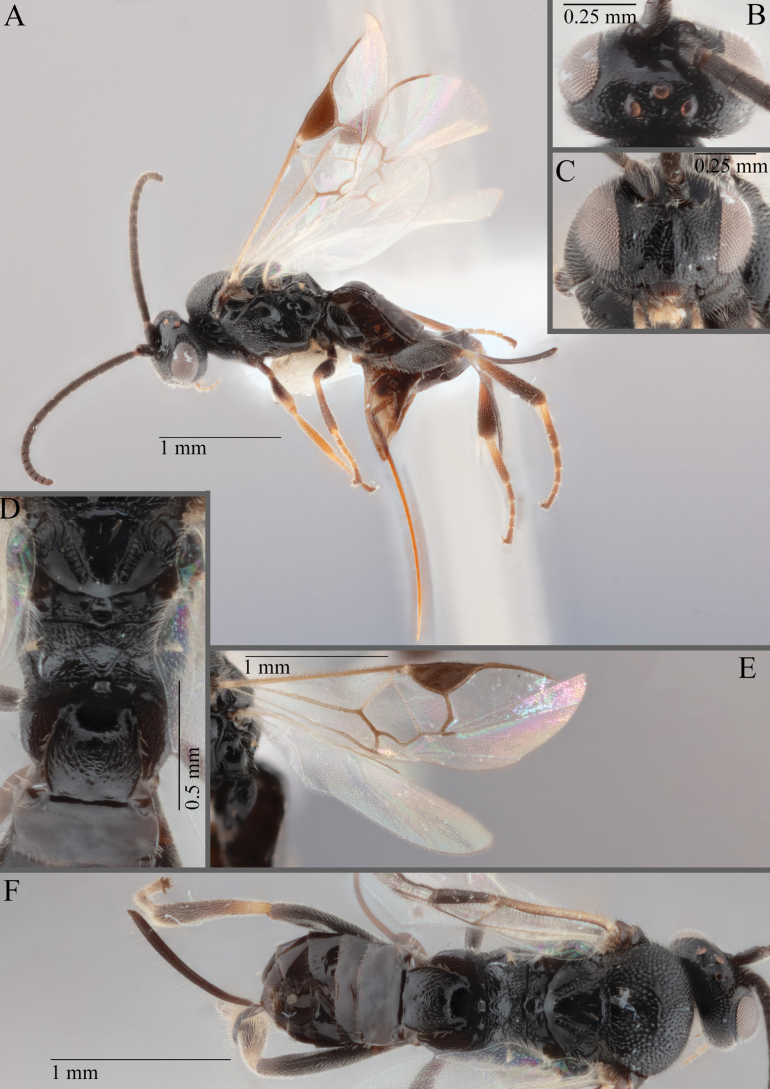
*Apantelesramsaris* (holotype AUMIC651-23). **A** lateral habitus **B** dorsal head **C** anterior head **D** dorsal propodeum and T1–3 **E** fore and hind wing **F** dorsal habitus.

Fig. 5B (distribution), Fig. 55 (holotype)

###### Type material.

***Holotype*.** Australia • ♀; SA, Banrock site B; -34.1708, 140.319; 12–14 Dec. 2019; R. Glatz leg.; Malaise trap; BOLD Process ID: AUMIC651-23; SAMA: 32-47762.

###### Diagnostic description.

***Size***: Total body length: 2.1 mm; fore wing length: 2.6 mm. ***Head***: antennae slightly shorter than body length; anterior scape colour similar or only very slightly paler than head colour; F2L/W ratio: 2.6; F14L/W ratio: 1.0. ***Mesosoma***: scutoscutellar sulcus with nine pits; mesoscutellar disc mostly smooth, or with very shallow scattered indentations; propodeal areola complete, or mostly so; propodeum mostly smooth posteriorly, mostly rugose anteriorly; coxae colour (pro, meso, meta): all dark; metafemur colour mostly dark. ***Wings***: centre of pterostigma pigmented to same degree as the outer edges; fore wing r vein length/2RS vein length ratio: 1.2. ***Metasoma***: T1 shape almost barrel shaped, very curved on lateral margins; T1 medial length/anterior width between 1–2 × longer than wide; mostly rugose distal 1/2, mostly smooth basal 1/2; T2 mostly smooth; ovipositor sheath length/metatibia length ratio: 1.2.

*Apantelesramsaris* can be separated from most other described species of *Apanteles* in Australia that have a dark metacoxa and metafemur and the pterostigma without a pale centre, the ovipositor sheath length > 0.6 × metatibia length by the pterostigma having a small pale spot proximally, the metatibia displaying a gradient of colouration from pale to dark, the colours merging in the centre, T1 with strong sculpture over at least most of posterior 1/2 of tergite, mesoscutellar disc with at most scattered punctures along margins, and fore wing vein 1M much less pigmented (often transparent/pale) compared to pigmentation of vein 1CUa. Compared to *A.brockhedgesi*, *A.ramsaris* has the scutoscutellar sulcus comparatively wider and with comparatively larger pits. The species can currently be best separated from *A.insulanus* by DNA barcoding, and placement of the unknown sequence in the context of a phylogeny with the holotype barcodes of both species (which are > 7% divergent).

###### Etymology.

The species epithet is an adjective formed from the term ‘Ramsar’ relating to the Ramsar accredited wetlands at Banrock Station, the collection locality.

###### Distribution.

*Apantelesramsaris* is currently only known from the type locality in the Riverland region of SA.

###### Molecular information.

The holotype of *Apantelesramsaris* is the only sequence in BIN BOLD:AFE7524. The COI sequences are at least 7% divergent from any of the other species treated here, or any available sequence on BOLD. The *wg* sequence of the holotype is at least 2 bp different to any other species. Most delimitation methods resolved *A.ramsaris* as a discrete species, except for *wg*ASAP and PTP, which grouped the species with *A.insulanus*.

##### ﻿﻿ Apanteles rufiterra

Taxon classificationAnimaliaHymenopteraBraconidae

Slater-Baker, Fagan-Jeffries, Fernández-Triana, Portmann & Oestmann sp. nov.

038F20D4-E03F-549C-96B7-49FB9DAEAC5F

https://zoobank.org/97546163-7F25-45B7-AAE9-AD69FD2C93FD

**Figure 56.**
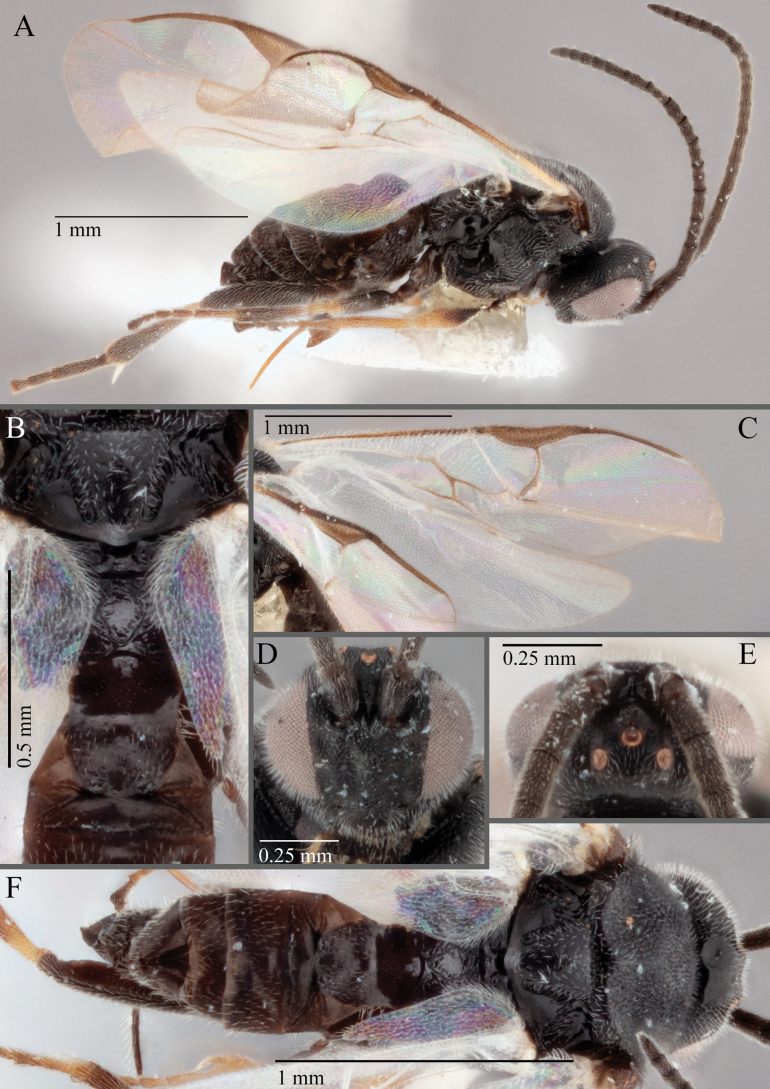
*Apantelesrufiterra* (holotype AUMIC1351-24) **A** lateral habitus **B** dorsal propodeum and T1–3 **C** fore and hindwing **D** anterior head **E** dorsal head **F** dorsal habitus.

Fig. 5C (distribution), Fig. 56 (holotype)

###### Type material.

***Holotype*.** Australia • ♀; WA, Western Pilbara, Hamersley station, Nanutarra-Wittenoom Rd., approx. 25 km NE of Railway Rd. crossing, North side of hill on East ridge.; -22.3558, 117.915; 15–19 May. 2006; Conservation Volunteers Australia leg.; Malaise trap; BOLD Process ID: AUMIC1351-24; WAM: 130555. ***Paratypes*.** Australia • ♀; QLD, 3.6 km NW Homestead on Plum Pudding Track, Cravens Peak Station; -23.3128, 138.562; 21–24 Apr. 2007; C Lemann leg.; Malaise trap; Spinifex; BOLD Process ID: AUMIC049-18; ANIC: 32-130195. • ♂; SA, Witjira NP, Purni Bore, 88 km EbS Mt Dare Hotel; -26.2847, 136.085; 19–22 Mar. 2017; D. Yeates, A. Landford, Y. Su, X. Li, J. Lumbers & M. Irwin leg.; Malaise trap; BOLD Process ID: AUMIC1047-24; WAM: 130556. • ♀; WA, Karikini NP, Juna Downs Rd; -22.7394, 118.407; 19–25 Apr. 2003; C. Lambkin & T. Weir leg.; Malaise trap; BOLD Process ID: AUMIC1386-24; WAM: 130557. • ♀; WA, Watheroo NP, Jingemia Caves; -30.2542, 115.999; 273 m; 17 Sep.–7 Nov. 2003; C Lambkin, N Starick, J Recsei leg.; Malaise closed heath; BOLD Process ID: AUMIC448-18; ANIC: 32-130210. • ♀; WA, Western Pilbara, Hamersley station, Nanutarra-Wittenoom Rd., approx. 10 km NE of Railway Rd. crossing, nr railway crossing; -22.4433, 117.813; 22–27 Sep. 2005; Conservation Volunteers Australia leg.; Malaise trap; BOLD Process ID: AUMIC1316-24; AM: K.247579. • ♂; WA, Western Pilbara, Hamersley station, Nanutarra-Wittenoom Rd., approx. 13 km NE of Railway Rd. crossing, near fence line; -22.4356, 117.83; 27–30 May. 2004; A. Donnelly & G. Carter leg.; Malaise trap; BOLD Process ID: AUMIC1350-24; WAM: 130558.

###### Diagnostic description.

***Size***: Total body length: 2.5 mm; fore wing length: 2.4 mm. ***Head***: anterior scape colour similar or only very slightly paler than head colour; F2L/W ratio: 2.8; F14L/W ratio: 1.4. ***Mesosoma***: scutoscutellar sulcus with 12 pits; mesoscutellar disc mostly smooth, or with very shallow scattered indentations; propodeal areola complete, or mostly so; propodeum mostly rugose; coxae colour (pro, meso, meta): dark all; metafemur colour mostly dark. ***Wings***: centre of pterostigma pigmented to same degree as the outer edges; fore wing r vein length/2RS vein length ratio: 1.9. ***Metasoma***: T1 shape narrowing in posterior third, T1 medial length/anterior width between 1–2 × longer than wide; mostly smooth; T2 mostly smooth; ovipositor sheath length/metatibia length ratio: 0.6.

*Apantelesrufiterra* can be separated from most of the other described species of *Apanteles* known from Australia with a dark metafemur and metacoxa and the centre of the pterostigma pale/hyaline by fore wing vein 1CUa pigmented whilst fore wing veins M+CU and 1M pigmented no more than half of their lengths. We do not morphologically diagnose this species against *A.pellucidus*, but as the species are not closely related based on molecular data, they can be identified through DNA barcoding and the placement of the sequences on a phylogeny in the context of the holotype barcodes.

###### Etymology.

The species epithet is roughly translated as ‘red earth/dirt’ from the Latin *rufus* (reddish) and *terra* (earth/soil) and relates to the beautiful red earth of many of the collection localities of this species in outback Australia. The epithet is a noun in apposition.

###### Distribution.

*Apantelesrufiterra* is found throughout the central latitudes of Australia, in both SA and WA.

###### Molecular information.

Sequences of *Apantelesrufiterra* are currently in BIN BOLD:ADL3292. The COI sequences are at least 8% divergent from any of the other species treated here, or any available sequence on BOLD. The *wg* sequences of the species are at least 19 bp different to any other species. All delimitation methods resolved *A.rufiterra* as a discrete species.

##### ﻿﻿ Apanteles sinusulus

Taxon classificationAnimaliaHymenopteraBraconidae

Slater-Baker, Fagan-Jeffries, Fernández-Triana, Portmann & Oestmann sp. nov.

F23523C0-D493-51BA-843B-FC7F81EECE5D

https://zoobank.org/D4A177E6-018B-4AC7-859E-4F6AE5E7CC33

**Figure 57.**
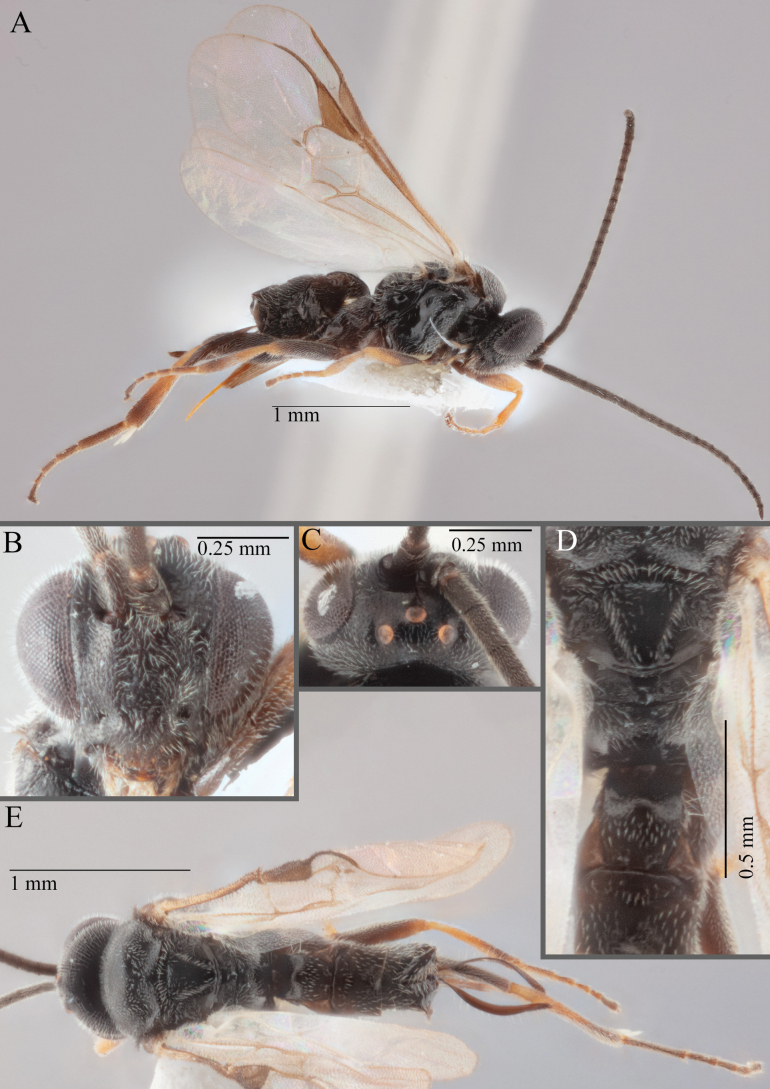
*Apantelessinusulus* (holotype AUMIC1313-24) **A** lateral habitus **B** anterior head **C** dorsal head **D** dorsal propodeum and T1–T3 **E** lateral habitus and wings.

Fig. 6A (distribution), Fig. 57 (holotype)

###### Type material.

***Holotype*.** Australia • ♀; WA, Western Pilbara, Hamersley station, Nanutarra-Wittenoom Rd., approx. 25 km NE of Railway Rd. crossing, North side of hill on East ridge; -22.3558, 117.915; 15–19 May. 2006; Conservation Volunteers Australia leg.; Malaise trap; BOLD Process ID: AUMIC1313-24; WAM: 130561. ***Paratypes*.** • 1? (missing metasoma); NSW, Canterbury-Bankstown region, Little Salt Pan Creek; -33.9606, 151.027; 08 Feb. 2006; K. Harvey & J. Lee leg.; vacuum sampler; BOLD Process ID: AUMIC1338-24; AM: K.379883. • ♀; NSW, Canterbury-Bankstown region, Little Salt Pan Creek; -33.9606, 151.027; 08 Feb. 2006; K. Harvey & J. Lee leg.; vacuum sampler; BOLD Process ID: AUMIC1337-24; AM: K.646432. • ♂; QLD, Townsville, Hermit Park; -19.2829, 146.801; 10 m; 14 Feb. 2017; Graeme Cocks leg.; UV Light trap; BOLD Process ID: GCQT658-17; WAM: 130562. • ♂; as previous except: 22 Feb. 2017; BOLD Process ID: GCQT712-17; WAM: 130563. • ♀; SA, Andamooka Station; -30.727, 137.204; 31 Aug. 2016; R. Leijs leg.; Vehicle net; Bush Blitz Lake Torrens, from -30.8198802, 137.1783585 to -30.6998403, 137.1574435; BOLD Process ID: AUMIC126-18; SAMA: 32-035446. • ♀; as previous except: BOLD Process ID: AUMIC359-18; SAMA: 32-035447. • ♂; as previous except: BOLD Process ID: AUMIC1252-24; SAMA: 32-035788. • ♀; as previous except: BOLD Process ID: AUMIC136-18; SAMA: 32-035456. • ♀; SA, Andamooka Station, 3.1 km ESE Andamooka HS; -30.7394, 137.229; 31 Aug.–3 Sep. 2016; B.A. Parslow & G. Taylor leg.; Malaise trap; chenopods on gibber BS 1097 AND002, Bushblitz Lake Torrens; BOLD Process ID: AUMIC132-18; SAMA: 32-035790. • ♀; SA, Australian Arid Land Botanic Gardens; -32.4618, 137.744; 72 m; 15 Sep. 2020; E. Fagan-Jeffries leg.; Sweeping; BOLD Process ID: OZBOL401-21; WAM: 130564. • ♀; SA, Streaky Bay; -32.798, 134.2; 22–29 Mar. 2022; Streaky Bay Area School students leg.; Malaise trap; Insect Investigators; BOLD Process ID: ASMII8316-22; SAMA: 32-47749.

###### Diagnostic description.

***Size***: Total body length: 2.2 mm; fore wing length: 2.4 mm. ***Head***: anterior scape colour similar or only very slightly paler than head colour; F2L/W ratio: 2.5; F14L/W ratio: 1.7. ***Mesosoma***: scutoscutellar sulcus with eight pits; mesoscutellar disc mostly smooth, or with very shallow scattered indentations; propodeal areola complete, or mostly so; propodeum mostly smooth; coxae colour (pro, meso, meta): all dark; metafemur colour mostly dark. ***Wings***: centre of pterostigma pigmented to same degree as the outer edges; fore wing r vein length/2RS vein length ratio: 1.4. ***Metasoma***: T1 shape narrowing distally, T1 medial length/anterior width between 1–2 × longer than wide; T1 mostly smooth; T2 mostly smooth; ovipositor sheath length/metatibia length ratio: 0.6.

*Apantelessinusulus* can be separated from most of the other species of *Apanteles* with dark metacoxa and metafemur, ovipositor sheaths > 0.6 × metatibia length, and antenna similar length or longer than body length, by having the metatibia mostly pale, the pterostigma uniformly coloured without a paler centre or pale spot on the proximal corner, and T1 mostly smooth with only a small rugose area in the centre. We do not morphologically diagnose this species against *A.focusalis*, but as the species are not closely related based on molecular data, they can be identified through DNA barcoding and the placement of the sequences on a phylogeny in the context of the holotype barcodes.

###### Etymology.

This species was named by students at Streaky Bay Area School (SA) who collected one of the paratypes. The epithet is a noun in apposition and means ‘little bay’, formed from the Latin ‘sinus’ (bay).

###### Distribution.

*Apantelessinusulus* has a broad distribution, with collection records in WA, SA, and NSW.

###### Molecular information.

Sequences of *Apantelessinusulus* are currently in BIN BOLD:ADH8678. The COI sequences are at least 8% divergent from any of the other species treated here, or any available sequence on BOLD. The *wg* sequences of the species are at least 24 bp different to any other species. All delimitation methods resolved *A.sinusulus* as a discrete species.

##### ﻿﻿ Apanteles subandinus

Taxon classificationAnimaliaHymenopteraBraconidae

Blanchard, 1947

000EE649-3B33-5E42-B03E-056E1D06579C

**Figure 58.**
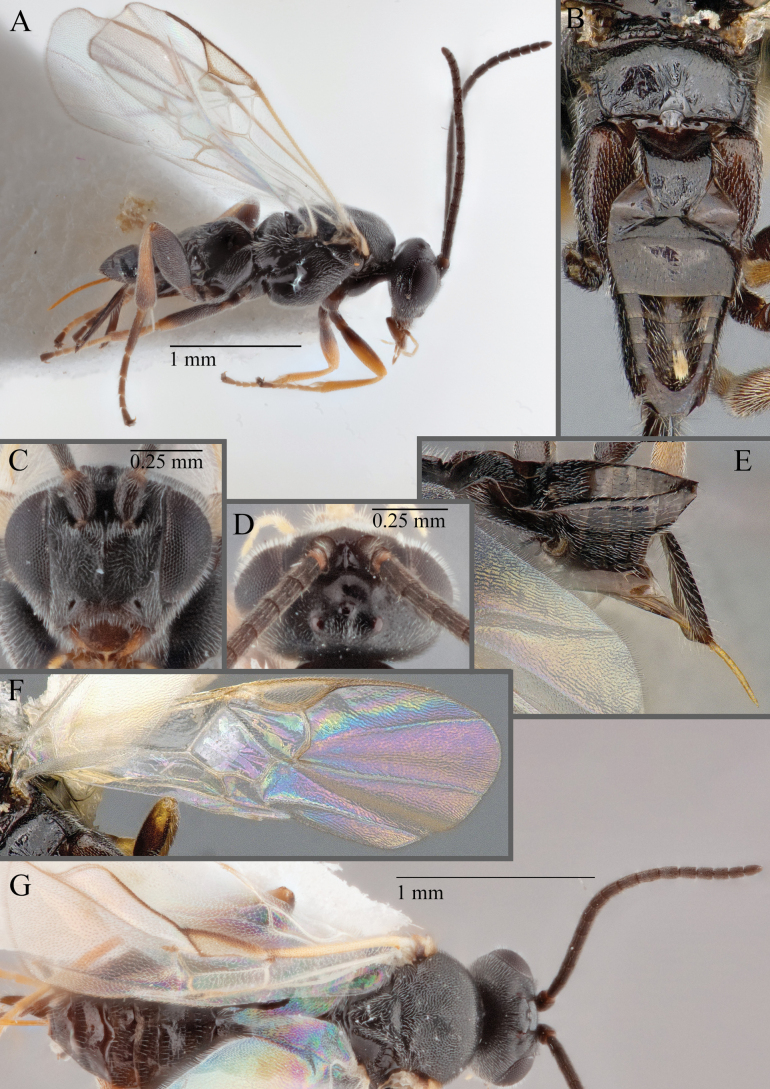
*Apantelessubandinus***A, C, D, G**WINC (Australia – SA) **B, E, F**CNC CNCHYM 00221 (USA) **A** lateral habitus **B** dorsal propodeum and metasoma **C** anterior head **D** dorsal head **E** lateral metasoma **F** fore wing **G** dorsal habitus.

Fig. 58 (examined material)

###### Holotype information.

♀; Argentina (MACN).

###### Examined material.

Diagnosis based on a female specimen from the USA: CNCHYM00221 (CNC); and a female specimen with the label data: “W.A.R.I [Waite Agricultural Research Institute] Glen Osmond SA, A. Eduayah, ex *Phthorimaeaoperculella*; ♀*Apantlessubandinus* Blanchard, I.D Naumann det. 1979 (WINC).

###### Diagnosis.

*Apantelessubandinus* is extremely distinct from all other described *Apanteles* species known from Australia, easily separated by T1 strongly narrowing posteriorly, pterostigma with pale centre, and the propodeum smooth with areola poorly defined by rather short carinae on posterior 1/2.

###### Notes.

*Apantelessubandinus* was introduced to Australia for biocontrol of the potato tuber moth (*Phthorimaeaoperculella* (Lepidoptera: Gelechiidae)) in the 1960s (Callan 1974) and was considered established, with shipments subsequently made to New Zealand in 1966 (Cameron et al. 1989). Whilst we have not collected any material that could possibly be *A.subandinus* during this project, it is possible it is restricted to agricultural areas where the host is present.

##### ﻿﻿ Apanteles translucentis

Taxon classificationAnimaliaHymenopteraBraconidae

Slater-Baker, Fagan-Jeffries, Fernández-Triana, Portmann & Oestmann sp. nov.

8133CE5E-FE52-5AA5-A8CE-59B1DA389BA4

https://zoobank.org/1A1A3D07-78C4-49D5-9EA3-1ECA2325A150

**Figure 59.**
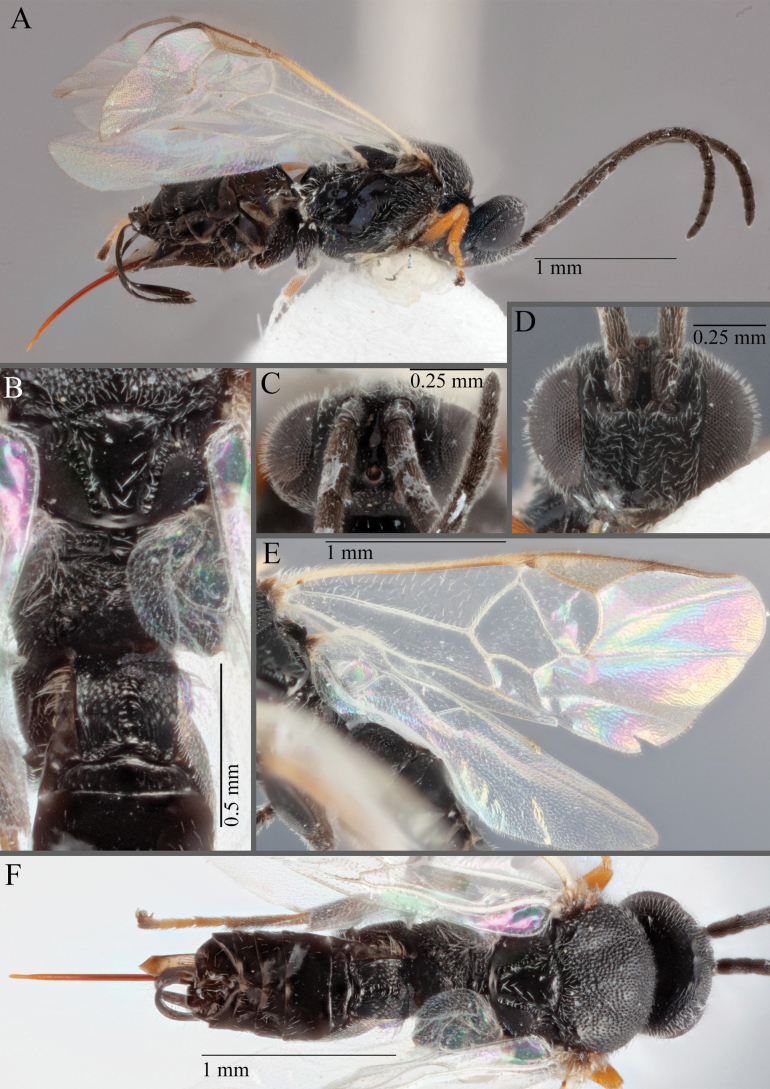
*Apantelestranslucentis* (holotype AUMIC1230-24). **A** lateral habitus **B** dorsal propodeum and T1–3 **C** dorsal head **D** anterior head **E** fore and hindwing **F** dorsal habitus.

Fig. 5C (distribution), Fig. 59 (holotype)

###### Type material.

***Holotype*.** Australia • ♀; QLD, Banana, Roadside few km south of Banana; -24.013, 150.88; 25 Nov. 2019; E. Fagan-Jeffries, J. B. Dorey & P. Ruhr leg.; sweeping vegetation; BOLD Process ID: AUMIC1230-24; QM: T261214.

###### Diagnostic description.

***Size***: Total body length: 2.8 mm; fore wing length: 2.9 mm. ***Head***: anterior scape colour similar or only very slightly paler than head colour; F2L/W ratio: 2.3; F14L/W ratio: 1.1. ***Mesosoma***: scutoscutellar sulcus with 11 pits; mesoscutellar disc mostly smooth, or with very shallow scattered indentations; propodeal areola complete, or mostly so; propodeum mostly rugose; coxae colour (pro, meso, meta): dark all; metafemur colour mostly dark. ***Wings***: centre of pterostigma paler (more hyaline) than outer edges; fore wing r vein length/2RS vein length ratio: 1.3. ***Metasoma***: T1 shape mostly parallel, T1 medial length/anterior width between 1–2 × longer than wide; mostly rugose; T2 mostly smooth; ovipositor sheath length/metatibia length ratio: 1.2.

*Apantelestranslucentis* can be separated from the other species of *Apanteles* in Australia which have the metacoxa and metafemur dark and the pterostigma with a paler centre by having fore wing with veins M+CU, 1 cu-a, 1M, 1CUa, 1CUb, (RS+M)a, 2RS, and 1m-cu all unpigmented or transparent, T1 with strong rugose sculpturing and the metatibia mostly pale. *Apantelestranslucentis* cannot be easily separated from *A.aeternus* using morphology, but the two species cluster discretely using COI and *wg* barcodes.

###### Etymology.

The species epithet is a Latin participle (in the genitive case) of *translucens*, meaning translucent, and refers to the centre of the pterostigma.

###### Distribution.

*Apantelestranslucentis* is currently only known from one collection record in northern QLD, from the town of Banana (which is famously named after a yellowish coloured bullock called “Banana”).

###### Molecular information.

The DNA barcode of the holotype of *Apantelestranslucentis* is the only sequence currently in – BIN BOLD:AFR5983. The COI sequence is at least 4% divergent from any of the other species treated here, or any available sequence on BOLD. The *wg* sequence of the holotype is at least 4 bp different to any other species. BINs, 2% divergent threshold, COIPTP and *wg* haplotypes resolved *A.translucentis* as a discrete species, whilst *wg*ASAP grouped it with *A.oenone*. COIASAP and *wg*PTP grouped *A.translucentis*, *A.oenone*, and *A.aeternus* together. This complex of species is not well resolved and would benefit from more intensive study to better establish species boundaries.

##### ﻿﻿ Apanteles vala

Taxon classificationAnimaliaHymenopteraBraconidae

Nixon, 1965

7C6F5A7D-F4FD-509F-9E12-09C2000260E2

Fig. 6B (distribution), Fig. 43A (holotype)

###### Holotype information.

♀; Australia, QLD, Tambourine Mts., 11–17 May 1935 R. E. Turner leg. (NHM).

###### Examined material.

Image of the type and original description used to form the diagnosis.

###### Diagnosis.

*Apantelesvala* can be separated from the other species of *Apanteles* in Australia with a dark metacoxa and metafemur, a uniformly pigmented pterostigma, ovipositor sheaths > 0.6 × metatibia length and antennae similar size to the body, by the metatibia mostly pale with dark colouration only on distal 1/3, T1 with strong sculpture over at least most of posterior 1/2 of tergite, mesoscutellar disc mostly smooth, fore wing veins 1M and 1CUa of similar pigmentation, and T2 posterior length/width ratio: ~ 3.5.

###### Notes.

We did not collect any specimens that appeared to be conspecific with *A.vala*.

## ﻿﻿Discussion

Australia’s insect biodiversity is fundamentally undiscovered and under-described (Raven and Yeates 2007) and the need for accelerated identification and bioassessment tools of hyper-diverse insect taxa is high (Meierotto et al. 2019; Meier et al. 2024). Parasitoid wasps perform high-value ecosystems services (Zhu et al. 2020) and their documentation as a hyper-diverse taxon group is known to be extremely difficult. Here, in a major contribution to accelerated taxonomy of Australia’s insect fauna we contribute 34 new species of parasitic wasp from the braconid genus *Apanteles* in Australia, increasing the number of named *Apanteles* species reported from Australia by 370%. In doing so, we contribute to the Taxonomy Australia mission of documenting Australia’s biodiversity in a generation (Taxonomy Decadal Plan Working Group 2018). We adopted a moderately accelerated approach using high-throughput DNA barcoding and a species concept of integrative species delimitation using a combination of molecular and morphological evidence. In more than 80% of cases, at least five of the seven molecular delimitation methods were congruent, and these molecular hypotheses were often supported by morphological characters. While there were cases of incongruence among methods, and several ‘less certain’ species that we recommend need more intensive research, the species hypotheses presented here attempts to strike a balance between increasing the speed of documenting Australia’s biodiversity and creating an accessible taxonomic framework for future research.

Sharkey et al. (2021) and Meierotto et al. (2019) advocate for minimalist approaches to taxonomic description using DNA barcodes, Barcode of Life Database identification codes, and specimen photographs alone as a mechanism for ameliorating a global taxonomic impediment (Wheeler et al. 2004), focussing on parasitic wasps for exemplar species descriptions. A substantial backlash from the taxonomic community followed (e.g., Meier et al. 2021; Zamani et al. 2021, 2022; Sharkey et al. 2022), highlighting the value that the taxonomic community places on rigorous and morphology-based taxonomic descriptions. However, with most researchers, we recognise that DNA sequence data and species delimitation play a critical role in a unified species concept (De Queiroz 2007) for incorporating and characterising genetic diversity in hyper-diverse groups. Without the use of DNA barcodes, our study would not have been possible; the rapid initial sorting of specimens into potential species, the identification of named species amongst our material, and the awareness that some of the lineages in Australia are found in other countries, were all made possible only by DNA barcoding and public reference databases.

Re-sampling from existing bulk material has revealed the exceptional biodiversity discovery resources available in bulk samples collected during field campaigns, frequently collected as part of collaborative programs, and stored in public institutions. Through using specimens collected up to 30 years ago and stored in bulk ethanol vials, we have demonstrated that the quality of the specimen morphology and DNA integrity is still high despite long-term storage, and therefore the value of museum collections to modern biodiversity documentation cannot be understated. A weakness of the specimen dataset used in this study is that almost all are devoid of host relationship information, having been mostly collected in Malaise traps and by sweep netting. However, it is hoped that by providing images and sequence data, that when *Apanteles* are reared by lepidopterists (both professional and amateur) in the future, that species can be gradually associated with hosts.

We also highlight the unique opportunity of engaging school children in citizen science projects. ‘Insect Investigators’, a citizen science program (2022) that partnered scientists with 50 schools in three Australian states, provided new specimens at locations frequently too expensive to undertake exhaustive surveys or expeditions. The program contributed 12,000+ new arthropod DNA sequences to public databases and 17 specimens of seven different species to the current study. The program successfully demonstrated the role of citizen scientists, especially school children and teachers, in documenting Australia’s insect biodiversity.

## Supplementary Material

XML Treatment for Apanteles

XML Treatment for Apanteles adustus

XML Treatment for Apanteles aeternus

XML Treatment for Apanteles alatomicans

XML Treatment for Apanteles allapsus

XML Treatment for Apanteles amicalis

XML Treatment for Apanteles apollo

XML Treatment for Apanteles apricus

XML Treatment for Apanteles artemis

XML Treatment for Apanteles aurantius

XML Treatment for Apanteles auroralis

XML Treatment for Apanteles banrock

XML Treatment for Apanteles breviflagellarius

XML Treatment for Apanteles brockhedgesi

XML Treatment for Apanteles carpatus

XML Treatment for Apanteles cuprum

XML Treatment for Apanteles darthvaderi

XML Treatment for Apanteles doreenwatlerae

XML Treatment for Apanteles ethanbeaveri

XML Treatment for Apanteles fenestrinus

XML Treatment for Apanteles ferripulvis

XML Treatment for Apanteles focusalis

XML Treatment for Apanteles fundulus

XML Treatment for Apanteles galleriae

XML Treatment for Apanteles hades

XML Treatment for Apanteles hemara

XML Treatment for Apanteles insulanus

XML Treatment for Apanteles ippeus

XML Treatment for Apanteles kelpiellus

XML Treatment for Apanteles lamingtonensis

XML Treatment for Apanteles ligdus

XML Treatment for Apanteles magicus

XML Treatment for Apanteles margaritarius

XML Treatment for Apanteles oenone

XML Treatment for Apanteles pellucidus

XML Treatment for Apanteles persephone

XML Treatment for Apanteles phantasmatus

XML Treatment for Apanteles pharusalis

XML Treatment for Apanteles ramsaris

XML Treatment for Apanteles rufiterra

XML Treatment for Apanteles sinusulus

XML Treatment for Apanteles subandinus

XML Treatment for Apanteles translucentis

XML Treatment for Apanteles vala

## Footnotes

These are closely related species and this is a subtle character, but the two species can be identified by DNA barcoding.

This character is subtle and these three closely related species (A. kelpiellus, A. cuprum, and A. margaritarius) may be best separated by DNA barcoding.

Apanteles aeternus and A. translucentis are closely related species most reliably separated by DNA barcoding.

These two species are very similar morphologically, and ideally identification should be confirmed by DNA barcoding.

Metatibia colour in A. adustus (Fig. 12M) could be interpreted either way; therefore, it is included in both halves of the couplet.

Whereas the holotype of A. magicus will key to the second half of this couplet, as it has a small amount of infuscation, other specimens (here assigned to this species) have no infuscation. The species in this part of the key (A. phantasmus, A. hades, A. darthvaderi, A. alatomicans, and A. magicus) should ideally be identified with the assistance of DNA barcoding as the morphological characters used here to separate them are subtle and may be prone to variation or different interpretation.

Apanteles persephone also runs to here if metatibia is interpreted in couplet 27 as going to the first half of that couplet, although the most likely interpretation based on Nixon’s description suggests that the second half of couplet 25 would be more likely to be correct.

If specimens of A. magicus are lacking fore wing infuscation, they will key to here also. These two species can be distinguished with the assistance of DNA barcoding.

See note concerning specimens of A. lamingtonensis from the ACT under the remarks of that species.

These species are most reliably separated by DNA barcoding.

A. persephone runs to here if metatibia is interpreted in couplet 25 as going to the second part of that couplet (likely the most valid interpretation based on Nixon’s description); if interpreted as going through the first part of couplet 27 it would end on couplet 27, similar to A. darthvaderi.

## ﻿﻿Additional information

### Author ORCIDs

Mollie-Rosae Slater-Baker https://orcid.org/0000-0003-0627-3117

Erinn P. Fagan-Jeffries https://orcid.org/0000-0002-3322-6255

Katherine J. Oestmann https://orcid.org/0009-0004-3775-7127

Olivia G. Portmann https://orcid.org/0009-0004-4099-3155

Tiahni M. Bament https://orcid.org/0009-0009-7749-5535

Andy G. Howe https://orcid.org/0000-0002-7460-5227

Michelle T. Guzik https://orcid.org/0000-0002-4947-9353

Tessa M. Bradford https://orcid.org/0000-0003-0607-1398

Alana R. McClelland https://orcid.org/0000-0002-1373-1051

José Fernández-Triana https://orcid.org/0000-0003-0425-0309

### Data availability

All of the data that support the findings of this study are available in the main text or Supplementary Information.

## Supplementary materials

﻿Supplementary material 1 Full species delimitation results Data type pdf Explanation note species delimitation results mapped alongside the phylogeny. https://binary.pensoft.net/file/1247781This dataset is made available under the Open Database License (http://opendatacommons.org/licenses/odbl/1.0/). The Open Database License (ODbL) is a license agreement intended to allow users to freely share, modify, and use this Dataset while maintaining this same freedom for others, provided that the original source and author(s) are credited.Mollie-Rosae Slater-Baker, Erinn P. Fagan-Jeffries

Supplementary material 2 Images Data type pdf Explanation note figure plates of the delimited species not described and found in countries other than Australia. https://binary.pensoft.net/file/1247782This dataset is made available under the Open Database License (http://opendatacommons.org/licenses/odbl/1.0/). The Open Database License (ODbL) is a license agreement intended to allow users to freely share, modify, and use this Dataset while maintaining this same freedom for others, provided that the original source and author(s) are credited.Erinn P. Fagan-Jeffries, Katherine J. Oestmann, Olivia G. Portmann

Supplementary material 3 Morphological characters Data type xlsx Explanation note table of morphological character coding. https://binary.pensoft.net/file/1247783This dataset is made available under the Open Database License (http://opendatacommons.org/licenses/odbl/1.0/). The Open Database License (ODbL) is a license agreement intended to allow users to freely share, modify, and use this Dataset while maintaining this same freedom for others, provided that the original source and author(s) are credited.Mollie-Rosae Slater-Baker, Erinn P. Fagan-Jeffries, Katherine J. Oestmann, Olivia G. Portmann

Supplementary material 4 Apanteles phylogeny raw file Data type newick Explanation note World Apanteles tree (Fig. 3) in newick format. https://binary.pensoft.net/file/1247784This dataset is made available under the Open Database License (http://opendatacommons.org/licenses/odbl/1.0/). The Open Database License (ODbL) is a license agreement intended to allow users to freely share, modify, and use this Dataset while maintaining this same freedom for others, provided that the original source and author(s) are credited.Erinn P. Fagan-Jeffries, Mollie-Rosae Slater-Baker

Supplementary material 5 Material examined Data type xlsx Explanation note spreadsheet of all sequence data and material examined. https://binary.pensoft.net/file/1247785This dataset is made available under the Open Database License (http://opendatacommons.org/licenses/odbl/1.0/). The Open Database License (ODbL) is a license agreement intended to allow users to freely share, modify, and use this Dataset while maintaining this same freedom for others, provided that the original source and author(s) are credited.Mollie-Rosae Slater-Baker, Erinn P. Fagan-Jeffries

## References

[B1] Ahrens D Ahyong ST Ballerio A Barclay MVL Eberle J Espeland M Huber BA Mengual X Pacheco TL Peters RS Rulik B Vaz-de-Mello F Wesener T Krell F-T (2021) Is it time to describe new species without diagnoses? – A comment on Sharkey et al. (2021). Zootaxa 5027(2): 151–159. 10.11646/zootaxa.5027.2.1 34811237

[B2] Austin AD Dangerfield PC (1992) Synopsis of Australasian Microgastrinae (Hymenoptera: Braconidae), with a key to genera and description of new taxa. Invertebrate Systematics 6: 1–76. 10.1071/IT9920001

[B3] Brower AVZ Desalle R (1998) Patterns of mitochondrial versus nuclear DNA sequence divergence among nymphalid butterflies: the utility of wingless as a source of characters for phylogenetic inference. Insect Molecular Biology 7: 73–82. 10.1046/j.1365-2583.1998.71052.x 9459431

[B4] Callan EMcC (1974) Changing status of the parasites of potato tuber moth *Phthorimaeaoperculella* (Lepidoptera: Gelechiidae) in Australia. Entomophaga 19: 97–101. 10.1007/BF02371513

[B5] Cameron P Hill R Bain J Thomas W (1989) A Review of Biological Control of Invertebrate Pests and Weeds in New Zealand 1874–1987. Technical Communication No 10. CAB International Institute of Biological Control. DSIR Entomology Division, 424 pp.

[B6] Cruaud P Rasplus JY Rodriguez LJ Cruaud A (2017) High-throughput sequencing of multiple amplicons for barcoding and integrative taxonomy. Scientific Reports 7: 41948. 10.1038/srep41948 PMC5292727 28165046

[B7] de Queiroz K (1998) The General Lineage Concept of Species, Species Criteria, and the Process of Speciation. In: Endless Forms: Species and Speciation. Oxford University Press, Oxford, 57–75. 10.1080/10635150701701083

[B8] De Queiroz K (2007) Species Concepts and Species Delimitation. Systematic Biology 56: 879–886. 10.1080/10635150701701083 18027281

[B9] Eady RD (1968) Some illustrations of microsculpture in the Hymenoptera. Proceedings of the Royal Entomological Society of London, Series A, General Entomology 43: 66–72. 10.1111/j.1365-3032.1968.tb01029.x

[B10] Fagan-Jeffries EP Cooper SJB Bertozzi T Bradford TM Austin AD (2018) DNA barcoding of microgastrine parasitoid wasps (Hymenoptera: Braconidae) using high-throughput methods more than doubles the number of species known for Australia. Molecular Ecology Resources 18: 1132–1143. 10.1111/1755-0998.12904 29791787

[B11] Fernández-Triana JL (2010) Eight new species and an annotated checklist of Microgastrinae (Hymenoptera, Braconidae) from Canada and Alaska. ZooKeys 63: 1–53. 10.3897/zookeys.63.565 PMC3088399 21594019

[B12] Fernández-Triana JL Whitfield JB Rodriguez JJ Alex Smith M Janzen DH Hallwachs WD Hajibabaei M Burns JM Alma Solis M Brown J Cardinal S Goulet H Hebert PDN (2014) Review of *Apanteles* sensu stricto (Hymenoptera, Braconidae, Microgastrinae) from Area de Conservación Guanacaste, northwestern Costa Rica, with keys to all described species from Mesoamerica. ZooKeys 383: 1–565. 10.3897/zookeys.383.6418 PMC3950464 24624021

[B13] Fernández-Triana J Beaudin M van Achterberg K Agbodzavu MK Othim STO Nyamu FW Fiaboe KKM (2017) DNA barcodes, expanded distribution, and redescription of *Apanteleshemara* Nixon, 1965 (Hymenoptera, Braconidae, Microgastrinae), a potential biocontrol species against amaranth leaf-webbers in Africa. Journal of Hymenoptera Research 58: 1–15. 10.3897/jhr.58.13361

[B14] Fernández-Triana J Shaw MR Boudreault C Beaudin M Broad GR (2020) Annotated and illustrated world checklist of Microgastrinae parasitoid wasps (Hymenoptera, Braconidae). ZooKeys 920: 1–1089. 10.3897/zookeys.920.39128 32390740 PMC7197271

[B15] Fernández-Triana JL Shimbori EM Whitfield JB Penteado-Dias AM Shaw SR Boudreault C Sones J Perez K Brown A Manjunath R Burns JM Hebert PDN Smith MA Hallwachs W Janzen DH (2023) A revision of the parasitoid wasp genus *Alphomelon* Mason with the description of 30 new species (Hymenoptera, Braconidae). ZooKeys 1175: 5–162. 10.3897/zookeys.1175.105068 37636532 PMC10448698

[B16] Folmer O Black M Hoeh Lutz R Vrijenhoek R (1994) DNA primers for amplification of mitochondrial cytochrome c oxidase subunit I from diverse metazoan invertebrates. Molecular Marine Biology and Biotechnology 3: 294–299. 7881515

[B17] Hebert PDN Cywinska A Ball SL DeWaard JR (2003) Biological identifications through DNA barcodes. Proceedings of the Royal Society B: Biological Sciences 270: 313–321. 10.1098/rspb.2002.2218 PMC1691236 12614582

[B18] Hoang DT Chernomor O Von Haeseler A Minh BQ Vinh LS (2018) UFBoot2: Improving the ultrafast bootstrap approximation. Molecular Biology and Evolution 35: 518–522. 10.1093/molbev/msx281 29077904 PMC5850222

[B19] Höcherl A Shaw MR Boudreault C Rabl D Haszprunar G Raupach MJ Schmidt S Baranov V Fernández-Triana J (2024) Scratching the tip of the iceberg: integrative taxonomy reveals 30 new species records of Microgastrinae (Braconidae) parasitoid wasps for Germany, including new Holarctic distributions. ZooKeys 1188: 305–386. 10.3897/zookeys.1188.112516 38250474 PMC10797786

[B20] Ivanova NV Dewaard JR Hebert PDN (2006) An inexpensive, automation-friendly protocol for recovering high-quality DNA. Molecular Ecology Notes 6: 998–1002. 10.1111/j.1471-8286.2006.01428.x

[B21] Jasso-Martínez JM Santos BF Zaldívar-Riverón A Fernández-Triana JL Sharanowski BJ Richter R Dettman JR Blaimer BB Brady SG Kula RR (2022) Phylogenomics of braconid wasps (Hymenoptera, Braconidae) sheds light on classification and the evolution of parasitoid life history traits. Molecular Phylogenetics and Evolution 173: 107452. 10.1016/j.ympev.2022.107452 35307517

[B22] Juen A Hogendoorn K Ma G Schmidt O Keller MA (2012) Analysing the diets of invertebrate predators using terminal restriction fragments. Journal of Pest Science 85: 89–100. 10.1007/s10340-011-0406-x

[B23] Kalyaanamoorthy S Minh BQ Wong TKF Von Haeseler A Jermiin LS (2017) ModelFinder: Fast model selection for accurate phylogenetic estimates. Nature Methods 14: 587–589. 10.1038/nmeth.4285 28481363 PMC5453245

[B24] Katoh K Standley DM (2013) MAFFT multiple sequence alignment software version 7: Improvements in performance and usability. Molecular Biology and Evolution 30: 772–780. 10.1093/molbev/mst010 23329690 PMC3603318

[B25] Katoh K Misawa K Kuma KI Miyata T (2002) MAFFT: A novel method for rapid multiple sequence alignment based on fast Fourier transform. Nucleic Acids Research 30: 3059–3066. 10.1093/nar/gkf436 12136088 PMC135756

[B26] Letunic I Bork P (2024) Interactive Tree of Life (iTOL) v6: recent updates to the phylogenetic tree display and annotation tool. Nucleic Acids Research 2024: 1–5. 10.1093/nar/gkae268 PMC11223838 38613393

[B27] Liu Z Chen XX (2023) The *taeniaticornis*-group of genus *Apanteles* Foerster (Hymenoptera, Braconidae, Microgastrinae) from China with one new species. Journal of Hymenoptera Research 96: 21–31. 10.3897/jhr.96.99649

[B28] Liu Z He J Chen X (2014) The *grandiculus*- and *metacarpalis*-groups of the genus *Apanteles* Foerster, 1862 (Hymenoptera, Braconidae, Microgastrinae) from China, with descriptions of eight new species. Zootaxa 3765: 435–457. 10.11646/zootaxa.3765.5.3 24870913

[B29] Liu Z He JH Chen XX (2015) The *lacteus*-, *laspeyresiella*- and *mycetophilus*-groups of *Apanteles* Foerster, 1862 (Hymenoptera, Braconidae, Microgastrinae) in China, with descriptions of eight new species. Zootaxa 3949: 370–392. 10.11646/zootaxa.3949.3.4 25947813

[B30] Liu Z He JH Chen XX Gupta A (2020) The *ater*-group of the genus *Apanteles* Foerster (Hymenoptera, Braconidae, Microgastrinae) from China with the descriptions of forty-eight new species. Zootaxa 4807: 1–205. 10.11646/zootaxa.4807.1.1 33056002

[B31] Martínez JJ Berta C Varone L Logarzo G Zamudio P Zaldívar-Riverón A Aguilar-Velasco RG (2012) DNA barcoding and morphological identification of Argentine species of *Apanteles* (Hymenoptera: Braconidae), parasitoids of cactus-feeding moths (Lepidoptera: Pyralidae: Phycitinae), with description of a new species. Invertebrate Systematics 26: 435–444. 10.1071/IS12060

[B32] Meier R Shiyang K Vaidya G Ng PKL (2006) DNA barcoding and taxonomy in Diptera: A tale of high intraspecific variability and low identification success. Systematic Biology 55: 715–728. 10.1080/10635150600969864 17060194

[B33] Meier R Blaimer B Buenaventura E Hartop E Rintelen T von Srivathsan A Yeo D (2021) A re-analysis of the data in Sharkey et al.’s (2021) minimalist revision reveals that BINs do not deserve names, but BOLD Systems needs a stronger commitment to open science. bioRxiv. 10.1101/2021.04.28.441626 34487362

[B34] Meier R Hartop E Pylatiuk C Srivathsan A (2024) Towards holistic insect monitoring: species discovery, description, identification and traits for all insects. Philosophical Transactions of the Royal Society B: Biological Sciences 379: 20230120. 10.1098/rstb.2023.0120 PMC11070263 38705187

[B35] Meierotto S Sharkey MJ Janzen DH Hallwachs W Hebert PDN Chapman EG Smith MA (2019) A revolutionary protocol to describe understudied hyperdiverse taxa and overcome the taxonomic impediment. Deutsche Entomologische Zeitschrift 66(2): 66: 119–145. 10.3897/dez.66.34683

[B36] Meyer M Kircher M (2010) Illumina Sequencing Library Preparation for Highly Multiplexed Target Capture and Sequencing. Cold Spring Harbor Protocols 2010. 10.1101/pdb.prot5448 20516186

[B37] Minh BQ Schmidt HA Chernomor O Schrempf D Woodhams MD von Haeseler A Lanfear R (2020) IQ-TREE 2: New models and efficient methods for phylogenetic inference in the genomic era. Molecular Biology and Evolution 37: 1530–1534. 10.1093/molbev/msaa015 32011700 PMC7182206

[B38] Nguyen LT Schmidt HA Von Haeseler A Minh BQ (2015) IQ-TREE: A fast and effective stochastic algorithm for estimating maximum-likelihood phylogenies. Molecular Biology and Evolution 32: 268–274. 10.1093/molbev/msu300 25371430 PMC4271533

[B39] Nixon GEJ (1965) A reclassification of the tribe Microgasterini (Hymenoptera: Braconidae). Bulletin of the British Museum (Natural History) Entomology Supp. 2 2: 1–284. 10.5962/p.144036

[B40] Puillandre N Brouillet S Achaz G (2021) ASAP: assemble species by automatic partitioning. Molecular Ecology Resources 21: 609–620. 10.1111/1755-0998.13281 33058550

[B41] Quicke DLJ Smith MA Janzen DH Hallwachs W Fernandez-Triana J Laurenne NM Zaldi A Shaw MR Broad GR Klopfstein S Shaw SR Hrcek JAN Hebert PDN Miller SE Rodriguez JJ Whitfield JB Sharkey MJ Sharanowski BJ Jussila R Gauld ID Chesters D Vogler AP (2012) Utility of the DNA barcoding gene fragment for parasitic wasp phylogeny (Hymenoptera: Ichneumonoidea): data release and new measure of taxonomic congruence. 44: 676–685. 10.1111/j.1755-0998.2012.03143.x 22487608

[B42] Ratnasingham S Hebert PDN (2007) BOLD: The Barcode of Life Data System. Molecular Ecology Notes 7: 355–364. 10.1111/j.1471-8286.2007.01678.x 18784790 PMC1890991

[B43] Ratnasingham S Hebert PDN (2013) A DNA-Based registry for all animal species: The Barcode Index Number (BIN) System. PLOS ONE 8: 66213. 10.1371/journal.pone.0066213 PMC3704603 23861743

[B44] Raven PH Yeates DK (2007) Australian biodiversity: threats for the present, opportunities for the future. Australian Journal of Entomology 46: 177–187. 10.1111/j.1440-6055.2007.00601.x

[B45] Rousse P Gupta A (2013) Microgastrinae (Hymenoptera: Braconidae) of Reunion Island: a catalogue of the local species, including 18 new taxa and a key to species. Zootaxa 3616: 501–547. 10.11646/zootaxa.3616.6.1 [Accessed 2 July 2 2015] 24758826

[B46] Sánchez-García JA Figueroa JI Whitfield JB Pineda S Martínez AM (2015) A new species of *Apanteles* Foerster (Hymenoptera: Braconidae) parasitic of two blackberry leafrollers (Lepidoptera: Tortricidae) in Mexico. Journal of the Kansas Entomological Society 88: 10–15. 10.2317/JKES1407.02.1

[B47] Sharkey MJ Janzen DH Hallwachs W Chapman EG Alex Smith M Dapkey T Brown A Ratnasingham S Naik S Manjunath R Perez K Milton M Hebert P Shaw SR Kittel RN Alma Solis M Metz MA Goldstein PZ Brown JW Quicke DLJ van Achterberg C Brown BV Burns JM (2021) Minimalist revision and description of 403 new species in 11 subfamilies of Costa Rican braconid parasitoid wasps, including host records for 219 species. ZooKeys 2021: 1–665. 10.3897/zookeys.1013.55600 PMC8390796 34512087

[B48] Sharkey MJ Tucker EM Baker A Smith MA Ratnasingham S Manjunath R Hebert P Hallwachs W Janzen D (2022) More discussion of minimalist species descriptions and clarifying some misconceptions contained in Meier et al. 2021. ZooKeys 1110: 135–149. 10.3897/zookeys.1110.85491 36761452 PMC9848685

[B49] Shokralla S Porter TM Gibson JF Dobosz R Janzen DH Hallwachs W Golding GB Hajibabaei M (2015) Massively parallel multiplex DNA sequencing for specimen identification using an Illumina MiSeq platform. Scientific Reports 5: 9687. 10.1038/srep09687 PMC4401116 25884109

[B50] Smith MA Fernández-Triana JL Eveleigh E Gómez J Guclu C Hallwachs W Hebert PDN Hrcek J Huber JT Janzen D Mason PG Miller S Quicke DLJ Rodriguez JJ Rougerie R Shaw MR Várkonyi G Ward DF Whitfield JB Zaldívar-Riverón A (2013) DNA barcoding and the taxonomy of Microgastrinae wasps (Hymenoptera, Braconidae): impacts after 8 years and nearly 20 000 sequences. Molecular Ecology Resources 13: 168–176. 10.1111/1755-0998.12038 23228011

[B51] Smith MA Rodriguez JJ Whitfield JB Deans AR Janzen DH Hallwachs W Hebert PDN (2008) Extreme diversity of tropical parasitoid wasps exposed by iterative integration of natural history, DNA barcoding, morphology, and collections, Proceedings of the National Academy of Sciences 105: 12359–12364. 10.1073/pnas.0805319105 PMC2518452 18716001

[B52] Srivathsan A Lee L Katoh K Hartop E Kutty SN Wong J Yeo D Meier R (2021) ONTbarcoder and MinION barcodes aid biodiversity discovery and identification by everyone, for everyone. BMC Biology 19: 1–21. 10.1186/s12915-021-01141-x 34587965 PMC8479912

[B53] Stahlhut JK Fernández-Triana J Adamowicz SJ Buck M Goulet H Hebert PDN Huber JT Merilo MT Sheffield CS Woodcock T Smith MA (2013) DNA barcoding reveals diversity of Hymenoptera and the dominance of parasitoids in a sub-arctic environment. BMC Ecology 13: 2. 10.1186/1472-6785-13-2 PMC3565895 23351160

[B54] Taxonomy Decadal Plan Working Group (2018) Discovering Biodiversity: A decadal plan for taxonomy and biosystematics in Australia and New Zealand 2018–2028. Canberra and Wellington. 10.1111/j.1469-8137.2010.03389.x

[B55] Trifinopoulos J Nguyen LT von Haeseler A Minh BQ (2016) W-IQ-TREE: a fast online phylogenetic tool for maximum likelihood analysis. Nucleic Acids Research 44: W232–W235. 10.1093/nar/gkw256 PMC4987875 27084950

[B56] van Achterberg C Hosaka T Ng YF Ghani IBA (2009) The braconid parasitoids (Hymenoptera: Braconidae) associated with seeds of Dipterocarpaceae in Malaysia. Journal of Natural History 43: 635–686. 10.1080/00222930802610501

[B57] Vasilita C Feng V Hansen AK Hartop E Srivathsan A Struijk R Meier R (2024) Express barcoding with NextGenPCR and MinION for species-level sorting of ecological samples. Molecular Ecology Resources 24: e13922. 10.1111/1755-0998.13922 38240168

[B58] Wheeler QD Raven PH Wilson EO (2004) Taxonomy: Impediment or expedient? Science 303: 285. 10.1126/science.303.5656.285 14726557

[B59] Whitfield JB Cameron Sydney A Rami SR Roesch K Messinger S Taylor OM Cole D (2001) Review of the *Apanteles* species (Hymenopter: Braconidae) attacking lepidoptera in Bombus (Fervidobombus) (Hymenoptera: Apidae) colonies in the New World, with description of a new species from South America. Annals of the Entomological Society of America 94: 851–857. 10.1603/0013-8746(2001)094[0851:ROTASH]2.0.CO;2

[B60] Yoder MJ Mikó I Seltmann KC Bertone MA Deans AR (2010) A Gross Anatomy Ontology for Hymenoptera. Moreau CS (Ed.). PLOS ONE 5: e15991. 10.1371/journal.pone.0015991 PMC3012123 21209921

[B61] Zamani A Vahtera V Sääksjärvi IE Scherz MD (2021) The omission of critical data in the pursuit of ‘revolutionary’ methods to accelerate the description of species. Systematic Entomology 46: 1–4. 10.1111/syen.12444

[B62] Zamani A Fric ZF Gante HF Hopkins T Orfinger AB Scherz MD Bartoňová AS Pos DD (2022) DNA barcodes on their own are not enough to describe a species. Systematic Entomology 47: 385–389. 10.1111/syen.12538

[B63] Zhang Z Schwartz S Wagner L Miller W (2000) A greedy algorithm for aligning DNA sequences, Journal of Computational Biology 7: 203–214. 10.1089/10665270050081478 10890397

[B64] Zhang J Kapli P Pavlidis P Stamatakis A (2013) A general species delimitation method with applications to phylogenetic placements. Bioinformatics 29: 2869–2876. 10.1093/bioinformatics/btt499 23990417 PMC3810850

[B65] Zhu P Zheng X Xie G Chen G Lu Z Gurr G (2020) Relevance of the ecological traits of parasitoid wasps and nectariferous plants for conservation biological control: a hybrid meta-analysis. Pest Management Science 76: 1881–1892. 10.1002/ps.5719 31840379

