# Supplementary material for: ﻿DNA barcoding, integrative taxonomy, citizen science, and Bush Blitz surveys combine to reveal 34 new species of Apanteles (Hymenoptera, Braconidae, Microgastrinae) in Australia
Source: Zookeys. 2025 Feb 11;1227:1–128. doi: 10.3897/zookeys.1227.130467 (PMC11836623; doi:10.3897/zookeys.1227.130467)
Supplement: ﻿Supplementary material 1 — Full species delimitation results [file zookeys-1227-001_article-130467__-s001.pdf]

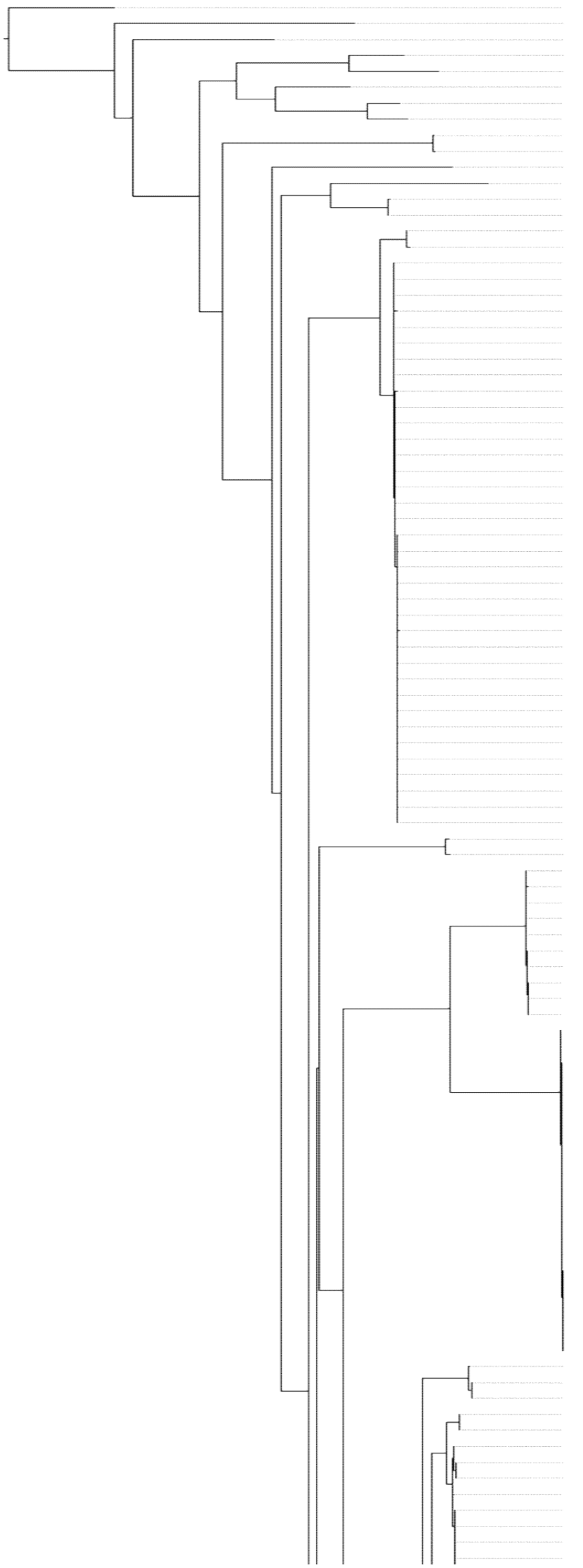

	COI				WG					
	BINS	2%	K80 ASAP	PTP	Haplotypes	K80 ASAP	PTP	Consensus	Collected from:	Agree
AUMIC045-18 Microplitis demolitor Australia BOLD AAD3894										
AUMIC143-18 Choeras bushblitz Australia BOLD ADL3153										
AUMIC078-18 Choeras morialta Australia BOLD ADK2306										
AUMIC462-18 Dolichogenidea garytaylori Australia BOLD ADL4226										
AUMIC459-18 Dolichogenidea bonbonensis Australia BOLD ADL4681										
AUMIC021-18 Glyptapanteles harveyi Australia BOLD ADL3315										
AUMIC134-18 Cotesia tjapekki Australia BOLD ADL5542										
AUMIC033-18 Cotesia deliadis Australia BOLD AAA7143										
AUMIC460-18 Iconella Australia BOLD ADL3294										
AUMIC377-18 Iconella Australia BOLD ADL3294										
AUMIC051-18 Apanteles Australia BOLD ADL5064								*A. apollo* / MRSB01	Northern Territory	7/7
AUMIC479-18 Apanteles Australia BOLD ADL5654								*A. artemis* / MRSB02	Northern Territory	7/7
AUMIC1282-24 Braconidae Australia BOLD AFQ8210								MRSB03	South Australia	7/7
AUMIC1279-24 Braconidae Australia BOLD AFQ8210					X	X	X		South Australia	
AUMIC240-18 Apanteles Australia BOLD AAM5747					X	X	X	*A.* sp. nr. *carpatus* / MRSB04.B	New South Wales	4/4
HYQT720-10 Apanteles Australia BOLD AAM5747					X	X	X		Queensland	
JSHYO003-11 Apanteles carpatus Canada BOLD AAC2372					X	X	X	*A. carpatus* / MRSB04	Canada	7/7
JSAUG1452-11 Apanteles carpatus Canada BOLD AAC2372					X	X	X		Canada	
CNCHZ239-09 Apanteles carpatus Canada BOLD AAC2372					X	X	X		Canada	
CNCHZ216-09 Apanteles carpatus Canada BOLD AAC2372					X	X	X		Canada	
SMTPL7612-15 Apanteles carpatus Canada BOLD AAC2372					X	X	X		Canada	
CNCHZ236-09 Apanteles carpatus Canada BOLD AAC2372					X	X	X		Canada	
CNCHZ294-09 Apanteles carpatus Canada BOLD AAC2372					X	X	X		Canada	
CNCHZ253-09 Apanteles carpatus Canada BOLD AAC2372					X	X	X		Canada	
AGAKL1018-17 Apanteles carpatus Canada BOLD AAC2372					X	X	X		Canada	
AGAKL1808-17 Apanteles carpatus Canada BOLD AAC2372					X	X	X		Canada	
BARSE446-16 Apanteles carpatus Canada BOLD AAC2372					X	X	X		Canada	
BRDNT090-19 Apanteles carpatus Canada BOLD AAC2372					X	X	X		Canada	
BRDNT075-19 Apanteles carpatus Canada BOLD AAC2372					X	X	X		Canada	
BRDNT091-19 Apanteles carpatus Canada BOLD AAC2372					X	X	X		Canada	
BRDNT089-19 Apanteles carpatus Canada BOLD AAC2372					X	X	X		Canada	
BRDNT092-19 Apanteles carpatus Canada BOLD AAC2372					X	X	X		Canada	
BRDNT074-19 Apanteles carpatus Canada BOLD AAC2372					X	X	X		Canada	
AUMIC877-23 Microgastrinae Australia BOLD AAC2372									South Australia	
AUMIC864-23 Microgastrinae Australia BOLD AAC2372									South Australia	
AGAKE252-17 Apanteles carpatus Canada BOLD AAC2372					X	X	X		Canada	
AUMIC062-18 Apanteles carpatus Australia BOLD AAC2372					X	X	X		South Australia	
CNCHZ307-09 Apanteles carpatus Canada BOLD AAC2372					X	X	X		Canada	
OZBOL6285-22 Microgastrinae Australia BOLD AAC2372					X	X	X		South Australia	
SMTPI7180-14 Apanteles carpatus Canada BOLD AAC2372					X	X	X		Canada	
SMTPI7075-14 Apanteles carpatus Canada BOLD AAC2372					X	X	X		Canada	
NZMG198-11 Apanteles carpatus New Zealand BOLD AAC2372					X	X	X		New Zealand	
NZMG199-11 Apanteles carpatus New Zealand BOLD AAC2372					X	X	X		New Zealand	
BARSF512-16 Apanteles carpatus Canada BOLD AAC2372					X	X	X		Canada	
SMTPI6971-14 Apanteles carpatus Canada BOLD AAC2372					X	X	X		Canada	
CNCHZ232-09 Apanteles carpatus Canada BOLD AAC2372					X	X	X		Canada	
CNCHZ213-09 Apanteles carpatus Canada BOLD AAC2372					X	X	X		Canada	
CNCHZ211-09 Apanteles carpatus Canada BOLD AAC2372					X	X	X		Canada	
CNCHZ210-09 Apanteles carpatus Canada BOLD AAC2372					X	X	X		Canada	
CNCHZ226-09 Apanteles carpatus Canada BOLD AAC2372					X	X	X		Canada	
CNCHZ243-09 Apanteles carpatus Canada BOLD AAC2372					X	X	X		Canada	
CNCHZ248-09 Apanteles carpatus Canada BOLD AAC2372					X	X	X		Canada	
ASMII13755-22 Apanteles Australia BOLD ADL3302					X	X	X	*A. apricus* / MRSB05	Western Australia	7/7
AUMIC068-18 Apanteles Australia BOLD ADL3302									Queensland	
AUMIC972-24 Braconidae Australia BOLD AAM7397								*A. cuprum* / MRSB09	Queensland	7/7
ASMII9771-22 Microgastrinae Australia BOLD AAM7397					X	X	X		Western Australia	
HYQT498-10 Microgastrinae Australia BOLD AAM7397					X	X	X		Queensland	
HYQT508-10 Microgastrinae Australia BOLD AAM7397					X	X	X		Queensland	
HYQT668-10 Microgastrinae Australia BOLD AAM7397					X	X	X		Queensland	
AUMIC1395-24 Braconidae Australia BOLD AAM7397					X	X	X		Northern Territory	
HYQT198-09 Microgastrinae Australia BOLD AAM7397					X	X	X		Queensland	
HYQTB354-12 Microgastrinae Australia BOLD AAM7397					X	X	X		Queensland	
ASMII3877-22 Microgastrinae Australia BOLD AAM7397					X	X	X		Queensland	
ASMII12307-22 Microgastrinae Australia BOLD AAM7397					X	X	X		Queensland	
HYQT374-10 Apanteles Australia BOLD AAG8155					X	X	X	*A. aurantius* / MRSB10	Queensland	7/7
AUMIC238-18 Apanteles Australia BOLD AAG8155									New South Wales	
AUMIC859-23 Microgastrinae Australia BOLD AAG8155									South Australia	
AUMIC1214-24 Braconidae Australia BOLD AAG8155									Queensland	
AUMIC1063-24 Braconidae Australia BOLD AAG8155									Queensland	
AUMIC411-18 Apanteles Australia BOLD AAG8155									Queensland	
AUMIC088-18 Apanteles Australia BOLD AAG8155									Queensland	
OZBOL422-21 Microgastrinae Australia BOLD AAG8155					X	X	X		Queensland	
AACTA4990-20 Apanteles Australia BOLD AAG8155					X	X	X		Australian Capital Territory	
ASMII6217-22 Apanteles Australia BOLD AAG8155					X	X	X		Western Australia	
GCQT1101-17 Apanteles Australia BOLD AAG8155					X	X	X		Queensland	
HYQTB145-12 Apanteles Australia BOLD AAG8155					X	X	X		Queensland	
HYQT277-09 Apanteles Australia BOLD AAG8155					X	X	X		Queensland	
HYQTB106-11 Apanteles Australia BOLD AAG8155					X	X	X		Queensland	
HYQT276-09 Apanteles Australia BOLD AAG8155					X	X	X		Queensland	
VAQT711-09 Apanteles Australia BOLD AAG8155					X	X	X		Queensland	
HYQT261-09 Apanteles Australia BOLD AAG8155					X	X	X		Queensland	
ASMII204-22 Apanteles Australia BOLD AAG8155					X	X	X		Queensland	
ASMII6178-22 Apanteles Australia BOLD AAG8155					X	X	X		Western Australia	
HYQT226-09 Apanteles Australia BOLD AAG8155					X	X	X		Queensland	
GCQT1055-17 Apanteles Australia BOLD AAG8155					X	X	X		Queensland	
AUMIC1235-24 Braconidae Australia BOLD AAZ1975								MRSB06	Queensland	5/7
AUMIC1236-24 Braconidae Australia BOLD AAZ1975									Queensland	
HYQTB014-11 Apanteles Australia BOLD AAZ1975					X	X	X		Queensland	
GBMNA31586-19 Iconella jason BOLD AAH1089					X	X	X	MRSB07	Unknown (Genbank)	5/7
CNCHW725-09 Apanteles Thailand BOLD AAH1089					X	X	X		Thailand	
GMBCB2248-15 Apanteles Bangladesh BOLD AAH1089					X	X	X		Bangladesh	
GMMBI660-16 Apanteles Malaysia BOLD AAH1089					X	X	X		Malaysia	
CNCHW473-09 Apanteles Thailand BOLD AAH1089					X	X	X		Thailand	
ASPNI700-09 Apanteles Papua New Guinea BOLD AAH1089					X	X	X		Papua New Guinea	
GMIAK837-17 Apanteles Indonesia BOLD AAH1089					X	X	X		Indonesia	
GMIAE643-17 Apanteles Indonesia BOLD AAH1089					X	X	X		Indonesia	
GMIAF057-17 Apanteles Indonesia BOLD AAH1089					X	X	X		Indonesia	
GMIAD888-17 Apanteles Indonesia BOLD AAH1089					X	X	X		Indonesia	

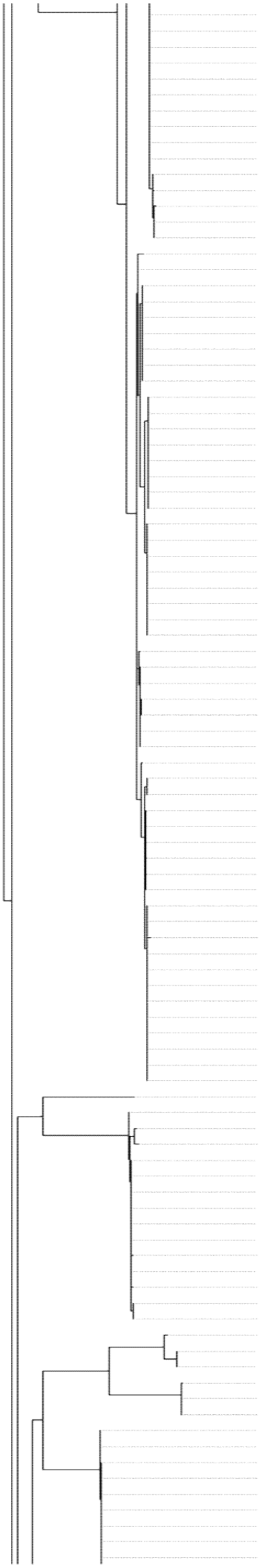

GMIAI114-17_Apanteles_Indonesia_BOLD_AAH1089	X	X	X		Indonesia	
GMMBI979-16_Apanteles_Malaysia_BOLD_AAH1089	X	X	X		Malaysia	
GMIAK7671-18_Apanteles_Indonesia_BOLD_AAH1089	X	X	X		Indonesia	
GMIAB619-17_Apanteles_Indonesia_BOLD_AAH1089	X	X	X		Indonesia	
GMIAH671-17_Apanteles_Indonesia_BOLD_AAH1089	X	X	X		Indonesia	
GMIAK347-17_Apanteles_Indonesia_BOLD_AAH1089	X	X	X		Indonesia	
GMIAG495-17_Apanteles_Indonesia_BOLD_AAH1089	X	X	X		Indonesia	
ASPNI615-09_Apanteles_Papua_New_Guinea_BOLD_AAH1089	X	X	X		Papua New Guinea	
GBMNA31584-19_Apanteles_melpomene__BOLD_AAH1089	X	X	X		Unknown (Genbank)	
GBMNA31579-19_Apanteles_melpomene__BOLD_AAH1089	X	X	X		Unknown (Genbank)	
AUMIC047-18_Apanteles_Australia_BOLD_AAH1089					Northern Territory	
AUMIC044-18_Apanteles_Australia_BOLD_AAH1089					Northern Territory	
OZBOL460-21_Microgastrinae_Australia_BOLD_AAH1089	X	X	X		Northern Territory	
HYQTB094-11_Apanteles_Australia_BOLD_AAH1089	X	X	X		Queensland	
HYQTB088-11_Apanteles_Australia_BOLD_AAH1089	X	X	X		Queensland	
GBMNA31588-19_Apanteles_sp._6_SY-2018__BOLD_AAH1201	X	X	X	MRSB08	Unknown (Genbank)	7/7
GMBCF3919-15_Apanteles_Bangladesh_BOLD_AAH1201	X	X	X		Bangladesh	
GMBCF3974-15_Apanteles_Bangladesh_BOLD_AAH1201	X	X	X		Bangladesh	
GMBCK705-15_Apanteles_Bangladesh_BOLD_AAH1201	X	X	X		Bangladesh	
GMBCH2266-15_Apanteles_Bangladesh_BOLD_AAH1201	X	X	X		Bangladesh	
GMBCD208-15_Apanteles_Bangladesh_BOLD_AAH1201	X	X	X		Bangladesh	
GMBCD259-15_Apanteles_Bangladesh_BOLD_AAH1201	X	X	X		Bangladesh	
GMBCD1601-15_Apanteles_Bangladesh_BOLD_AAH1201	X	X	X		Bangladesh	
GMBCJ2896-15_Apanteles_Bangladesh_BOLD_AAH1201	X	X	X		Bangladesh	
GMBCF3794-15_Apanteles_Bangladesh_BOLD_AAH1201	X	X	X		Bangladesh	
GMBCF3764-15_Apanteles_Bangladesh_BOLD_AAH1201	X	X	X		Bangladesh	
GMBCI2849-15_Apanteles_Bangladesh_BOLD_AAH1201	X	X	X		Bangladesh	
GMBCI3151-15_Apanteles_Bangladesh_BOLD_AAH1201	X	X	X		Bangladesh	
GMBCE065-15_Apanteles_Bangladesh_BOLD_AAH1201	X	X	X		Bangladesh	
GMBCF4089-15_Apanteles_Bangladesh_BOLD_AAH1201	X	X	X		Bangladesh	
GMBCM316-15_Apanteles_Bangladesh_BOLD_AAH1201	X	X	X		Bangladesh	
GMBCC1588-15_Apanteles_Bangladesh_BOLD_AAH1201	X	X	X		Bangladesh	
GMBCF3814-15_Apanteles_Bangladesh_BOLD_AAH1201	X	X	X		Bangladesh	
GMBCG2067-15_Apanteles_Bangladesh_BOLD_AAH1201	X	X	X		Bangladesh	
GMBCG2112-15_Apanteles_Bangladesh_BOLD_AAH1201	X	X	X		Bangladesh	
MAMTV072-14_Apanteles_PK03_Pakistan_BOLD_AAH1201	X	X	X		Pakistan	
GMBCG393-15_Apanteles_Bangladesh_BOLD_AAH1201	X	X	X		Bangladesh	
GMBCF3992-15_Apanteles_Bangladesh_BOLD_AAH1201	X	X	X		Bangladesh	
GMBCG371-15_Apanteles_Bangladesh_BOLD_AAH1201	X	X	X		Bangladesh	
GMBCF3996-15_Apanteles_Bangladesh_BOLD_AAH1201	X	X	X		Bangladesh	
GMBCH2629-15_Apanteles_Bangladesh_BOLD_AAH1201	X	X	X		Bangladesh	
GMBCH2223-15_Apanteles_Bangladesh_BOLD_AAH1201	X	X	X		Bangladesh	
GMBCD1125-15_Apanteles_Bangladesh_BOLD_AAH1201	X	X	X		Bangladesh	
CNCHW484-09_Apanteles_Thailand_BOLD_AAH1201	X	X	X		Thailand	
CNCHW470-09_Apanteles_Thailand_BOLD_AAH1201	X	X	X		Thailand	
WOMIA370-11_Apanteles_China_BOLD_AAH1201	X	X	X		China	
WOMIA368-11_Apanteles_China_BOLD_AAH1201	X	X	X		China	
CNCBA159-10_Apanteles_South_Korea_BOLD_AAH1201	X	X	X		South Korea	
GMBCB2104-15_Apanteles_Bangladesh_BOLD_AAH1201	X	X	X		Bangladesh	
GMBCK221-15_Apanteles_Bangladesh_BOLD_AAH1201	X	X	X		Bangladesh	
AUMIC1502-24_Braconidae_Australia_BOLD_AAH1201					Queensland	
AUMIC159-18_Apanteles_Australia_BOLD_AAH1201					Queensland	
AUMIC486-18_Apanteles_Australia_BOLD_AAH1201					Queensland	
AUMIC1495-24_Braconidae_Australia_BOLD_AAH1201					Queensland	
AUMIC1507-24_Braconidae_Australia_BOLD_AAH1201					Queensland	
AUMIC1479-24_Braconidae_Australia_BOLD_AAH1201					Queensland	
GBMNA31590-19_Glyptapanteles_merope__BOLD_AAH1201	X	X	X		Unknown (Genbank)	
GBMNA31576-19_Glyptapanteles_merope__BOLD_AAH1201	X	X	X		Unknown (Genbank)	
GBAH22739-19_Apanteles_sp._AAZ_054__BOLD_AAH1201	X	X	X		Unknown (Genbank)	
GMMGN1375-14_Apanteles_Malaysia_BOLD_AAH1201	X	X	X		Malaysia	
GMBCJ2515-15_Apanteles_Bangladesh_BOLD_AAH1201	X	X	X		Bangladesh	
GMMBI612-16_Apanteles_Malaysia_BOLD_AAH1201	X	X	X		Malaysia	
CNCHW722-09_Apanteles_Thailand_BOLD_AAH1201	X	X	X		Thailand	
GMMBC214-16_Apanteles_Malaysia_BOLD_AAH1201	X	X	X		Malaysia	
GMIAB592-17_Apanteles_Indonesia_BOLD_AAH1201	X	X	X		Indonesia	
GMMBP374-16_Apanteles_Malaysia_BOLD_AAH1201	X	X	X		Malaysia	
GMMBI1009-16_Apanteles_Malaysia_BOLD_AAH1201	X	X	X		Malaysia	
GMMBI613-16_Apanteles_Malaysia_BOLD_AAH1201	X	X	X		Malaysia	
AUMIC081-18_Apanteles_Australia_BOLD_ADI5121				*A. breviflagellarius* / MRSB11	Queensland	7/7
MAHYM475-10_Apanteles_galleriae_Pakistan_BOLD_AAG1400	X	X	X	*A. galleriae*	Pakistan	4/4
GBAH20789-19_Apanteles_galleriae_Argentina_BOLD_AAG1400	X	X	X		Argentina	
GBAH22366-19_Apanteles_galleriae_India_BOLD_AAG1400	X	X	X		India	
GMMDK2665-15_Apanteles_galleriae_Madagascar_BOLD_AAG1400	X	X	X		Madagascar	
GBDTA392-21_Apanteles_galleriae_Germany_BOLD_AAG1400	X	X	X		Germany	
GBDTA391-21_Apanteles_galleriae_Germany_BOLD_AAG1400	X	X	X		Germany	
GBDTA14497-21_Apanteles_galleriae_Germany_BOLD_AAG1400	X	X	X		Germany	
MARKB239-21_Apanteles_galleriae_United_Kingdom_BOLD_AAG1400	X	X	X		United Kingdom	
GBAH20787-19_Apanteles_galleriae__BOLD_AAG1400	X	X	X		Unknown (Genbank)	
NZMG202-11_Apanteles_galleriae_New_Zealand_BOLD_AAG1400	X	X	X		New Zealand	
MARKB091-21_Apanteles_galleriae_Malta_BOLD_AAG1400	X	X	X		Malta	
GBAH20788-19_Apanteles_galleriae__BOLD_AAG1400	X	X	X		Unknown (Genbank)	
NZMG203-11_Apanteles_galleriae_New_Zealand_BOLD_AAG1400	X	X	X		New Zealand	
NZMG201-11_Apanteles_galleriae_New_Zealand_BOLD_AAG1400	X	X	X		New Zealand	
AUMIC1233-24_Braconidae_Australia_BOLD_ADL3948				*A. kelpiellus* / MRSB13	Queensland	5/7
AUMIC334-18_Apanteles_Australia_BOLD_ADL3948					Queensland	
AUMIC330-18_Apanteles_Australia_BOLD_ADL3948					Queensland	
AUMIC282-18_Apanteles_Australia_BOLD_ADL4093				*A. margaritarius* / MRSB12	New South Wales	6/7
AUMIC1239-24_Braconidae_Australia_BOLD_ADL4093					New South Wales	
AUMIC1148-24_Braconidae_Australia_BOLD_ADL4093					Australian Capital Territory	
GBMIN74050-17_Apanteles_subandinus__BOLD_AAV2170	X	X	X	*A. subandinus*	Unknown (Genbank)	4/4
GBMIN74052-17_Apanteles_subandinus__BOLD_AAV2170	X	X	X		Unknown (Genbank)	
NZMG206-11_Apanteles_subandinus_New_Zealand_BOLD_AAV2170	X	X	X		New Zealand	
NZMG227-11_Apanteles_subandinus_New_Zealand_BOLD_AAV2170	X	X	X		New Zealand	
NZMG205-11_Apanteles_subandinus_New_Zealand_BOLD_AAV2170	X	X	X		New Zealand	
GBAH22743-19_Apanteles_subandinus__BOLD_AAV2170	X	X	X		Unknown (Genbank)	
WOMIA182-11_Apanteles_subandinus_Colombia_BOLD_AAV2170	X	X	X		Colombia	
GBMIN74053-17_Apanteles_subandinus__BOLD_AAV2170	X	X	X		Unknown (Genbank)	
GMSAW916-14_Apanteles_subandinus_South_Africa_BOLD_AAV2170	X	X	X		South Africa	

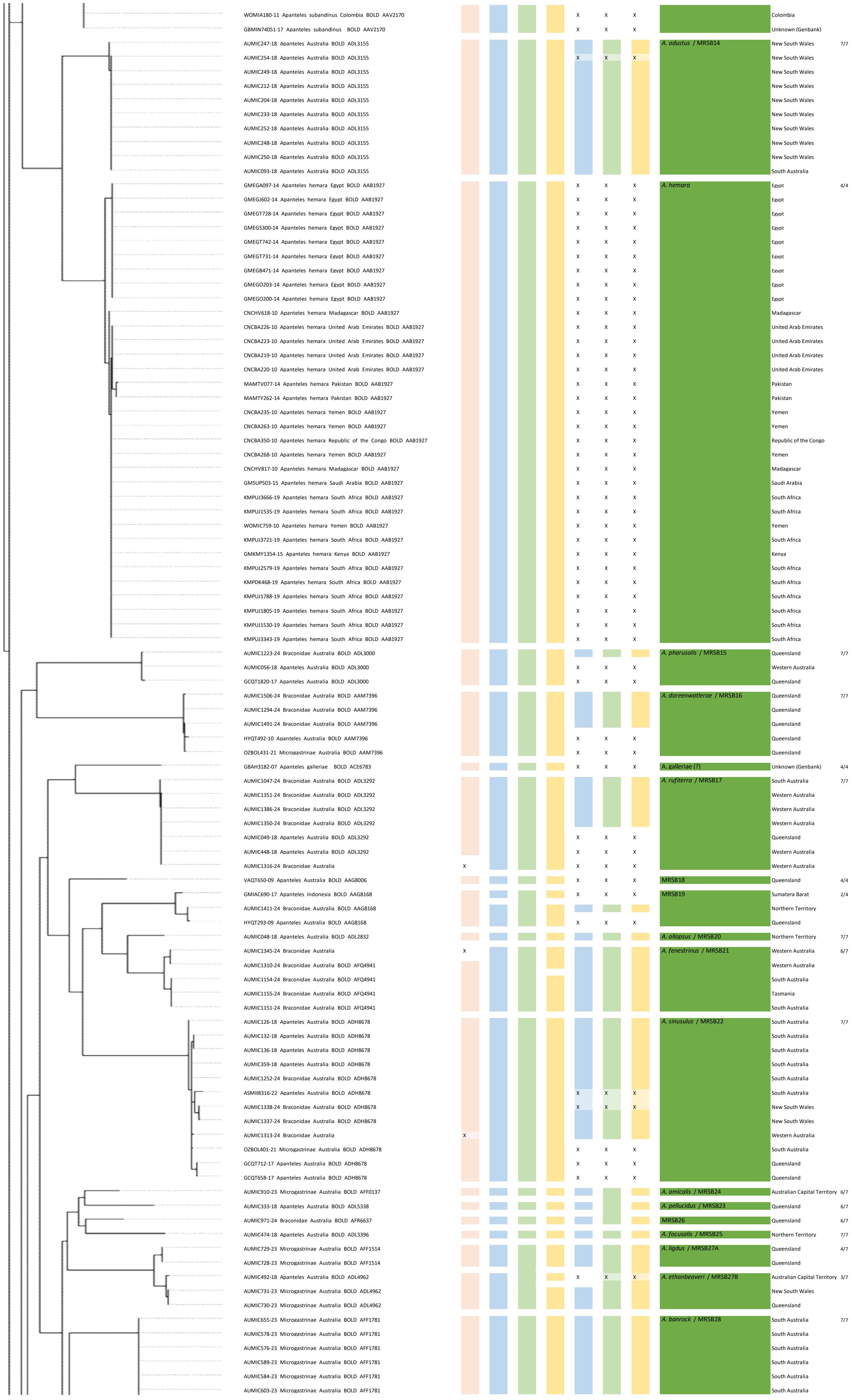
WOMIA180-11 Apanteles subandinus Colombia BOLD AAV2170 X X X Colombia
GBMIN74051-17 Apanteles subandinus BOLD AAV2170 X X X Unknown (Genbank)
AUMIC247-18 Apanteles Australia BOLD ADL3155 A. adustus / MRSB14 New South Wales 7/7
AUMIC254-18 Apanteles Australia BOLD ADL3155 X X X New South Wales
AUMIC249-18 Apanteles Australia BOLD ADL3155 New South Wales
AUMIC212-18 Apanteles Australia BOLD ADL3155 New South Wales
AUMIC204-18 Apanteles Australia BOLD ADL3155 New South Wales
AUMIC233-18 Apanteles Australia BOLD ADL3155 New South Wales
AUMIC252-18 Apanteles Australia BOLD ADL3155 New South Wales
AUMIC248-18 Apanteles Australia BOLD ADL3155 New South Wales
AUMIC250-18 Apanteles Australia BOLD ADL3155 New South Wales
AUMIC093-18 Apanteles Australia BOLD ADL3155 South Australia
GMEGA097-14 Apanteles hemara Egypt BOLD AAB1927 X X X A. hemara Egypt 4/4
GMEGJ602-14 Apanteles hemara Egypt BOLD AAB1927 X X X Egypt
GMEGT728-14 Apanteles hemara Egypt BOLD AAB1927 X X X Egypt
GMEGS300-14 Apanteles hemara Egypt BOLD AAB1927 X X X Egypt
GMEGT742-14 Apanteles hemara Egypt BOLD AAB1927 X X X Egypt
GMEGT731-14 Apanteles hemara Egypt BOLD AAB1927 X X X Egypt
GMEGB471-14 Apanteles hemara Egypt BOLD AAB1927 X X X Egypt
GMEGO203-14 Apanteles hemara Egypt BOLD AAB1927 X X X Egypt
GMEGO200-14 Apanteles hemara Egypt BOLD AAB1927 X X X Egypt
CNCHV618-10 Apanteles hemara Madagascar BOLD AAB1927 X X X Madagascar
CNCBA226-10 Apanteles hemara United Arab Emirates BOLD AAB1927 X X X United Arab Emirates
CNCBA223-10 Apanteles hemara United Arab Emirates BOLD AAB1927 X X X United Arab Emirates
CNCBA219-10 Apanteles hemara United Arab Emirates BOLD AAB1927 X X X United Arab Emirates
CNCBA220-10 Apanteles hemara United Arab Emirates BOLD AAB1927 X X X United Arab Emirates
MAMTV077-14 Apanteles hemara Pakistan BOLD AAB1927 X X X Pakistan
MAMTY262-14 Apanteles hemara Pakistan BOLD AAB1927 X X X Pakistan
CNCBA235-10 Apanteles hemara Yemen BOLD AAB1927 X X X Yemen
CNCBA263-10 Apanteles hemara Yemen BOLD AAB1927 X X X Yemen
CNCBA350-10 Apanteles hemara Republic of the Congo BOLD AAB1927 X X X Republic of the Congo
CNCBA268-10 Apanteles hemara Yemen BOLD AAB1927 X X X Yemen
CNCHV817-10 Apanteles hemara Madagascar BOLD AAB1927 X X X Madagascar
GMSUP503-15 Apanteles hemara Saudi Arabia BOLD AAB1927 X X X Saudi Arabia
KMPUJ3666-19 Apanteles hemara South Africa BOLD AAB1927 X X X South Africa
KMPUJ1535-19 Apanteles hemara South Africa BOLD AAB1927 X X X South Africa
WOMIC759-10 Apanteles hemara Yemen BOLD AAB1927 X X X Yemen
KMPUJ3721-19 Apanteles hemara South Africa BOLD AAB1927 X X X South Africa
GMKMY1354-15 Apanteles hemara Kenya BOLD AAB1927 X X X Kenya
KMPUJ2579-19 Apanteles hemara South Africa BOLD AAB1927 X X X South Africa
KMPDK468-19 Apanteles hemara South Africa BOLD AAB1927 X X X South Africa
KMPUJ1788-19 Apanteles hemara South Africa BOLD AAB1927 X X X South Africa
KMPUJ1805-19 Apanteles hemara South Africa BOLD AAB1927 X X X South Africa
KMPUJ1530-19 Apanteles hemara South Africa BOLD AAB1927 X X X South Africa
KMPUJ3343-19 Apanteles hemara South Africa BOLD AAB1927 X X X South Africa
AUMIC1223-24 Braconidae Australia BOLD ADL3000 A. pharusalis / MRSB15 Queensland 7/7
AUMIC056-18 Apanteles Australia BOLD ADL3000 X X X Western Australia
GCQT1820-17 Apanteles Australia BOLD ADL3000 X X X Queensland
AUMIC1506-24 Braconidae Australia BOLD AAM7396 A. doreenwatlerae / MRSB16 Queensland 7/7
AUMIC1294-24 Braconidae Australia BOLD AAM7396 Queensland
AUMIC1491-24 Braconidae Australia BOLD AAM7396 Queensland
HYQT492-10 Apanteles Australia BOLD AAM7396 X X X Queensland
OZBOL431-21 Microgastrinae Australia BOLD AAM7396 X X X Queensland
GBAH3182-07 Apanteles galleriae BOLD ACE6783 X X X A. galleriae (?) Unknown (Genbank) 4/4
AUMIC1047-24 Braconidae Australia BOLD ADL3292 A. rufiterra / MRSB17 South Australia 7/7
AUMIC1351-24 Braconidae Australia BOLD ADL3292 Western Australia
AUMIC1386-24 Braconidae Australia BOLD ADL3292 Western Australia
AUMIC1350-24 Braconidae Australia BOLD ADL3292 Western Australia
AUMIC049-18 Apanteles Australia BOLD ADL3292 X X X Queensland
AUMIC448-18 Apanteles Australia BOLD ADL3292 X X X Western Australia
AUMIC1316-24 Braconidae Australia X X X X Western Australia
VAQT650-09 Apanteles Australia BOLD AAG8006 X X X MRSB18 Queensland 4/4
GMIAC690-17 Apanteles Indonesia BOLD AAG8168 X X X MRSB19 Sumatera Barat 2/4
AUMIC1411-24 Braconidae Australia BOLD AAG8168 Northern Territory
HYQT293-09 Apanteles Australia BOLD AAG8168 X X X Queensland
AUMIC048-18 Apanteles Australia BOLD ADL2832 A. allapsus / MRSB20 Northern Territory 7/7
AUMIC1345-24 Braconidae Australia X A. fenestrinus / MRSB21 Western Australia 6/7
AUMIC1310-24 Braconidae Australia BOLD AFQ4941 Western Australia
AUMIC1154-24 Braconidae Australia BOLD AFQ4941 South Australia
AUMIC1155-24 Braconidae Australia BOLD AFQ4941 Tasmania
AUMIC1151-24 Braconidae Australia BOLD AFQ4941 South Australia
AUMIC126-18 Apanteles Australia BOLD ADH8678 A. sinusulus / MRSB22 South Australia 7/7
AUMIC132-18 Apanteles Australia BOLD ADH8678 South Australia
AUMIC136-18 Apanteles Australia BOLD ADH8678 South Australia
AUMIC359-18 Apanteles Australia BOLD ADH8678 South Australia
AUMIC1252-24 Braconidae Australia BOLD ADH8678 South Australia
ASMII8316-22 Apanteles Australia BOLD ADH8678 X X X South Australia
AUMIC1338-24 Braconidae Australia BOLD ADH8678 X X X New South Wales
AUMIC1337-24 Braconidae Australia BOLD ADH8678 New South Wales
AUMIC1313-24 Braconidae Australia X Western Australia
OZBOL401-21 Microgastrinae Australia BOLD ADH8678 X X X South Australia
GCQT712-17 Apanteles Australia BOLD ADH8678 X X X Queensland
GCQT658-17 Apanteles Australia BOLD ADH8678 X X X Queensland
AUMIC910-23 Microgastrinae Australia BOLD AFF0137 A. amicalis / MRSB24 Australian Capital Territory 6/7
AUMIC333-18 Apanteles Australia BOLD ADL5338 A. pellucidus / MRSB23 Queensland 6/7
AUMIC971-24 Braconidae Australia BOLD AFR6637 MRSB26 Queensland 6/7
AUMIC474-18 Apanteles Australia BOLD ADL3396 A. focusalis / MRSB25 Northern Territory 7/7
AUMIC729-23 Microgastrinae Australia BOLD AFF1514 A. ligdus / MRSB27A Queensland 4/7
AUMIC728-23 Microgastrinae Australia BOLD AFF1514 Queensland
AUMIC492-18 Apanteles Australia BOLD ADL4962 X X X A. ethanbeaveri / MRSB27B Australian Capital Territory 3/7
AUMIC731-23 Microgastrinae Australia BOLD ADL4962 New South Wales
AUMIC730-23 Microgastrinae Australia BOLD ADL4962 Queensland
AUMIC655-23 Microgastrinae Australia BOLD AFF1781 A. banrock / MRSB28 South Australia 7/7
AUMIC578-23 Microgastrinae Australia BOLD AFF1781 South Australia
AUMIC576-23 Microgastrinae Australia BOLD AFF1781 South Australia
AUMIC589-23 Microgastrinae Australia BOLD AFF1781 South Australia
AUMIC584-23 Microgastrinae Australia BOLD AFF1781 South Australia
AUMIC603-23 Microgastrinae Australia BOLD AFF1781 South Australia

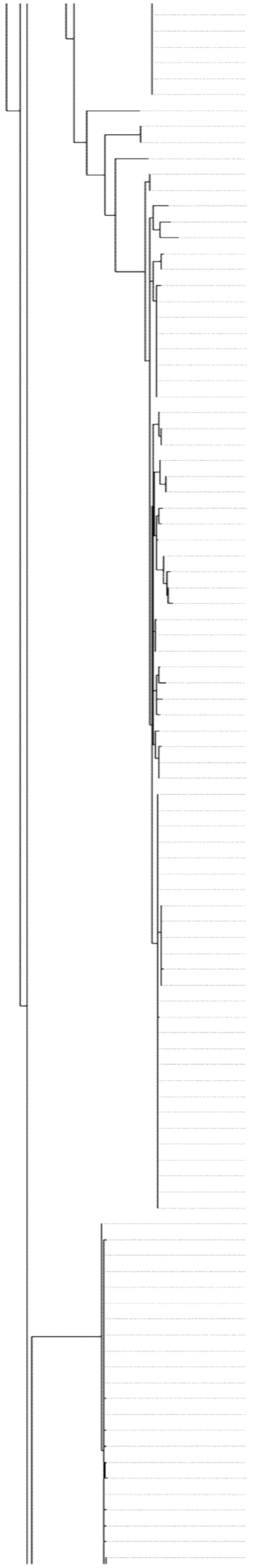

AUMIC591-23_Microgastrinae_Australia_BOLD_AFF1781									South Australia	
AUMIC590-23_Microgastrinae_Australia_BOLD_AFF1781									South Australia	
AUMIC575-23_Microgastrinae_Australia_BOLD_AFF1781									South Australia	
AUMIC593-23_Microgastrinae_Australia_BOLD_AFF1781									South Australia	
AUMIC577-23_Microgastrinae_Australia_BOLD_AFF1781									South Australia	
AUMIC592-23_Microgastrinae_Australia_BOLD_AFF1781									South Australia	
AUMIC1157-24_Braconidae_Australia_BOLD_AFQ8413								MRSB29	New South Wales	7/7
AUHYM012-22_Braconidae_Australia_BOLD_AEZ9092					X	X	X	*A. aeternus* / MRSB30	Western Australia	5/7
AUMIC1220-24_Braconidae_Australia_BOLD_AEZ9092									Queensland	
AUMIC1230-24_Braconidae_Australia_BOLD_AFR5983								*A. translucentis* / MRSB31	Queensland	4/7
AUMIC186-18_Apanteles_Australia_BOLD_AAD2366					//////<br>//////			*A. oenone* / MRSB32	Western Australia	2/7
AUMIC1218-24_Braconidae_Australia_BOLD_AAD2366					//////				Queensland	
ASPNI727-09_Apanteles_Papua_New_Guinea_BOLD_AAD2366					X	X	X		Papua New Guinea	
ASPNI921-09_Apanteles_Papua_New_Guinea_BOLD_AAD2366					X	X	X		Papua New Guinea	
AUMIC103-18_Apanteles_Australia_BOLD_AFO8520					X	X	X		Northern Territory	
AUMIC478-18_Apanteles_Australia_BOLD_AAD2366									Northern Territory	
GCQT689-17_Apanteles_Australia_BOLD_AAD2366					X	X	X		Queensland	
AUMIC110-18_Apanteles_Australia_BOLD_AAD2366					X	X	X		Northern Territory	
AUMIC036-18_Apanteles_Australia_BOLD_AAD2366					X	X	X		Western Australia	
AUMIC473-18_Apanteles_Australia_BOLD_AAD2366					X	X	X		Northern Territory	
AUMIC862-23_Microgastrinae_Australia_BOLD_AAD2366									Northern Territory	
AUMIC1412-24_Braconidae_Australia_BOLD_AAD2366									Northern Territory	
AUMIC037-18_Apanteles_Australia_BOLD_AAD2366									Northern Territory	
AUMIC183-18_Apanteles_Australia_BOLD_AAD2366									Western Australia	
AUMIC034-18_Apanteles_Australia_BOLD_AAD2366									Queensland	
ASPN238-07_Apanteles_Papua_New_Guinea_BOLD_AAD2366					X	X	X		Madang	
ASPN250-07_Apanteles_Whitfield17_Papua_New_Guinea_BOLD_AAD2366					X	X	X		Madang	
ASPN243-07_Apanteles_Whitfield17_Papua_New_Guinea_BOLD_AAD2366					X	X	X		Madang	
ASPNI693-09_Apanteles_Papua_New_Guinea_BOLD_AAD2366					X	X	X		Papua New Guinea	
ASPNI913-09_Apanteles_Papua_New_Guinea_BOLD_AAD2366					X	X	X		Papua New Guinea	
ASPNI690-09_Apanteles_Papua_New_Guinea_BOLD_AAD2366					X	X	X		Papua New Guinea	
AUMIC1186-24_Braconidae_Australia_BOLD_AAD2366					//////<br>//////				Queensland	
ASPNI653-09_Apanteles_Papua_New_Guinea_BOLD_AAD2366					X	X	X		Papua New Guinea	
ASPNI564-09_Apanteles_Papua_New_Guinea_BOLD_AAD2366					X	X	X		Papua New Guinea	
AUMIC1212-24_Braconidae_Australia_BOLD_AAD2366					//////<br>//////				Queensland	
AUMIC1062-24_Braconidae_Australia_BOLD_AAD2366									Queensland	
AUMIC973-24_Braconidae_Australia_BOLD_AAD2366					X	X	X		Queensland	
AUMIC1135-24_Braconidae_Australia_BOLD_AAD2366									Queensland	
ASPNJ010-09_Apanteles_Papua_New_Guinea_BOLD_AAD2366					X	X	X		Papua New Guinea	
ASPNI748-09_Apanteles_Papua_New_Guinea_BOLD_AAD2366					X	X	X		Papua New Guinea	
ASPNI604-09_Apanteles_Papua_New_Guinea_BOLD_AAD2366					X	X	X		Papua New Guinea	
ASPNI677-09_Apanteles_Papua_New_Guinea_BOLD_AAD2366					X	X	X		Papua New Guinea	
ASPN574-09_Apanteles_Whitfield17_Papua_New_Guinea_BOLD_AAD2366					X	X	X		Papua New Guinea	
ASPNJ215-09_Apanteles_Papua_New_Guinea_BOLD_AAD2366					X	X	X		Papua New Guinea	
ASPN590-09_Apanteles_Whitfield17_Papua_New_Guinea_BOLD_AAD2366					X	X	X		Papua New Guinea	
ASPNI708-09_Apanteles_Papua_New_Guinea_BOLD_AAD2366					X	X	X		Papua New Guinea	
ASPN334-07_Apanteles_Whitfield17_Papua_New_Guinea_BOLD_AAD2366					X	X	X		Papua New Guinea	
ASPNI532-09_Apanteles_Papua_New_Guinea_BOLD_AAD2366					X	X	X		Papua New Guinea	
ASPN846-09_Apanteles_Whitfield17_Papua_New_Guinea_BOLD_AAD2366					X	X	X		Papua New Guinea	
ASPN676-09_Apanteles_Whitfield17_Papua_New_Guinea_BOLD_AAD2366					X	X	X		Papua New Guinea	
ASPN314-07_Apanteles_Whitfield17_Papua_New_Guinea_BOLD_AAD2366					X	X	X		Papua New Guinea	
ASPN354-07_Apanteles_Whitfield17_Papua_New_Guinea_BOLD_AAD2366					X	X	X		Papua New Guinea	
ASPN330-07_Apanteles_Whitfield17_Papua_New_Guinea_BOLD_AAD2366					X	X	X		Papua New Guinea	
ASPN368-07_Apanteles_Papua_New_Guinea_BOLD_AAD2366					X	X	X		Papua New Guinea	
ASPN366-07_Apanteles_Papua_New_Guinea_BOLD_AAD2366					X	X	X		Papua New Guinea	
ASPN295-07_Apanteles_Whitfield17_Papua_New_Guinea_BOLD_AAD2366					X	X	X		Papua New Guinea	
ASPNJ369-09_Apanteles_Papua_New_Guinea_BOLD_AAD2366					X	X	X		Papua New Guinea	
ASPNJ283-09_Apanteles_Papua_New_Guinea_BOLD_AAD2366					X	X	X		Papua New Guinea	
ASPNJ368-09_Apanteles_Papua_New_Guinea_BOLD_AAD2366					X	X	X		Papua New Guinea	
ASPN269-07_Apanteles_Papua_New_Guinea_BOLD_AAD2366					X	X	X		Madang	
ASPNJ142-09_Apanteles_Papua_New_Guinea_BOLD_AAD2366					X	X	X		Papua New Guinea	
ASPN360-07_Apanteles_Whitfield17_Papua_New_Guinea_BOLD_AAD2366					X	X	X		Papua New Guinea	
ASPN668-09_Apanteles_Whitfield17_Papua_New_Guinea_BOLD_AAD2366					X	X	X		Papua New Guinea	
ASPN656-09_Apanteles_Whitfield17_Papua_New_Guinea_BOLD_AAD2366					X	X	X		Papua New Guinea	
ASPNJ062-09_Apanteles_Papua_New_Guinea_BOLD_AAD2366					X	X	X		Papua New Guinea	
ASPNJ074-09_Apanteles_Papua_New_Guinea_BOLD_AAD2366					X	X	X		Papua New Guinea	
ASPNJ121-09_Apanteles_Papua_New_Guinea_BOLD_AAD2366					X	X	X		Papua New Guinea	
ASPNJ063-09_Apanteles_Papua_New_Guinea_BOLD_AAD2366					X	X	X		Papua New Guinea	
ASPNJ085-09_Apanteles_Papua_New_Guinea_BOLD_AAD2366					X	X	X		Papua New Guinea	
ASPN624-09_Apanteles_Whitfield17_Papua_New_Guinea_BOLD_AAD2366					X	X	X		Papua New Guinea	
ASPN662-09_Apanteles_Whitfield17_Papua_New_Guinea_BOLD_AAD2366					X	X	X		Papua New Guinea	
ASPN642-09_Apanteles_Whitfield17_Papua_New_Guinea_BOLD_AAD2366					X	X	X		Papua New Guinea	
ASPN632-09_Apanteles_Whitfield17_Papua_New_Guinea_BOLD_AAD2366					X	X	X		Papua New Guinea	
ASPN618-09_Apanteles_Whitfield17_Papua_New_Guinea_BOLD_AAD2366					X	X	X		Papua New Guinea	
ASPN674-09_Apanteles_Whitfield17_Papua_New_Guinea_BOLD_AAD2366					X	X	X		Papua New Guinea	
ASPN654-09_Apanteles_Whitfield17_Papua_New_Guinea_BOLD_AAD2366					X	X	X		Papua New Guinea	
AUMIC184-18_Apanteles_ippeus_Australia_BOLD_ACD8377					#####<br>#####			*A. ippeus* / MRSB33	Western Australia	6/7
AUMIC735-23_Microgastrinae_Australia_BOLD_ACD8377					#####<br>#####				South Australia	
OZBOL397-21_Microgastrinae_Australia_BOLD_ACD8377					X	X	X		South Australia	
AUMIC328-18_Apanteles_ippeus_Australia_BOLD_ACD8377					X	X	X		South Australia	
AUMIC323-18_Apanteles_ippeus_Australia_BOLD_ACD8377					X	X	X		South Australia	
AUMIC312-18_Apanteles_ippeus_Australia_BOLD_ACD8377					X	X	X		South Australia	
AUMIC285-18_Apanteles_ippeus_Australia_BOLD_ACD8377					X	X	X		South Australia	
AUMIC516-18_Apanteles_ippeus_Australia_BOLD_ACD8377					X	X	X		South Australia	
AUMIC319-18_Apanteles_ippeus_Australia_BOLD_ACD8377					X	X	X		South Australia	
OZBOL6321-22_Microgastrinae_Australia_BOLD_ACD8377					X	X	X		South Australia	
OZBOL6319-22_Microgastrinae_Australia_BOLD_ACD8377					X	X	X		South Australia	
AUMIC1355-24_Braconidae_Australia_BOLD_ACD8377					X	X	X		Western Australia	
ASMII7555-22_Apanteles_ippeus_Australia_BOLD_ACD8377					X	X	X		South Australia	
AUMIC1317-24_Braconidae_Australia_BOLD_ACD8377					X	X	X		Western Australia	
OZBOL440-21_Microgastrinae_Australia_BOLD_ACD8377					X	X	X		New South Wales	
AUMIC329-18_Apanteles_ippeus_Australia_BOLD_ACD8377					X	X	X		South Australia	
AUMIC1299-24_Braconidae_Australia_BOLD_ACD8377									Western Australia	
AUMIC1533-24_Braconidae_Australia_BOLD_ACD8377					#####<br>#####				Australian Capital Territory	
AUMIC513-18_Apanteles_ippeus_Australia_BOLD_ACD8377					#####<br>#####				South Australia	
AUMIC1319-24_Braconidae_Australia_BOLD_ACD8377					#####<br>#####				Western Australia	
AUMIC1143-24_Braconidae_Australia_BOLD_ACD8377					#####				Queensland	
AUMIC1301-24_Braconidae_Australia	X	X	X	X					Western Australia	

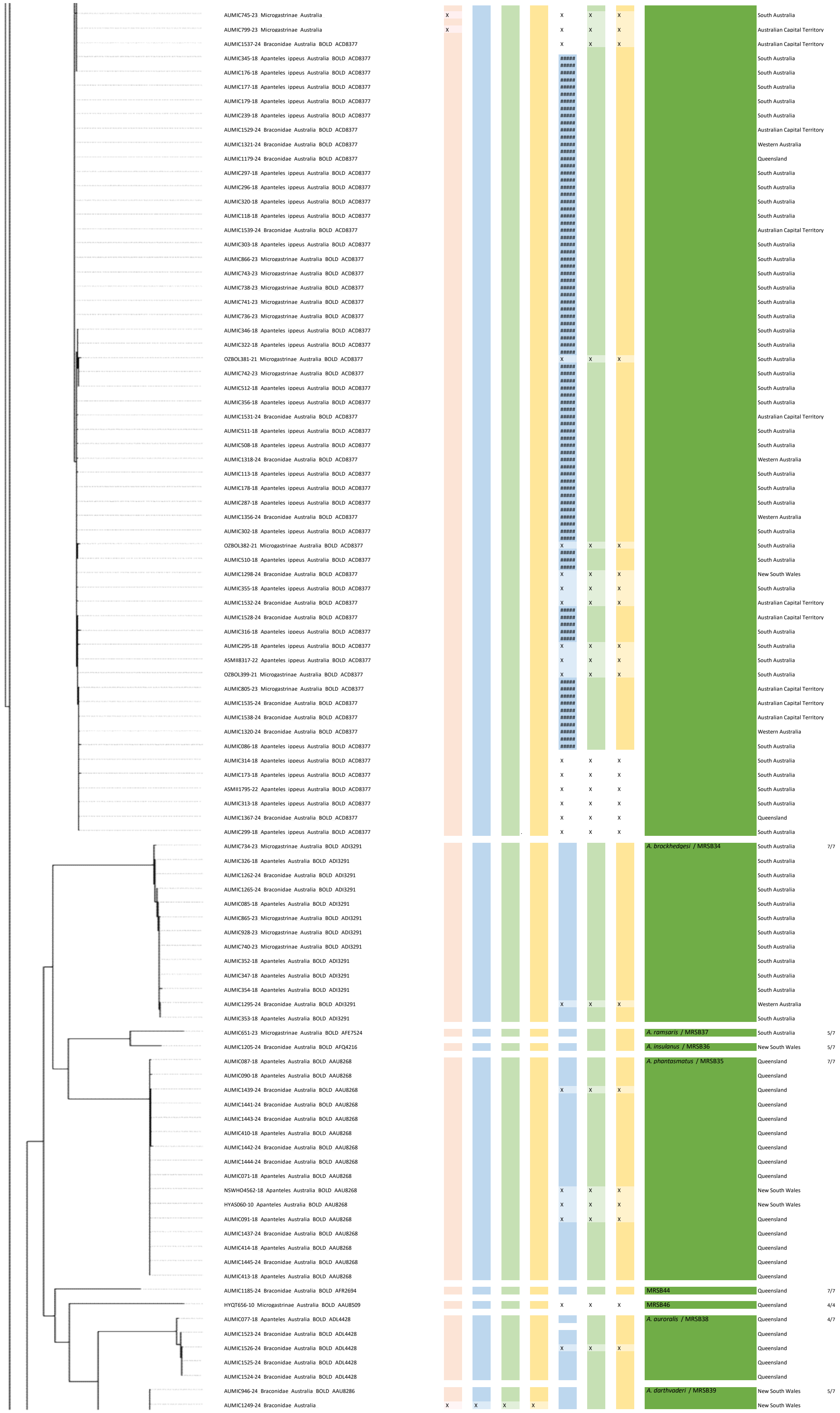

AUMIC745-23_Microgastrinae_Australia South Australia
AUMIC799-23_Microgastrinae_Australia Australian Capital Territory
AUMIC1537-24_Braconidae_Australia_BOLD_ACD8377 Australian Capital Territory
AUMIC345-18_Apanteles_ippeus_Australia_BOLD_ACD8377 South Australia
AUMIC176-18_Apanteles_ippeus_Australia_BOLD_ACD8377 South Australia
AUMIC177-18_Apanteles_ippeus_Australia_BOLD_ACD8377 South Australia
AUMIC179-18_Apanteles_ippeus_Australia_BOLD_ACD8377 South Australia
AUMIC239-18_Apanteles_ippeus_Australia_BOLD_ACD8377 South Australia
AUMIC1529-24_Braconidae_Australia_BOLD_ACD8377 Australian Capital Territory
AUMIC1321-24_Braconidae_Australia_BOLD_ACD8377 Western Australia
AUMIC1179-24_Braconidae_Australia_BOLD_ACD8377 Queensland
AUMIC297-18_Apanteles_ippeus_Australia_BOLD_ACD8377 South Australia
AUMIC296-18_Apanteles_ippeus_Australia_BOLD_ACD8377 South Australia
AUMIC320-18_Apanteles_ippeus_Australia_BOLD_ACD8377 South Australia
AUMIC118-18_Apanteles_ippeus_Australia_BOLD_ACD8377 South Australia
AUMIC1539-24_Braconidae_Australia_BOLD_ACD8377 Australian Capital Territory
AUMIC303-18_Apanteles_ippeus_Australia_BOLD_ACD8377 South Australia
AUMIC866-23_Microgastrinae_Australia_BOLD_ACD8377 South Australia
AUMIC743-23_Microgastrinae_Australia_BOLD_ACD8377 South Australia
AUMIC738-23_Microgastrinae_Australia_BOLD_ACD8377 South Australia
AUMIC741-23_Microgastrinae_Australia_BOLD_ACD8377 South Australia
AUMIC736-23_Microgastrinae_Australia_BOLD_ACD8377 South Australia
AUMIC346-18_Apanteles_ippeus_Australia_BOLD_ACD8377 South Australia
AUMIC322-18_Apanteles_ippeus_Australia_BOLD_ACD8377 South Australia
OZBOL381-21_Microgastrinae_Australia_BOLD_ACD8377 South Australia
AUMIC742-23_Microgastrinae_Australia_BOLD_ACD8377 South Australia
AUMIC512-18_Apanteles_ippeus_Australia_BOLD_ACD8377 South Australia
AUMIC356-18_Apanteles_ippeus_Australia_BOLD_ACD8377 South Australia
AUMIC1531-24_Braconidae_Australia_BOLD_ACD8377 Australian Capital Territory
AUMIC511-18_Apanteles_ippeus_Australia_BOLD_ACD8377 South Australia
AUMIC508-18_Apanteles_ippeus_Australia_BOLD_ACD8377 South Australia
AUMIC1318-24_Braconidae_Australia_BOLD_ACD8377 Western Australia
AUMIC113-18_Apanteles_ippeus_Australia_BOLD_ACD8377 South Australia
AUMIC178-18_Apanteles_ippeus_Australia_BOLD_ACD8377 South Australia
AUMIC287-18_Apanteles_ippeus_Australia_BOLD_ACD8377 South Australia
AUMIC1356-24_Braconidae_Australia_BOLD_ACD8377 Western Australia
AUMIC302-18_Apanteles_ippeus_Australia_BOLD_ACD8377 South Australia
OZBOL382-21_Microgastrinae_Australia_BOLD_ACD8377 South Australia
AUMIC510-18_Apanteles_ippeus_Australia_BOLD_ACD8377 South Australia
AUMIC1298-24_Braconidae_Australia_BOLD_ACD8377 New South Wales
AUMIC355-18_Apanteles_ippeus_Australia_BOLD_ACD8377 South Australia
AUMIC1532-24_Braconidae_Australia_BOLD_ACD8377 Australian Capital Territory
AUMIC1528-24_Braconidae_Australia_BOLD_ACD8377 Australian Capital Territory
AUMIC316-18_Apanteles_ippeus_Australia_BOLD_ACD8377 South Australia
AUMIC295-18_Apanteles_ippeus_Australia_BOLD_ACD8377 South Australia
ASMII8317-22_Apanteles_ippeus_Australia_BOLD_ACD8377 South Australia
OZBOL399-21_Microgastrinae_Australia_BOLD_ACD8377 South Australia
AUMIC805-23_Microgastrinae_Australia_BOLD_ACD8377 Australian Capital Territory
AUMIC1535-24_Braconidae_Australia_BOLD_ACD8377 Australian Capital Territory
AUMIC1538-24_Braconidae_Australia_BOLD_ACD8377 Australian Capital Territory
AUMIC1320-24_Braconidae_Australia_BOLD_ACD8377 Western Australia
AUMIC086-18_Apanteles_ippeus_Australia_BOLD_ACD8377 South Australia
AUMIC314-18_Apanteles_ippeus_Australia_BOLD_ACD8377 South Australia
AUMIC173-18_Apanteles_ippeus_Australia_BOLD_ACD8377 South Australia
ASMII1795-22_Apanteles_ippeus_Australia_BOLD_ACD8377 South Australia
AUMIC313-18_Apanteles_ippeus_Australia_BOLD_ACD8377 South Australia
AUMIC1367-24_Braconidae_Australia_BOLD_ACD8377 Queensland
AUMIC299-18_Apanteles_ippeus_Australia_BOLD_ACD8377 South Australia
AUMIC734-23_Microgastrinae_Australia_BOLD_ADI3291 A. brockhedgesi / MRSB34 South Australia 7/7
AUMIC326-18_Apanteles_Australia_BOLD_ADI3291 South Australia
AUMIC1262-24_Braconidae_Australia_BOLD_ADI3291 South Australia
AUMIC1265-24_Braconidae_Australia_BOLD_ADI3291 South Australia
AUMIC085-18_Apanteles_Australia_BOLD_ADI3291 South Australia
AUMIC865-23_Microgastrinae_Australia_BOLD_ADI3291 South Australia
AUMIC928-23_Microgastrinae_Australia_BOLD_ADI3291 South Australia
AUMIC740-23_Microgastrinae_Australia_BOLD_ADI3291 South Australia
AUMIC352-18_Apanteles_Australia_BOLD_ADI3291 South Australia
AUMIC347-18_Apanteles_Australia_BOLD_ADI3291 South Australia
AUMIC354-18_Apanteles_Australia_BOLD_ADI3291 South Australia
AUMIC1295-24_Braconidae_Australia_BOLD_ADI3291 Western Australia
AUMIC353-18_Apanteles_Australia_BOLD_ADI3291 South Australia
AUMIC651-23_Microgastrinae_Australia_BOLD_AFE7524 A. ramsaris / MRSB37 South Australia 5/7
AUMIC1205-24_Braconidae_Australia_BOLD_AFQ4216 A. insulanus / MRSB36 New South Wales 5/7
AUMIC087-18_Apanteles_Australia_BOLD_AAU8268 A. phantasmatus / MRSB35 Queensland 7/7
AUMIC090-18_Apanteles_Australia_BOLD_AAU8268 Queensland
AUMIC1439-24_Braconidae_Australia_BOLD_AAU8268 Queensland
AUMIC1441-24_Braconidae_Australia_BOLD_AAU8268 Queensland
AUMIC1443-24_Braconidae_Australia_BOLD_AAU8268 Queensland
AUMIC410-18_Apanteles_Australia_BOLD_AAU8268 Queensland
AUMIC1442-24_Braconidae_Australia_BOLD_AAU8268 Queensland
AUMIC1444-24_Braconidae_Australia_BOLD_AAU8268 Queensland
AUMIC071-18_Apanteles_Australia_BOLD_AAU8268 Queensland
NSWHO4562-18_Apanteles_Australia_BOLD_AAU8268 New South Wales
HYAS060-10_Apanteles_Australia_BOLD_AAU8268 New South Wales
AUMIC091-18_Apanteles_Australia_BOLD_AAU8268 Queensland
AUMIC1437-24_Braconidae_Australia_BOLD_AAU8268 Queensland
AUMIC414-18_Apanteles_Australia_BOLD_AAU8268 Queensland
AUMIC1445-24_Braconidae_Australia_BOLD_AAU8268 Queensland
AUMIC413-18_Apanteles_Australia_BOLD_AAU8268 Queensland
AUMIC1185-24_Braconidae_Australia_BOLD_AFR2694 MRSB44 Queensland 7/7
HYQT656-10_Microgastrinae_Australia_BOLD_AAU8509 MRSB46 Queensland 4/4
AUMIC077-18_Apanteles_Australia_BOLD_ADL4428 A. auroralis / MRSB38 Queensland 4/7
AUMIC1523-24_Braconidae_Australia_BOLD_ADL4428 Queensland
AUMIC1526-24_Braconidae_Australia_BOLD_ADL4428 Queensland
AUMIC1525-24_Braconidae_Australia_BOLD_ADL4428 Queensland
AUMIC1524-24_Braconidae_Australia_BOLD_ADL4428 Queensland
AUMIC946-24_Braconidae_Australia_BOLD_AAU8286 A. darthvaderi / MRSB39 New South Wales 5/7
AUMIC1249-24_Braconidae_Australia New South Wales

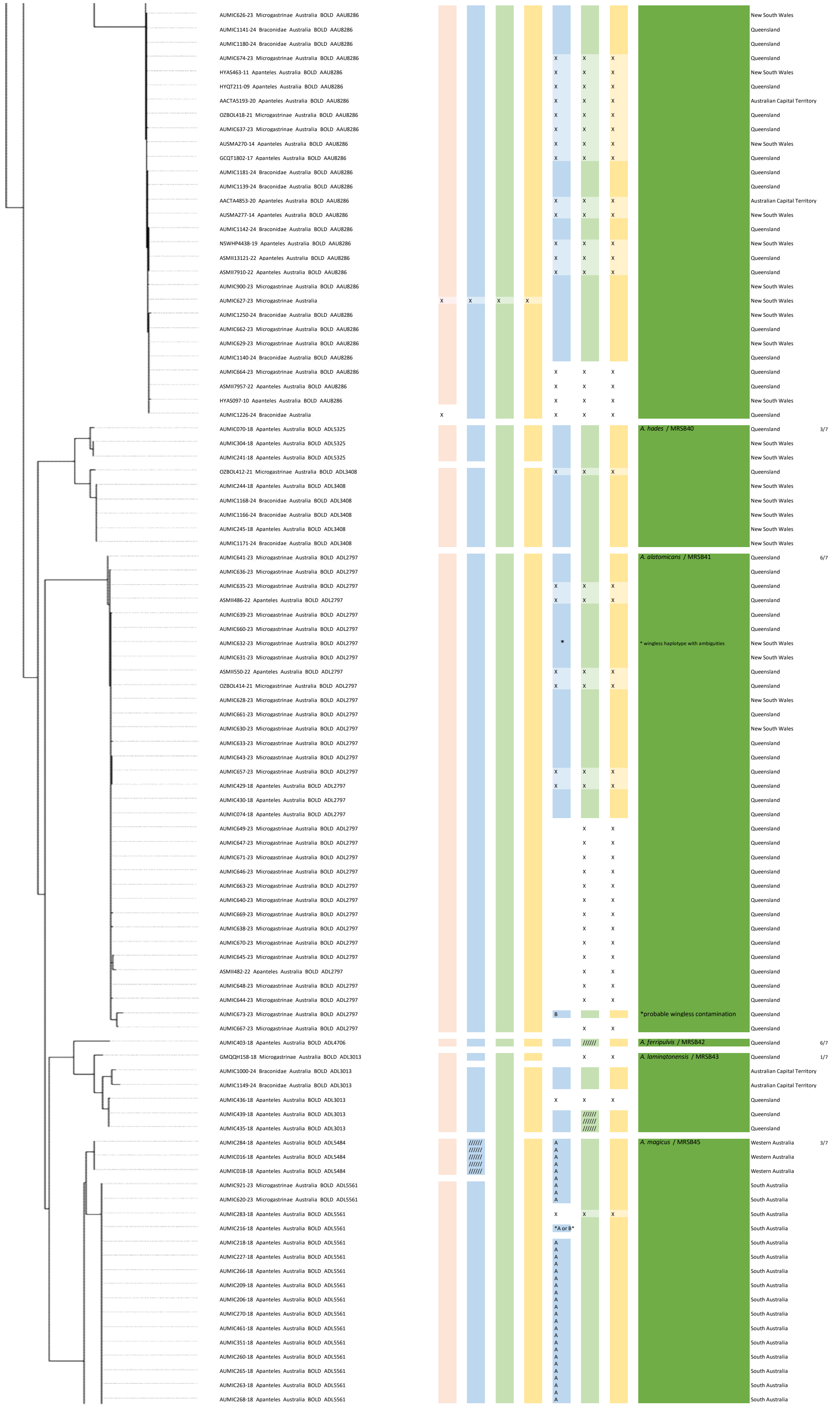
AUMIC626-23 Microgastrinae Australia BOLD AAU8286 New South Wales
AUMIC1141-24 Braconidae Australia BOLD AAU8286 Queensland
AUMIC1180-24 Braconidae Australia BOLD AAU8286 Queensland
AUMIC674-23 Microgastrinae Australia BOLD AAU8286 Queensland
HYAS463-11 Apanteles Australia BOLD AAU8286 New South Wales
HYQT211-09 Apanteles Australia BOLD AAU8286 Queensland
AACTA5193-20 Apanteles Australia BOLD AAU8286 Australian Capital Territory
OZBOL418-21 Microgastrinae Australia BOLD AAU8286 Queensland
AUMIC637-23 Microgastrinae Australia BOLD AAU8286 Queensland
AUSMA270-14 Apanteles Australia BOLD AAU8286 New South Wales
GCQT1802-17 Apanteles Australia BOLD AAU8286 Queensland
AUMIC1181-24 Braconidae Australia BOLD AAU8286 Queensland
AUMIC1139-24 Braconidae Australia BOLD AAU8286 Queensland
AACTA4853-20 Apanteles Australia BOLD AAU8286 Australian Capital Territory
AUSMA277-14 Apanteles Australia BOLD AAU8286 New South Wales
AUMIC1142-24 Braconidae Australia BOLD AAU8286 Queensland
NSWHP4438-19 Apanteles Australia BOLD AAU8286 New South Wales
ASMII3121-22 Apanteles Australia BOLD AAU8286 Queensland
ASMII7910-22 Apanteles Australia BOLD AAU8286 Queensland
AUMIC900-23 Microgastrinae Australia BOLD AAU8286 New South Wales
AUMIC627-23 Microgastrinae Australia New South Wales
AUMIC1250-24 Braconidae Australia BOLD AAU8286 New South Wales
AUMIC662-23 Microgastrinae Australia BOLD AAU8286 Queensland
AUMIC629-23 Microgastrinae Australia BOLD AAU8286 New South Wales
AUMIC1140-24 Braconidae Australia BOLD AAU8286 Queensland
AUMIC664-23 Microgastrinae Australia BOLD AAU8286 Queensland
ASMII7957-22 Apanteles Australia BOLD AAU8286 Queensland
HYAS097-10 Apanteles Australia BOLD AAU8286 New South Wales
AUMIC1226-24 Braconidae Australia Queensland
AUMIC070-18 Apanteles Australia BOLD ADL5325 A. hades / MRSB40 Queensland 3/7
AUMIC304-18 Apanteles Australia BOLD ADL5325 New South Wales
AUMIC241-18 Apanteles Australia BOLD ADL5325 New South Wales
OZBOL412-21 Microgastrinae Australia BOLD ADL3408 Queensland
AUMIC244-18 Apanteles Australia BOLD ADL3408 New South Wales
AUMIC1168-24 Braconidae Australia BOLD ADL3408 New South Wales
AUMIC1166-24 Braconidae Australia BOLD ADL3408 New South Wales
AUMIC245-18 Apanteles Australia BOLD ADL3408 New South Wales
AUMIC1171-24 Braconidae Australia BOLD ADL3408 New South Wales
AUMIC641-23 Microgastrinae Australia BOLD ADL2797 A. alatomicans / MRSB41 Queensland 6/7
AUMIC636-23 Microgastrinae Australia BOLD ADL2797 Queensland
AUMIC635-23 Microgastrinae Australia BOLD ADL2797 Queensland
ASMII486-22 Apanteles Australia BOLD ADL2797 Queensland
AUMIC639-23 Microgastrinae Australia BOLD ADL2797 Queensland
AUMIC660-23 Microgastrinae Australia BOLD ADL2797 Queensland
AUMIC632-23 Microgastrinae Australia BOLD ADL2797 * wingless haplotype with ambiguities New South Wales
AUMIC631-23 Microgastrinae Australia BOLD ADL2797 New South Wales
ASMII550-22 Apanteles Australia BOLD ADL2797 Queensland
OZBOL414-21 Microgastrinae Australia BOLD ADL2797 Queensland
AUMIC628-23 Microgastrinae Australia BOLD ADL2797 New South Wales
AUMIC661-23 Microgastrinae Australia BOLD ADL2797 Queensland
AUMIC630-23 Microgastrinae Australia BOLD ADL2797 New South Wales
AUMIC633-23 Microgastrinae Australia BOLD ADL2797 Queensland
AUMIC643-23 Microgastrinae Australia BOLD ADL2797 Queensland
AUMIC657-23 Microgastrinae Australia BOLD ADL2797 Queensland
AUMIC429-18 Apanteles Australia BOLD ADL2797 Queensland
AUMIC430-18 Apanteles Australia BOLD ADL2797 Queensland
AUMIC074-18 Apanteles Australia BOLD ADL2797 Queensland
AUMIC649-23 Microgastrinae Australia BOLD ADL2797 Queensland
AUMIC647-23 Microgastrinae Australia BOLD ADL2797 Queensland
AUMIC671-23 Microgastrinae Australia BOLD ADL2797 Queensland
AUMIC646-23 Microgastrinae Australia BOLD ADL2797 Queensland
AUMIC663-23 Microgastrinae Australia BOLD ADL2797 Queensland
AUMIC640-23 Microgastrinae Australia BOLD ADL2797 Queensland
AUMIC669-23 Microgastrinae Australia BOLD ADL2797 Queensland
AUMIC638-23 Microgastrinae Australia BOLD ADL2797 Queensland
AUMIC670-23 Microgastrinae Australia BOLD ADL2797 Queensland
AUMIC645-23 Microgastrinae Australia BOLD ADL2797 Queensland
ASMII482-22 Apanteles Australia BOLD ADL2797 Queensland
AUMIC648-23 Microgastrinae Australia BOLD ADL2797 Queensland
AUMIC644-23 Microgastrinae Australia BOLD ADL2797 Queensland
AUMIC673-23 Microgastrinae Australia BOLD ADL2797 B *probable wingless contamination Queensland
AUMIC667-23 Microgastrinae Australia BOLD ADL2797 Queensland
AUMIC403-18 Apanteles Australia BOLD ADL4706 A. ferripulvis / MRSB42 Queensland 6/7
GMQQH158-18 Microgastrinae Australia BOLD ADL3013 A. lamingtonensis / MRSB43 Queensland 1/7
AUMIC1000-24 Braconidae Australia BOLD ADL3013 Australian Capital Territory
AUMIC1149-24 Braconidae Australia BOLD ADL3013 Australian Capital Territory
AUMIC436-18 Apanteles Australia BOLD ADL3013 Queensland
AUMIC439-18 Apanteles Australia BOLD ADL3013 Queensland
AUMIC435-18 Apanteles Australia BOLD ADL3013 Queensland
AUMIC284-18 Apanteles Australia BOLD ADL5484 A. magicus / MRSB45 Western Australia 3/7
AUMIC016-18 Apanteles Australia BOLD ADL5484 Western Australia
AUMIC018-18 Apanteles Australia BOLD ADL5484 Western Australia
AUMIC921-23 Microgastrinae Australia BOLD ADL5561 South Australia
AUMIC620-23 Microgastrinae Australia BOLD ADL5561 South Australia
AUMIC283-18 Apanteles Australia BOLD ADL5561 South Australia
AUMIC216-18 Apanteles Australia BOLD ADL5561 *A or B* South Australia
AUMIC218-18 Apanteles Australia BOLD ADL5561 South Australia
AUMIC227-18 Apanteles Australia BOLD ADL5561 South Australia
AUMIC266-18 Apanteles Australia BOLD ADL5561 South Australia
AUMIC209-18 Apanteles Australia BOLD ADL5561 South Australia
AUMIC206-18 Apanteles Australia BOLD ADL5561 South Australia
AUMIC270-18 Apanteles Australia BOLD ADL5561 South Australia
AUMIC461-18 Apanteles Australia BOLD ADL5561 South Australia
AUMIC351-18 Apanteles Australia BOLD ADL5561 South Australia
AUMIC260-18 Apanteles Australia BOLD ADL5561 South Australia
AUMIC265-18 Apanteles Australia BOLD ADL5561 South Australia
AUMIC263-18 Apanteles Australia BOLD ADL5561 South Australia
AUMIC268-18 Apanteles Australia BOLD ADL5561 South Australia

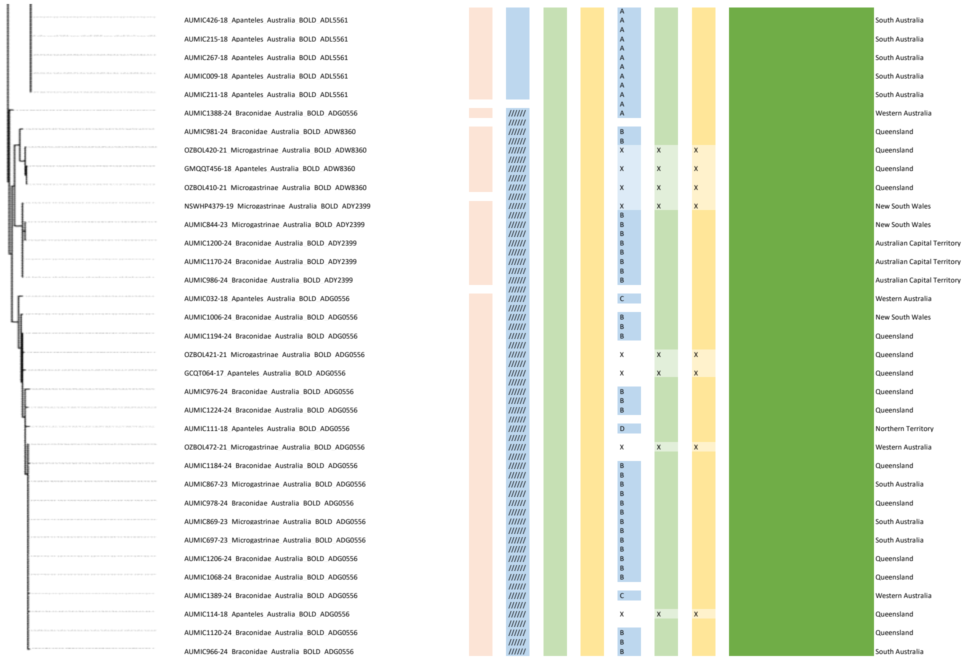
AUMIC426-18_Apanteles_Australia_BOLD_ADL5561 South Australia
AUMIC215-18_Apanteles_Australia_BOLD_ADL5561 South Australia
AUMIC267-18_Apanteles_Australia_BOLD_ADL5561 South Australia
AUMIC009-18_Apanteles_Australia_BOLD_ADL5561 South Australia
AUMIC211-18_Apanteles_Australia_BOLD_ADL5561 South Australia
AUMIC1388-24_Braconidae_Australia_BOLD_ADG0556 Western Australia
AUMIC981-24_Braconidae_Australia_BOLD_ADW8360 Queensland
OZBOL420-21_Microgastrinae_Australia_BOLD_ADW8360 Queensland
GMQQT456-18_Apanteles_Australia_BOLD_ADW8360 Queensland
OZBOL410-21_Microgastrinae_Australia_BOLD_ADW8360 Queensland
NSWHP4379-19_Microgastrinae_Australia_BOLD_ADY2399 New South Wales
AUMIC844-23_Microgastrinae_Australia_BOLD_ADY2399 New South Wales
AUMIC1200-24_Braconidae_Australia_BOLD_ADY2399 Australian Capital Territory
AUMIC1170-24_Braconidae_Australia_BOLD_ADY2399 Australian Capital Territory
AUMIC986-24_Braconidae_Australia_BOLD_ADY2399 Australian Capital Territory
AUMIC032-18_Apanteles_Australia_BOLD_ADG0556 Western Australia
AUMIC1006-24_Braconidae_Australia_BOLD_ADG0556 New South Wales
AUMIC1194-24_Braconidae_Australia_BOLD_ADG0556 Queensland
OZBOL421-21_Microgastrinae_Australia_BOLD_ADG0556 Queensland
GCQT064-17_Apanteles_Australia_BOLD_ADG0556 Queensland
AUMIC976-24_Braconidae_Australia_BOLD_ADG0556 Queensland
AUMIC1224-24_Braconidae_Australia_BOLD_ADG0556 Queensland
AUMIC111-18_Apanteles_Australia_BOLD_ADG0556 Northern Territory
OZBOL472-21_Microgastrinae_Australia_BOLD_ADG0556 Western Australia
AUMIC1184-24_Braconidae_Australia_BOLD_ADG0556 Queensland
AUMIC867-23_Microgastrinae_Australia_BOLD_ADG0556 South Australia
AUMIC978-24_Braconidae_Australia_BOLD_ADG0556 Queensland
AUMIC869-23_Microgastrinae_Australia_BOLD_ADG0556 South Australia
AUMIC697-23_Microgastrinae_Australia_BOLD_ADG0556 South Australia
AUMIC1206-24_Braconidae_Australia_BOLD_ADG0556 Queensland
AUMIC1068-24_Braconidae_Australia_BOLD_ADG0556 Queensland
AUMIC1389-24_Braconidae_Australia_BOLD_ADG0556 Western Australia
AUMIC114-18_Apanteles_Australia_BOLD_ADG0556 Queensland
AUMIC1120-24_Braconidae_Australia_BOLD_ADG0556 Queensland
AUMIC966-24_Braconidae_Australia_BOLD_ADG0556 South Australia
