# Supplementary figures and images for: ﻿DNA barcoding, integrative taxonomy, citizen science, and Bush Blitz surveys combine to reveal 34 new species of Apanteles (Hymenoptera, Braconidae, Microgastrinae) in Australia
Source: Zookeys. 2025 Feb 11;1227:1–128. doi: 10.3897/zookeys.1227.130467 (PMC11836623; doi:10.3897/zookeys.1227.130467)

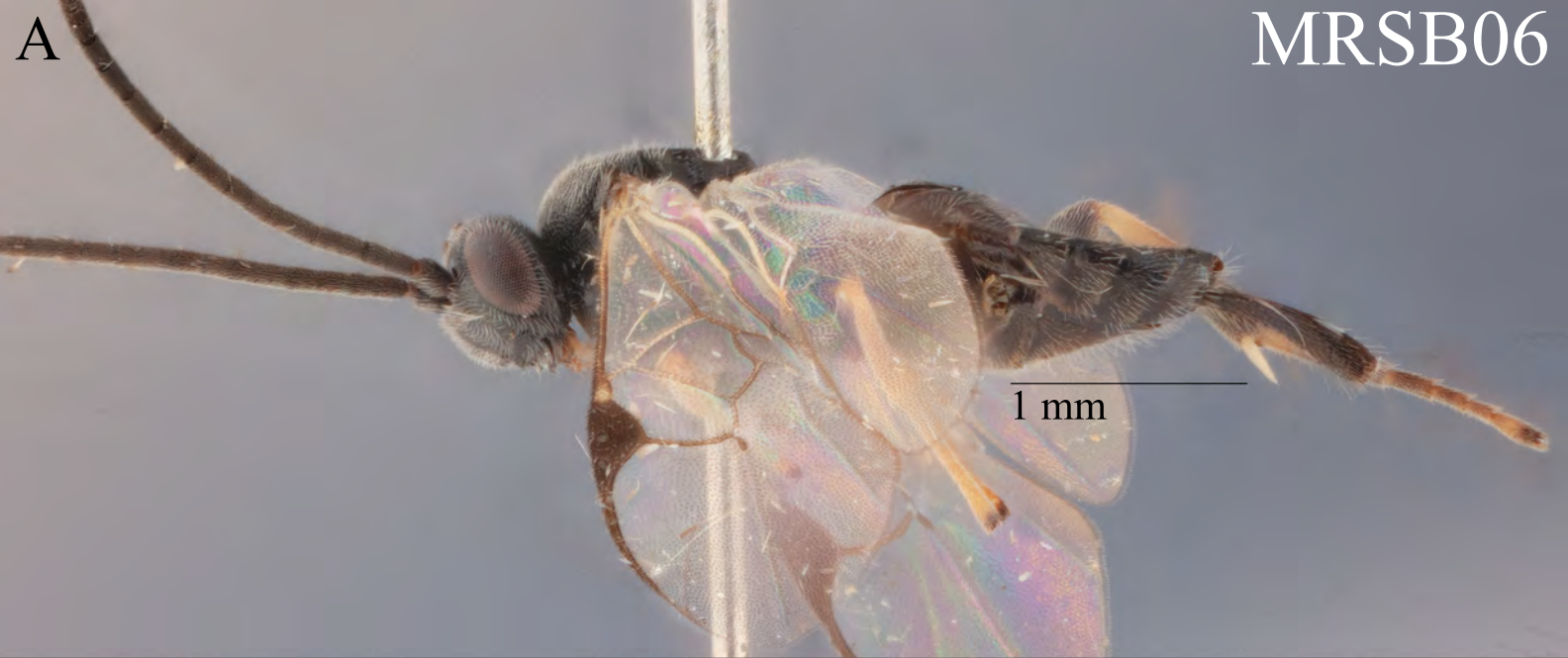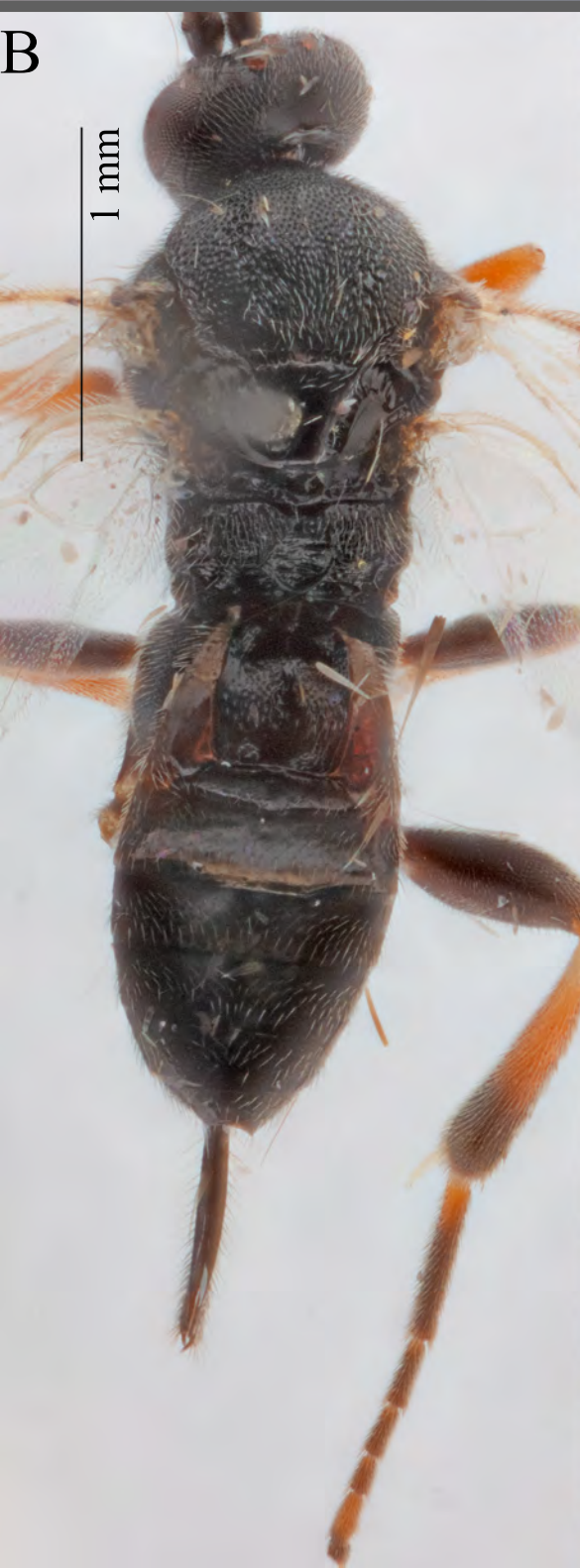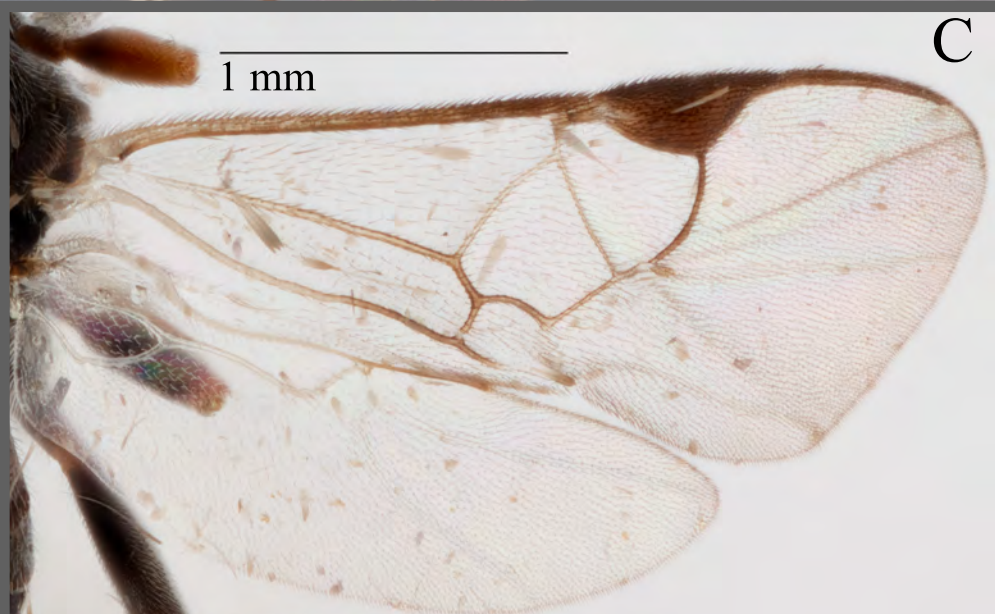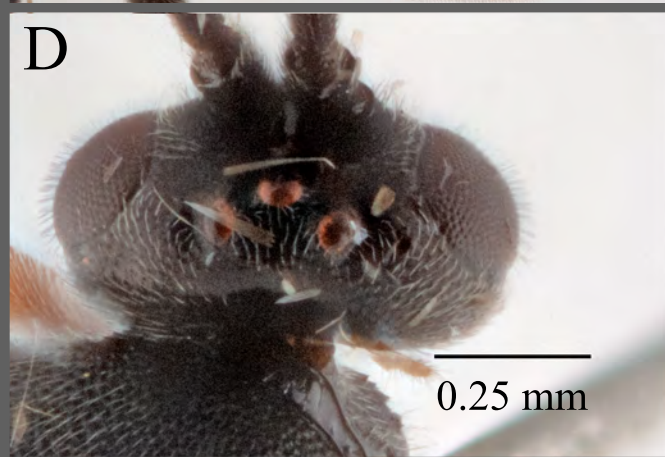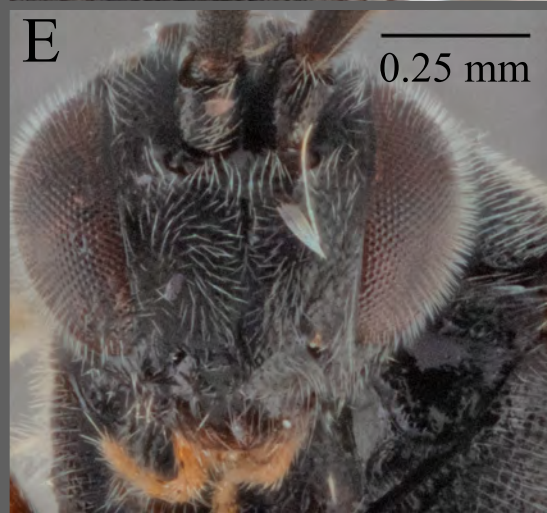

A

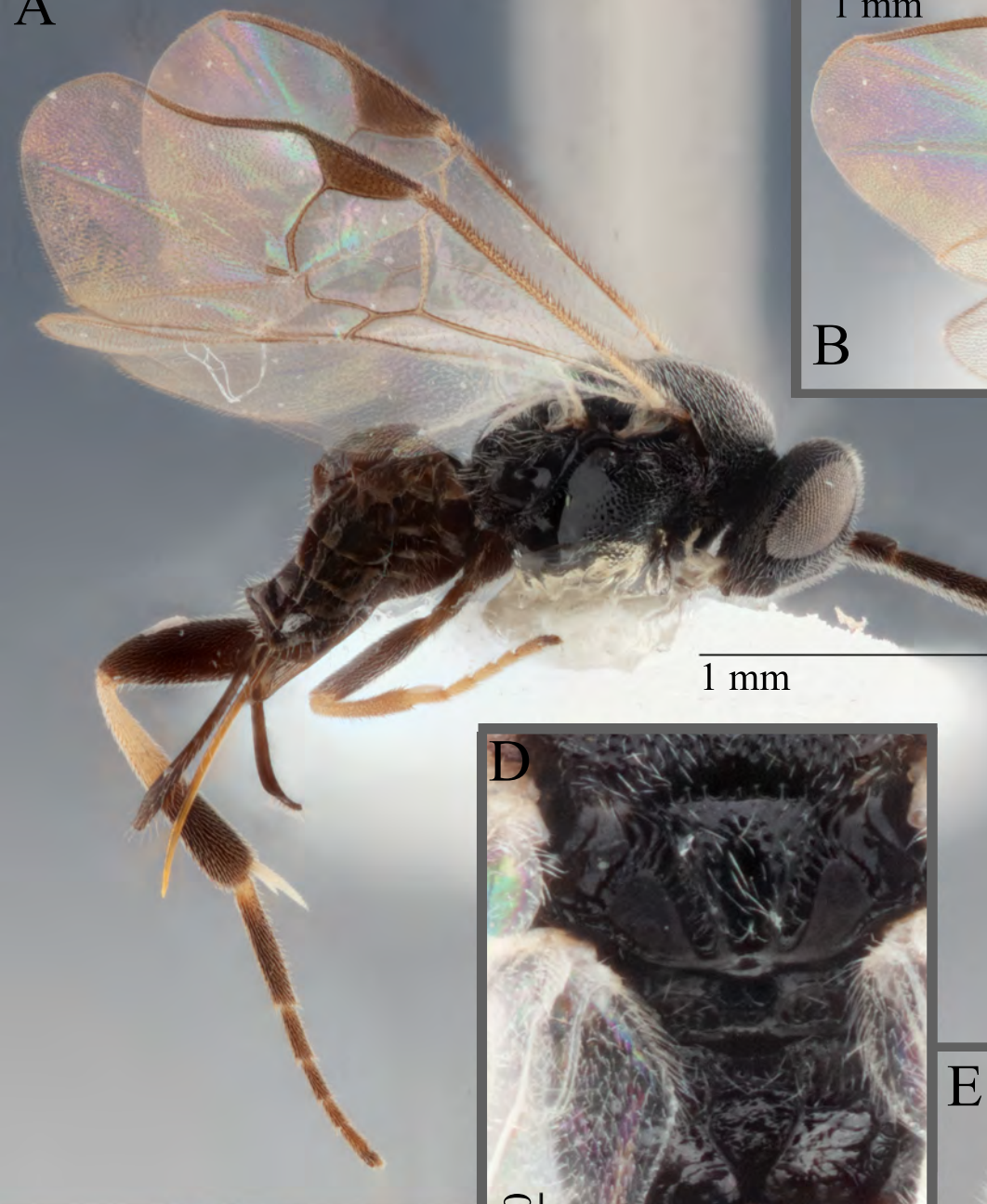

1 mm

MRSB07

B

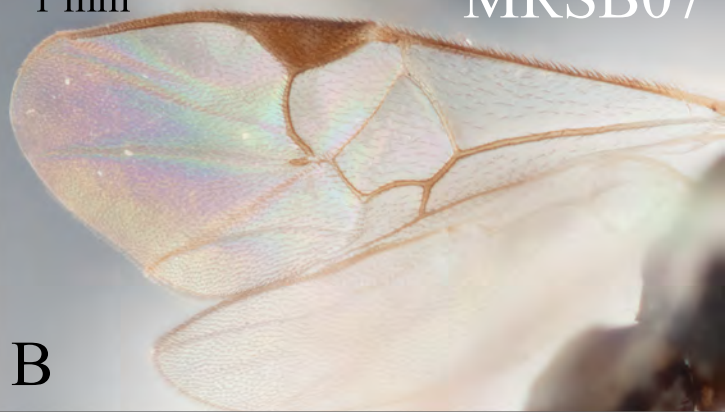

D

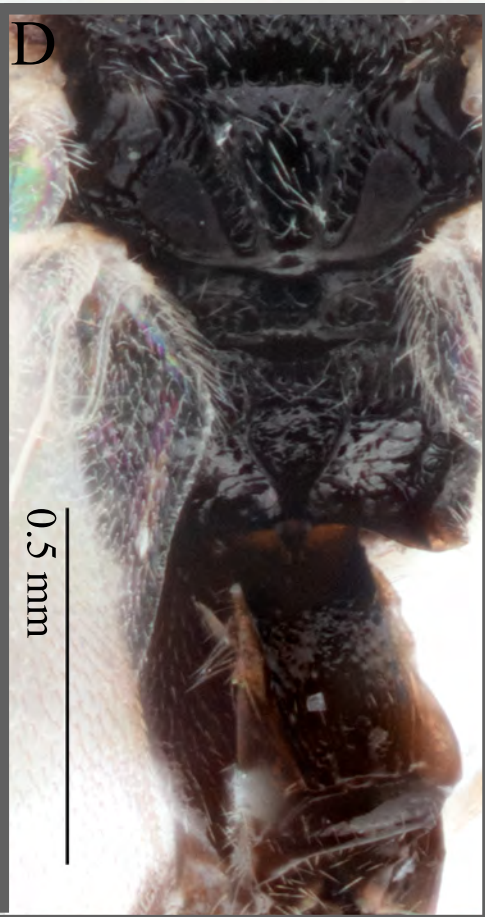

0.5 mm

E

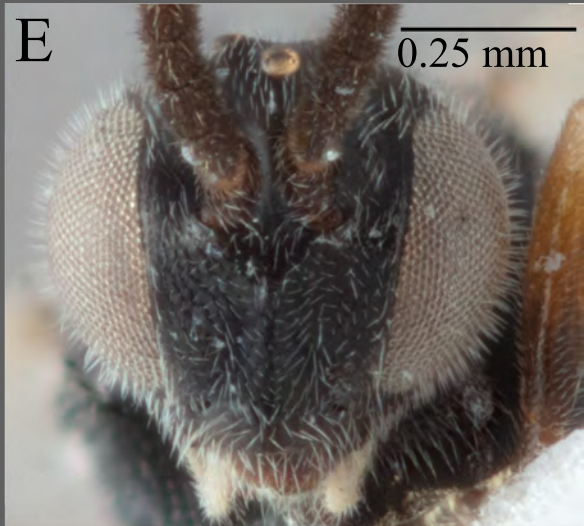

0.25 mm

C

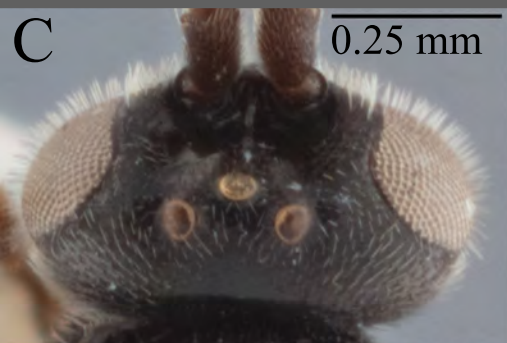

0.25 mm

F

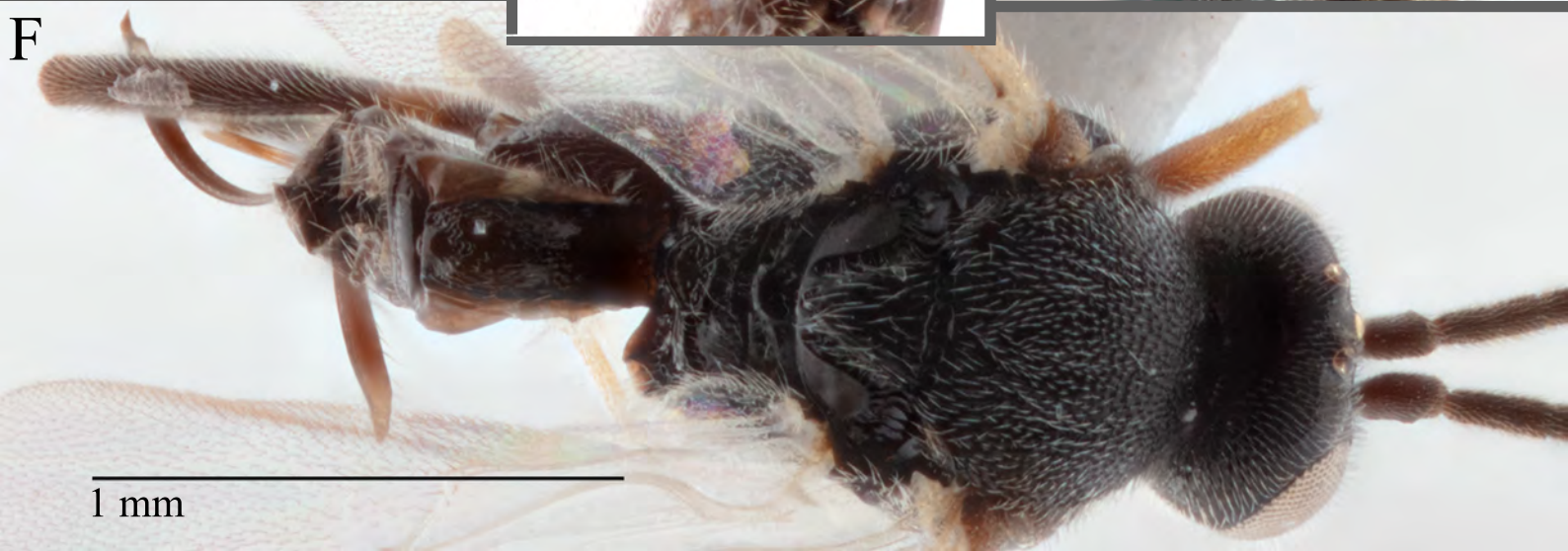

1 mm

A MRSB08

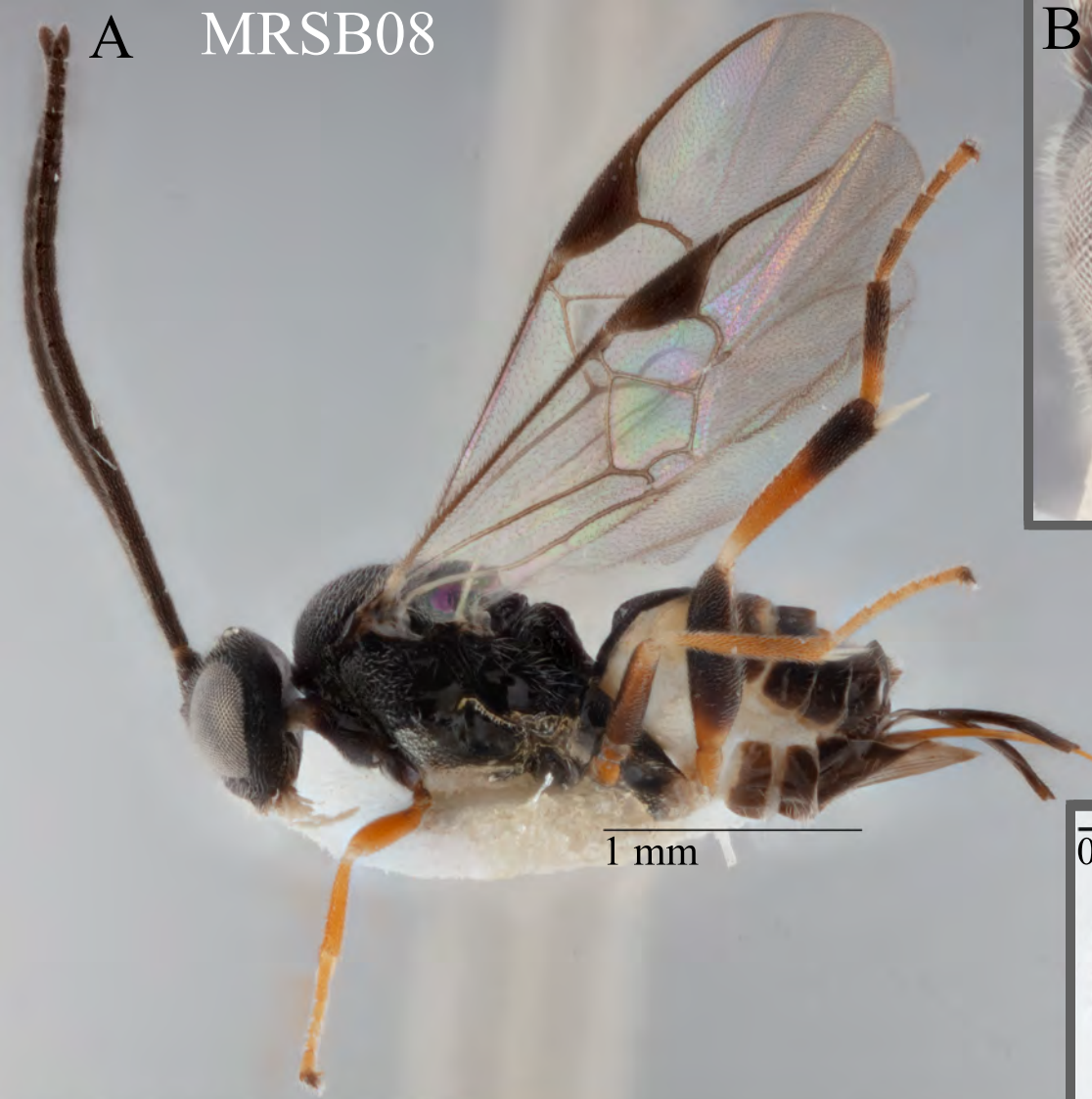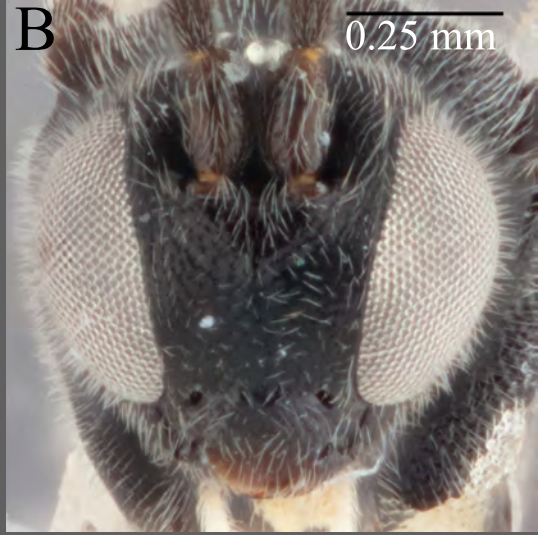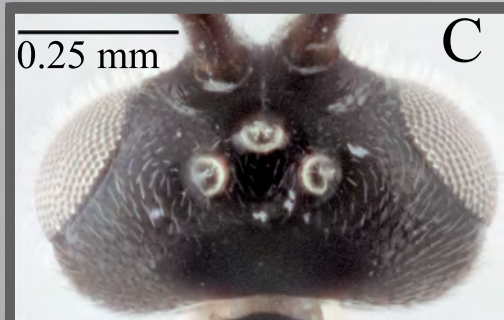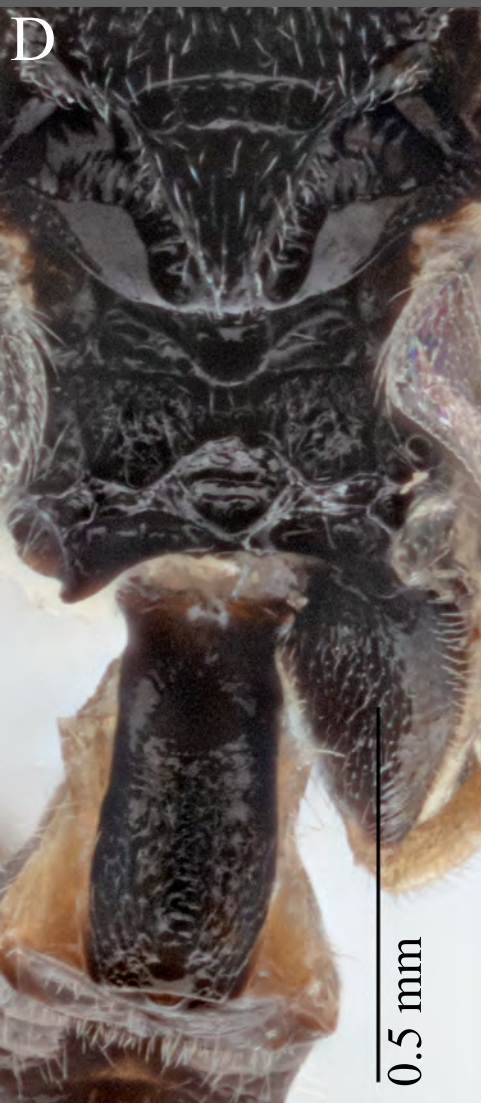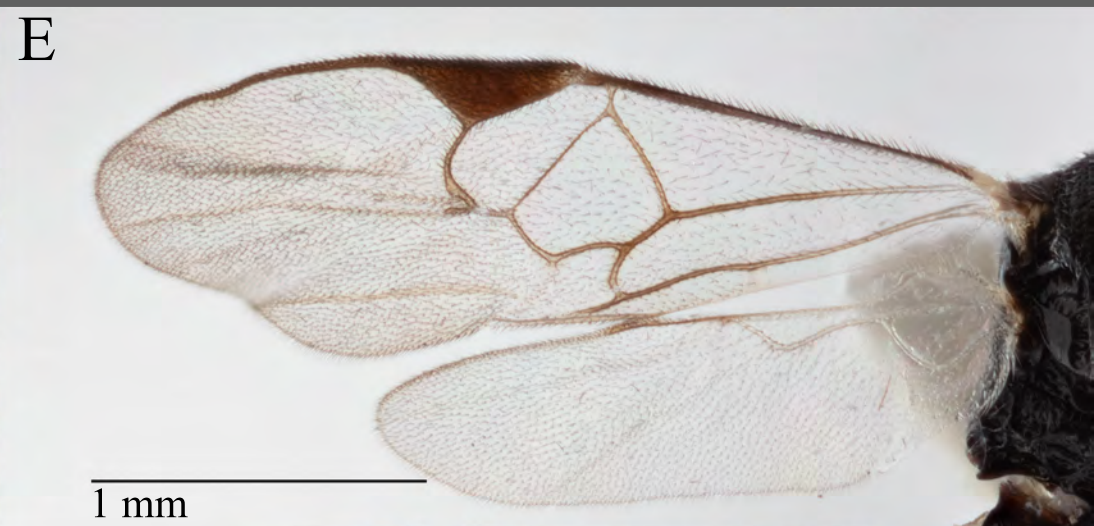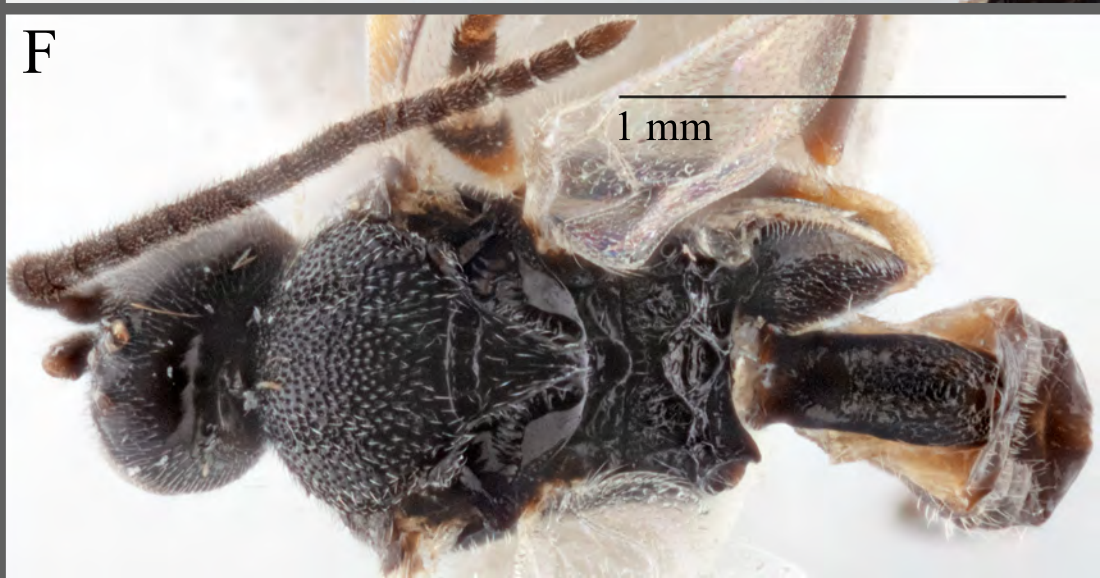

A

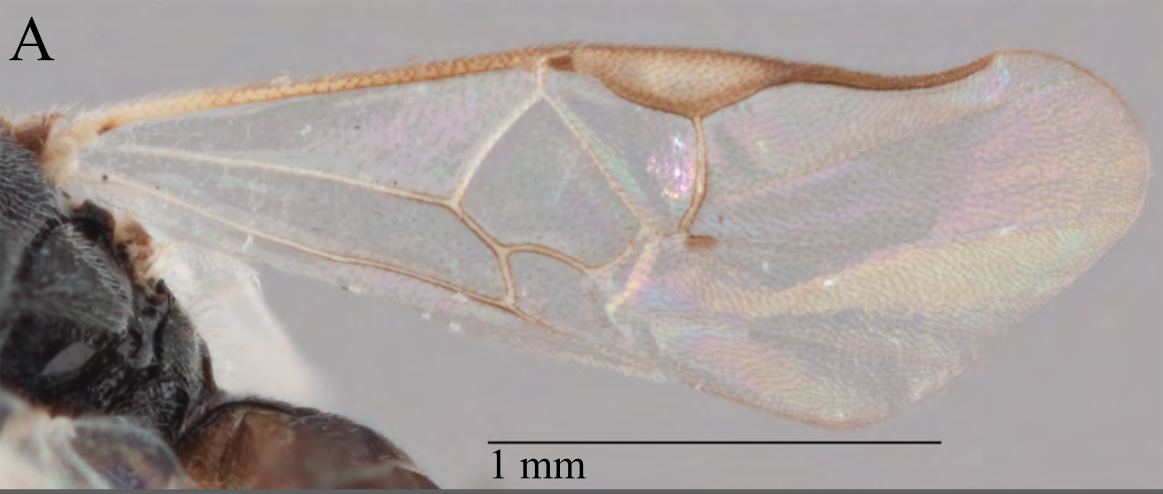

MRSB19

B

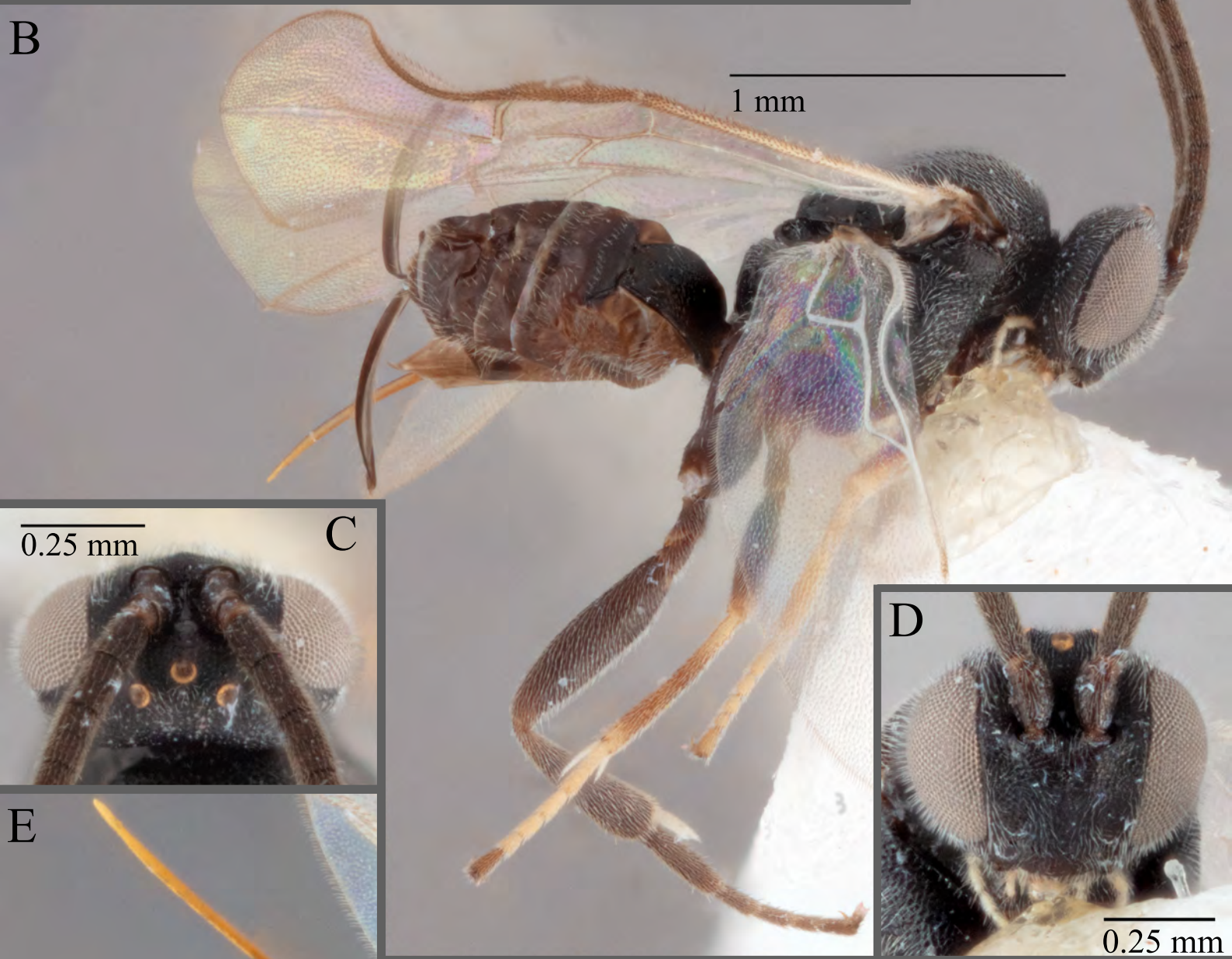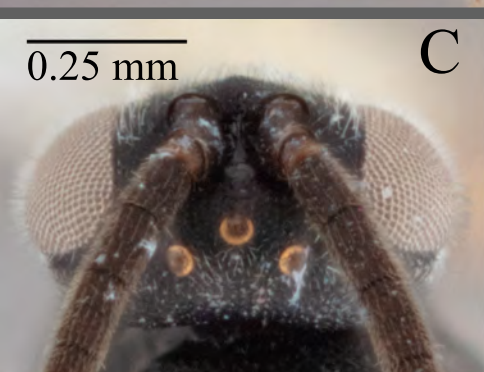

C

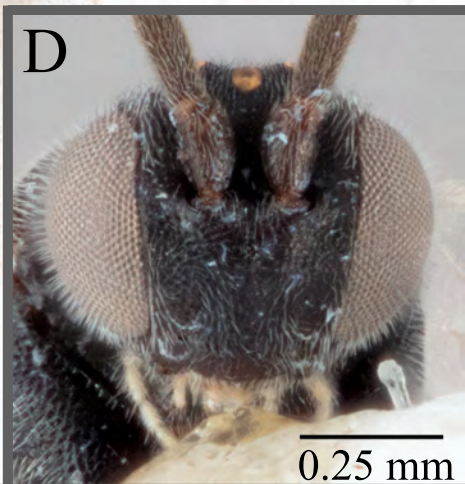

D

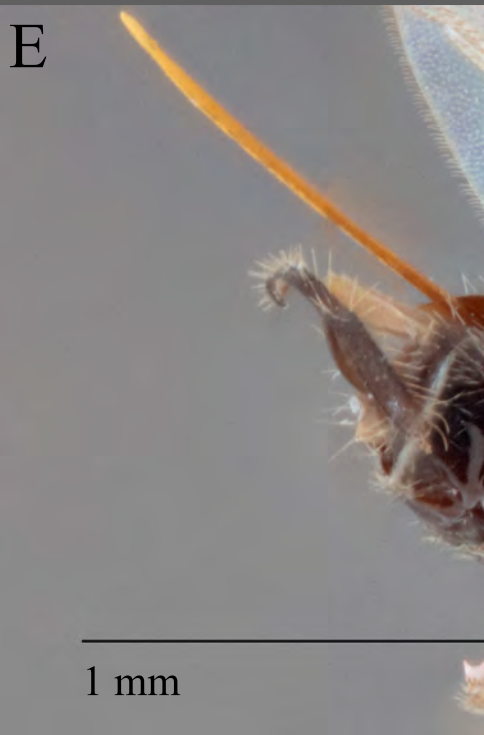

E

Supplement: Supplementary material 2 — Images [file zookeys-1227-001_article-130467__-s002.pdf]
